# Isolation and Identification of Isocoumarin Derivatives With Specific Inhibitory Activity Against Wnt Pathway and Metabolome Characterization of *Lasiodiplodia venezuelensis*

**DOI:** 10.3389/fchem.2021.664489

**Published:** 2021-08-12

**Authors:** Léonie Pellissier, Alexey Koval, Laurence Marcourt, Emerson Ferreira Queiroz, Nicole Lecoultre, Sara Leoni, Luis-Manuel Quiros-Guerrero, Morgane Barthélémy, Bastiaan L. Duivelshof, Davy Guillarme, Sébastien Tardy, Véronique Eparvier, Karl Perron, Jérôme Chave, Didier Stien, Katia Gindro, Vladimir Katanaev, Jean-Luc Wolfender

**Affiliations:** ^1^ School of Pharmaceutical Sciences, University of Geneva, CMU, Geneva, Switzerland; ^2^ Institute of Pharmaceutical Sciences of Western Switzerland, University of Geneva, CMU, Geneva, Switzerland; ^3^ Department of Cell Physiology and Metabolism, Translational Research Centre in Oncohaematology, Faculty of Medicine, University of Geneva, CMU, Geneva, Switzerland; ^4^ Mycology Group, Research Department Plant Protection, Agroscope, Nyon, Switzerland; ^5^ Microbiology Unit, Department of Botany and Plant Biology, University of Geneva, Geneva, Switzerland; ^6^ Université Paris-Saclay, CNRS, Institut de Chimie des Substances Naturelles, Gif-sur-Yvette, France; ^7^ CNRS, Biological Diversity and Evolution (UMR 5174), Toulouse, France; ^8^ Sorbonne Université, CNRS, Laboratoire de Biodiversité et Biotechnologie Microbiennes, LBBM, Observatoire Océanologique, Banyuls-Sur-Mer, France; ^9^ School of Biomedicine, Far Eastern Federal University, Vladivostok, Russia

**Keywords:** natural product, Wnt pathway inhibitors, anticancer, Lasiodiplodia venezuelensis, endophytic fungi, bio-guided fractionation, isocoumarins, molecular networking

## Abstract

The Wnt signaling pathway controls multiple events during embryonic development of multicellular animals and is carcinogenic when aberrantly activated in adults. Breast cancers are dependent on Wnt pathway overactivation mostly through dysregulation of pathway component protein expression, which necessitates the search for therapeutically relevant compounds targeting them. Highly diverse microorganisms as endophytes represent an underexplored field in the therapeutic natural products research. In the present work, the objective was to explore the chemical diversity and presence of selective Wnt inhibitors within a unique collection of fungi isolated as foliar endophytes from the long-lived tropical palm *Astrocaryum sciophilum*. The fungi were cultured, extracted with ethyl acetate, and screened for their effects on the Wnt pathway and cell proliferation. The endophytic strain *Lasiodiplodia venezuelensis* was prioritized for scaled-up fractionation based on its selective activity. Application of geometric transfer from analytical HPLC conditions to semi-preparative scale and use of dry load sample introduction enabled the isolation of 15 pure compounds in a single step. Among the molecules identified, five are original natural products described for the first time, and six are new to this species. An active fraction obtained by semi-preparative HPLC was re-purified by UHPLC-PDA using a 1.7 µm phenyl column. 75 injections of 8 µg were necessary to obtain sufficient amounts of each compound for structure elucidation and bioassays. Using this original approach, in addition to the two major compounds, a third minor compound identified as (*R*)-(-)-5-hydroxymellein (18) was obtained, which was found to be responsible for the significant Wnt inhibition activity recorded. Further studies of this compound and its structural analogs showed that only 18 acts in a highly specific manner, with no acute cytotoxicity. This compound is notably selective for upstream components of the Wnt pathway and is able to inhibit the proliferation of three triple negative breast cancer cell lines. In addition to the discovery of Wnt inhibitors of interest, this study contributes to better characterize the biosynthetic potential of *L. venezuelensis*.

## Introduction

The ever-increasing threat of cancer is one of the greatest challenges of modern medicine (Kunnumakkara et al., 2019). The current paradigm in anticancer drug development is that of targeted therapies, i.e., agents specifically targeting highly specific cancer pathways and components, thus minimizing toxic effects on healthy tissues (Ke and Shen, 2017).

While higher plants and soil microbes are being extensively explored as a source of new bioactive chemical entities, exploring uncommon microbial communities may increase the chances of finding such compounds (Zhu et al., 2012). In that respect, efforts have been recently turned towards the rarely encountered microorganisms inhabiting the tissues of higher plants, notably, bacteria, and fungi called endophytes (Stone et al., 2000). Endophytes are described as a fascinating group of microorganisms that reside in living plant tissues (beneath the epidermal cell layer) during a considerable part of their life cycle, without causing any symptoms (Petrini et al., 1991; Schulz et al., 2006). They occupy millions of unique biological niches, growing in biodiverse environments; almost all plant species studied until now have been found to host at least one to hundreds of endophytes (Arnold et al., 2000). Those microorganisms are thus considered as an underexploited reservoir of genetic and chemical diversity and novelty (Schulz et al., 2002). In a co-evolutionary perspective, the host plant provides nutrition and structural protection to the endophytes, which in return help enhance the fitness and resistance of the host to adversity by producing functional secondary metabolites (Zhang et al., 2006; Martin et al., 2017). According to Tan et al., they possess a resistance mechanism to overcome pathogenic invasion and are considered unmatchable “chemical synthesizers” acting as producers of a wide range of pharmacologically active substances (Tan and Zou, 2001). Their residency in the host plant for a substantial period prompts a constant chemical innovation with potentially reduced toxicity towards the host cells, notably eukaryotic cells, making them a unique resource notably for discovering anticancer molecules (Omeje et al., 2017). Following the discovery that the Food and Drug Administration approved anticancer agent Taxol, originally isolated from *Taxus brevifolia*, was also produced by endophytic fungi such as *Taxomyces andreanae*, numerous endophytes have been found to be producers of potent anticancer agents (Stierle et al., 1995; Kusari et al., 2009).

The Wnt pathway is an intracellular signal transduction cascade that is activated in a highly specific and orchestrated manner during the embryonic development of multicellular animal organisms, from sponges to humans (Holstein, 2012). In mammals, the pathway plays an additional important role in adult tissue homeostasis through controlling the tissue regeneration and renewal (Ring et al., 2014; Steinhart and Angers, 2018). This multitude of activities controlled by the activated Wnt pathway provides a significant benefit for the tumor cells of different cancer types, most notably triple-negative breast cancer (TNBC), colorectal and hepatocellular carcinomas, lung, and ovarian cancers, by endorsing their proliferation, migration, and metastasis formation (Zhan et al., 2017).

The most prominent and well-described role in cancer belongs to the so-called canonical branch of Wnt signaling. In this branch, the signals emanating from the binding of secreted Wnt proteins to their cognate GPCRs Frizzled receptors are transduced intracellularly through G proteins and the phosphoprotein Dishevelled (Dvl) (Koval and Katanaev, 2011). This action results in inhibition of the so-called “destruction complex”—an assembly of proteins Axin, APC, and GSK3beta, which in the absence of signal efficiently phosphorylates the cytoplasmic pool of beta-catenin, mediating its proteasomal degradation. Upon the arrival of the Wnt signal, the destruction complex becomes inactive and beta-catenin accumulates in the cytoplasm and subsequently penetrates the nucleus, where it serves as a transcriptional co-factor to the TCF/LEF family of the proteins (Koval et al., 2011). The transcriptional program initiated this way by Wnt ligands includes several hundred genes and provides a major boost for cell proliferation (Koval and Katanaev, 2018).

However, direct targeting of the Wnt pathway by acting on its most widespread components has detrimental effects on the Wnt-dependent healthy tissues resulting in poorly manageable adverse effects and failure to advance in clinical trials (Shaw et al., 2019a). More selective Wnt-targeting agents are thus required, targeting only the components essential for cancers (Blagodatski et al., 2014; Kahn, 2014). Such components are naturally more abundant in the upstream parts of the pathway, with the diversity of the Wnt ligand and Frizzled receptor orthologs differentially expressed in the tissues being particularly attractive (Shaw et al., 2019b).

In the search for new natural products targeting the Wnt pathway, we sought to investigate cultivated communities of endophytic fungi in model plants known for their long-life cycle and possibly hosting steady microbial communities over many years.

In this context, we focused on a collection of foliar endophytic fungi isolated from the host model *Astrocaryum sciophilum* (Miq.) Pulle. *A. sciophilum* is a solitary, understory palm endemic to the northeast region of the Amazon basin (Amapá and Pará states of Brazil, French Guiana, Guyana, Suriname) (Kahn, 2008). Its remarkable maturation age of around 170 years makes it a long-lived, resistant species, with leaves that can be up to 20 years old (Charles-Dominique et al., 2003). This suggests that endophytes residing in its leaves can persist for a considerable time and can be resistant to environmental menace. They might have developed antipathogens and cytotoxic metabolites involved in the longevity of the shoots and leaves of the palm (Arnold et al., 2003). Moreover, the highly competitive rainforest ecosystem makes the selection pressure intense, probably prompting microbes associated with a tropical host to produce potent secondary metabolites (Rosa et al., 2011).

Previous work on this plant model has shown, in another collection of endophytes, that it contained some cytotoxic and antimicrobial strains in which some bioactive compounds have been identified (Barthélemy et al., 2019; Donald et al., 2019; Barthélemy et al., 2020). In the present work, a collection of fungi isolated as cultivable endophyte strains from the leaves of *A. sciophilum* were cultured, extracted, and screened for their effects on cell proliferation and Wnt pathway inhibition. Among them, the endophytic strain *Lasiodiplodia venezuelensis* Burgess, Barber, and Mohali (Burgess et al., 2006) was prioritized for scaled-up fractionation for its selective activity and taxonomical originality.

The genus *Lasiodiplodia* belongs to the phylum Ascomycota, class Dothideomycetes, order Botryospheaeriales, and family Botryiosphaeriaceae (Robert et al., 2005). Members of this family, as the *Lasiodiplodia* spp., have been found on a large variety of plant hosts as important pathogens of several woody perennial plants, causing diseases as cankers, blights, and rots and dieback symptoms (Correia et al., 2016; Vieira et al., 2018; Li et al., 2019). They can also survive as saprophytes and/or endophytes within seeds and living tissues, in a latent phase, not causing any symptoms (Smith et al., 1996). *Lasiodiplodia* species are common mostly in tropical and subtropical regions (De Wet et al., 2008). *L. venezuelensis* was first described by Burgess et al. in 2006 (Burgess et al., 2006) in the living wood tissues of *Acacia mangium* Willd. in Venezuela and later on by Urbez-Torres et al. from *Pinus caribaea* var. *hondurensis* Barret and Golfari (Úrbez-Torres et al., 2016). This species was also associated with a blue stain of wood in Venezuelan lumber yards (Mohali et al., 2017). Since they were re-isolated from asymptomatic wood and did not cause any lesions when re-inoculated, they are considered endophyte or latent pathogens. In comparison with other *Lasiodiplodia* spp., *L. venezuelensis* seems to have a restricted distribution since it has not been reported elsewhere in the world to date (Mohali et al., 2017).

In the frame of the present study, the anti-Wnt metabolites are characterized, and detailed profiling of this strain is provided to document its metabolite composition.

## Results and Discussion

### Biological Activities of Fungal Extracts

A collection of 15 strains isolated as cultivable endophytes from the leaves of *A. sciophilum* were cultured and extracted with ethyl acetate (EtOAc). The extracts were then partitioned with water (W). All extracts were screened for their effects on Wnt pathway inhibition. The specificity was controlled by analyzing general cytotoxicity. As a system to screen compounds for the potential anti-Wnt signaling activities, we used the BT-20 TNBC cells, stably transfected with the TopFlash reporter construct, responding by luciferase expression to stimulation with purified Wnt3a (Koval et al., 2014). EtOAc and W extracts of fungi were tested at different concentrations in this assay. Only the extracts exhibiting either no cytotoxicity or at least a cytotoxic IC_50_ 3-fold higher than the Wnt-specific one were considered as those with selective Wnt inhibition. Among them, the EtOAc extract of the strain A02 identified as *Lasiodiplodia venezuelensis* exhibited a clear specific inhibitory activity (Wnt IC_50_ of 180 ± 56 μg/ml) and low cytotoxicity (Renilla inhibition IC_50_ > 500 μg/ml (estimated)). Based on the bioactivity results and a preliminary metabolite profiling revealing an interesting composition (see section *Deep Metabolome Analysis*), the A02 strain was prioritized and cultivated on a large scale (100 14 cm diameter Petri dishes) and extracted with the same procedure as for the screening. The large-scale EtOAc extract (A02Et) was then tested in an independent series of measurements and confirmed the anti-Wnt activity observed on a small scale (IC_50_ of 27 ± 6 μg/ml, cytotoxic IC_50_ of 143 ± 9 μg/ml).

### Bioactivity-Guided Fractionation and Isolation

A first chemical profile of the bioactive A02Et extract by HPLC-PDA-ELSD revealed the presence of a significant amount of lipophilic compounds as shown by ELSD. In order to rule out a possible interference of lipid compounds in the Wnt assay, the EtOAc extract was further simplified by liquid/liquid partitioning hexane/methanol:H_2_O (7:3). This process yielded an apolar hexane fraction (A02EtH) and an enriched hydroalcoholic fraction (A02EtM) (Supplementary Figure 1). The specific anti-Wnt activity was only exhibited by A02EtM extract, which was enriched in more polar secondary metabolites (Wnt IC_50_ of 13 ± 3.5 μg/ml; cytotoxic IC_50_ of 97 ± 16 μg/ml).

**FIGURE 1:**
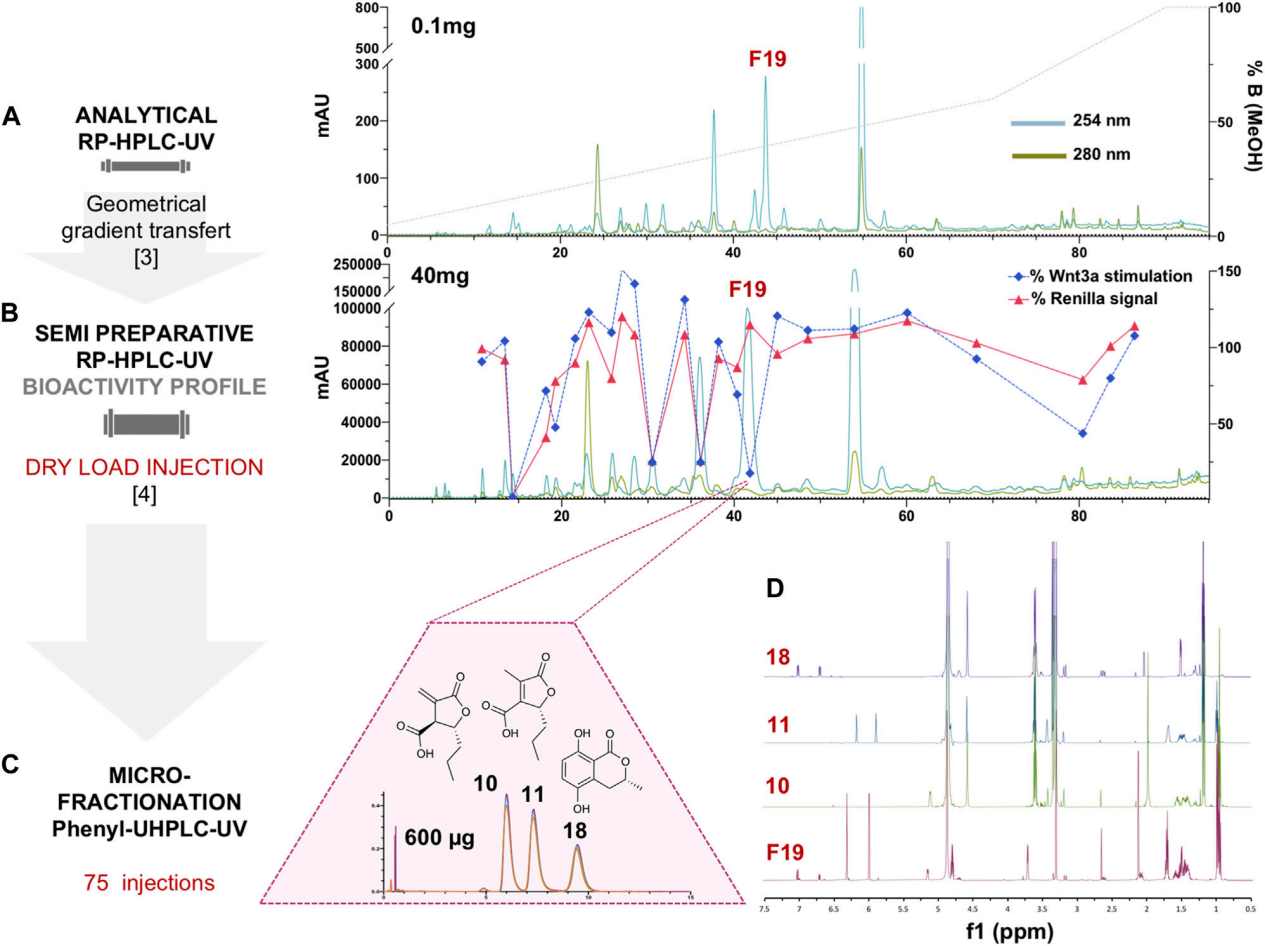
**(A)** Analytical HPLC-PDA profile of the enriched hydroalcoholic fraction of *L. venezuelensis* extract (A02EtM). **(B)** Semi-preparative HPLC-UV profile of the fraction A02EtM with an overlay of the Wnt inhibition bioactivity profile of fractions collected by semi-preparative fractionation. Bioactivity values of each fraction at 33 μg/ml are expressed as a response to Wnt3a stimulation in % of control and response of Renilla luciferase in % of control (cytotoxicity). The fraction named F19 was the only one to show specific anti-Wnt activity, while other fractions were cytotoxic and the decrease of Wnt signal was due to this effect. **(C)** UHPLC-UV microfractionation of F19 repeated 75 times on a phenyl column for enhanced chromatographic selectivity. **(D)** Comparison of the ^1^H-NMR spectra of F19 and the isolated compounds 10, 11, and 18.

For efficient isolation of the active compounds, the reverse phase HPLC-PDA-ELSD conditions were optimized in order to obtain an efficient separation (Figure 1A). Those conditions were transferred to semi-preparative HPLC using a geometric gradient transfer method to obtain similar chromatographic selectivity (Guillarme et al., 2008). In order to avoid loss of resolution, the extract was introduced by dry load, following a protocol recently developed in our laboratory (Queiroz et al., 2019). The high-resolution HPLC semi-preparative fractionation of the extract provided a good baseline separation of most compounds detected by UV (Figure 1B and Supplementary Figure 2). This efficient process yielded thirty-three fractions containing mainly single HPLC peaks that were directly submitted to the Wnt inhibition bioassays (Supplementary Figure 3). The results of the assay were displayed as a bioactivity profile (% of control activity at 33 μg/ml), which highlighted the chromatographic zone of activity (Figure 1B). The F19 fraction was the only one to show specific anti-Wnt activity, while other fractions were cytotoxic, with the decrease of the Wnt signal due to this effect. Fraction F19 Wnt IC_50_ was 15 ± 3.9 μg/ml, and cytotoxic IC_50_ was >200 μg/ml**.**

The A02EtM and the F19 fraction were profiled by UHPLC-PDA-HRMS/MS using data-dependent acquisition mode followed by metabolite annotation to estimate its chemical composition. The detailed procedure for metabolite annotation is described in the following section (*Deep Metabolome Analysis*).

A careful analysis of the HRMS and HRMS/MS spectra of the active fraction F19 in the chemical profile of the extract revealed that it contained molecular ions corresponding to two distinct molecules (Supplementary Figure 4). The first feature at *m/z* 185.0809 [M + H]^+^ and 183.0661 [M−H]^−^ indicated a compound with a molecular formula of C_9_H_12_O_4_ (calcd. for C_9_H_13_O_4_^+^, 185.0808, Δppm = 0.30 and C_9_H_11_O_4_^−^, 183.0663, Δppm = 1.09), corresponding to 4 degrees of unsaturation. In MS1 and MS/MS, this ion was dereplicated as decumbic acid, already isolated from *Lasiodiplodia theobromae* (He et al., 2004). The second ion at *m/z* 195.0652 [M + H]^+^ and 193.0505 [M−H]^−^ indicated a compound with a molecular formula of C_10_H_10_O_4_ (calcd. for C_10_H_11_O_4_^+^, 195.0652, Δppm = 0.00 and C_10_H_9_O_4_^−^, 193.0506, Δppm = 0.52), corresponding to 6 degrees of unsaturation. In MS1, this ion was dereplicated as 3,4-dihydro-4,8-dihydroxy-3-methyl-1H-2-benzopyran-1-one (4-hydroxymellein), also isolated from *Lasiodiplodia theobromae* (Salvatore et al., 2020)*.* MS/MS dereplication against the DNP-ISDB, an in-house database containing the *in silico* fragmentation spectra of all the compounds present in the Dictionary of Natural Products (DNP), annotated the compound corresponding to this feature as 3,4-dihydro-3,8-dihydroxy-3-methyl-1H-2-benzopyran-1-one, which differed in the hydroxylation pattern. This indicated that this feature was an hydroxymellein derivative, but its exact structure could not be asserted at this level.

This fraction was also profiled by NMR, which revealed that it was composed of two isomers along with a minor compound with a ratio of 1 (40%)/1.3 (50%)/ 0.4 (10%) moles. The main signals in the NMR spectrum corresponded to decumbic acid, as suggested by the MS/MS annotation. However, signals at δ_H_ 6.32 and 6.00 (*J* = 2.8 Hz) corresponding to an exocyclic double bond that replaced the methyl at δ_H_ 2.13 of the decumbic acid (11), and an additional methine at δ_H_ 3.72 (dt, *J* = 5.6, 2.8 Hz) revealed that an isomer of decumbic acid with a close structure was also present (10). The minor signals in this fraction seem to correspond partly to the hydroxymellein derivative (18) dereplicated by MS/MS, but a full *de novo* identification could not be made at this stage (Figure 1D).

In order to identify which metabolite(s) was responsible for the Wnt inhibition, careful purification was needed since none of them was described as an anti-Wnt inhibitor.

Several analytical HPLC conditions were tested but did not enable a baseline separation of the three constituents. Further UHPLC-PDA profiling of this fraction using an analytical phenyl column packed with 1.7 µm silica beads finally yielded an efficient separation of the three constituents (Figure 1C). This challenging separation could not be upscaled and a microfractionation strategy had to be devised directly on the UHPLC platform at the analytical scale.

Since the loading capacity of the UHPLC column was very limited, a series of 75 injections of 8 µg each was performed (Figure 1C). All separations exhibited well-aligned profiles, allowing efficient multiple collections of each of the three peaks (Supplementary Figure 5). This led to the purification of the three compounds of fraction F19. The two major isomers were obtained at 94 and 83 μg, and the minor constituent was obtained at 29 µg (Figures 1C,D). Since only minute amounts could be obtained at the UHPLC scale, they were quantified during NMR analysis for a precise concentration assignment for the bioassays.

The two major compounds were confirmed to be two lactone derivatives, (3*S*,4*R*)-3-carboxy-2-methylene-heptan-4-olide (10), a toxin responsible for the decay of tropical fruit, and its inactive derivative, decumbic acid (11), isolated from *L. theobromae* (He et al., 2004)*.* The third minor compound was finally identified as (*R*)-(-)-5-hydroxymellein (18). Melleins are a subgroup of 3,4-dihydrocoumarins that belong to the subgroup of isocoumarins, a well-known polyketides family. Such derivatives are produced by bacteria, fungi, higher plants, insects, lichens, and marine sponges, the most important source of mellein being the fungi. They have shown a wide range of biological activities (Reveglia et al., 2020). They were mainly reported as phytotoxins produced by Botryosphaeriaceae species (Cimmino et al., 2017; Reveglia et al., 2019). (–)-(*R*)-Mellein (3*R*,4*S*) and (3*R*,4*R*)-4-hydroxymellein and (3*R*)-5-hydroxymellein have already been isolated from *Lasiodiplodia sp*. 5-Hydroxymellein has been already isolated from *Lasiodiplodia theobromae* but never reported in *L. venezuelensis* (Salvatore et al., 2020). It has also been isolated from the endophytic fungus *Aspergillus flocculus*, inhibiting the growth of K562 cancer cell lines (Tawfike et al., 2019). The NMR analysis of 18 unambiguously confirmed its hydroxylation and configuration pattern, which could not be inferred by dereplication.

**FIGURE 2:**
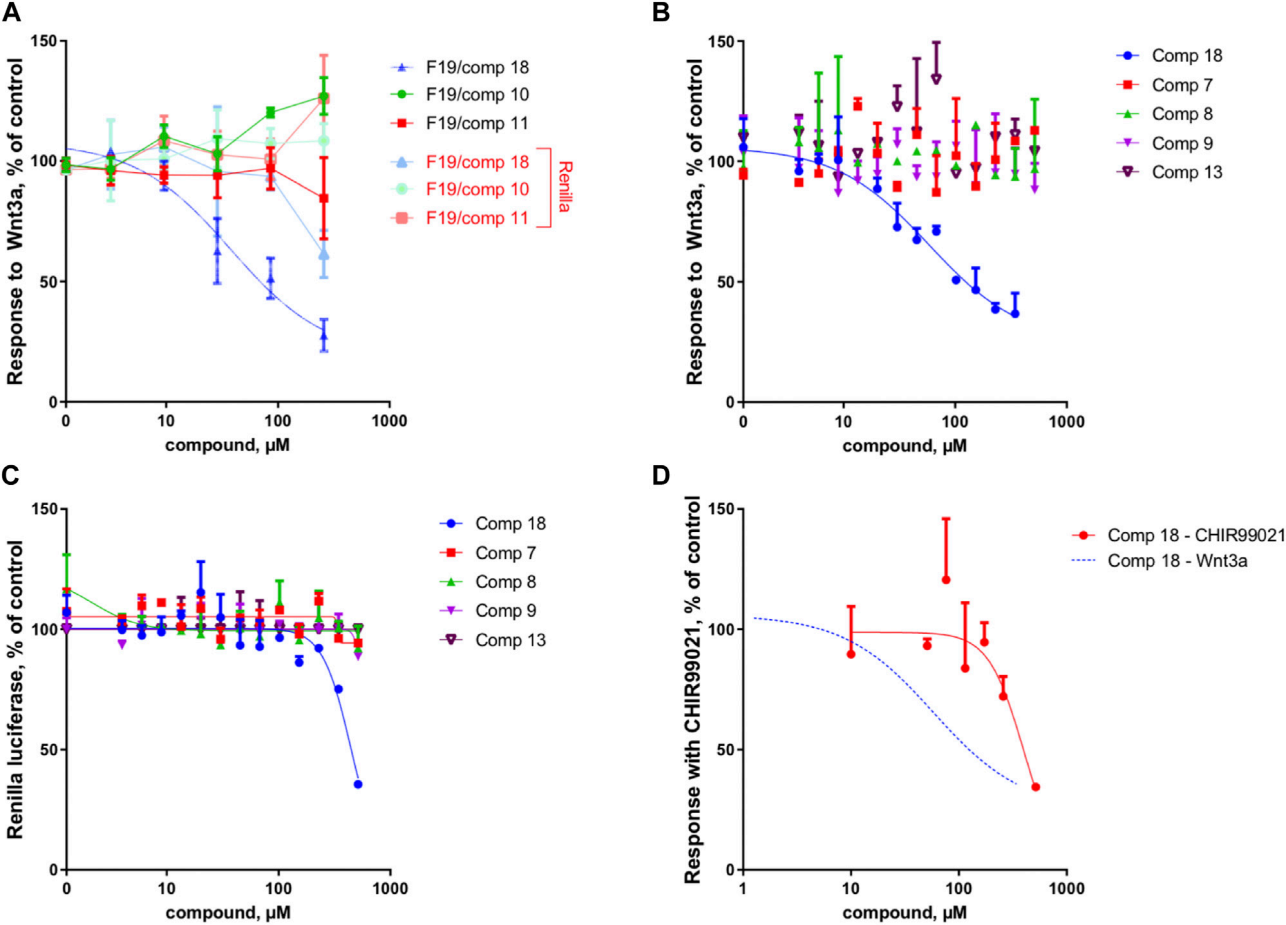
Compound 18 mediates the Wnt inhibition activity at the upstream level of the pathway. **(A)** Response to Wnt3a stimulation, in % of control and response of Renilla luciferase in % of control (cytotoxicity) of the three constituents of fraction 19 (10, 11, and 18). In the fraction F19, only 18 is able to inhibit Wnt activity, with cytotoxicity occurring only at high concentrations. **(B)** Response to Wnt3a stimulation, in % of control of 18 (re-isolated as a second batch) compared to isolated analogs (7, 8, 9, and 13). Only 18 is able to inhibit Wnt activity. **(C)** Response of Renilla luciferase in % of control (cytotoxicity) obtained in the same experiment as B. Cytotoxicity occurs only at high concentrations with compound 18. **(D)** Response with CHIR99021 stimulation, in % of control. Comparison of Wnt3a- and CHIR99021-induced stimulation of the TopFlash reporter. Inhibition by 18 is highly selective for upstream components of the pathway since the inhibition of downstream activator CHIR99021 occurs only at very high concentrations and is thus unspecific (for comparison, the Wnt3a fit curve of panel B is shown).

Because of the small amount obtained, we took advantage of the NMR analysis to precisely quantify each of the three compounds in view of the bioassays. Those final bioassays clearly demonstrated that the third minor compound (*R*)-(-)-5-hydroxymellein (18) was responsible for the significant Wnt inhibition activity of the fraction F19, with a Wnt IC_50_ of 6.7 ± 1.2 μg/ml (58 ± 16 µM) (cytotoxic IC_50_ > 20 μg/ml (>500 µM)) (Figure 2A).

### Chemical Characterization of Other Secondary Metabolites From the Hydroalcoholic Extract (A02EtM)

The first chemical profiling of the extract by UHPLC-HRMS/MS indicated a potentially interesting chemical composition of the ethyl acetate extract of *L. venezuelensis*. Moreover, the metabolome of this particular species was not described in the literature. In order to document the composition of this strain and understand its chemodiversity, we took advantage of having the UHPLC-HRMS/MS metabolite profiling and the NMR analysis of the high-resolution semi-preparative HPLC fractions to combine this information for a deep metabolome characterization. Furthermore, we highlighted a cluster of potential analogs of the active 5-hydroxymellein 18 and exploited the possibility of isolating them to further assess any structure-activity relationship (see section *Deep Metabolome Analysis*).

### *De Novo* Structure Identification of All Pure Fractions by NMR

**FIGURE 3:**
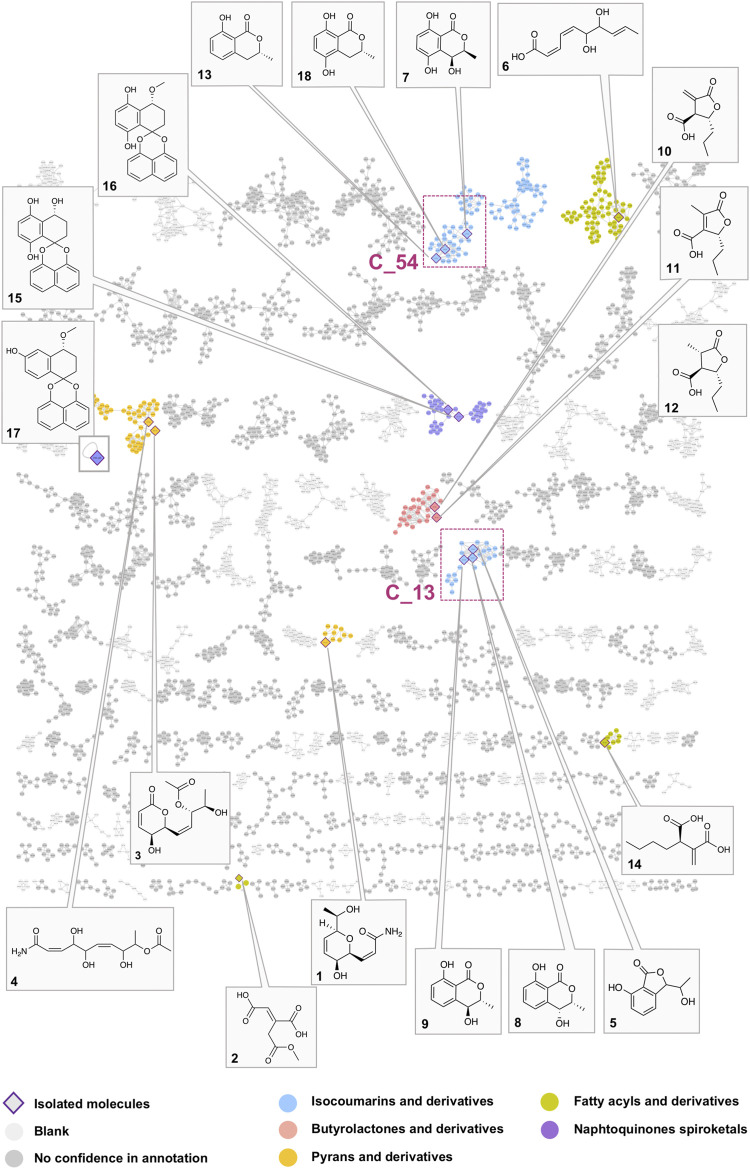
Global molecular network of the A02EtM extract and the 33 semi-preparative fractions with attribution of the nodes of all pure compounds isolated from the HPLC semi-preparative purification and their corresponding clusters. Colors of the nodes correspond to chemical families tentatively assigned to the clusters through the use of a consensus structural annotation at different Classyfire taxa levels. Clusters C_54 and C_13, highlighted with red squares, are annotated as benzopyran derivatives (C_54 with the active compound 18 and isolated analogs 7 and 13) (C_13 with compounds 5, eight and 9). A detailed view of the selected clusters is displayed in Figure 6.

From the 33 fractions obtained from semi-preparative HPLC semi-preparative purification, fifteen pure compounds (1–15) were successfully isolated in a single step (Figure 3). Ten compounds were identified by comparison with the literature as aconitate B (2) (F7) (Cai et al., 2001), 3ξ-(1ξ-hydroxyethyl)-7-hydroxy-1-isobenzofuranone (mixture of two isomers) (5) (F10) (Rahman and Gray, 2005), (+)-(3R,4S)-4,5-dihydroxymellein (7) (F13) (Meepagala et al., 2018), (–)-(3R,4R)-4-hydroxymellein (8) (F14) (Djoukeng et al., 2009), (–)-(3R,4S)-4-hydroxymellein (9) (F16) (Cimmino et al., 2017), decumbic acid B (12) (F20) (Zhou et al., 2016), (–)-(R)-mellein (13) (F22) (Djoukeng et al., 2009), (2R)-butylitaconic acid (14) (F24) (Nakahashi et al., 2009), the enantiomers of dihydroxynaphtalene derivatives CJ-12,372 (15) (F26) (Sakemi et al., 1995), and palmarumycin EG1 (16) (F29) (Macías-Rubalcava et al., 2014). All compounds were described as fungal metabolites, but only compounds 8, 9, and 13 were already described in the Botryosphaeriaceae family. In addition, five other compounds are original natural products, and their full *de novo* structure identification is described hereafter.

**FIGURE 4:**
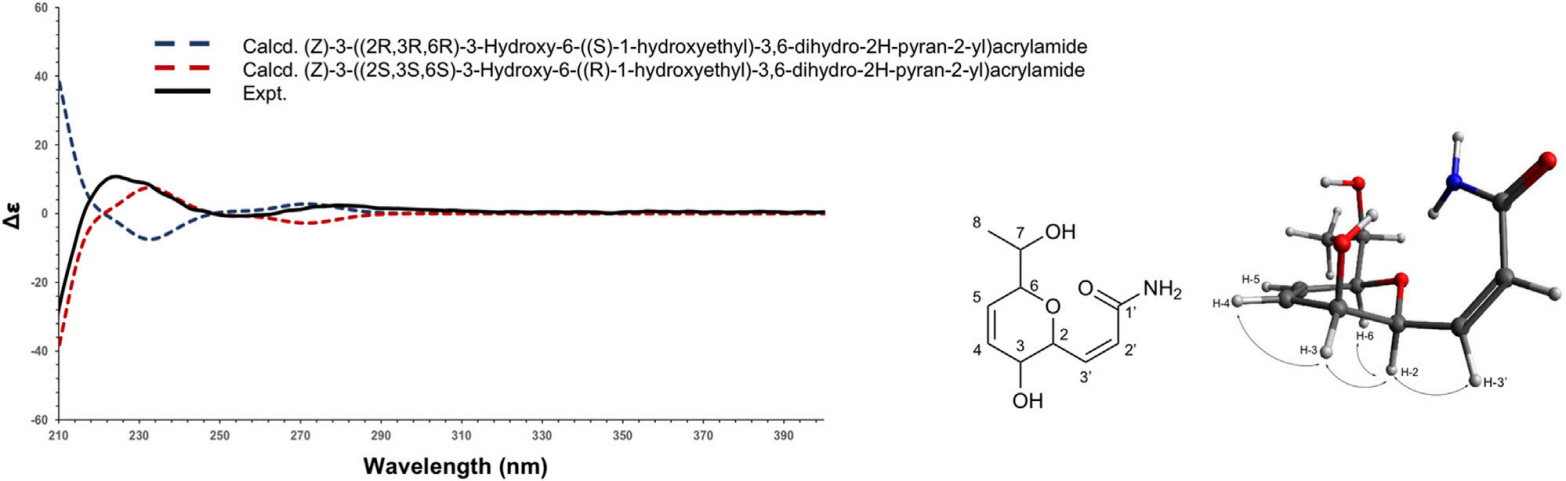
Experimental (black) and TFT-TD-calculated ECD spectra of the two enantiomers (2*R*,3*R*,6*R*), ((*S*)-1-hydroxyethyl) (blue), and (2*S*,3*S*,6*S*), ((*R*)-1-hydroxyethyl) (red) of compound 1.

Compound 1 (F6) was isolated as an oil. The ESI(+)-HRMS experiment indicated a molecular formula of C_19_H_15_NO_4_ with a *m/z* of 214.1071 [M + H]^+^ (calcd. for C_10_H_16_NO_4_^+^, 214.1074, Δppm = 1.40), which corresponded to 4 degrees of unsaturation. Analysis of the ^1^H, ^13^C and HSQC NMR data of 1 indicated the presence of a dihydropyran scaffold consisting of two olefinic carbons at δ_C_ 135.0 (C-3) and 123.3 (C-4) and three oximethines at δ_C_ 80.6 (C-2), 71.5 (C-5), 66.2 (C-6). In addition to these signals, a methyl group at δ_H_ 1.19 (3H, d, *J* = 6.4 Hz, H-8), an oximethine at δ_H_ 3.71 (1H, qd, *J* = 6.4, 5.8 Hz, H-7), two olefinic protons at δ_H_ 7.00 (1H, dd, *J* = 9.8, 5.8 Hz, H-1') and δ_H_ 6.18 (1H, d, *J* = 9.8 Hz, H-2'), and an ester carbonyl at δ_C_ 165.4 (C-3') were observed. Sequential COSY correlations from H-6 at δ_H_ 4.17 (1H, dd, *J* = 5.8, 2.8 Hz) to H-5 (δ_H_ 4.74), H-4 (δ_H_ 6.10), and H-3 (δ_H_ 6.32) and HMBC correlations from the olefinic proton H-4 at δ_H_ 6.10 (1H, ddd, *J* = 10.4, 5.2, 2.3 Hz) to C-2 and C-6 and from the oximethine proton H-6 to C-5 and C-2 allowed defining a 3,6-dihydro-*2H*-pyran-3-ol ring. The COSY correlations from H-1' to H-2' and H-6 and HMBC correlations from H-1' and H-2' to the ester carbonyl C-3' positioned a propenamide side chain at C-6. The 9.8 Hz coupling constant of the set of olefinic protons H-1' and H-2' demonstrated the *cis* configuration of the double bond on this side chain. Correlation from the methyl protons H-8 to the oximethine C-7 (δ_C_ 69.9) and C-2 located a 1-hydroxyethyl group in C-2. Further, COSY correlations from H-7 to H-8 and H-2 supported the position of this second side chain. The ROESY correlations from H-6 to H-5 and H-2 and the small coupling constant between H-5 and H-6 (*J =* 2.8 Hz) indicated the relative configuration of the 3,6-dihydro-*2H*-pyran-3-ol ring (Supplementary Figures 6–11). To establish the absolute configuration of compound 1, the ECD spectrum was calculated based on the relative configuration proposed by NMR and compared to the experimental data (Figure 4). After the comparison, three stereocenters of the 6,6-dihydro-*2H*-pyran-3-ol were all deduced as (*R*) configurations. To define the absolute configuration of C-7, the *S*-MTPA and *R*-MTPA ester derivatives were synthesized from the (*R*)- and (*S*)-α-methoxy-α-trifluoromethylphenylacetyl chloride, respectively (Supplementary Figure 12) (Hoye et al., 2007). The downfield shift of the methyl H-8 and the upfield shift of H-2 in the *R*-MTPA ester relative to the *S*-MTPA ester were consistent with a C-7 (*R*) configuration (Supplementary Figures 13–18). After comparison, 1 was assigned as the new molecule (2*Z*)-3[(1*R*,2*R*,5*R*)-2-hydroxy-5[(1*R*)-1-hydroxyethyl]cyclohex-3-en-1-yl]prop-2-enamide.

Compound 3 (F8) was isolated as an oil. The ESI(+)-HRMS experiment indicated a molecular formula of C_12_H_16_O_6_ with a *m/z* of 257.1020 [M + H]^+^ (calcd. for C_12_H_17_O_6_^+^, 257.1020, Δppm = 0.00), which corresponded to 5 degrees of unsaturation. Analysis of the ^1^H-NMR and HSQC data of 3, as this was the case for 1 indicated the presence of a dihydropyranone scaffold consisting of two olefinic carbons at δ_C_ 121.1 (C-3) and 146.1 (C-4) two oximethines carbons at δ_C_ 60.8 (C-5) and 76.1 (C-6). In addition to these signals, two olefinic protons at δ_H_ 5.90 (1H, t, *J =* 11.1, 9.2 Hz, H-1') and δ_H_ 5.71 (1H, ddd, *J =* 11.1, 9.3, 1.1 Hz, H-2'), two oximethines protons at δ_H_ 5.29 (1H, dd, *J =* 9.3, 3.1 Hz, H-3') and at δ_H_ 3.77 (1H, qdd, *J =* 6.4, 5.3, 3.1 Hz, H-4'), two methyl groups at δ_H_ 1.05 (3H, d, *J =* 6.4 Hz, H-5') and δ_H_ 2.01 (3H, s, H-7'), and two protons from hydroxy groups at δ_H_ 5.60 (1H, d, *J =* 7.1 Hz, OH5) and at δ_H_ 4.87 (1H, d, *J =* 5.3 Hz, OH4’). Analysis of the HMBC spectrum revealed correlations from the olefinic proton H-3 to the ester carbonyl C-2 at δ_C_ 162.9 and to C-5 and from the second olefinic proton H-4 to C-5, C-6, and C-2. Furthermore, COSY correlations between the olefinic protons H-3 and H-4 and from H-4 to H-5 defined the dihydropyranone moiety. COSY correlation from H-5 to the hydroxyl group at δ_H_ 5.60 placed the first hydroxy group in position C-5. Sequential COSY correlations from H-6 to H-1', H-2', and H-3' and HMBC correlations from the methyl H-5' to C-4' (δ_C_ 66.8) and C-3' (δ_C_ 73.4) positioned the side chain at C-6. COSY correlation between H-4' and the hydroxyl group at δ_H_ 4.87 placed the second hydroxy group in position C-4'. HMBC correlations from the ester carbonyl at δ_C_ 169.5 to H-3' and to the methyl at δ_H_ 2.01 positioned an acetate in C-3'. The 11.1 Hz coupling constant of the set of olefinic protons H-1' and H-2' demonstrated the *cis* configuration of the double bond on the side chain. The ROESY correlation between H-5 and H-6 and the *J*_5,6_ value of 2.0 Hz positioned the propenamide side chain at C-6 in a pseudo-equatorial orientation and the hydroxy group at C-5 in axial orientation. Moreover, the positive Cotton effect in the circular dichroism curve (Δε 260–290 nm) was correlated with the C-6(*S*) configuration as described for substituted dihydro-α-pyrones in the literature (Pereda-Miranda et al., 1993). The small coupling constant between H-3' and H-4' (*J*_3’,4’_ = 3.1 Hz) and the ROESY correlations from H-3' to H-4' and OH4’ and from H-4' to H-2' indicated a 3'*S*,4'*R* or 3'*R*,4'*S* configuration of the side chain (Supplementary Figures 25–29).

**FIGURE 5:**
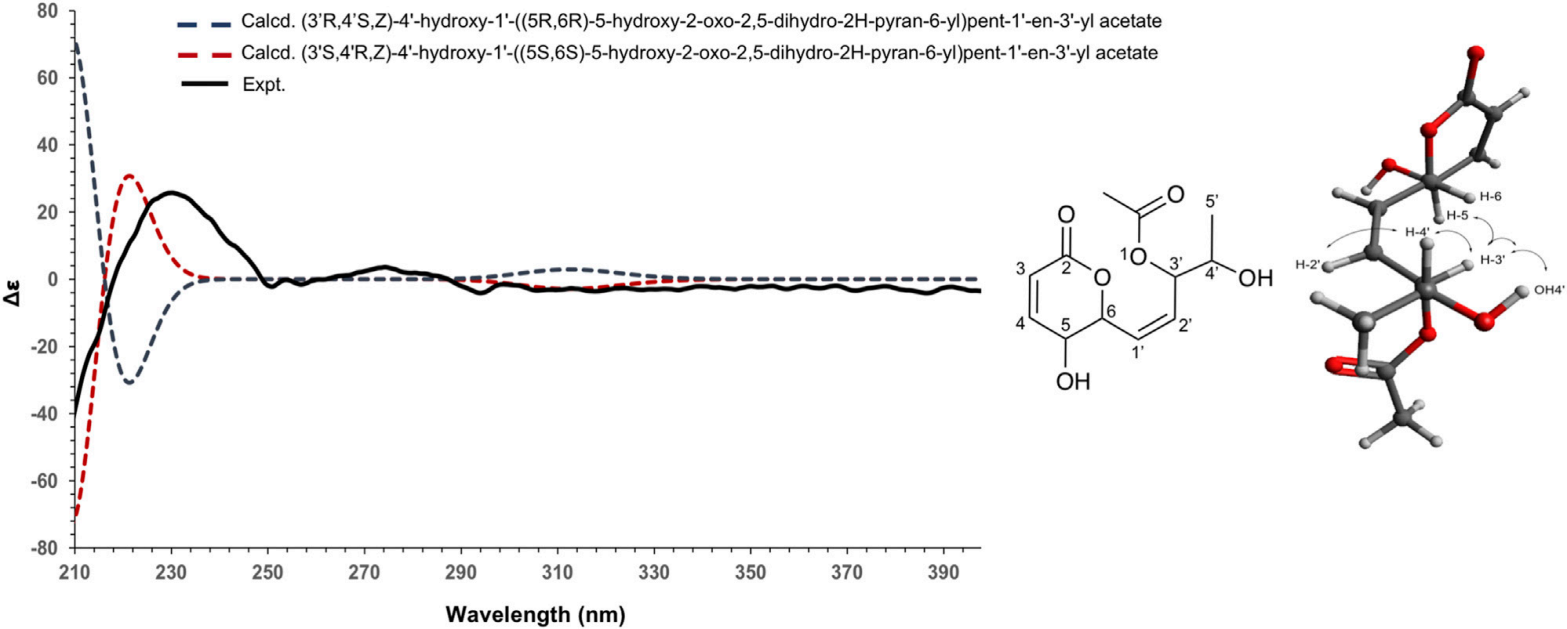
Experimental (black) and TFT-TD-calculated ECD spectra of the two enantiomers (3’*R*,4’*S*,Z), (5R,6R) (blue), and (3’*S*,4’*R*,Z), (5S, 6S) (red) of compound 3.

To establish the absolute configuration of 3, the ECD spectrum was calculated based on the relative configuration proposed by NMR and compared to the experimental data (Figure 5). After the comparison, 3 was assigned as the new molecule (1*Z*,3*S*,4*R*)‐4‐hydroxy‐1‐[(2*S*,3*S*)‐3‐hydroxy‐6‐oxo‐3,6‐dihydro‐2H‐pyran‐2‐yl]pent‐1‐en‐3‐yl acetate.

Molecules containing such an α,β-unsaturated lactone ring are commonly occurring natural products from various biological sources. Compounds with such a structural pattern are known to exhibit a wide range of biological activities. Representative examples are the cytotoxic and antibacterial pectinolides isolated from the plant species *Hyptis pectinata* (Vogt et al., 2009). Other examples include pironetin isolated from *Streptomyces* sp. NK10958, antimitotic and inhibitor of plant growth (Zhou et al., 2017), and asperlin isolated from the marine fungus *Aspergillus versicolor* (Matsumoto and Nago, 1994). To date, apart from isocoumarin derivatives, two 2H-pyran-2-one derivatives, (R)-2-octeno-δ-lactone (lasiolactone) and (R)-2-deceno-δ-lactone (massoialactone), have been isolated from *L. theobromae* (Martínez-Luis et al., 2008).

Compound 4 (F9) was isolated as an oil. The ESI(+)-HRMS experiment indicated a molecular formula of C_12_H_19_NO_6_ with a *m/z* of 274.1291 [M + H]^+^ (calcd. for C_12_H_20_NO_6_^+^, 274.1285, Δppm = 2.15), which corresponded to 4 degrees of unsaturation. Analysis of the ^1^H and ^13^C-DEPTQ NMR data of 4 showed the presence of two methyl groups at δC 15.6 (C-10) and 21.2 (C-12); 4 oximethines carbons at δ_C_ 63.4 (C-4), 70.7 (C-8), 74.4 (C-9), and 78.3 (C-5); 4 olefinic carbons at δ_C_ 122.8 (C-2), 127–7 (C-6), 135.4 (C-7), and 147.1 (C-3); two ester carbonyls at δ_C_ 165.9 (C-1) and 172.6 (C-11). A n-nonane chain was easily followed through the COSY correlations from the ethylenic proton H-2 to the methyl H-10. The HMBC correlation of the carbonyl C-1 with the olefinic set of protons H-2 and H-3 positioned the amide group at one side of the chain and an acetate group was placed on the other side thanks to the HMBC correlation of the carbonyl C-11 to the oximethine H-9 and to the methyl H-12. The 9.8 and 11.3 Hz coupling constants for the sets of olefinic protons H-2/H-3 and H-6/H-7, respectively, demonstrated the *cis* configuration of both double bonds (Supplementary Figures 30–35). Moreover, 4 was identified as the new (4*Z*,8*Z*)-10-amino-3,6,7-trihydroxy-10-oxodeca-4,8-dien-2-yl-acetate.

Compound 6 (F11) was isolated as an oil. The ESI(+)-HRMS experiment indicated a molecular formula of C_10_H_14_O_4_ with a *m/z* of 199.0964 [M + H]^+^ (calcd. for C_10_H_15_O_4_^+^, 199.0965, Δppm = 0.51), which corresponded to 4 degrees of unsaturation. Analysis of the ^1^H and edited HSQC NMR data indicated the presence of two compounds, the minor one being (3*R*,4*R*) or (3*S*,4*S*)-4,5-dihydroxymellein. The major compound (1 mol for 0.5 mol of 4,5-dihydroxymellein) was linked to the following NMR signals: one methyl group at δ_C_ 18.0 (C-10), six olefinic carbons at δ_C_ 120.8 (C-2), 139.6 (C-3), 127.4 (C-4), 139.0 (C-5), 131.1 (C-8), and 129.5 (C-9), and two oximethines carbons at δ_C_ 71.5 (C-6) and 76.9 (C-7). Sequential COSY correlation from the methyl H-10 at δ_H_ 1.67 (dd, *J* = 6.5, 1.5 Hz) to H-9 (δ_H_ 5.72), H-8 (δ_H_ 5.45), H-7 (δ_H_ 3.92), H-6 (δ_H_ 4.45), and H-5 (δ_H_ 5.72) defined one side of the molecule. HMBC correlations from H-7 to C-5, C-6, and C-9 and from the methyl H-10 to C-9 and C-8 confirmed this assignment. The HMBC correlation of the carbonyl at δ_C_ 170.0 (C-1) with the olefinic proton H-3 and the COSY correlation of H-3 with an olefinic proton H-2 at δ_H_ 5.74 positioned the carboxylic acid group in C-1. HMBC correlation of H-3 with C-5 and COSY correlation of H-3 with the olefinic proton H-4 at δ_H_ 7.32 and of H-4 and H-5 helped position the olefinic groups and link both chains. The 11.5 Hz coupling constant for the set of olefinic protons H-2/H-3 and H-4/H-5 demonstrated the *cis* configuration of those double bonds. The 15.2 Hz coupling constant for the third set of olefinic protons H-8/H-9 demonstrated the *trans* configuration of the double bond (Supplementary Figures 42–47). Compound 6 was identified as the new (2*Z*,4*Z*,8*E*)-6,7-dihydroxydeca-2,4,8-trienoic acid.

The NMR data of 15 and 16 were similar to the previously reported data for naphthoquinone spiroketals named CJ-12,372 (Sakemi et al., 1995) and palmarumycin EG1 (Macías-Rubalcava et al., 2014). However, their optical rotations were positive (+2 and +92, respectively), while they were negative for CJ-12,372 and palmarumycin EG1 reported in an unidentified fungus and the endophyte *Edenia gomezpompae*, respectively. Compounds 15 and 16 were thus identified as the new naphthoquinone spiroketals enantiomers (+)-(*R*)-CJ-12,372 and (+)-(*R*)-palmarumycin EG1 (Supplementary Figures 93–102).

Compound 17 (F32) was isolated as an oil. The ESI(-)-HRMS experiment indicated a molecular formula of C_21_H_18_O_4_ with a *m/z* of 335.1253 [M + H]^+^ (calcd. for C_21_H_19_O_4_^+^, 335.1278, Δppm = 7.00) and a *m/z* of 333.1253 [M−H]^−^ (calcd. for C_21_H_17_O_4_^+^, 333.1132, Δppm = 0.51), which corresponded to 13 degrees of unsaturation. ^1^H and ^13^C NMR spectra suggested that the molecule was a deoxypreussomerin derivative related to palmarumycin CP19 (Martínez-Luis et al., 2008). The signals for 1,8-dihydronaphthalene ring system (δ_H_ 6.92, dd, *J* = 7.4, 0.9 Hz, H-2'; 7.45, dd, *J* = 8.4, 7.4 Hz, H-3'; 7.51, dd, *J* = 8.4, 0.9 Hz, H-4'; 7.50, dd, *J* = 8.4, 0.8 Hz, H-5'; 7.43, dd, *J* = 8.4, 7.4 Hz, H-6'; 6.85, dd, *J* = 7.4, 0.8 Hz, H-7') and the substitution pattern in the non-aromatic ring (δ_H_ 4.73, t, *J* = 3.2 Hz, H-1, 2.12, m, H-2a; 2.02, m, H-2b; 2.20, m, H-3a; 2.14, m, H-3b) were identical in both molecules. However, the signals from the third aromatic ring were different, and the multiplicity (δ_H_ 7.27, dd, J = 6.8 Hz, H-5; δ_H_ 6.93, dd, J = 6.8, 2.3 Hz, H-6; δ_H_ 7.26, dd, J = 2.3 Hz, H-8) indicated a 1,2,4-trisubstituted aromatic ring instead of a 1,2,3-trisubstituted aromatic ring in palmarumycin CP19. 17 has a single stereogenic center. Its absolute C-1 configuration was deduced by comparing the sign of the optical rotation with the one of the closely related palmarumycin CP19 (Martínez-Luis et al., 2008). Since the optical rotation of (*S*)-palmarumycin CP19 is negative and 17 has a positive optical rotation, the C-1 configuration was thus determined as *R* (Supplementary Figures 103–106). Compound 17 was identified as the new molecule (*R*)-4-methoxy-3,4-dihydro-2*H*-spiro[naphthalene-1,2'-naphtho[1,8-*de*][1,3]dioxin]-6-ol.

Naphthoquinone spiroketals as palmarumycins belong to a class of natural products isolated from various fungal cultures, exhibiting a wide range of biological properties (Cai et al., 2010). Compounds such as palmarumycin LP1 and cladospirone have been isolated from *Lasiodiplodia pseudotheobromae* (Lü et al., 2014).

### Deep Metabolome Analysis of the *Lasiodiplodia venezuelensis* Strain and Search for Analogs of the Bioactive 5-Hydroxymellein 18

In order to obtain a comprehensive survey of the metabolome of *L. venezuelensis*, the 33 fractions obtained after semi-preparative separation were profiled using data-dependent UHPLC-HRMS/MS. These data were combined with the metabolite profiling of the A02EtM enriched extract used for dereplication. The extract and the fractions MS/MS data were gathered and organized into a single molecular network (MN) (Figure 3). The resulting MN for positive ion data allowed the grouping of 8,500 spectra in 840 clusters. Interestingly, the constitution of this global MN allowed highlighting approximately three times more features than for the extract metabolite profile alone. All the steps and tools involved in the creation of the MN are detailed in the experimental section.

Annotation was performed against an in-house database containing the *in silico* fragmentation spectra of all the compounds present in the DNP (DNP-ISDB) (Allard et al., 2016). Spectral matching against this DB provided a list of the top 50 chemical structures for each node (initial rank). A taxonomic reweighting step allowed the candidate structures to be re-ranked with a weight inversely proportional to the distance between the biological source of the candidate and that of the analyzed sample (species: *Lasiodiplodia venezuelensis,* genus: *Lasiodiplodia*, family: Botryosphaeriaceae) (Rutz et al., 2019). The top six candidates were finally retained (final rank). The annotation was also checked at the MS1 level by verifying the molecular formula of all features after heuristic filtering (Kind and Fiehn, 2007). For all clusters, a consensus structural annotation was returned at the different Classyfire taxa levels (superclass, class, subclass, and parent) (Djoumbou Feunang et al., 2016). The annotation table reports only the top-ranked structures that gave a taxonomically relevant output and/or for which there was a structural consistency in the annotation of the corresponding clusters (Supplementary Tables 1–2). The full MS data set was uploaded and is accessible on the GNPS servers as Massive Data set n° MSV000086605.

Pie charts on the nodes and their proportions refer to the relative intensities of the ions in the extract (dark coral pink) or in the fractions (light coral pink), based on the respective areas of the corresponding extracted ion chromatography (XIC). The numbering of the clusters corresponds to the component index number.

In order to have a confident annotation of the largest possible number of metabolites, all nodes corresponding to the protonated molecule of the 18 isolated compounds were located in the MN (Figure 3) and occurred in nine clusters.

**FIGURE 6:**
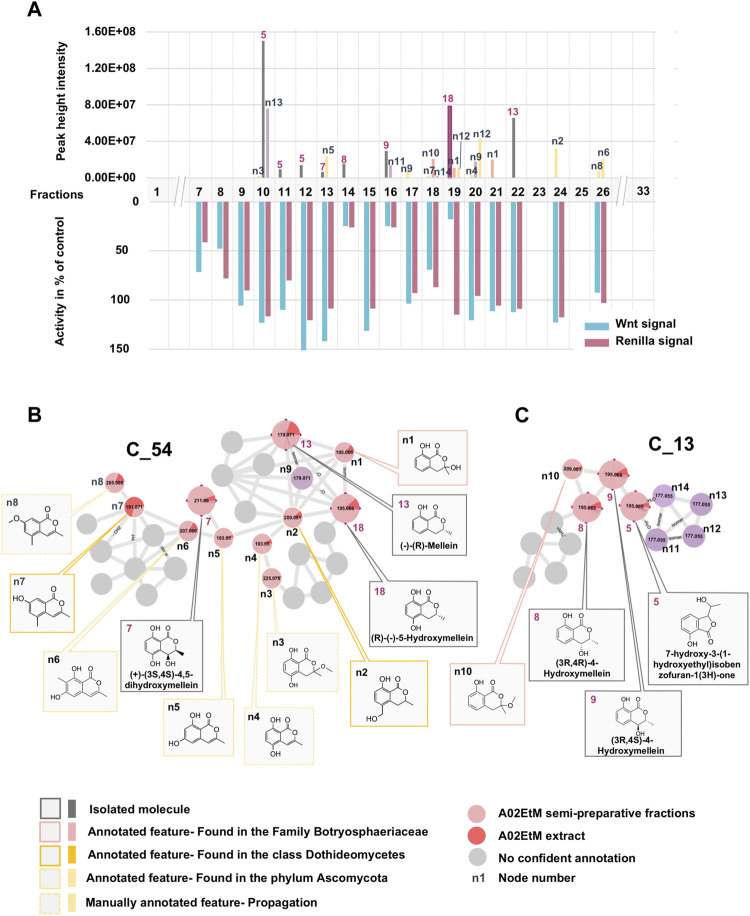
Detailed view of the fraction composition and bioactivity across all fractions containing isocoumarins analogs. **(A)** Histogram represented as a function of the 33 collected fractions showing the peak height intensity of the related putative analogs and their occurrence in the fractions. Associated is the activity of each fraction: in blue, activity on Wnt signal (inhibition activity), in pink, effect on Renilla signal (cytotoxicity). Fraction F19 is considered as specifically active since it inhibits Wnt signaling without having an impact on Renilla signaling. **(B)** Cluster C_54 containing node 18 corresponding to compound **18**, isolated (7, 13) and hypothetical (n1–8) analogs. **(C)** Cluster C_13 containing isolated (5, 8, 9) and hypothetical (n10–14) analogs.

In a first instance, the MN was analyzed to assess the presence of potential analogs of the active compound 18. For this, two clusters containing the nodes of all isolated mellein derivatives were analyzed in-depth to evidence additional putative isocoumarin analogs: cluster C_54 (**7** (F13), 13 (F22), 18 (F19)) and C_13 (8 (F14), 9 (F16)), (Figure 3 and Figures 6B,C). All these nodes were thus unambiguously identified.

For cluster C_54, the occurrence of three isocoumarins demonstrated a good consistency of the annotation and allowed assigning its consensus chemical family to benzopyrans. The MS/MS and molecular formula of all the related features and their taxonomical occurrence (class Dothideomycetes, family Bothryosphaeriaceae, order Bothryosphaeriales, genus *Lasiosiplodia*) were carefully analyzed. This enabled annotating with good confidence eight additional nodes (n2 and n8) as isocoumarin derivatives (Figure 6B). This seems consistent since mellein, dihydroxymellein, methylisocoumarins, and isocoumarins have a common biosynthetic pathway related to fatty acid biosynthesis catalyzed by polyketides synthases (PKS) (Reveglia et al., 2020).

The other two isolated mellein derivatives 8 and 9, bearing a hydroxyl in position 4, were located in C_13 (Figure 6C). In this smaller cluster, several additional nodes were found to be related to 5, another structurally different benzopyran. The node n10 could be putatively annotated as another mellein derivative found in the Bothryosphaeriaceae family (Xu et al., 2012) (Figure 6C).

Overall, the dereplication of this cluster using the isolated molecules as anchor points showed that the extract potentially contained analogs of the active compound 18. All these annotated compounds were located in the fractions in which they mainly occur. Based on the semi-preparative fractionation and continuous bioactivity monitoring (Figure 1), a hypothetical assessment of their possible anti-Wnt or cytotoxic effects was performed. Figure 6A shows the location of each feature in the fractions, alongside the activity of the fraction. This is represented as a histogram ordered according to the fractionation process illustrated in Figure 1. Among all 19 isolated and annotated structural analogs of 18, this approach shows that the large majority have neither Wnt inhibition (no reduction of Wnt signal) nor cytotoxicity (no reduction of Renilla signal) (Figure 6).

Interestingly, two fractions F14 and F16 showed important Wnt inhibition but also similar cytotoxic effects. They were found to contain 8 (F14), 9 (F16), and n11 (F16). All these compounds are 4-hydroxy derivatives without hydroxyl in position 5, while 18 did not have any hydroxylation in 4. However, the IC_50_ determination of the pure 8 and 9 demonstrated that they did not show any cytotoxicity up to high concentrations (see Figures 2B,C). The annotated compound n11 could not be isolated but might contribute to the cytotoxicity of fraction F16. Fraction 18 showed a moderate Wnt effect and equivalent cytotoxicity. This could be related to the presence of the annotated compound n10, which has possibly no hydroxylation in position 4, as this was the case for 18. Overall, the analyses of all fractions and their content and the activity measurement showed an important selectivity of 18 for Wnt inhibition, whereas the majority of derivatives had no effect.

The IC_50_ measurement of isolated compounds (7, 8, 9, 13) confirmed that these compounds did not show any specific inhibition of Wnt3a-induced signal up until estimated concentrations of ∼500 μM, indicating that 18 acts in a highly specific manner (Figure 2B). Moreover, we have confirmed that 18 is very specific to Wnt inhibition and shows some acute unspecific toxicity only at very high concentrations (cytotoxic IC_50_ estimate is at ∼800 μM, (Figure 2C). Finally, by assessing the activity of 18 when the Wnt pathway was stimulated downstream through GSK3beta inhibition, we demonstrated that it is highly selective for upstream components of the pathway since inhibition of CHIR99021 signal occurs only at cytotoxic concentrations and is thus unspecific (Figure 2D).

**FIGURE 7:**
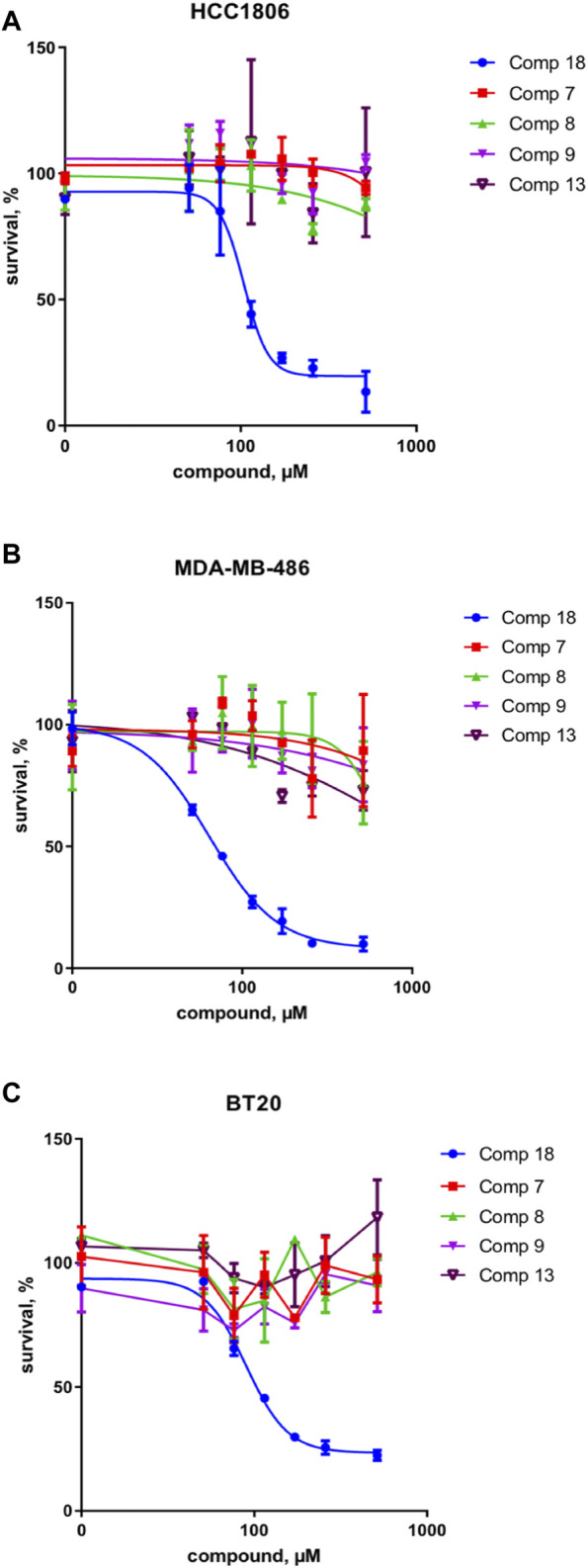
Survival of three different TNBC cancer cell lines in % of control for compounds 18 and 7, 8, 9, and 13 in **(A)** HCC 1806, **(B)** MDA-MB-486, and **(C)** BT20 cell lines. Compound 18 inhibits the proliferation of three TNBC cell lines and thus shows a broad effect on the TNBC cell types.

These results were complemented by analyzing if this activity against the upstream component of the Wnt pathway can be translated into the inhibition of the cancer cell growth. Indeed, only the mellein 18 was able to inhibit the proliferation of three TNBC cell lines (Figures 7A–C). Interestingly, the IC_50_ of this effect was rather different across the cell lines, indicating that their sensitivity is likely regulated by the expression levels of the potential target. The small set investigated indicated the specificity of 18 among the series of analogs within the strain. A larger set of other mellein derivatives would be interesting to investigate. The use of MS-targeted isolation performed at a larger scale could be considered in the future for the selective isolation of some of the minor analogs detected.

### Annotation of the Metabolome of the *Lasiodiplodia venezuelensis* Strain Based on all Isolated Compounds

We took advantage of having isolated in one step several main metabolites of the *L. venezuelensis* extract including the new compounds to evaluate the presence of their analogs, following the same strategy as for the active compound 18. This provided an overview of its metabolome composition.

Compounds 1–4, 6, 10–12, 14, and 15–17 were found to be spread across seven additional clusters (Figure 3) to those containing isocoumarin derivatives (C_54 and C_13).

The new pyran derivative 1 was attributed to node C_28_1. Two additional analogs, putatively annotated as diplopyrone in the Botryosphaeriaceae family, and another pyranone were evidenced (nodes C_28_n1 and n2, Supplementary Figures 107). Similarly, the new pyranone derivative 3 was attributed to C_ 23_3 and compound 4 was also present in the cluster (C_23_4). We have assumed that those two molecules are in the same cluster because 3 could correspond to a cyclized form of 4 (Supplementary Figures 108). C_23_n1 and n2 bearing a pyranone core structure similar to 3 were also annotated in this cluster. As said previously, apart from isocoumarins, such 2H-pyran-2-one derivatives have been isolated from *L. theobromae* (lasiolactone and massoialactone) (Matsumoto and Nago, 1994). Another pyran-2-one derivative has been isolated from a strain of *Lasiodiplodia* PSU-M114 as tetrahydro-4-hydroxy-6-propylpyran-2-one (Rukachaisirikul et al., 2009).

Compounds 2, 6, and 14 were spotted in three different clusters of fatty acyl derivatives (C_1,497, C_43, and C_164, Supplementary Figures 109). They are new to this family of fungi, but diverse free fatty acids and their esters have been identified in the cultures of botryosphaeriaceous fungi (Uranga et al., 2016). Free fatty acids are important as they are the starting material of many secondary metabolites such as oxylipins, and a source of acetyl CoA required for polyketides produced by Botryosphaeriacae. They are also involved in plant growth regulation, virulence, and plant-fungi communication (Salvatore et al., 2020).

The butyrolactones 10 and 11 occurred in cluster C_97 (nodes C_97_10 and C_97_11, Supplementary Figures 110). They have previously been isolated for *L*. *theobromae* and are often found in this genus. In the same cluster, n1 and n2 were manually annotated based on their fragmentation pattern. Nodes n3 and n4 correspond to butyrolactone derivatives with higher molecular weight. n3 is a metabolite reported in the Botryospheariales. Another analog, decumbic acid B (12), was isolated, but its associated feature could not be evidenced in the MN in positive mode since it ionizes in negative mode only (Supplementary Figures 111). It is the first report of its occurrence in the genus.

The naphthoquinone spiroketals 15 and 16 were associated with nodes C_72_15 and C_72_16 (Supplementary Figures 112). Two nodes n1 and n2 corresponding to naphthoquinone spiroketals were annotated as palmarumycins isolated from fungi from the Dothideomycetes class (Xu et al., 2015). The nodes n4 and n6 were attributed to potential isomers of 16 and n3. The other derivative 17 did not belong to this cluster and occurred as a single node which can be explained by a different fragmentation pattern related to the position of the hydroxy group. The presence of three new naphthoquinone spiroketals (15,16, and 17) and several derivatives detected by MN shows the biosynthetic potential this strain. This kind of compounds was never reported in the genus *Lasiodiplodia*.

Following this strategy, nine clusters were assigned to five chemical classes and significantly increase metabolome information on this strain. All annotations performed were carefully checked with respect to taxonomic consistency and/or annotation propagation from the compounds unambiguously identified by NMR following our one-step isolation strategy. This provided good confidence for the identification of the various analogs highlighted.

Several metabolites found in the remaining clusters were annotated solely on the basis of the comparison of their MS/MS pattern against the *in silico* fragmentation database and the taxonomical re-ranking. This made it possible to highlight the presence of ubiquitous fungal chemical families such as various steroids, lipids, and amino acid derivatives. Metabolites more specifically occurring in the Botryosphaeriaceae were also highlighted and include jasmonates (cyclic ketone) derivatives (Tsukada et al., 2010; Andolfi et al., 2014), benzopyrans (Rukachaisirikul et al., 2009; Salvatore et al., 2020), and lactones derivatives such as as lasiodiplodins (Shen et al., 2015), dioxanes (Rukachaisirikul et al., 2009), and depsidones (Umeokoli et al., 2019). The annotation of the corresponding features is available in Supplementary Table 2 and Supplementary Figures 113–120.

The annotation of the metabolome of this strain shows that the most represented chemical families correspond to those reported for the genus *Lasiodiplodia*, which shows good consistency and has revealed the presence of many analogs not yet described in this genus.

The inclusion of metabolite enriched fractions to the MN metabolite profiling enables the detection of many additional minor constituents of the metabolome, further revealing the biosynthetic potential of this strain. This deep metabolome characterization is worth further investigating but would require working at a much larger scale with the current technologies for both biological assessment and precise structural assignment of the wealth of metabolites present.

## Conclusion

Inhibition of Wnt signaling is a highly desired mode of action for targeted agents against TNBC and a number of other cancers due to its efficacy, especially against the tumor recurrence (Zhan et al., 2017; Steinhart and Angers, 2018). However, all attempts to inhibit this pathway did not result in clinically approved agents due to the safety concerns on clinical and/or preclinical stages. Largely, this is the result of direct, unselective inhibition of the pathway, leading to disruption of its functioning in healthy tissues (Shaw et al., 2019a). Nevertheless, the pathway structure allows finding cancer-selective compounds; as such, they must target the upstream components (Shaw et al., 2019b). We have identified novel Wnt inhibitory properties of the mellein derivative 18 and validated it as an inhibitor of upstream levels of the pathway. Inhibition seems to be highly specific to the arrangement of this exact scaffold since a variety of similar compounds do not show inhibitory activity. As expected for a Wnt inhibitor, the compound is able to efficiently inhibit the proliferation of three TNBC cell lines. Of note, the growth inhibition is observed within the same range as the Wnt signal decrease, thus further emphasizing the specificity of the compound’s action. Overall, our results lay a solid foundation for further development of 18 and, likely, its potency can be enhanced by further modifications, especially if driven by further identification of its molecular target. The approach used in this study allowed the direct isolation and study of analogs of 18 and shows the presence of several additional minor derivatives in the strain. Interestingly, it has been shown that a mellein derivative, *O*-methylmellein, was strongly induced when *Botryosphaeria obtusa* (Botryosphaeriaceae) was in confrontation with another wood-decaying fungus *Eutypa lata* and exhibited toxicity towards other fungi (Glauser et al., 2009). Confrontation experiments with *L. venezuelensis* might be an interesting way to obtain new bioactive analogs of 18. In that respect, it would be interesting to test 18 against other targets as fungi or bacteria.

Overall, the high throughput semi-preparative HPLC-UV fractionation using dry load injection, combined with UHPLC-PDA microfractionation, led to the isolation of 18 compounds from *Lasiodiplodia venezuelensis*. Five are original natural compounds *de novo* identified in this study. Seven are new to the Botryosphaeriaceae family, or analogs of compounds related to this family of fungi. In addition to the discovery of a Wnt inhibitor of interest, the UHPLC-HRMS/MS metabolite profiling and the MN contribute to characterize better the metabolome of *L. venezuelensis* and its large biosynthetic potential. The metabolome information on this strain and other strains of the community may provide an interesting complement to metagenomic approaches applied to Astrocaryum and its ecosystem (Donald et al., 2020).

This work also stresses the importance of carefully assessing purity through appropriate methods for an accurate bioactivity assessment. Indeed, as illustrated here, the activity can be due to minor compounds and raises the question of residual complexity when compounds are isolated from natural product extracts (Choules et al., 2018). Nevertheless, as shown in the present study, the comprehensive workflow developed that combines UHPLC-HRMS/MS dereplication, targeted microfractionation, NMR, and qNMR helped to successfully assign the bioactivity to the right constituent and assess the link between the analogs present and bioactivity in a complex fungal strain extract.

## Experimental Section

### General Experimental Procedures

NMR spectroscopic data were recorded on a Bruker Avance Neo 600 MHz NMR spectrometer equipped with a QCI 5 mm Cryoprobe and a SampleJet automated sample changer (Bruker BioSpin, Rheinstetten, Germany). Chemical shifts are reported in parts per million (*δ*) using the residual CD_3_OD (δ_H_ 3.31; δ_C_ 49.0) or DMSO-*d*_6_ (δ_H_ 2.50; δ_C_ 39.5) as internal standards for ^1^H and ^13^C NMR, respectively, and coupling constants (*J*) are reported in Hz. Complete assignments were obtained based on 2D-NMR experiments: COSY, ROESY, edited HSQC, and HMBC. HRESIMS data were obtained on a Q-Exactive Focus mass spectrometer (Thermo Scientific, Bremen, Germany), using heated electrospray ionization (HESI-II) source. Analytical HPLC was carried out on a HP 1260 system equipped with a photodiode array and an ELSD detector (Sedere, Alfortville, France). Semi-preparative HPLC-UV analyses were conducted on a Shimadzu system (Kyoto, Japan) equipped with LC-20A module pumps, a SPD-20A UV/VIS, a 7725I Rheodyne® valve, and a FRC-10A fraction collector. The system is controlled by the software LabSolutions from Shimadzu.

### Electronic Circular Dichroism and Optical Rotation

The spectra obtained by electronic circular dichroism (ECD) and UV were analyzed on a J-815 circular dichroism spectrometer (Loveland, CO, United States). The solvent used was methanol. The cell temperature was 24.5°C and stabilized using a thermostat ED circulation bath (Julabo, Seelbach, Germany) and a PFD-350S thermostat (Jasco). The set scan speed was 200 nm/min in continuous mode with a scan start at 800 nm and an end at 200 nm. The optical rotations were measured in methanol solutions on Jasco P-1030 polarimeter in a 1 cm tube.

### Plant Material

*Astrocaryum sciophilum* palm tree leaves were sampled in August 2017 at the Nouragues ecological research station in French Guiana. Leaf samples were collected at the Inselberg research station (4°05'N–52°41'W) by randomly cutting 5 × 3 cm pieces of fresh leaflets and rachis from individual plants using sterile tweezers. The pieces were placed in Eppendorf tubes containing Potato Dextrose Broth 1/4 (PDB) medium to allow regrowth of the fungi and tubes containing cetyltrimethylammonium bromide (CTAB) to preserve the genetic material. Petri dishes containing “potato dextrose agar” medium and aureomycin were also left open on the stipe in order to sample fungi from the environment close to the palm tree. These media were enriched with aureomycin to avoid the growth of bacteria and promote the growth of fungi only.

### Isolation of Endophytes

The surface of the plant fragments was thoroughly washed for 3 hours under a stream of tap water in a suitable container and then washed in three successive baths of sterile water for 10 min each under a laminar flow hood. This non-chemical surface cleaning method was used to reduce the possible loss of diversity due to aggressive sterilization using alcoholic solutions or even Paraquat (Gindrat and Pezet, 1994). The surface-sterilized leaves were aseptically cut into small segments (0.5 cm^2^), placed individually in Petri dishes containing potato dextrose agar medium (PDA, Potatoe Dextrose Agar, Sigma-Aldrich, Germany), amended with aureomycin (25 ppm. L^−1^) in 9 cm Petri dishes, and cultured at room temperature. Each individual emerging hypha fragment was removed and placed in 9 cm Petri dishes containing PDA and aureomycin. The isolated and selected strains were then individually cultured on a small scale in 9 cm Petri dishes containing PDA medium without aureomycin. Each strain has been integrated into the dynamic fungal library of Agroscope, whose content is available on the web (database Mycoscope: www.mycoscope.ch), in vials containing 5 ml of diluted PDB (PDB, Potato Dextrose Broth, Sigma-Aldrich, Germany) aqueous solution (1:4) at 4°C.

### Identification of Fungal Strains

Identification of the isolated strains was performed by extraction of the ribosomal DNA (phenol/chloroform), PCR amplification of the ITS1F and ITS4 region, and sequencing (Fasteris, Geneva, Switzerland). The obtained sequences were then submitted to BLAST on NCBI (https://blast.ncbi.nlm.nih.gov/Blast.cgi) to identify the strain. The specimens were then entered in the dynamic mycotheca of Agroscope and registered in the fungal database (www.mycoscope.ch) under the reference numbers 1883 to 2024.

### Culture and Extraction

Each strain was cultivated at 21°C under alternating light and dark (12 h each) in 10 cm Petri dishes containing PDA culture medium. The culture medium was then extracted by maceration in ethyl acetate (EtOAc) for 24 h at room temperature under agitation. The organic phase was recovered by vacuum filtration and washed three times with Millipore Corporation (MilliQ) water. The organic and water phases were dried under reduced pressure with a rotary evaporator to yield crude mixtures. Agar from uninoculated PDA plates was used as controls.

### Scale-Up of the Fungal Culture of *Lasiodiplodia venezuelensis*

Strains were cultivated for 15 days at room temperature in 14 cm Petri dishes containing PDA medium. Culture media were then consecutively extracted three times by maceration in EtOAc for 24 h at room temperature under agitation (after each 24 h maceration, the organic phase was harvested and the medium was macerated again in renewed EtOAc). The combined organic solution was washed three times with MilliQ water. The organic and water phases were dried under reduced pressure with a rotary evaporator to yield the ethyl acetate fraction (Et) and the water fraction (W). The Et fraction was then degreased by liquid/liquid partition in a hexane/methanol:water (1/1) system with methanol:water (7:3) to give the hexane (EtH) and the methanol/water fraction (EtM) (Supplementary Figure 1). Large-scale cultivation of the *Lasiodiplodia venezuelensis* strain was conducted on 100 14 cm Petri dishes to yield the factions A02Et (1 g) and A02W (335 mg). 600 mg of the fraction A02Et was then degreased to yield fractions A02EtH (320 mg) and A02EtM (250 mg).

### UHPLC-PDA-CAD-HRMS/MS Analysis

Metabolite profiling of the extracts was performed by UHPLC-PDA-CAD-HRMS/MS as follows. The separation was achieved on an Acquity BEH C18 column (2.1 × 50 mm; 1.7 μm). The temperatures in the autosampler and in the column oven were, respectively, set at 10 and 40°C. The mobile phase constituted of water (A) and acetonitrile (B), both containing 0.1% formic acid; the separation was performed with a linear gradient from 5 to 100% of B in 7 min followed by a 1 min isocratic step at 100% of B. The flow rate was set at 600 *μ*L/min and the injection volume was set at 2 *μ*L. The optimized HESI-II parameters were as follows: source voltage, 3.5 kV (pos), 4kV (neg); sheath gas flow rate (N_2_), 55 units; auxiliary gas flow rate, 15 units; spare gas flow rate, 3.0; capillary temperature, 275°C (pos), 320°C (neg); S-Lens RF Level, 45. The data-dependent MS/MS events were performed on the four most intense ions detected in full-scan MS (top three experiments). The MS/MS isolation window width was 1 Da, and the normalized collision energy was set to 35 units. In data-dependent MS/MS experiments, full scans were acquired at a resolution of 35,000 FWHM (at 200 m*/z*) and MS/MS scans at 17,500 FWHM, both with a maximum injection time of 50 ms. After being acquired in a MS/MS scan, parent ions were placed in a dynamic exclusion list for 2.0. In positive mode, the di-isoctyl phthalate C_24_H_38_O_4_ [M + H]^+^ ion (*m/z* 391.28429) was used as an internal lock mass. The mass analyzer was calibrated using a mixture of caffeine, methionine-arginine-alanine-acetate (MRFA), sodium dodecyl sulfate, sodium taurocholate, and Ultramark 1,621 in an acetonitrile/methanol/water solution containing 1% formic acid by direct injection. An Acquity UPLC photodiode array detector was used to acquire UV spectra detected in the 200–500 nm range.

### UHPLC-HRMS/MS Data Processing

The raw files containing UHPLC-HRMS/MS data were converted into mzXML files using the msConvert software. The mzXML files were then processed using MZmine (v2.51) (Pluskal et al., 2010). Mass detection was carried out using a centroid mass detector with a noise level set at 5 E^4^. The ADAP chromatogram builder was employed with a minimum height of 5 E^4^, a minimum group size of scans of 5, a minimum group intensity threshold of 5 E^4^, a minimum highest intensity of 5 E^4^, and a *m/z* tolerance of 8 ppm. The wavelets ADAP algorithm was used for chromatogram deconvolution with the following settings: a single-to-noise (S/N) threshold of 10, intensity window SN, a minimum feature height of 5 E^4^, a coefficient area threshold of 100, a peak duration range between 0.02 and 0.9 min, and a wavelet range between 0.00 and 0.05 min. The *m/z* and retention time (RT) ranges for MS^2^ scan pairing were, respectively, set to 0.025 Da and 0.1 min. The chromatograms were deisotoped using the isotope peak grouper algorithm with a *m/z* tolerance of 8 ppm, a RT tolerance of 0.08 min, and a maximum charge of 2, while the representative isotope used was the most intense. The peak alignment was performed using the join aligner method with a *m/z* tolerance of 8 ppm, a RT absolute tolerance of 0.05 min, and a weight for *m/z* and RT at 20. An adduct search (Na^+^, K^+^, NH_4_^+^, ACN^+^, and HCOOH) was performed on the peak list with an RT tolerance of 0.01 min, a *m/z* tolerance of 8 ppm, and a maximum relative peak height of 1,000%. A complex search was performed on the peak list with an RT tolerance of 0.01 min, a *m/z* tolerance of 8 ppm, and a maximum relative peak height of 1,000%. The peak list was then gap-filled using the same RT and *m/z* range gap filler with a *m/z* tolerance of 8 ppm*.* A custom database of the fungi group (17,255 compounds) was applied for in silico identification from the Dictionary of Natural Product (DVD version 26.2) (Dictionary of Natural Pro, 2020).

### Molecular Network Analysis

The MZmine files were exported in MGF format for the processing of the molecular network (MN) in the Global Natural Products Social (GNPS) platform (Wang et al., 2016). In order to maintain the RT and exact mass information and allow isomer separation, feature-based MN was created using the MGF file resulting from the MZmine pre-treatment steps detailed above. The spectral data were then uploaded on the GNPS MN platform. A network was created where edges were filtered to have a cosine score above 0.65 and more than six matched peaks. Further edges between two nodes were kept in the network if and only if each of the nodes appeared in each other’s respective top 10 most similar nodes. The spectra in the network were then searched against GNPS’ spectral libraries. All matches kept between network spectra and library spectra were required to have a score above 0.7 and at least six matched peaks. The analogs spectra were searched against the DNP-ISDB database (Allard et al., 2016). The top 50 2D chemical structures were provided for each node according to the *in silico* MS/MS fragmentation spectra match, called “initial rank.” A chemotaxonomical filter was applied to weight the search according to the occurrence of structures in Botryosphaeriaceae to give a ranking of the top six most probable structures and was called “final rank.” The output was visualized using Cytoscape v3.7.1 (https://cytoscape.org/). Peak areas of different analyses were represented as pie chart diagrams. Node sizes were proportional to the peak areas of the extract. The full MS dataset is uploaded and accessible on the GNPS servers as Massive Data set n° MSV000086605.

### HPLC-PDA-ELSD Analyses

HPLC-PDA analyses were conducted as follows: Waters® X-bridge C18 column (250 × 4.6 mm i. d., 5 μm) (Waters®, Milford, MA, United States) equipped with a Waters® C18 pre-column cartridge holder (20 × 4.6 mm i.d.); solvent system H_2_O (A) and MeOH (B), both containing 0.1% formic acid (FA). The column was equilibrated with 5% of B for 15 min. The separation was performed with a gradient mode from 5 to 60% of B over 70 min, then from 60 to 100% of B over 20 min. The column was then washed with 100% of B for 10 min and equilibrated with 5% of B over 15 min. The flow rate was 1 ml/min, the injection volume 20 μL, and the sample concentration 1 mg/ml in MeOH. The UV absorbance was measured at 254 and 280 nm and the ELSD set at 45°C, 3.5 bar N_2_, gain 8.

### UHPLC-UV-ELSD-QDa Analyses

UHPLC-UV-ELSD-QDa analyses were performed on an Acquity BEH C18 column (2.1 × 50 mm; 1.7 *μ*m) using a mobile phase consisting of water (A) and acetonitrile (B), both with 0.1% FA. The separation was performed with a linear gradient from 5 to 100% of B in 7 min followed by a 1 min isocratic step at 100% of B. The flow rate was set at 600 *μ*L/min and the injection volume at 2 *μ*L. The temperatures in the autosampler and in the column oven were, respectively, set at 10 and 40°C. UV data were acquired from 200 to 600 nm and the ELSD temperature was set at 45°, the pressure at 3.5 bars,, and the gain at 8. Mass data were obtained by a fully automated acquisition at 10 Hz for *m/z* between 100 and 1,000. Data acquisition, instrument control, and data processing were performed with the Masslynx® software (Waters®).

### Semi-Preparative HPLC-UV Purification of the Hydro Alcoholic Fraction of the Strain *Lasiodiplodia venezuelensis* (A02EtM)

Semi-preparative HPLC-UV analyses were conducted as follows: Waters® X-bridge C18 column (250 × 19mm i.d., 5 μm) equipped with a Waters® C18 pre-column cartridge holder (10 × 4.6 mm i.d); solvent system H_2_O (A) and MeOH (B), both containing 0.1% FA. The column was equilibrated with 5% of B for 15 min. The separation was performed with a gradient mode from 5 to 60% of B over 71.63 min, then from 60 to 100% of B over 21.46 min. The column was then washed with 100% of B for 10 min and equilibrated with 5% of B over 15 min. The flow rate was 17 ml/min. These conditions were calculated from a geometric gradient transfer from the analytical HPLC using the software developed by Guillarme et al. (Guillarme et al., 2008). The UV traces were recorded at 254 and 280 nm. The HPLC-UV chromatograms of the different separation are shown (Supplementary Figure 2). The A02EtM extract (10, 30, 35, 32, and 32 mg) was injected in the semi-preparative HPLC-UV using dry load. The dry load injection was performed using a dry load cell, i.e., a commercial small aluminum pre-column cartridge from Waters® (10 × 19 mm i.d.) that was opened on one side. The extract was mixed with the same ratio of stationary phase (C18 Zeoprep® 40–63 μm), and this mixture was transferred in the dry load cell as a thin layer at the bottom of the cell. That thin layer was then covered with stationary phase until reaching a final mass of 1000 mg and the cell was closed with a metallic sinter. The dry load was then directly connected to the Rheodyne® valve (Queiroz et al., 2019). According to UV signals, 33 fractions were collected. All the fractions were then analyzed by UHPLC-UV-ELSD-QDa and tested in the Wnt inhibition assay to localize bioactive fractions.

### Micro UHPLC-PDA Purification of the Active Fraction F19

Separation was performed on a Waters® Acquity UPLC system (Waters®, Milford, MA, United States) equipped with a UV-Vis programmable detector and an autosampler with a 2 *μ*L loop. Data acquisition, data handling, and instrument control were performed with Empower Software (Waters®). The separation analyses of fraction F19 were performed on an Acquity BEH phenyl column (2.1 × 30 mm; 1.7 *μ*m) using a mobile phase consisting of water (A) and acetonitrile (B), both with 0.1% FA. The separation was conducted with an isocratic mode: 5% of B during 15 min. The temperatures in the autosampler and column oven were, respectively, set at 10 and 40°C. UV data were acquired at 210 nm with a time constant set at 100 ms and a data sampling rate at 20 points/s. The flow rate was set at 300 *μ*L/min. The fraction was concentrated at 4 mg/ml in ACN. 75 injections of 2 *μ*L were performed. Three peaks were collected based on the time frame by which their UV signals were received. The collection for the first peak was set from 5.67 to 6.39 min, the second one from 7.09 to 7.91 min, and third one from 9.05 to 10.12 min. This allowed yielding 94 *μ*g of peak 1 (compound 10), 83 *μ*g of peak 2 (compound 11), and 30 *μ*g of peak 3 (compound 22). The reproducibility of the 75 injections can be accessed in Supplementary Figure 5.

### Description of the Isolated Compounds

(Z)-3-((2R,3R,6R)-3-Hydroxy-6-((R)-1-hydroxyethyl)-3,6-dihydro-2H-pyran-2-yl)acrylamide (1) (F6). ${\left[a\right]}_{D}^{25}$ 14.14 (c. 0.1, MeOH); UV(MeOH) λ_max_ 205 (3.94), 321 (2.32) nm. ^1^H NMR (MeOD, 600 MHz) *δ* 1.19 (3H, d, *J* = 6.4 Hz, H-8), 3.71 (1H, *p*, *J* = 6.4, 5.8 Hz, H-7), 4.04 (1H, dtd, *J* = 5.8, 2.3, 2.0, 1.2 Hz, H-2), 4.17 (1H, dd, *J* = 5.8, 2.8 Hz, H-6), 4.74 (1H, dtd, *J* = 5.8, 2.8, 2.0, 1.2 Hz H-5), 6.10 (1H, ddd, *J* = 10.4, 5.2, 2.3 Hz, H-4), 6.18 (1H, d, *J* = 9.8 Hz, H-2'), 6.32 (1H, dt, *J* = 10.4, 1.2 Hz, H-3), 7.00 (1H, dd, *J* = 9.8, 5.8 Hz, H-1'); ^13^C NMR (MeOD, 151 MHz) *δ* 18.8 (C-8), 66.2 (C-6), 69.9 (C-7), 71.5 (C-5), 80.6 (C-2), 123.3 (C-4), 124.8 (C-2'), 135.0 (C-3), 142.7 (C-1'), 165.4 (C-3'). ESI(+)-HRMS *m/z* 214.1071 [M + H]^+^ (calcd. for C_10_H_16_NO_4_^+^, 214.1074, Δppm = 1.40). For NMR spectra, see Supplementary Figures 6–11.

Aconitate B (6-methyl ester trans-aconitate) (2) (F7). UV(MeOH) λ_max_ 209 (3.49) nm. ^1^H NMR (MeOD, 600 MHz) *δ* 3.81 (3H, s, H-7), 3.88 (2H, s, H-4), 6.91 (1H, s, H-2); 13C NMR (MeOD, 151 MHz) *δ* 34.0 (C-4), 53.1 (C-7), 131.7 (C-2), 139.9 (C-3), 168.2 (C-1, C-6), 173.8 (C-5). ESI(+)-HRMS *m/z* 189.0394 [M + H]^+^ (calcd. for C_7_H_9_O_6_^+^, 189.0394, Δppm = 0.00). For NMR spectra, see Supplementary Figures 19–24 (Cai et al., 2001).

(3R,4R,Z)-4-Hydroxy-1-((2S,3S)-3-hydroxy-6-oxo-3,6-dihydro-2H-pyran-2-yl)pent-1-en-3-yl acetate (3) (F8). ${\left[a\right]}_{D}^{25}$ 9.98 (c. 0.1, MeOH); UV(MeOH) λ_max_ 205 (3.99), sh 264 (3.37), 336 (3.25) nm. ^1^H NMR (DMSO-*d6*, 600 MHz) *δ* 1.05 (3H, d, *J* = 6.4 Hz, H-5'), 2.01 (3H, s, H-7'), 3.77 (1H, qdd, *J* = 6.4, 5.3, 3.1 Hz, H-4'), 4.02 (1H, ddd, *J* = 7.1, 5.6, 2.0 Hz, H-5), 4.87 (1H, d, *J* = 5.3 Hz, OH4’), 5.25 (1H, dt, *J* = 9.2, 2.0, 1.1 Hz, H-6), 5.29 (1H, dd, *J* = 9.3, 3.1 Hz, H-3'), 5.60 (1H, d, *J* = 7.1 Hz, OH5), 5.71 (1H, ddd, *J* = 11.1, 9.3, 1.1 Hz, H-2'), 5.90 (1H, t, *J* = 11.1, 9.2 Hz, H-1'), 6.03 (1H, d, *J* = 9.8 Hz, H-3), 7.07 (1H, dd, *J* = 9.8, 5.6 Hz, H-4); ^13^C NMR (DMSO-*d6*, 151 MHz) *δ* 18.4 (C-5'), 20.7 (C-7'), 60.8 (C-5), 66.8 (C-4'), 73.4 (C-3'), 76.1 (C-6), 121.1 (C-3), 128.2 (C-1'), 129.7 (C-2'), 146.1 (C-4), 162.9 (C-2), 169.4 (C-6'). ESI(+)-HRMS *m/z* 257.1020 [M + H]^+^ (calcd. for C_12_H_17_O_6_^+^, 257.1020, Δppm = 0.00). For NMR spectra, see Supplementary Figures 25–29.

(2Z,6Z)-4,5,8,9-Tetrahydroxydeca-2,6-dienamide (4) (F9). ${\left[a\right]}_{D}^{25}$ 32.40 (c. 0.08, MeOH); UV(MeOH) λ_max_ 206 (2.65), sh 266 (1.93), 343 (1.85) nm. ^1^H NMR (MeOD, 600 MHz) *δ* 1.24 (3H, d, *J* = 6.5 Hz, H-10), 2.05 (3H, s, H-12), 4.18 (1H, dd, *J* = 5.6, 2.9 Hz, H-4), 4.46 (1H, ddd, *J* = 8.3, 5.0, 1.2 Hz, H-8), 4.90 (1H, dd, *J* = 6.5, 5.0 Hz, H-9), 5.33 (1H, ddd, *J* = 9.1, 2.9, 1.0 Hz, H-5), 5.78 (1H, ddd, *J* = 11.3, 8.3, 1.0 Hz, H-7), 5.93 (1H, ddd, *J* = 11.3, 9.1, 1.2 Hz, H-6), 6.09 (1H, d, *J* = 9.8 Hz, H-2), 7.07 (1H, dd, *J* = 9.8, 5.6 Hz, H-3); ^13^C NMR (MeOD, 151 MHz) *δ* 15.6 (C-10), 21.2 (C-12), 63.4 (C-4), 70.7 (C-8), 74.4 (C-9), 78.3 (C-5), 122.8 (C-2), 127.7 (C-6), 135.4 (C-7), 147.1 (C-3), 165.9 (C-1), 172.6 (C-11). ESI(+)-HRMS *m/z* 274.1291 [M + H]^+^ (calcd. for C_12_H_20_NO_6_^+^, 274.1285, Δppm = 2.15). For NMR spectra, see Supplementary Figures 30–35.

3ξ-(1ξ-Hydroxyethyl)-7-hydroxy-1-isobenzofuranone (5) (F10). UV(MeOH) λ_max_ 207 (3.64), sh 240 (2.98), 303 (2.88) nm. Isomer 1: ^1^H NMR (MeOD, 600 MHz) *δ* 1.26 (3H, d, *J* = 6.5 Hz, H-2'), 4.20 (1H, qd, *J* = 6.5, 3.1 Hz, H-1'), 5.37 (1H, d, *J* = 3.1 Hz, H-3), 6.90 (1H, d, *J* = 7.8 Hz, H-6), 7.05 (1H, d, *J* = 7.8 Hz, H-4), 7.54 (1H, t, *J* = 7.8 Hz, H-5); ^13^C NMR (MeOD, 151 MHz) *δ* 18.9 (C-2'), 68.7 (C-1'), 85.2 (C-3), 113.6 (C-8), 114.7 (C-4), 116.8 (C-6), 137.4 (C-5), 151.0 (C-9), 158.2 (C-7), 172.0 (C-1). Isomer 2: ^1^H NMR (MeOD, 600 MHz) *δ* 1.18 (3H, d, *J* = 6.5 Hz, H-2'), 4.05 (1H, qd, *J* = 6.5, 4.5 Hz, H-1'), 5.34 (1H, d, *J* = 4.5 Hz, H-3), 6.88 (1H, d, *J* = 7.8 Hz, H-6), 7.07 (1H, d, *J* = 7.8 Hz, H4), 7.54 (1H, t, *J* = 7.8 Hz, H-5); ^13^C NMR (MeOD, 151 MHz) *δ* 18.0 (C-2'), 69.7 (C-1'), 85.7 (C-3), 113.1 (C-8), 115.0 (C-4), 116.9 (C-6), 137.4 (C-5), 150.6 (C-9), 158.3 (C-7), 171.9 (C-1). ESI(+)-HRMS *m/z* 195.0652 [M + H]^+^ (calcd. for C_10_H_11_O_4_^+^, 195.0652, Δppm = 0.15). For NMR spectra, see Supplementary Figures 36–41 (Rahman and Gray, 2005).

(2Z,4Z,8E)-6,7-Dihydroxydeca-2,4,8-trienoic acid (6) (F11). UV(MeOH) λ_max_ 205 (3.61), sh 225 (3.51), 257 (3.42) nm. ^1^H NMR (MeOD, 600 MHz) *δ* 1.67 (3H, dd, *J* = 6.5, 1.5 Hz, H-10), 3.92 (1H, t, *J* = 7.1 Hz, H-7), 4.45 (1H, dd, *J* = 9.1, 7.1 Hz, H-6), 5.45 (1H, ddq, *J* = 15.2, 7.1, 1.5 Hz, H-8), 5.72 (1H, dq, *J* = 15.2, 6.5 Hz, H-9), 5.74 (1H, d, *J* = 11.5 Hz, H-2), 5.75 (1H, dd, *J* = 11.5, 9.1 Hz, H-5), 7.01 (1H, t, *J* = 11.5 Hz, H-3), 7.32 (1H, t, *J* = 11.5 Hz, H-4); ^13^C NMR (MeOD, 151 MHz) *δ* 18.0 (C-10), 71.5 (C-6), 76.9 (C-7), 120.8 (C-2), 127.4 (C-4), 129.5 (C-9), 131.1 (C-8), 139.0 (C-5), 139.6 (C-3), 170.0 (C-1). ESI(+)-HRMS *m/z* 199.0964 [M + H]^+^ (calcd. for C_10_H_15_O_4_^+^, 199.0965, Δppm = 0.51). For NMR spectra, see Supplementary Figures 42–47.

(+)-(3R,4S)-4,5-Dihydroxymellein (7) (F13). ${\left[a\right]}_{D}^{25}$27.62 (c.0.115, MeOH); UV(MeOH) λ_max_ 213 (4.01), 222 (4.02) nm. ^1^H NMR (MeOD, 600 MHz) *δ* 1.28 (3H, d, *J* = 6.8 Hz, H-11), 4.85 (1H, overlapped, H-3), 4.94 (1H, d, *J* = 2.5 Hz, H-4), 6.85 (1H, d, *J* = 9.0 Hz, H-7), 7.11 (1H, d, *J* = 9.0 Hz, H-6); ^13^C NMR (MeOD, 151 MHz) *δ* 18.1 (C-11), 64.4 (C-4), 82.7 (C-3), 108.1 (C-9), 119.1 (C-7), 124.2 (C-10), 126.2 (C-6), 148.8 (C-5), 156.1 (C-8), 169.9 (C-1). ESI(+)-HRMS *m/z* 211.0601 [M + H]^+^ (calcd. for C_10_H_11_O_5_^+^, 211.0601, Δppm = 0.10). For NMR spectra, see Supplementary Figures 48–53 (Meepagala et al., 2018).

(-)-(3R,4R)-4-Hydroxymellein (8) (F14). ${\left[a\right]}_{D}^{25}$-17.06 (c.0.18, MeOH); UV(MeOH) λ_max_ 211 (3.88) nm. ^1^H NMR (MeOD, 600 MHz) *δ* 1.52 (3H, d, *J* = 6.6 Hz, H-11), 4.56 (1H, d, *J* = 2.1 Hz, H-4), 4.72 (1H, qd, *J* = 6.6, 2.1 Hz, H-3), 6.97 (2H, m, H-5, H-7), 7.56 (1H, dd, *J* = 8.4, 7.4 Hz, H-6); ^13^C NMR (MeOD, 151 MHz) *δ* 16.3 (C-11), 67.7 (C-4), 80.0 (C-3), 108.4 (C-9), 118.4 (C-7), 119.8 (C-5), 137.7 (C-6), 143.2 (C-10), 162.9 (C-8), 171.0 (C-1). ESI(+)-HRMS *m/z* 195.0654 [M + H]^+^ (calcd. for C_10_H_11_O_4_^+^, 195.0652, Δppm = 1.03). For NMR spectra, see Supplementary Figures 54–59 (Djoukeng et al., 2009).

(-)-(3R,4S)-4-Hydroxymellein (9) (F16). ${\left[a\right]}_{D}^{25}$-6.83 (c.0.1, MeOH); UV(MeOH) λ_max_ 209 (3.57) nm. ^1^H NMR (MeOD, 600 MHz) *δ* 1.47 (3H, d, *J* = 6.3 Hz, H-11), 4.56 (2H, m, H-3, H-4), 6.94 (1H, dd, *J* = 8.4, 1.0 Hz, H-7), 7.08 (1H, dt, *J* = 7.5, 1.0 Hz, H-5), 7.57 (1H, dd, *J* = 8.4, 7.5 Hz, H-6); ^13^C NMR (MeOD, 151 MHz) *δ* 18.2 (C-11), 69.5 (C-4), 81.6 (C-3), 108.0 (C-9), 117.7 (C-5), 117.8 (C-7), 137.8 (C-6), 144.1 (C-10), 162.9 (C-8), 170.2 (C-1). ESI(+)-HRMS *m/z* 195.0652 [M + H]^+^ (calcd. for C_10_H_11_O_4_^+^, 195.0652, Δppm = 0.00). For NMR spectra, see Supplementary Figures 60–65 (Cimmino et al., 2017).

(3S,4R)-3-Carboxy-2-methylene-heptan-4-olide (10) (F19). ^1^H NMR (MeOD, 600 MHz) *δ* 0.99 (3H, t, *J* = 7.4 Hz, H-7), 1.48 (2H, m, H-6), 1.72 (2H, m, H-5), 3.72 (1H, dt, *J* = 5.6, 2.8 Hz, H-3), 4.80 (1H, dt, *J* = 6.7, 5.6 Hz, H-4), 6.00 (1H, d, *J* = 2.8 Hz, H-8'), 6.32 (1H, d, *J* = 2.8 Hz, H-8''); ^13^C NMR (MeOD, 151 MHz) *δ* 14.0 (7), 19.3 (6), 38.7 (5), 81.0 (4), 125.2 (8), 135.6 (2), 170.6 (1), 172.5 (9). ESI(-)-HRMS *m/z* 183.0661 [M-H]^-^ (calcd. for C_9_H_11_O_4_^−^, 183.0663, Δppm = 1.09). For NMR spectra, see Supplementary Figures 68–72 (He et al., 2004).

Decumbic acid (11) (F19). ^1^H NMR (MeOD, 600 MHz) *δ* 0.96 (3H, t, *J* = 7.4 Hz, H-7), 1.48 (2H, m, H-6), 1.59 (1H, m, H-5''), 2.09 (1H, m, H-5'), 2.13 (3H, d, *J* = 2.1 Hz, H-8), 5.15 (1H, m, H-4); ^13^C NMR (MeOD, 151 MHz) *δ* 10.6 (8), 14.1 (7), 19.1 (6), 35.7 (5), 83.0 (4), 136.9 (2), 151.2 (3), 165.0 (9), 175.3 (1). ESI(-)-HRMS *m/z* 183.0660 [M-H]^-^ (calcd. for C_9_H_11_O_4_^−^, 183.0663, Δppm = 1.53). For NMR spectra, see Supplementary Figures 68–71,73 (He et al., 2004).

Decumbic acid B (12) (F20). ${\left[a\right]}_{D}^{25}5.03\;$ (c.0.12, MeOH). ^1^H NMR (MeOD, 600 MHz) *δ* 0.98 (3H, t, *J* = 7.4 Hz, H-7), 1.29 (3H, d, *J* = 7.1 Hz, H-8), 1.46 (1H, m, H-6''), 1.55 (1H, m, H-6'), 1.70 (1H, m, H-5''), 1.82 (1H, m, H-5'), 2.70 (1H, dd, *J* = 11.4, 9.4 Hz, H-3), 2.99 (1H, dq, *J* = 11.4, 7.1 Hz, H-2), 4.49 (1H, td, *J* = 9.4, 8.4, 4.0 Hz, H-4); ^13^C NMR (MeOD, 151 MHz) *δ* 14.1 (C-7), 14.6 (C-8), 19.9 (C-6), 38.0 (C-5), 41.1 (C-2), 55.5 (C-3), 81.4 (C-4), 174.1 (C-9), 179.7 (C-1). ESI(-)-HRMS *m/z* 185.0818 [M-H]^-^ (calcd. for C_9_H_14_O_4_, 185.0819, Δppm = 0.54). For NMR spectra, see Supplementary Figures 75–80 (Zhou et al., 2016).

(-)-(R)-Mellein (13) (F22). ${\left[a\right]}_{D}^{25}$-16.28 (c.0.08, MeOH); UV(MeOH) λ_max_ 208 (3.78), 236 (3.52) nm. ^1^H NMR (MeOD, 600 MHz) *δ* 1.49 (3H, d, *J* = 6.3 Hz, H-11), 2.92 (1H, dd, *J* = 16.4, 11.3 Hz, H-4''), 3.03 (1H, dd, *J* = 16.4, 3.3 Hz, H-4'), 4.75 (1H, dqd, *J* = 11.3, 6.3, 3.3 Hz, H-3), 6.78 (1H, d, *J* = 7.3 Hz, H-5), 6.85 (1H, d, *J* = 8.4 Hz, H-7), 7.45 (1H, dd, *J* = 8.4, 7.3 Hz, H-6); ^13^C NMR (MeOD, 151 MHz) *δ* 20.9 (C-11), 35.2 (C-4), 77.8 (C-3), 109.3 (C-9), 116.7 (C-7), 119.3 (C-5), 137.4 (C-6), 141.6 (C-10), 163.2 (C-8), 171.6 (C-1). ESI(+)-HRMS *m/z* 179.0702 [M + H]^+^ (calcd. for C_10_H_11_O_3_^+^, 179.0703, Δppm = 0.56). For NMR spectra, see Supplementary Figures 81–86 (Djoukeng et al., 2009).

(2R)-Butylitaconic acid (14) (F24). ${\left[a\right]}_{D}^{25}$-1.61 (c.0.14, MeOH); ^1^H NMR (MeOD, 600 MHz) *δ* 0.91 (3H, t, *J* = 7.1 Hz, H-8), 1.32 (2H, m, H-6), 1.35 (2H, m, H-7), 1.69 (1H, m, H-5''), 1.86 (1H, m, H-5'), 3.37 (1H, t, *J* = 7.7 Hz, H-2), 5.60 (1H, s, H-9''), 6.14 (1H, s, H-9'); ^13^C NMR (MeOD, 151 MHz) *δ* 14.3 (C-8), 23.5 (C-7), 31.0 (C-6), 32.1 (C-5), 49.8 (C-2), 125.4 (C-9), 142.7 (C-3), 172.0 (C-4), 178.5 (C-1). ESI(+)-HRMS *m/z* 187.0965 [M + H]^+^ (calcd. for C_9_H_15_O_4_^+^, 187.0965, Δppm = 0.00). For NMR spectra, see Supplementary Figures 87–92 (Nakahashi et al., 2009).

(+)-(R)-CJ-12,372 (15) (F26). ${\left[a\right]}_{D}^{25}$2.0 (c.0.09, MeOH); ^1^H NMR (MeOD, 600 MHz) *δ* 1.89 (1H, m, H-2b), 2.05 (1H, m, H-2a), 2.10 (1H, m, H-3b), 2.35 (1H, ddd, *J* = 14.6, 11.8, 2.9 Hz, H-3a), 5.10 (1H, t, *J* = 4.5 Hz, H-1), 6.77 (1H, d, *J* = 8.8 Hz, H-6), 6.85 (1H, d, *J* = 8.8 Hz, H-7), 6.94 (1H, d, *J* = 7.5 Hz, H-7'), 6.98 (1H, d, *J* = 7.5 Hz, H-2'), 7.46 (1H, t, *J* = 7.5 Hz, H-6'), 7.47 (1H, t, *J* = 7.9 Hz, H-3'), 7.54 (1H, d, *J* = 7.5 Hz, H-5'), 7.56 (1H, d, *J* = 7.5 Hz, H-4'); ^13^C NMR (MeOD, 151 MHz) *δ* 27.3 (C-3), 28.2 (C2), 64.0 (C-1), 110.5 (C-2', C-7'), 114.6 (C-9'), 118.5 (C-6), 118.6 (C-7), 121.5 (C-4', C-5'), 127.1 (C-9), 127.3 (C-10), 128.2 (C-3', C-6'), 135.3 (C-10'), 148.4 (C-1', C-8'), 149.6 (C-8), 150.1 (C-5). ESI(+)-HRMS *m/z* 337.1076 [M + H]^+^ (calcd. for C_20_H_17_O_5_^+^, 337.1071, Δppm = 1.48). For NMR spectra, see Supplementary Figures 93–96 (Sakemi et al., 1995).

(+)-(R)-Palmarumycin EG1 (16) (F29). ${\left[a\right]}_{D}^{25}$92.20 (c.0.02, MeOH); UV(MeOH) λ_max_ 226 (5.07), sh 207(4.94) nm. ^1^H NMR (MeOD, 600 MHz) *δ* 1.91 (1H, tt, *J* = 14.0, 3.1 Hz, H-2b), 2.08 (1H, dq, *J* = 14.0, 3.1 Hz, H-2a), 2.18 (1H, dt, *J* = 14.0, 3.1 Hz, H-3b), 2.25 (1H, td, *J* = 14.0, 3.1 Hz, H-3a), 3.44 (3H, s, OMe), 4.71 (1H, t, *J* = 3.1 Hz, H-1), 6.79 (1H, d, *J* = 8.7 Hz, H-6), 6.86 (1H, d, *J* = 8.7 Hz, H-7), 6.92 (1H, d, *J* = 7.5 Hz, H-7'), 6.98 (1H, d, *J* = 7.5 Hz, H-2'), 7.46 (0H, t, *J* = 7.5 Hz, H-6'), 7.47 (1H, t, *J* = 7.5 Hz, H-3'), 7.54 (1H, d, *J* = 7.5 Hz, H-5'), 7.56 (1H, d, *J* = 7.5 Hz, H-4'); ^13^C NMR (MeOD, 151 MHz) *δ* 24.1 (C-2), 27.0 (C-3), 57.1 (OMe), 71.8 (C-1), 103.7 (C-4), 110.8 (C-7'), 110.8 (C-2'), 115.0 (C-9'), 118.9 (C-7), 119.2 (C-6), 121.6 (C-5'), 122.0 (C-4'), 122.0 (C-10), 125.7 (C-9), 128.5 (C-3'), 128.6 (C-6'), 135.7 (C-10'), 148.6 (C-1'), 149.1 (C-8'), 150.1 (C-8), 150.5 (C-5). ESI(+)-HRMS *m/z* 351.1227 [M + H]^+^ (calcd. for C_21_H_19_O_5_^+^, 351.1227, Δppm = 0.10). For NMR spectra, Supplementary Figures 97–102 (Macías-Rubalcava et al., 2014).

(R)-4-Methoxy-3,4-dihydro-2H-spiro[naphthalene-1,2'-naphtho[1,8-de][1,3]dioxin]-6-ol (17) (F32). ${\left[a\right]}_{D}^{25}$ 47.9(c. 0.04, MeOH); UV(MeOH) λ_max_ 226 (4.17), sh 214 (4.06) nm. ^1^H NMR (MeOD, 600 MHz) *δ* 2.02 (1H, m, H-2b), 2.12 (1H, m, H-2a), 2.14 (1H, m, H-3b), 2.20 (1H, m, H-3a), 3.47 (3H, s, OMe), 4.73 (1H, t, *J* = 3.2 Hz, H-1), 6.85 (1H, dd, *J* = 7.4, 0.8 Hz, H-7'), 6.92 (1H, dd, *J* = 7.4, 0.9 Hz, H-2'), 6.93 (1H, dd, *J* = 6.8, 2.3 Hz, H-6), 7.26 (1H, d, *J* = 2.3 Hz, H-8), 7.27 (1H, d, *J* = 6.8 Hz, H-5), 7.43 (1H, dd, *J* = 8.4, 7.4 Hz, H-6'), 7.45 (1H, dd, *J* = 8.4, 7.4 Hz, H-3'), 7.50 (1H, dd, *J* = 8.4, 0.8 Hz, H-5'), 7.51 (1H, dd, *J* = 8.4, 0.9 Hz, H-4'); ^13^C NMR (MeOD, 151 MHz) *δ* 23.8 (C-2), 26.1 (C-3), 56.8 (OMe), 71.6 (C-1), 101.1 (C-4), 109.9 (C-2', C-7'), 114.5 (C-9'), 116.7 (C-6), 119.0 (C-8), 121.1 (C-4', C-5'), 128.2 (C-3', C-6'), 130.0 (C-5), 135.3 (C-10'), 148.9 (C-8'), 149.4 (C-1'). ESI(−)-HRMS *m/z* 333.1134 [M−H]^−^ (calcd. for C_21_H_17_O_4_^+^, 333.1132, Δppm = 0.51). For NMR spectra, see Supplementary Figures 103–106.

(R)-(-)-5-Hydroxymellein (18) (F19). ${\left[a\right]}_{D}^{25}$ -31.0 (c. 0.007, MeOH); UV(MeOH) λ_max_ 224 (5.3), sh 26(4.4), 351 (4.2)nm. ^1^H NMR (MeOD, 600 MHz) *δ* 1.50 (3H, d, *J* = 6.3 Hz, H-11), 2.62 (1H, ddd, *J* = 16.9, 11.3, 0.9 Hz, H-4b), 3.17 (1H, dd, *J* = 16.9, 3.4 Hz, H-4a), 4.70 (1H, dqd, *J* = 11.3, 6.3, 3.4 Hz, H-3), 6.71 (1H, dd, *J* = 8.9, 0.9 Hz, H-7), 7.02 (1H, d, *J* = 8.9 Hz, H-6); ^13^C NMR (MeOD, 151 MHz) *δ* 21.1 (C-11), 29.4 (C-4), 77.7 (C-3), 109.2 (C-9), 116.5 (C-7), 125.1 (C-6), 125.9 (C-10), 146.9 (C-5), 156.4 (C-8), 171.8 (C-1). ESI(+)-HRMS *m/z* 195.0652 [M + H]^+^ (calcd. for C_10_H_11_O_4_^+^, 195.0652, Δppm = 0.00). For NMR spectra, see Supplementary Figures 68–71,74 (Salvatore et al., 2020).

### Preparation of the *S*- and *R*-*α*-Methoxy-*α*-Trifluoromethylphenylacetyl Chloride Esters Derivatives 6*S* and 6*R* for Compound 1 (59)

*R*-MTPA ester derivative: to a stirred solution of 1 (1, 1.3 mg, 0.0061 mmol) and dry deuterated pyridine-d5 (19 μL, 0.24 mmol, 39 equiv.) in dry deuterochloroform CDCl_3_ (100 μL, [pyridine-d5] = 0.32 M) at room temperature, (*S*)-(+)-MTPA-Cl (3*S*, 16 μL, 0.086 mmol, 14 equiv.) was added under inert atmosphere (Argon). The reaction was left for 2 h. The reaction mixture was then diluted in dry CDCl_3_ (0.6 ml) and submitted to ^1^H-NMR analysis. This gave the *R*-MTPA-methyl ester of 1 (34 mg) as a brown oil: ^1^H NMR (CDCl_3_, 600 MHz) *δ* 1.31 (3H, d, *J* = 6.5 Hz, H-8), 3.44 (3H, d, *J* = 1.3 Hz, OMe), 3.94 (1H, dd, *J* = 5.9, 2.9 Hz, H-6), 4.17 (1H, dq, *J* = 4.8, 2.2 Hz, H-2), 4.54 (1H, dt, *J* = 5.2, 2.9, 2.2 Hz, H-5), 5.12 (1H, qd, *J* = 6.5, 4.8 Hz, H-7), 5.91 (1H, d, *J* = 10.3 Hz, H-3), 6.03 (1H, ddd, *J* = 10.3, 5.2, 2.2 Hz, H-4), 6.11 (1H, d, *J* = 9.7 Hz, H-2'), 6.71 (1H, dd, *J* = 9.7, 5.8 Hz, H-1'); ^13^C NMR (CDCl_3_, 151 MHz) *δ* 13.7 (C-8), 54.3 (OMe), 64.0 (C6), 68.1 (C-5), 72.6 (C-7), 74.9 (C-2), 122.4 (C-4), 123.6 (C-2'), 130.5 (C-3), 138.7 (C-1'). (Supplementary Figures 13–15). *S*-MTPA ester derivative: to a stirred solution of 1 (1, 1.3 mg, 0.0061 mmol) and dry deuterated pyridine-d5 (19 μL, 0.24 mmol, 39 equiv.) in dry deuterochloroform CDCl_3_ (100 μL [pyridine-d5] = 0.32 M) at room temperature, (*R*)-(-)-MTPA-Cl (3*S*, 18.3 μL, 0.098 mmol, 16 equiv.) was added under inert atmosphere (Argon). The reaction was left for 2 h. The reaction mixture was then diluted in dry CDCl_3_ (0.6 ml) and submitted to ^1^H-NMR analysis. This gave the *S*-MTPA-methyl ester of **1** (19.9 mg) as a brown oil: ^1^H NMR (CDCl_3_, 600 MHz) *δ* 1.23 (3H, d, *J* = 6.6 Hz, H-8), 3.46 (3H, d, *J* = 1.3 Hz, OMe), 4.03 (1H, dd, *J* = 5.8, 2.9 Hz, H-6), 4.27 (1H, dq, *J* = 3.1, 2.2 Hz, H-2), 4.59 (1H, dt, *J* = 5.1, 2.5 Hz, H-5), 5.21 (1H, qd, *J* = 6.6, 3.1 Hz, H-7), 6.04 (1H, d, *J* = 10.2 Hz, H-3), 6.09 (1H, ddd, *J* = 10.2, 5.1, 2.2 Hz, H-4), 6.16 (1H, d, *J* = 9.8 Hz, H-2'), 6.80 (1H, dd, *J* = 9.8, 5.8 Hz, H-1'); ^13^C NMR (CDCl_3_, 151 MHz) *δ* 13.7 (C-8), 54.5 (OMe), 64.0 (C6), 68.2 (C-5), 72.4 (C-7), 75.3 (C-2), 122.7 (C-4), 123.8 (C-2'), 130.1 (C-3), 138.7 (C-1') (Supplementary Figures 16–18).

The raw NMR data files of the isolated compounds are available at the following link: https://doi.org/10.26037/yareta:k4p3ozyehbhg5nr4s7iuc5tkf4

### MTT Assay

Indicated cell lines were detached and resuspended at 120,000 cells/ml and added to each well of a transparent 384-well plate in the final volume of 25 µl/well. The cells were maintained in DMEM containing 10% FBS at 37°C, 5% CO_2_ overnight. The next day, the medium in each well was replaced with 40 μl of the fresh one containing the indicated concentrations of compounds. After incubation for 3 days, the medium in each well was replaced with 25 μl of 0.5 mg/ml thiazolyl blue solution in 1xPBS followed by incubation for 3 h at 37°C. Then, the solution was removed and 25 μl DMSO was added to each well. Absorbance at 510 nm was measured in a Tecan Infinite M200 PRO plate reader.

### Wnt Activity Evaluation

The Wnt-induced and basal luciferase activities were analyzed as described (Koval et al., 2014; Shaw et al., 2019b). BT-20 TNBC cell line stably transfected with TopFlash reporter plasmid was seeded at 240,000 cells/ml in a white opaque 384-well plate in the final volume of 25 μl. The cells were maintained incubated at 37°C, 5% CO_2_ overnight for attachment. Subsequently, they were transfected by a plasmid encoding Renilla luciferase under the CMV promoter as described in the manufacturer’s protocol using 12 μg/ml of DNA and 40 μl/ml XtremeGENE 9 reagent. The next day, the medium in each well was replaced with a 20 μl fresh medium containing Wnt3a (2.5 μg/ml) or GSK3β inhibitor CHIR99021 (Koval and Katanaev, 2011). After overnight incubation, the supernatant in each well was removed, and the luciferase activity was measured as described (Koval et al., 2014).

To measure the activity of luciferases, the culture medium was completely removed from all wells of the plate. Next, the luciferase activity of the firefly and Renilla luciferases was detected sequentially in individual wells of a 384-well plate through injection of corresponding measurement solutions in Infinite M Plex multifunctional plate reader with injection module (Dyer et al., 2000).

## Data Availability

The raw data supporting the conclusions of this article will be made available by the authors, without undue reservation.
