# Supplementary material for: Isolation and Identification of Isocoumarin Derivatives With Specific Inhibitory Activity Against Wnt Pathway and Metabolome Characterization of Lasiodiplodia venezuelensis
Source: Front Chem. 2021 Aug 12;9:664489. doi: 10.3389/fchem.2021.664489 (PMC8397479; doi:10.3389/fchem.2021.664489)
Supplement: Supplementary file 2 [file DataSheet1.PDF]

Supplementary data

Isolation and identification of isocoumarin derivatives with specific inhibitory activity against Wnt pathway and metabolome characterization of *Lasiodiplodia venezuelensis.*

Léonie Pellissier[1,2*], Alexey Koval[3], Laurence Marcourt[1,2], Emerson Ferreira Queiroz[1,2], Nicole Lecoultre[4], Sara Leoni[9], Luis-Manuel Quiros-Guerrero[1,2], Morgane Barthélémy[5], Bastiaan L. Duivelshof[1,2], Davy Guillarme[1,2], Sébastien Tardy[1,2], Véronique Eparvier[5], Karl Perron[2,9], Jérome Chave[6], Didier Stien[7], Katia Gindro[4], Vladimir Katanaev[3,8], Jean-Luc Wolfender[1,2*]

* Corresponding authors at: Institute of Pharmaceutical Sciences of Western Switzerland, CMU - rue Michel Servet 1, CH-1211 Geneva 4, Switzerland. *E-mail address*: Jean-Luc.Wolfender@unige.ch (J-L. Wolfender); leonie.pellissier@unige.ch (L. Pellissier)

Supplementary Figure 1. A) HPLC-ELSD and B) HPLC-PDA (254nm) analysis of the different extracts obtained from the A02 strain: ethyle acetate (A02Et) and water extract (A02W), and enriched hydro-alcoholic fraction obtained after degreasing (A02EtM).

Supplementary Figure 2. HPLC-UV (black: 254nm and pink: 280nm) semi-preparative fractionation profiles of the 4 dry load injections showing an excellent reproductivity.

Supplementary Figure 3. HPLC-UV semi-preparative fractionation profile with fractions isolated as single peaks, based on UV signal.

Supplementary Figure 4. UHPLC-HRMS-ESI profiles of fraction 19 in A) positive and B) negative mode

Supplementary Figure 5. Alignment of the 75 UHPLC-UV profiles of the injections showing the consistency of the UHPLC-UV microfractionation

Supplementary Figure 6. ^{1}H NMR spectrum of compound **1** in CD_3OD at 600 MHz.

Supplementary Figure 7. COSY NMR spectrum of compound **1** in CD_3OD at 600 MHz.

Supplementary Figure 8. ^{1}C NMR spectrum of compound **1** in CD_3OD at 600 MHz.

Supplementary Figure 9. HSQC NMR spectrum of compound **1** in CD_3OD at 600 MHz.

Supplementary Figure 10. HMBC NMR spectrum of compound **1** in CD_3OD at 600 MHz.

Supplementary Figure 11. ROESY NMR spectrum of compound **1** in CD_3OD at 600 MHz.

Supplementary Figure 12. Synthesis of the *R*- and *S*- MTPA esters from compound **1**.
Supplementary Figure 13. ^{1}H NMR spectrum of **1** *R*MTPA in $CDCl_3$ at 600 MHz.
Supplementary Figure 14. COSY NMR spectrum of **1** *R*MTPA in $CDCl_3$ at 600 MHz.
Supplementary Figure 15. Edited HSQC NMR spectrum of **1** *R*MTPA in $CDCl_3$ at 600 MHz.
Supplementary Figure 16. ^{1}H NMR spectrum of **1** *S*MTPA in $CDCl_3$ at 600 MHz.
Supplementary Figure 17. COSY NMR spectrum of **1** *S*MTPA in $CDCl_3$ at 600 MHz.
Supplementary Figure 18. Edited HSQC NMR spectrum of **1** *S*MTPA in $CDCl_3$ at 600 MHz.
Supplementary Figure 19. ^{1}H NMR spectrum of **2** in MeOD at 600 MHz.
Supplementary Figure 20. COSY NMR spectrum of **2** in MeOD at 600 MHz.
Supplementary Figure 21. ^{1}C NMR spectrum of **2** in MeOD at 600 MHz.
Supplementary Figure 22. HSQC NMR spectrum of **2** in MeOD at 600 MHz.
Supplementary Figure 23. HMBC NMR spectrum of **2** in MeOD at 600 MHz.
Supplementary Figure 24. ROESY NMR spectrum of **2** in MeOD at 600 MHz.
Supplementary Figure 25. ^{1}H NMR spectrum of **3** in DMSO-d_6 at 600 MHz.
Supplementary Figure 26. COSY NMR spectrum of **3** in DMSO-d_6 at 600 MHz.
Supplementary Figure 27. HSQC NMR spectrum of **3** in DMSO-d_6 at 600 MHz.
Supplementary Figure 28. HMBC NMR spectrum of **3** in DMSO-d_6 at 600 MHz.
Supplementary Figure 29. ROESY NMR spectrum of **3** in DMSO-d_6 at 600 MHz.
Supplementary Figure 30. ^{1}H NMR spectrum of **4** in MeOD at 600 MHz.
Supplementary Figure 31. COSY NMR spectrum of **4** in MeOD at 600 MHz.
Supplementary Figure 32. ^{1}C NMR spectrum of **4** in MeOD at 600 MHz.
Supplementary Figure 33. HSQC NMR spectrum of **4** in MeOD at 600 MHz.
Supplementary Figure 34. HMBC NMR spectrum of **4** in MeOD at 600 MHz.
Supplementary Figure 35. ROESY NMR spectrum of **4** in MeOD at 600 MHz.
Supplementary Figure 36. ^{1}H NMR spectrum of **5** in MeOD at 600 MHz.
Supplementary Figure 37. COSY NMR spectrum of **5** in MeOD at 600 MHz.
Supplementary Figure 38. ^{1}C NMR spectrum of **5** in MeOD at 600 MHz.
Supplementary Figure 39. HSQC NMR spectrum of **5** in MeOD at 600 MHz.
Supplementary Figure 40. HMBC NMR spectrum of **5** in MeOD at 600 MHz.
Supplementary Figure 41. ROESY NMR spectrum of **5** in MeOD at 600 MHz.
Supplementary Figure 42. ^{1}H NMR spectrum of **6** in MeOD at 600 MHz.
Supplementary Figure 43. COSY NMR spectrum of **6** in MeOD at 600 MHz.
Supplementary Figure 44. ^{1}C NMR spectrum of **6** in MeOD at 600 MHz.
Supplementary Figure 45. HSQC NMR spectrum of **6** in MeOD at 600 MHz.
Supplementary Figure 46. HSQC NMR spectrum of **6** in MeOD at 600 MHz.
Supplementary Figure 47. ROESY NMR spectrum of **6** in MeOD at 600 MHz.
Supplementary Figure 48. ^{1}H NMR spectrum of **7** in MeOD at 600 MHz.
Supplementary Figure 49. COSY NMR spectrum of **7** in MeOD at 600 MHz.
Supplementary Figure 50. ^{1}C NMR spectrum of **7** in MeOD at 600 MHz.
Supplementary Figure 51. HSQC NMR spectrum of **7** in MeOD at 600 MHz.
Supplementary Figure 52. HMBC NMR spectrum of **7** in MeOD at 600 MHz.
Supplementary Figure 53. ROESY NMR spectrum of **7** in MeOD at 600 MHz.
Supplementary Figure 54. ^{1}H NMR spectrum of **8** in MeOD at 600 MHz.
Supplementary Figure 55. COSY NMR spectrum of **8** in MeOD at 600 MHz.
Supplementary Figure 56. ^{1}C NMR spectrum of **8** in MeOD at 600 MHz.
Supplementary Figure 57. HSQC NMR spectrum of **8** in MeOD at 600 MHz.
Supplementary Figure 58. HMBC NMR spectrum of **8** in MeOD at 600 MHz.
Supplementary Figure 59. ROESY NMR spectrum of **8** in MeOD at 600 MHz.

Supplementary Figure 60. ^{1}H NMR spectrum of **9** in MeOD at 600 MHz.
Supplementary Figure 61. COSY NMR spectrum of **9** in MeOD at 600 MHz.
Supplementary Figure 62. ^{1}C NMR spectrum of **9** in MeOD at 600 MHz.
Supplementary Figure 63. HSQC NMR spectrum of **9** in MeOD at 600 MHz.
Supplementary Figure 64. HMBC NMR spectrum of **9** in MeOD at 600 MHz.
Supplementary Figure 65. ROESY NMR spectrum of **9** in MeOD at 600 MHz.
Supplementary Figure 66. ^{1}H NMR spectrum of **10, 11** and **18** (fraction F19) in MeOD at 600 MHz.
Supplementary Figure 67. COSY NMR spectrum of **10, 11** and **18** (fraction F19) in MeOD at 600 MHz.
Supplementary Figure 68. ^{1}C NMR spectrum of **10,11** and **18** (fraction F19) in MeOD at 600 MHz.
Supplementary Figure 69. HSQC NMR spectrum of **10,11** and **18** (fraction F19) in MeOD at 600 MHz.
Supplementary Figure 70. HMBC NMR spectrum of **10,11** and **18** (fraction F19) in MeOD at 600 MHz.
Supplementary Figure 71. ROESY NMR spectrum of **10,11** and **18** (fraction F19) in MeOD at 600 MHz.
Supplementary Figure 72. ^{1}H NMR spectrum of **10** MeOD at 600 MHz.
Supplementary Figure 73. ^{1}H NMR spectrum of **11** MeOD at 600 MHz.
Supplementary Figure 74. ^{1}H NMR spectrum of **18** MeOD at 600 MHz.
Supplementary Figure 75. ^{1}H NMR spectrum of **12** in MeOD at 600 MHz.
Supplementary Figure 76. COSY NMR spectrum of **12** in MeOD at 600 MHz.
Supplementary Figure 77. ^{1}C NMR spectrum of **12** in MeOD at 600 MHz.
Supplementary Figure 78. HSQC NMR spectrum of **12** in MeOD at 600 MHz.
Supplementary Figure 79. HMBC NMR spectrum of **12** in MeOD at 600 MHz.
Supplementary Figure 80. ROESY NMR spectrum of **12** in MeOD at 600 MHz.
Supplementary Figure 81. ^{1}H NMR spectrum of **13** in MeOD at 600 MHz.
Supplementary Figure 82. COSY NMR spectrum of **13** in MeOD at 600 MHz.
Supplementary Figure 83. ^{1}C NMR spectrum of **13** in MeOD at 600 MHz.
Supplementary Figure 84. HSQC NMR spectrum of **13** in MeOD at 600 MHz.
Supplementary Figure 85. HMBC NMR spectrum of **13** in MeOD at 600 MHz.
Supplementary Figure 86. ROESY NMR spectrum of **13** in MeOD at 600 MHz.
Supplementary Figure 87. ^{1}H NMR spectrum of **14** in MeOD at 600 MHz.
Supplementary Figure 88. COSY NMR spectrum of **14** in MeOD at 600 MHz.
Supplementary Figure 89. ^{1}C NMR spectrum of **14** in MeOD at 600 MHz.
Supplementary Figure 90. HSQC NMR spectrum of **14** in MeOD at 600 MHz.
Supplementary Figure 91. HMBC NMR spectrum of **14** in MeOD at 600 MHz.
Supplementary Figure 92. ROESY NMR spectrum of **14** in MeOD at 600 MHz.
Supplementary Figure 93. ^{1}H NMR spectrum of **15** in MeOD at 600 MHz.
Supplementary Figure 94. COSY NMR spectrum of **15** in MeOD at 600 MHz.
Supplementary Figure 95. HSQC NMR spectrum of **15** in MeOD at 600 MHz.
Supplementary Figure 96. HMBC NMR spectrum of **15** in MeOD at 600 MHz.
Supplementary Figure 97. ^{1}H NMR spectrum of **16** in MeOD at 600 MHz.
Supplementary Figure 98. COSY NMR spectrum of **16** in MeOD at 600 MHz.
Supplementary Figure 99. ^{1}C NMR spectrum of **16** in MeOD at 600 MHz.
Supplementary Figure 100. HSQC NMR spectrum of **16** in MeOD at 600 MHz.
Supplementary Figure 101. HMBC NMR spectrum of **16** in MeOD at 600 MHz.
Supplementary Figure 102. ROESY NMR spectrum of **16** in MeOD at 600 MHz.
Supplementary Figure 103. ^{1}H NMR spectrum of **17** in MeOD at 600 MHz.

Supplementary Figure 104. COSY NMR spectrum of **17** in MeOD at 600 MHz.
Supplementary Figure 105. HSQC NMR spectrum of **17** in MeOD at 600 MHz.
Supplementary Figure 106. HMBC NMR spectrum of **17** in MeOD at 600 MHz.

Molecular Networking

Supplementary Figure 107. Cluster C_28
Supplementary Figure 108. Cluster C_23
Supplementary Figure 109. Clusters C_43, C_164, C_1497
Supplementary Figure 110. Clusters C_97
Supplementary Figure 111. UHPLC-HRMS-ESI (-) analysis of compound **12**
Supplementary Figure 112. Cluster C_72 and single node corresponding to compound **17**
Supplementary Figure 113. Cluster C_33
Supplementary Figure 114. Cluster C_19
Supplementary Figure 115. Cluster C_118
Supplementary Figure 116. Clusters C_961, C_193, C_717, C_937
Supplementary Figure 117. Cluster C_1
Supplementary Figure 118. Cluster C_395
Supplementary Figure 119. Cluster C_73
Supplementary Figure 120. Cluster C_20

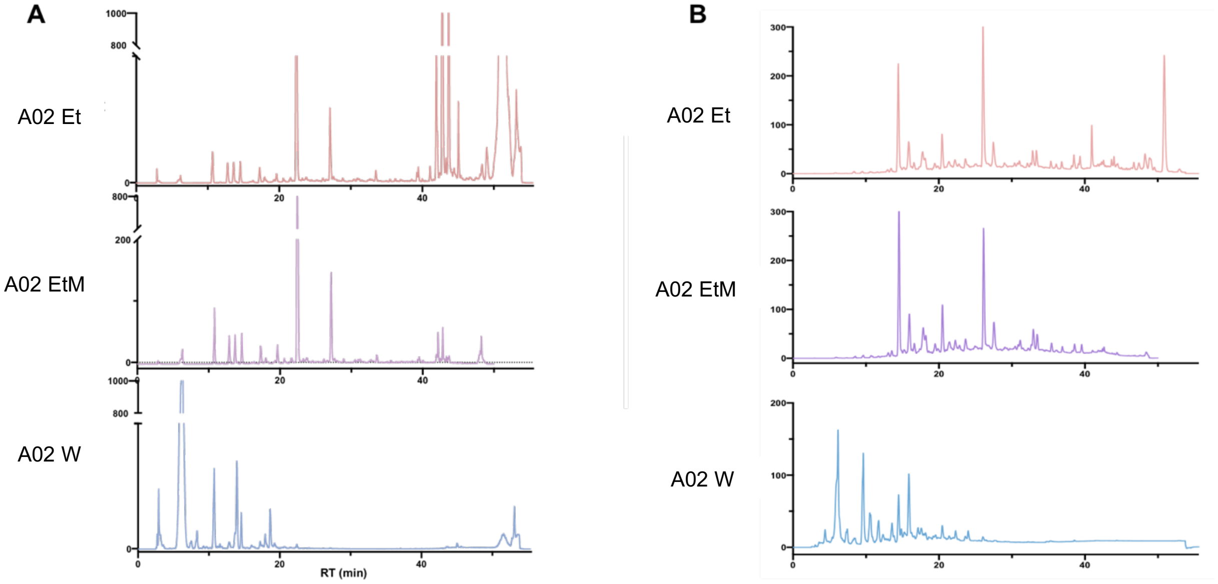

Supplementary Figure 1. A) HPLC-ELSD and B) HPLC-PDA (254nm) analysis of the different extracts obtained from the A02 strain: ethyle acetate (A02Et) and water extract (A02W), and enriched hydro-alcoholic fraction obtained after degreasing (A02EtM).

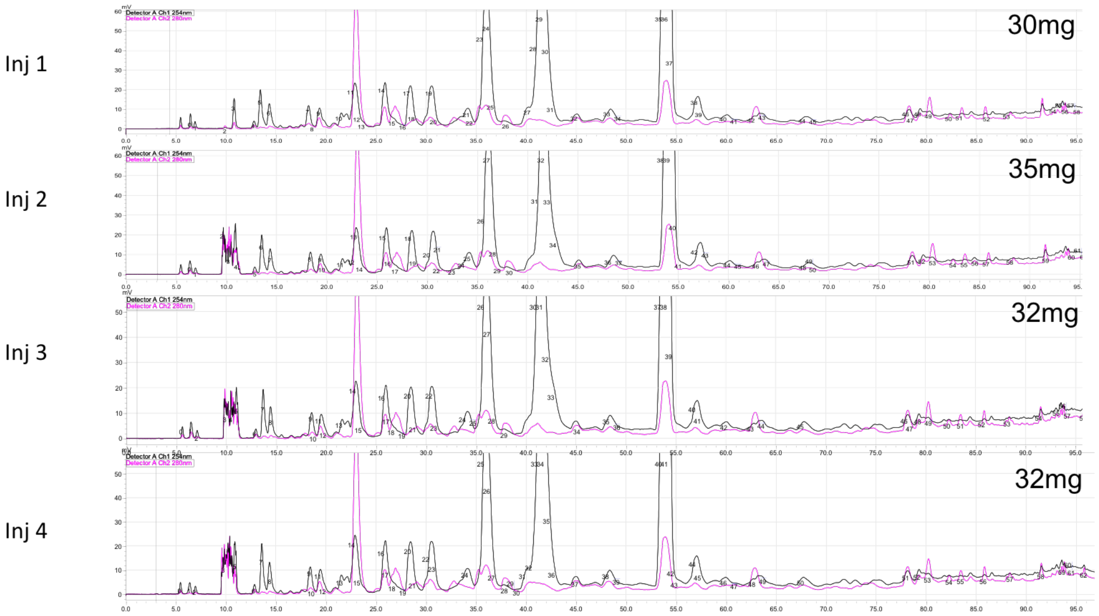

Supplementary Figure 2. HPLC-UV (black: 254nm and pink: 280nm) semi-preparative fractionation profiles of the 4 dry load injections showing an excellent reproductivity.

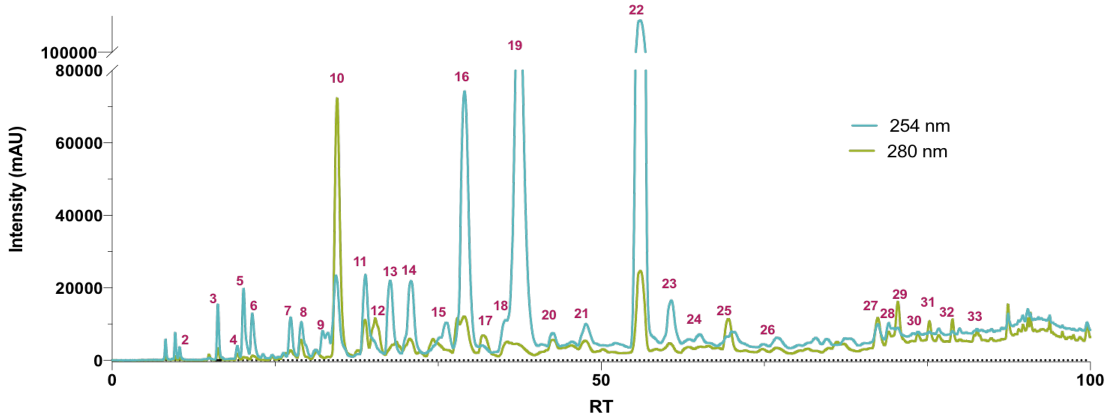

Supplementary Figure 3. HPLC-UV semi-preparative fractionation profile with fractions isolated as single peaks, based on UV signal.

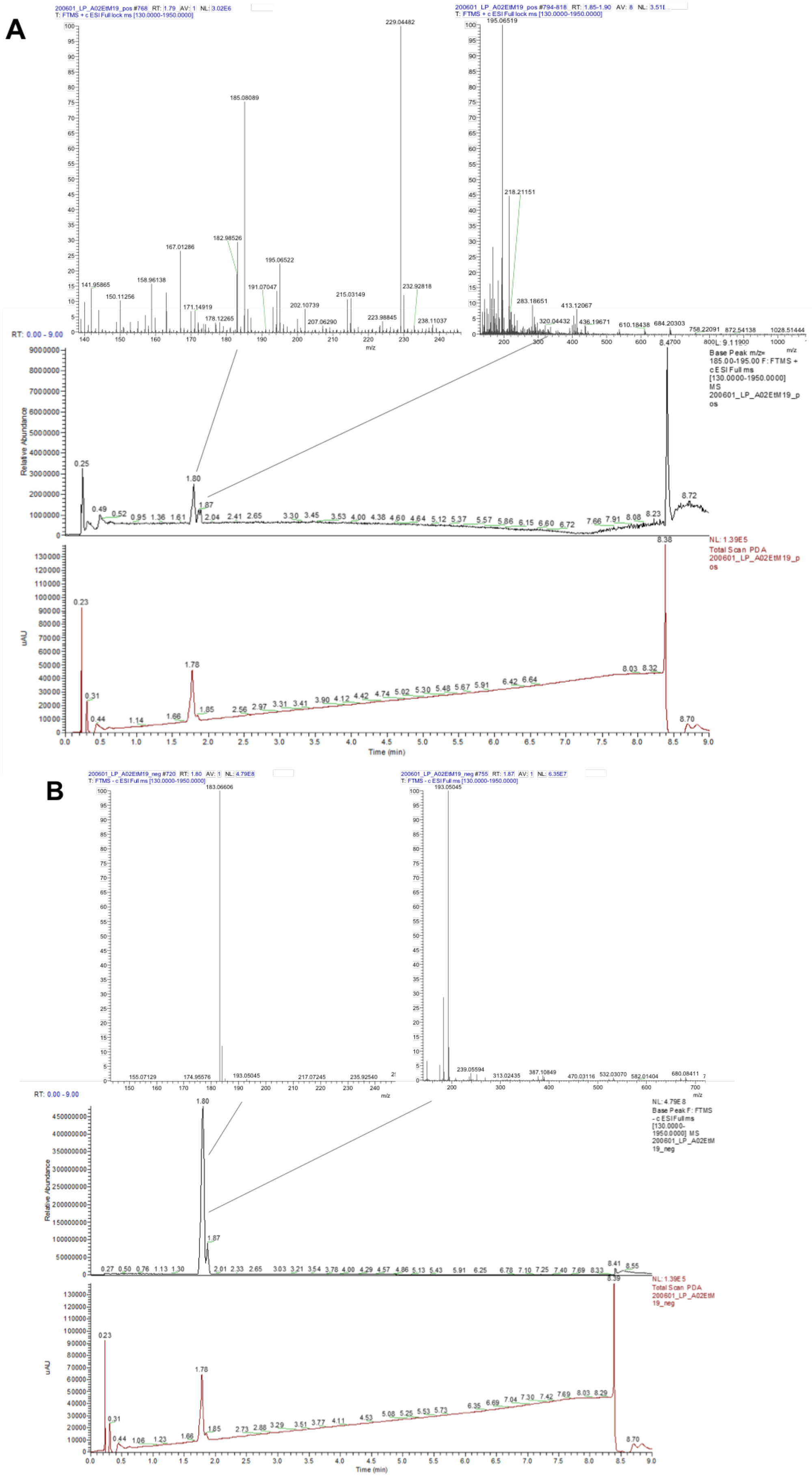

Supplementary Figure 4. UHPLC-HRMS-ESI profiles of fraction 19 in A) positive and B) negative mode

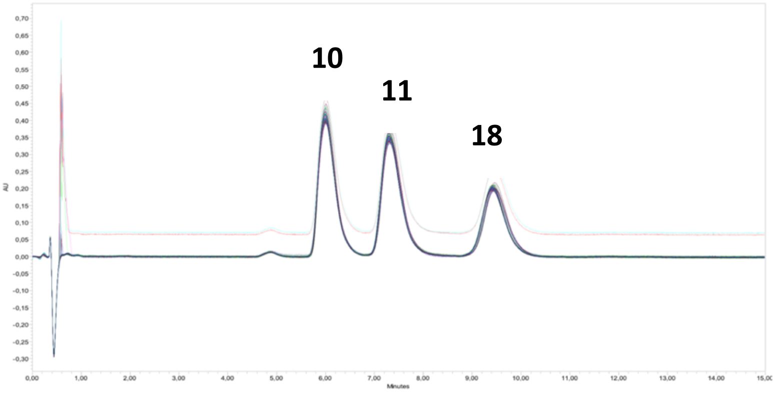

Supplementary Figure 5. Alignment of the 75 UHPLC-UV profiles of the injections showing the consistency of the UHPLC-UV microfractionation

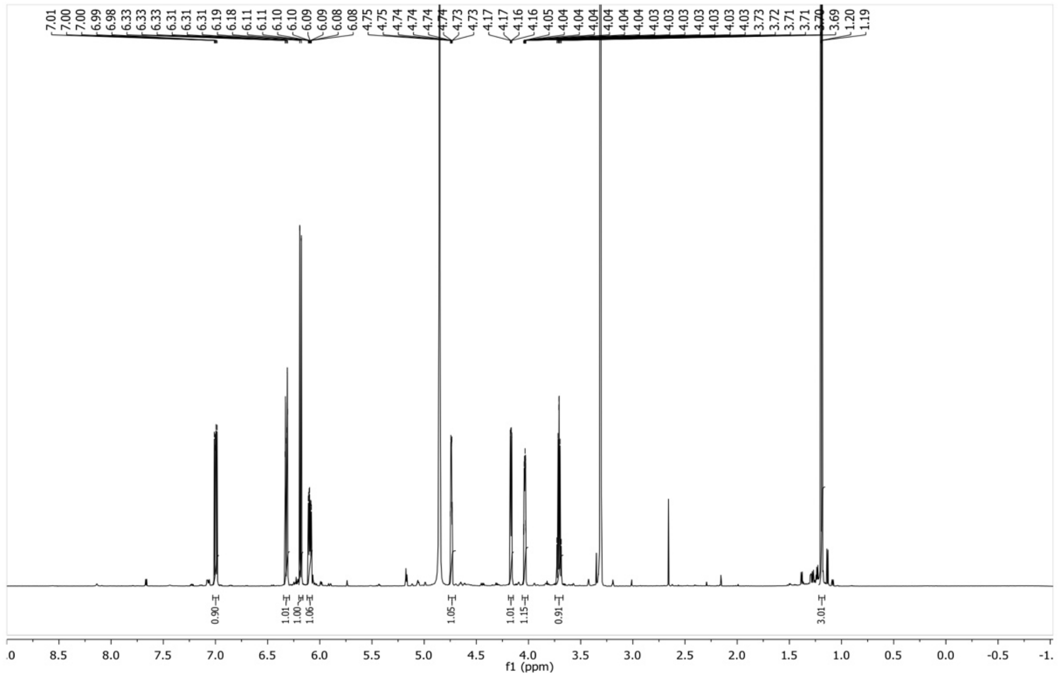

Supplementary Figure 6. ^{1}H NMR spectrum of compound **1** in CD_3OD at 600 MHz.

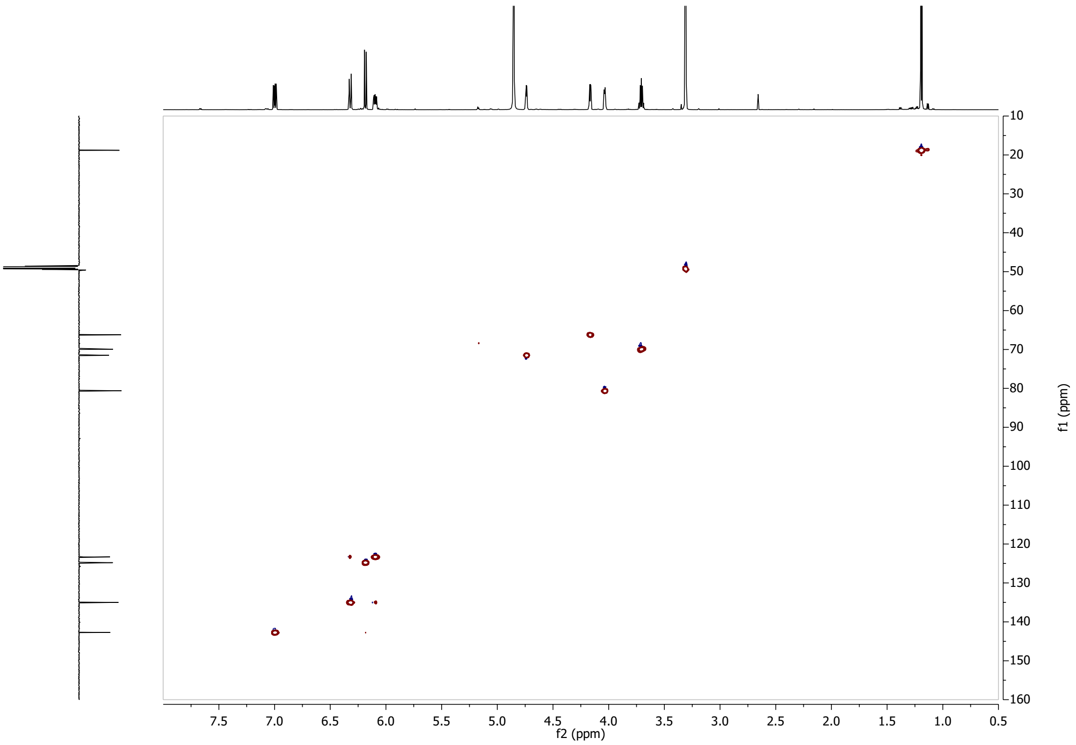

Supplementary Figure 7. COSY NMR spectrum of compound **1** in CD_3OD at 600 MHz.

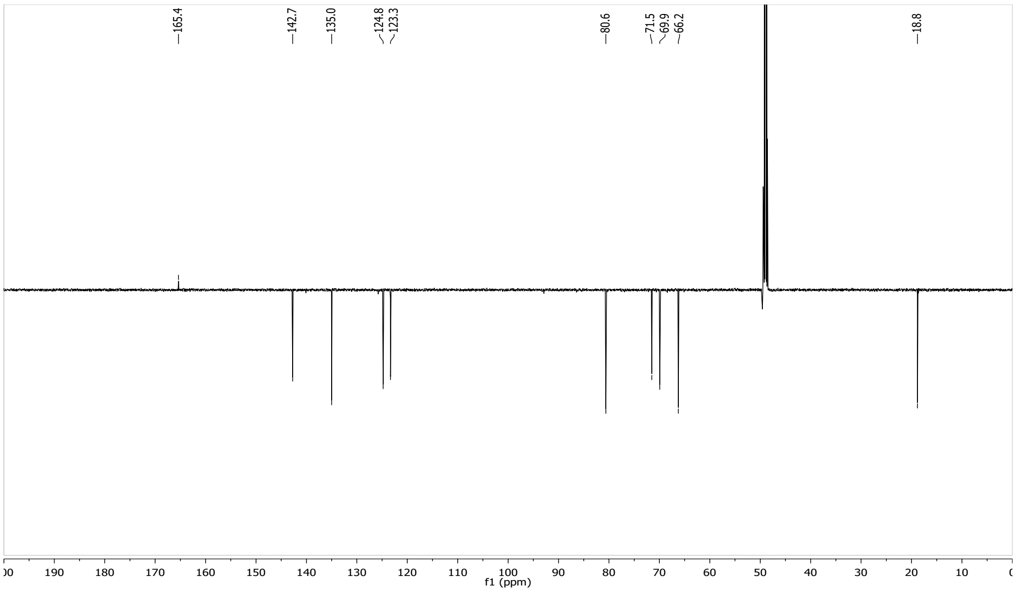

Supplementary Figure 8. ^{1}C NMR spectrum of compound **1** in CD_3OD at 600 MHz.

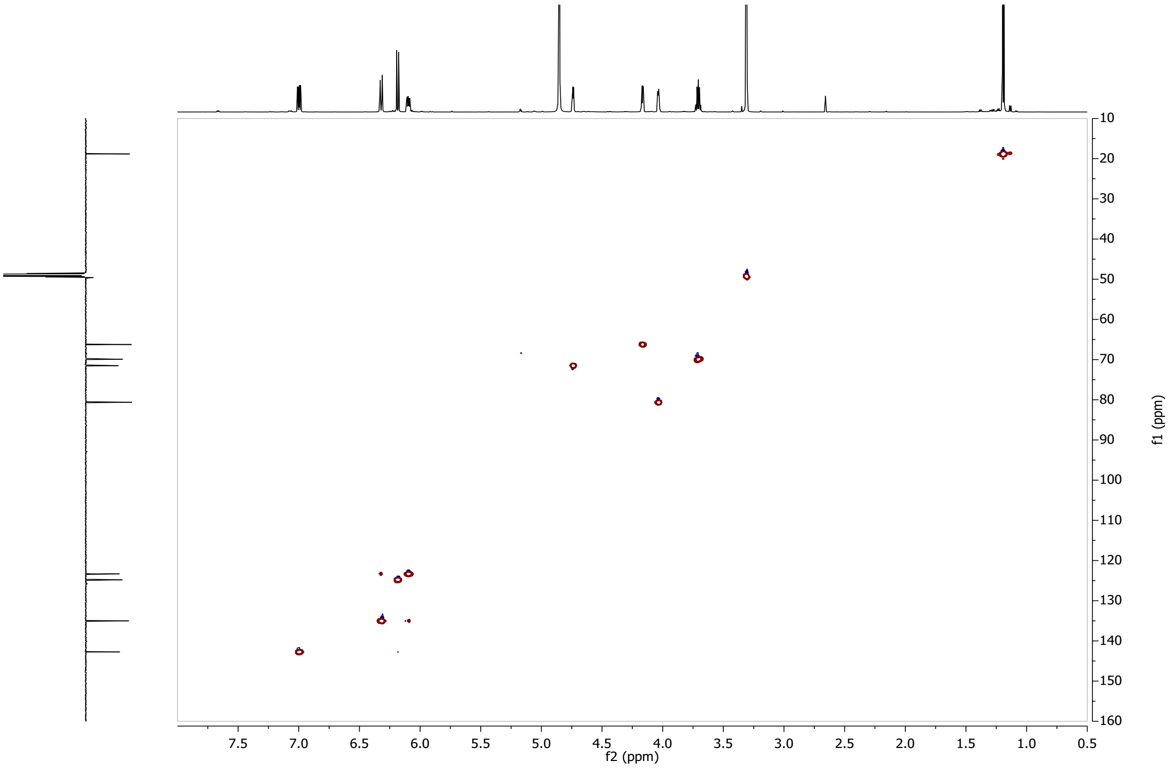

Supplementary Figure 9. HSQC NMR spectrum of compound **1** in CD_3OD at 600 MHz.

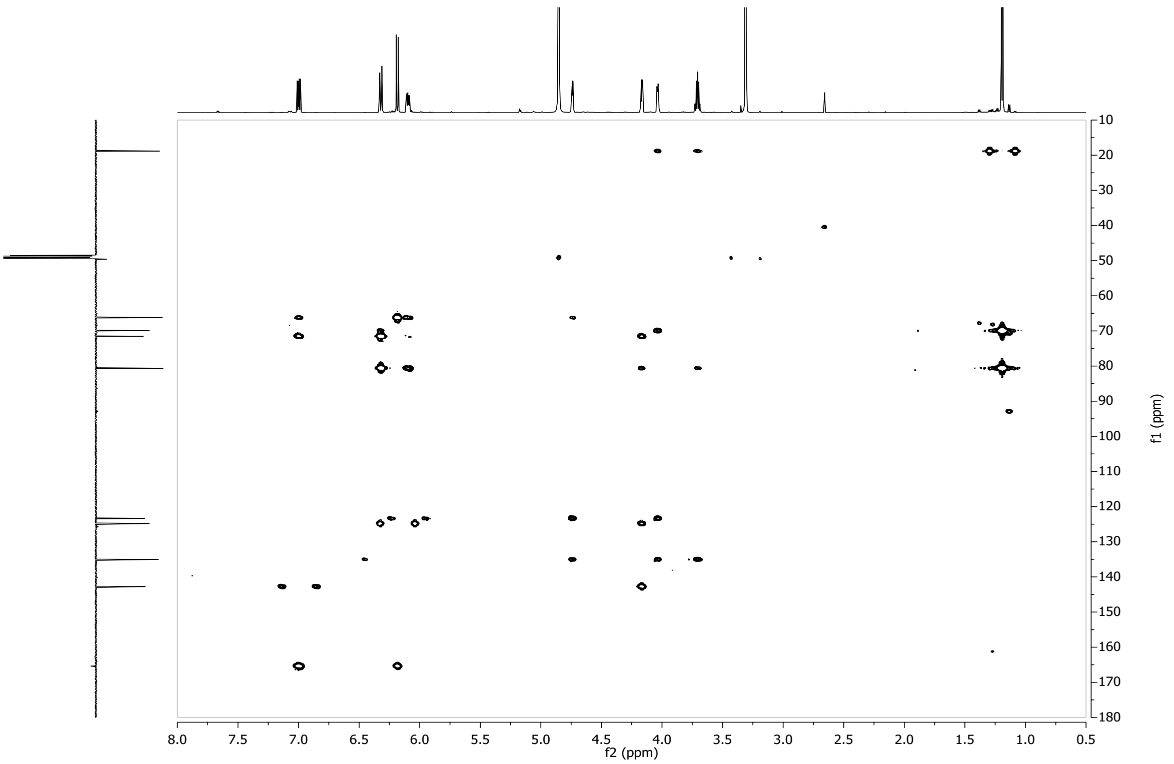

Supplementary Figure 10. HMBC NMR spectrum of compound **1** in CD_3OD at 600 MHz.

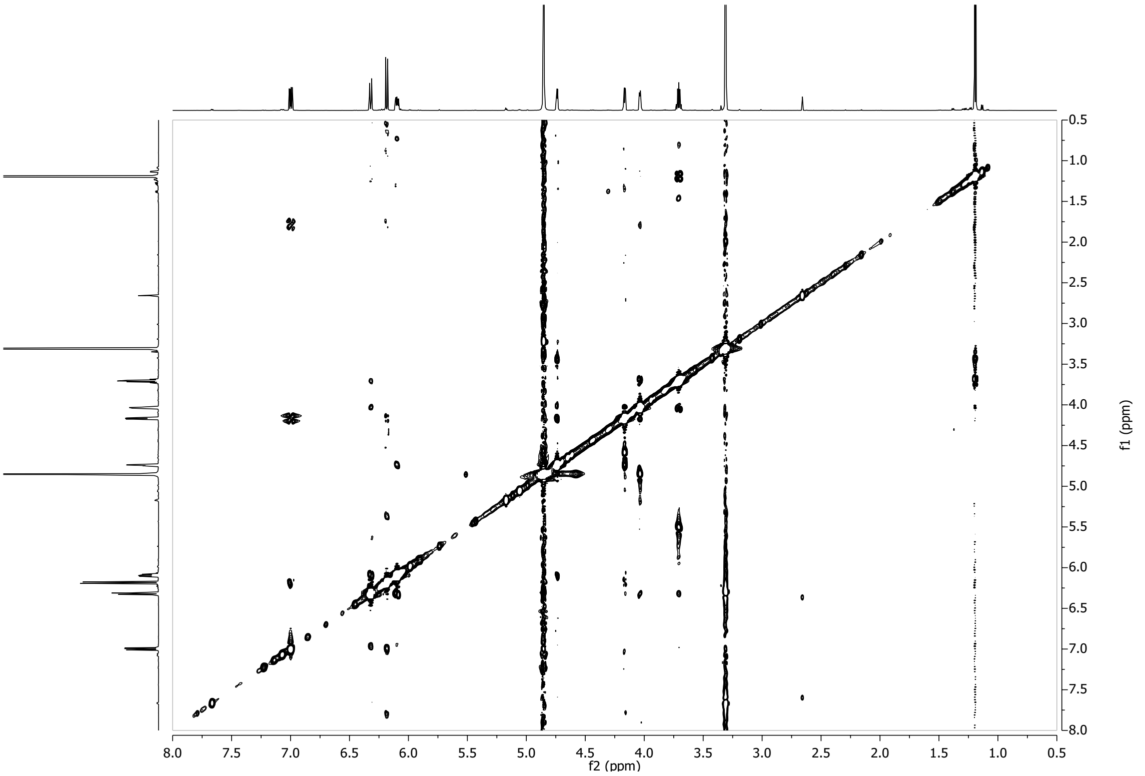

Supplementary Figure 11. ROESY NMR spectrum of compound **1** in CD_3OD at 600 MHz.

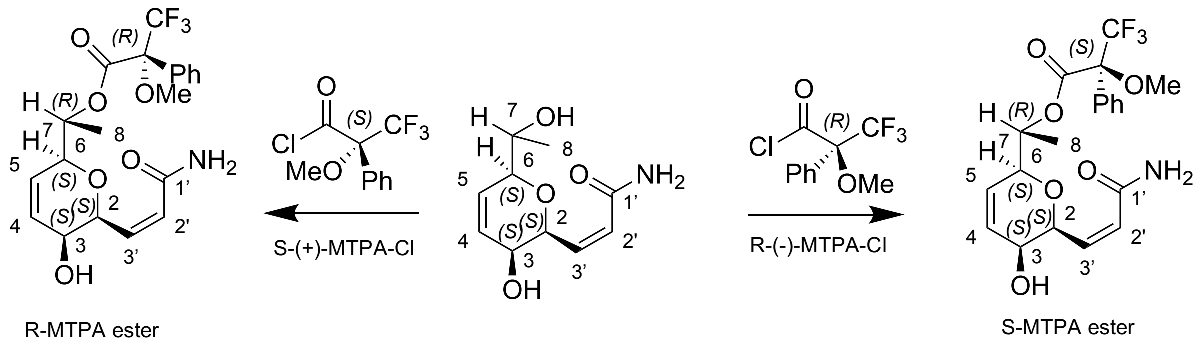

Supplementary Figure 12. Synthesis of the *R*- and *S*- MTPA esters from compound **1**.

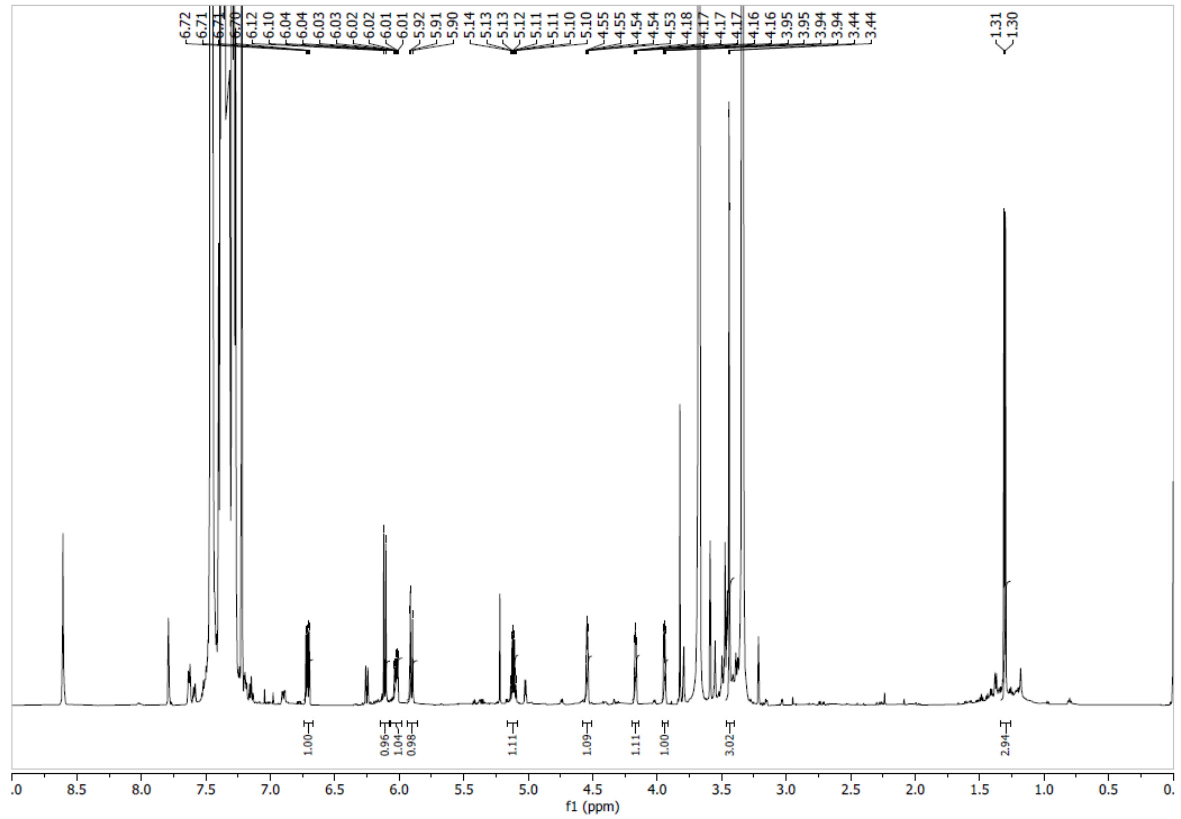

Supplementary Figure 13. ^{1}H NMR spectrum of **1** *R*MTPA in $CDCl_3$ at 600 MHz.

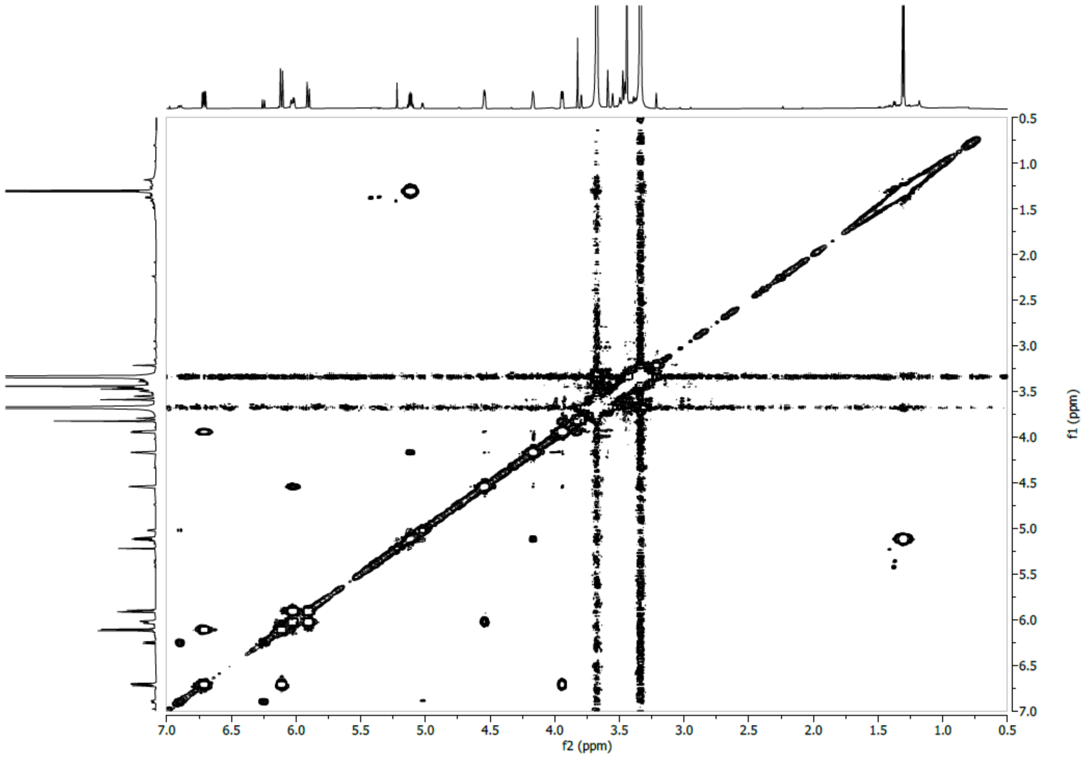

Supplementary Figure 14. COSY NMR spectrum of **1** *R*MTPA in $CDCl_3$ at 600 MHz.

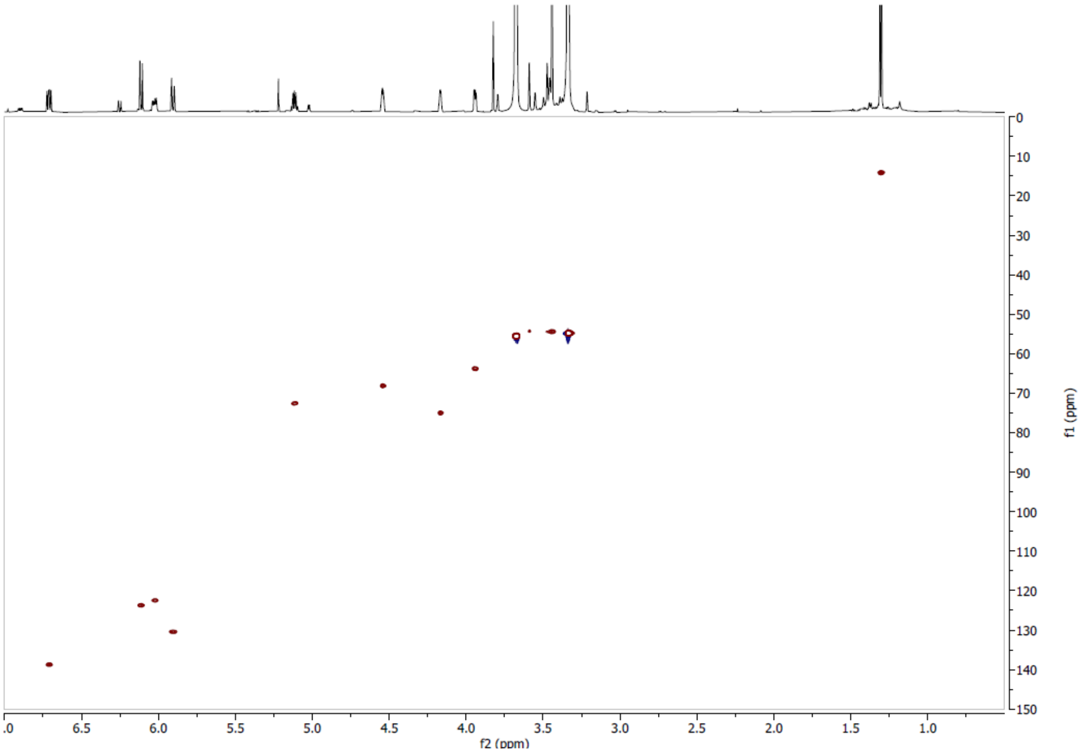

Supplementary Figure 15. Edited HSQC NMR spectrum of **1** *R*MTPA in $CDCl_3$ at 600 MHz.

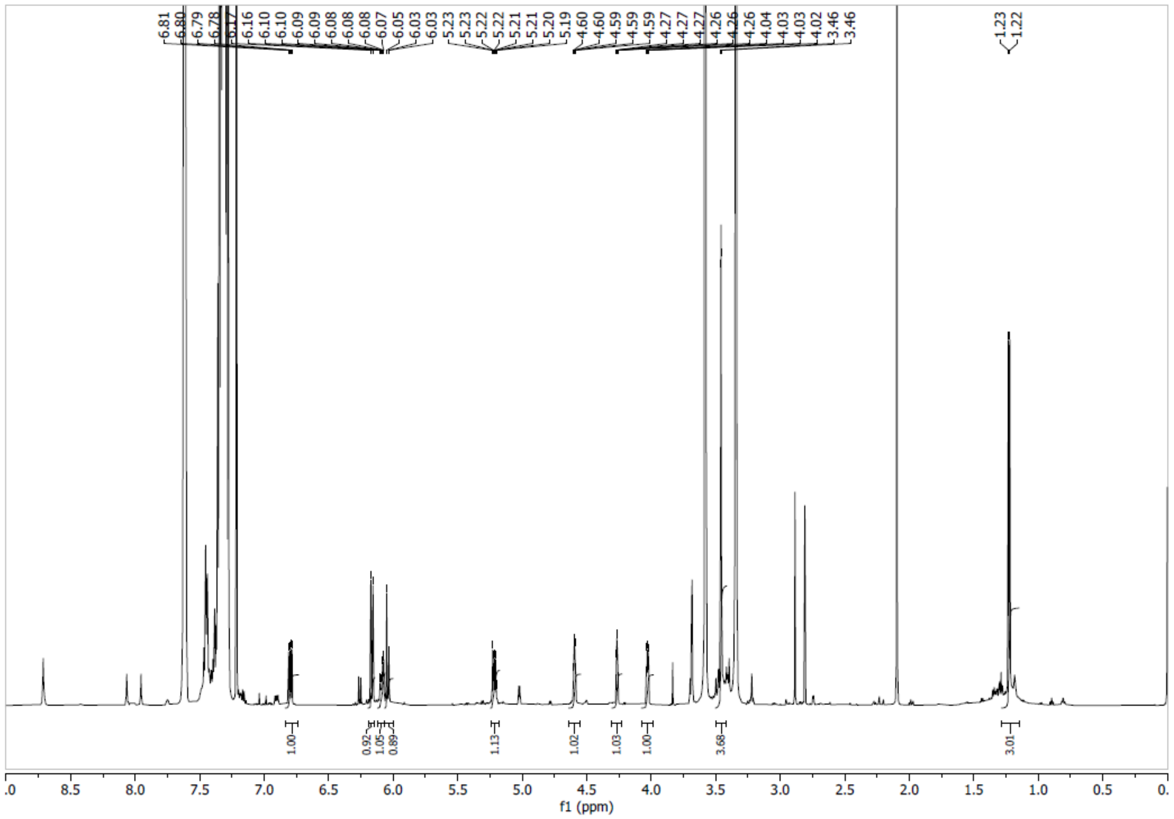

Supplementary Figure 16. ^{1}H NMR spectrum of **1** *S*MTPA in $CDCl_3$ at 600 MHz.

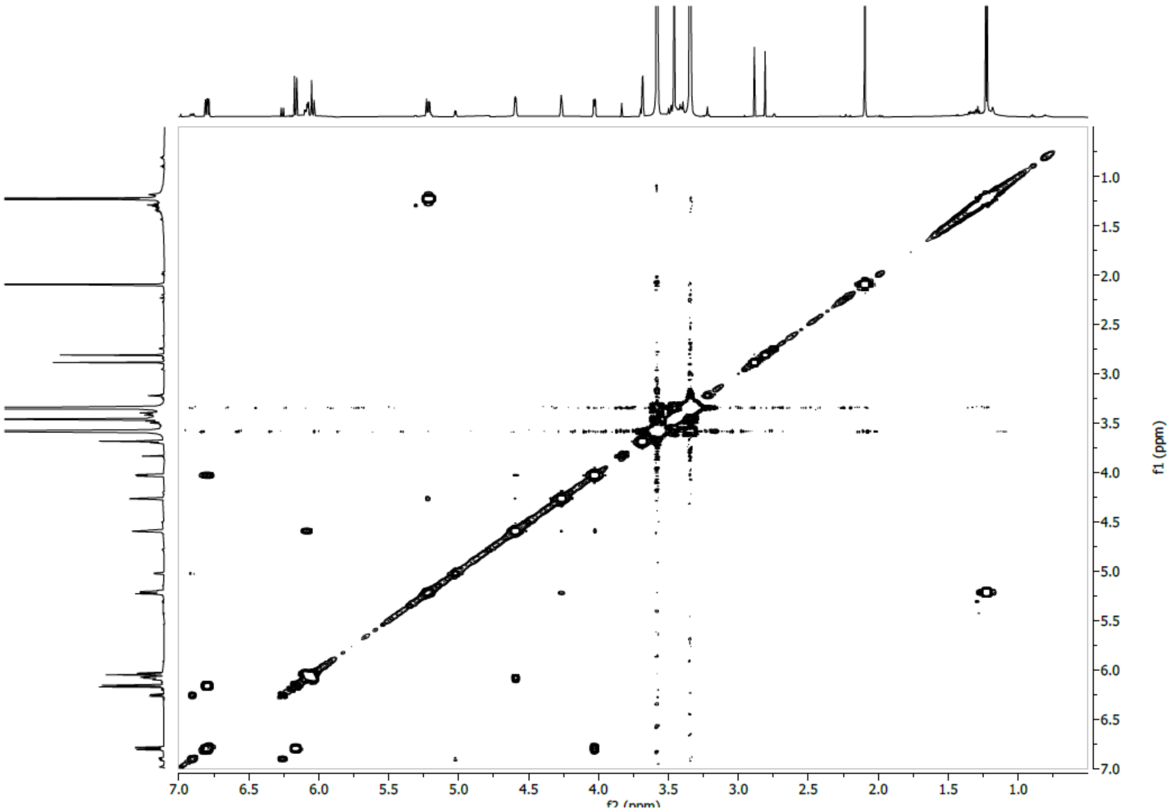

Supplementary Figure 17. COSY NMR spectrum of **1** *S*MTPA in $CDCl_3$ at 600 MHz.

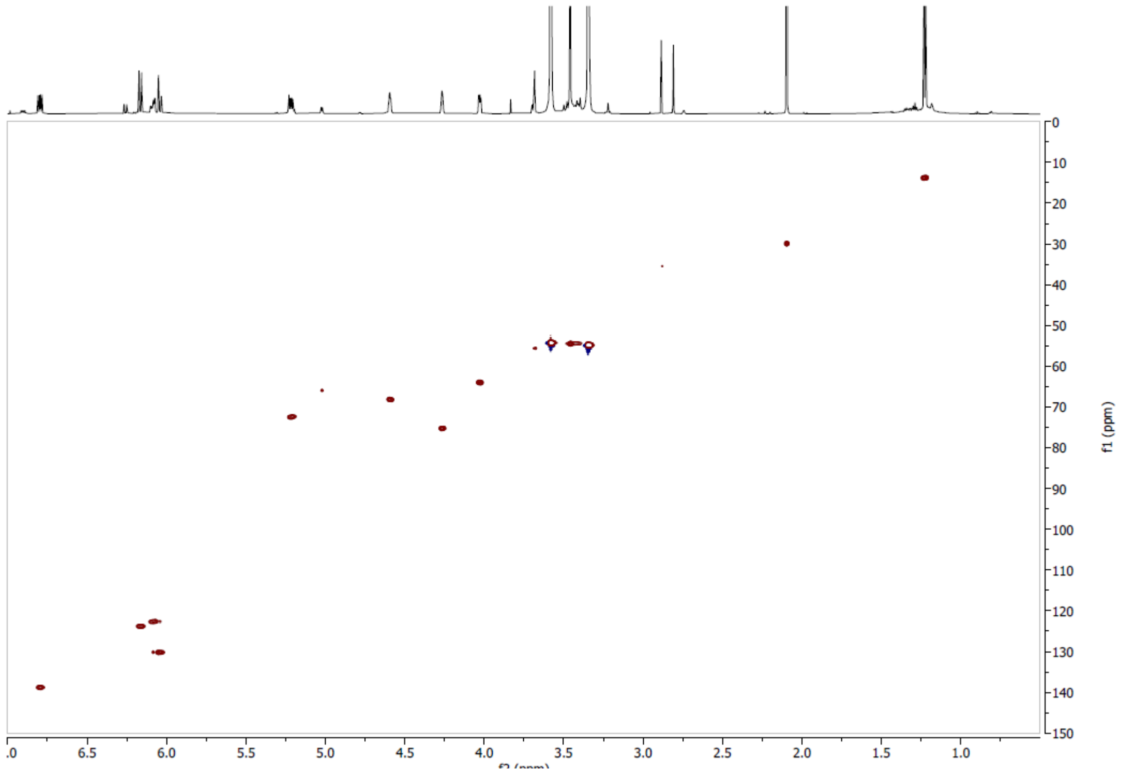

Supplementary Figure 18. Edited HSQC NMR spectrum of **1** *S*MTPA in $CDCl_3$ at 600 MHz.

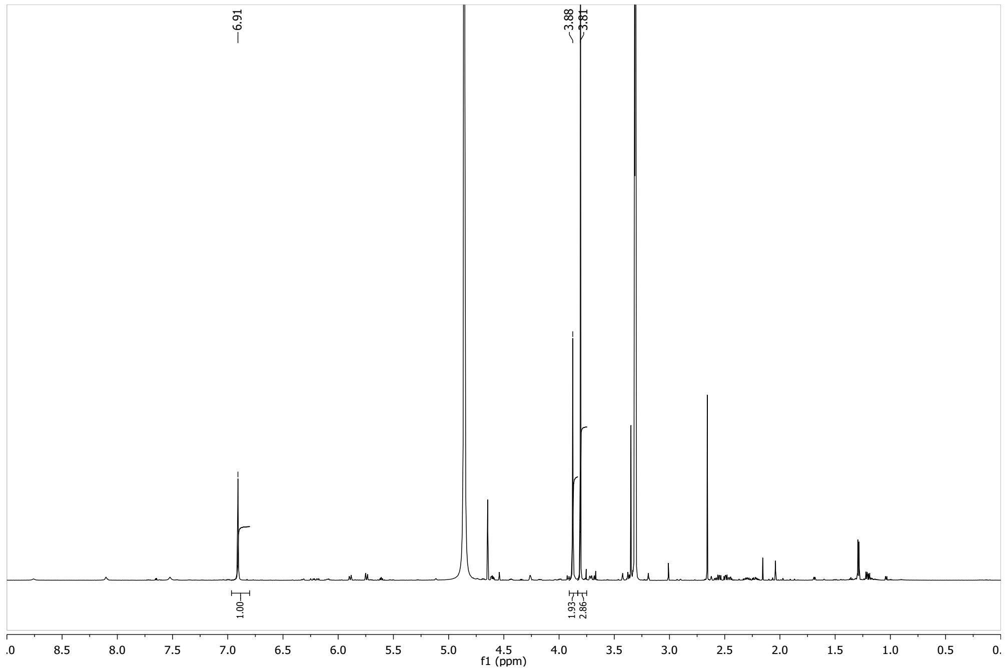

Supplementary Figure 19. ^{1}H NMR spectrum of **2** in MeOD at 600 MHz.

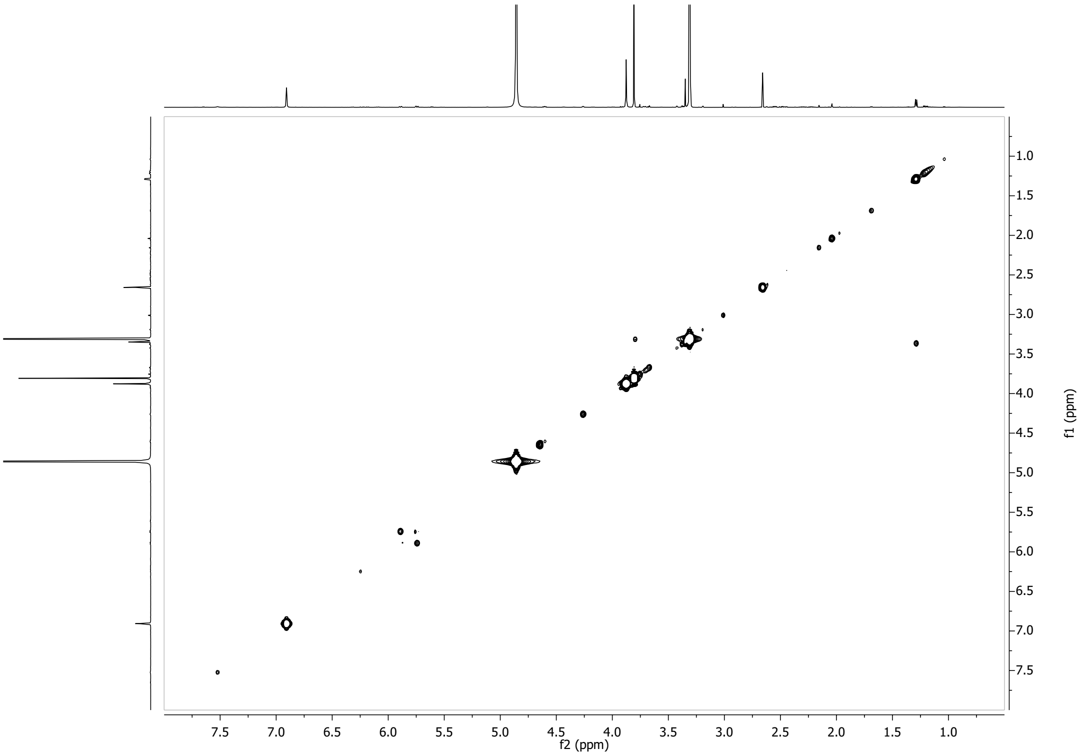

Supplementary Figure 20. COSY NMR spectrum of **2** in MeOD at 600 MHz.

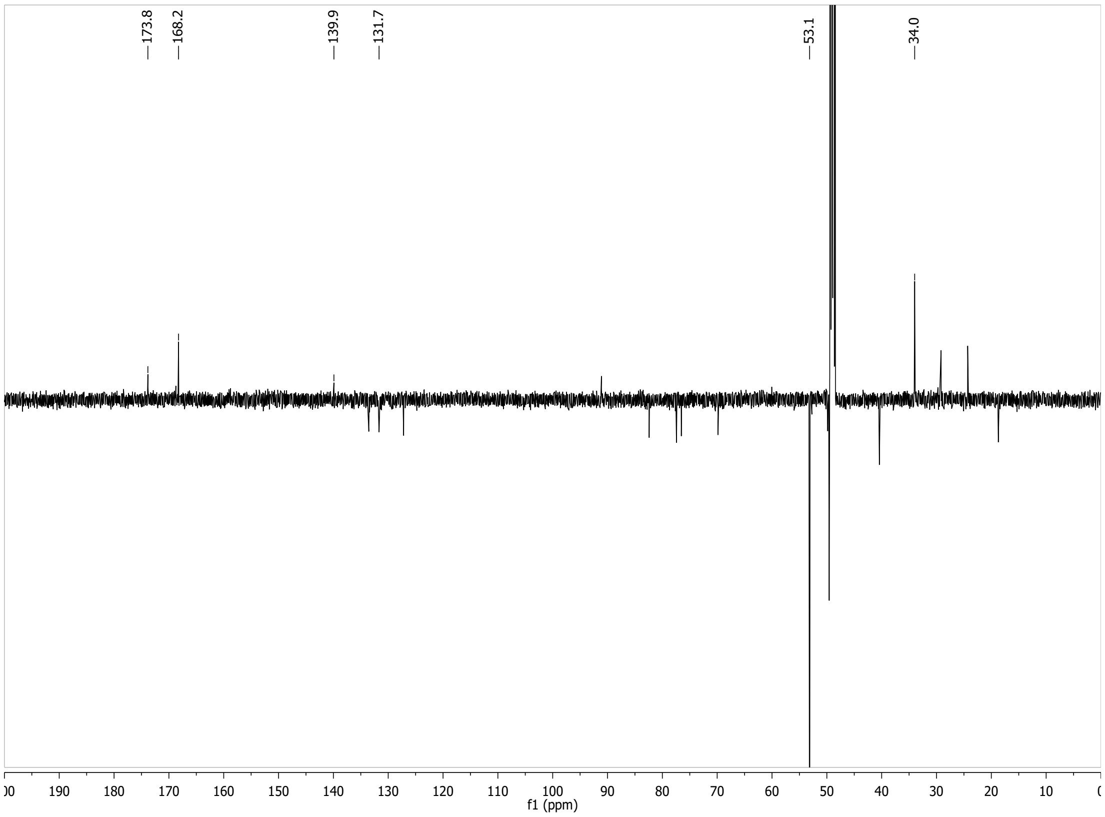

Supplementary Figure 21. ^{1}C NMR spectrum of **2** in MeOD at 600 MHz.

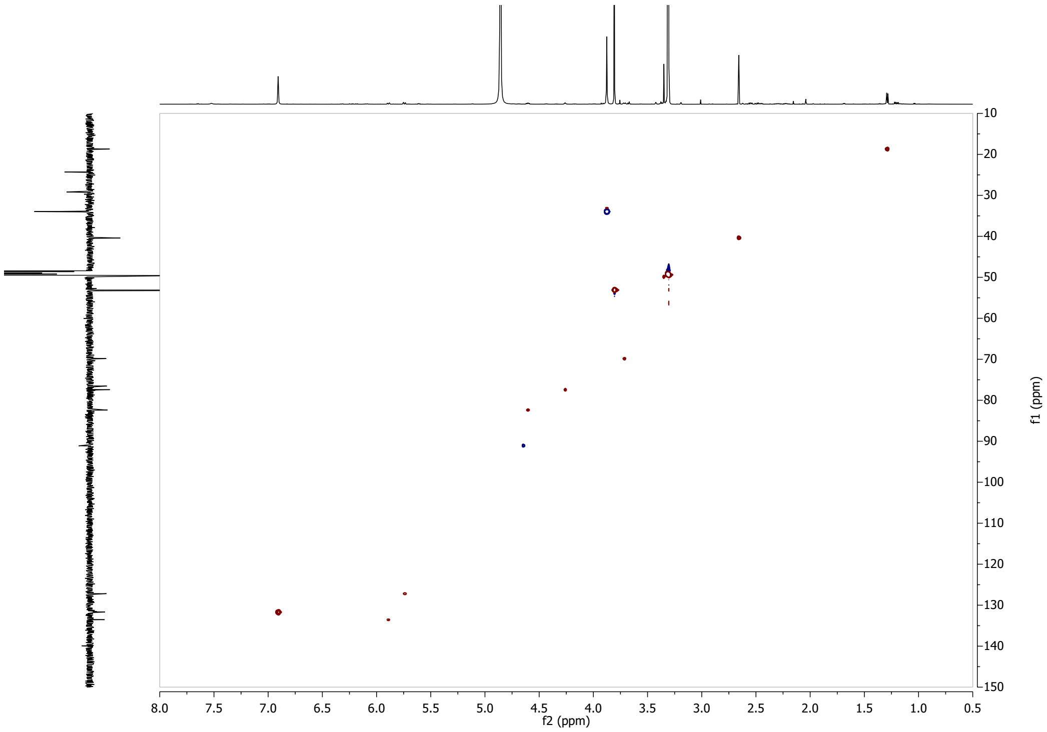

Supplementary Figure 22. HSQC NMR spectrum of **2** in MeOD at 600 MHz.

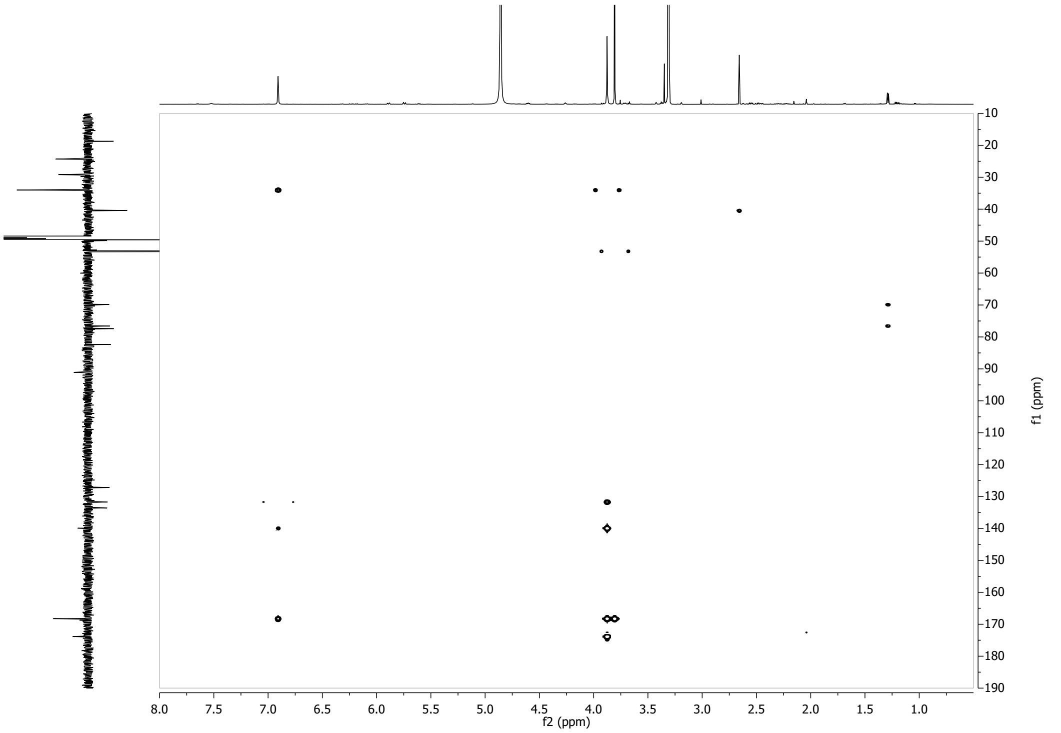

Supplementary Figure 23. HMBC NMR spectrum of **2** in MeOD at 600 MHz.

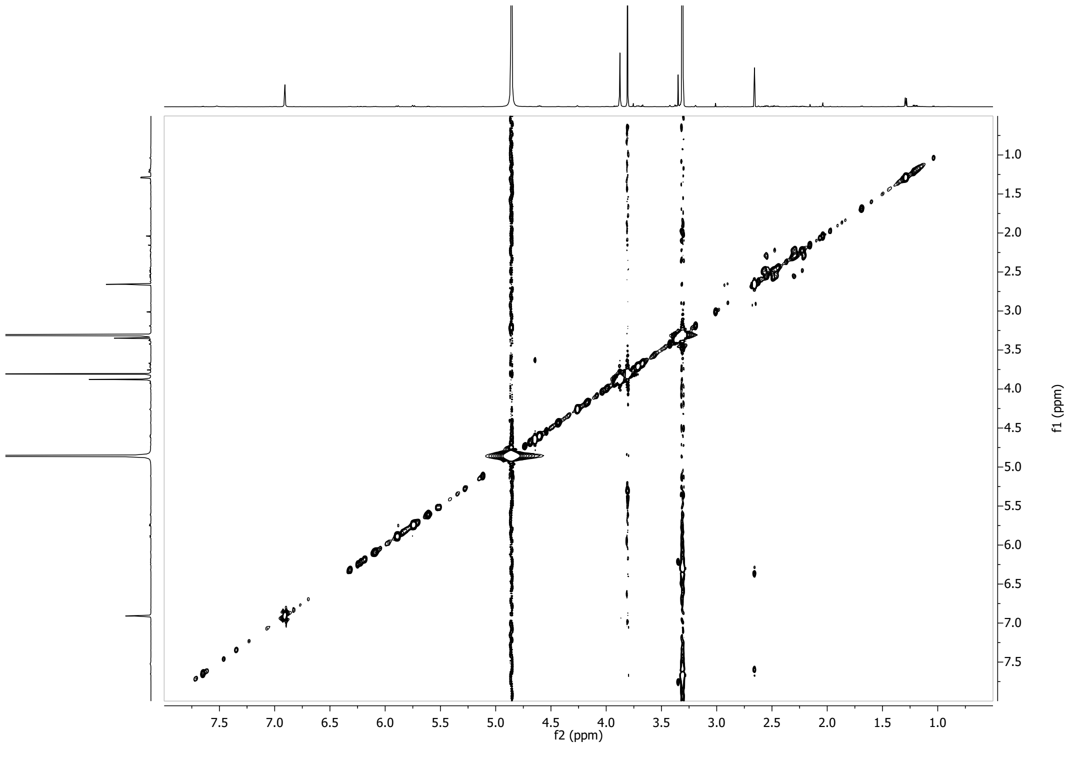

Supplementary Figure 24. ROESY NMR spectrum of **2** in MeOD at 600 MHz.

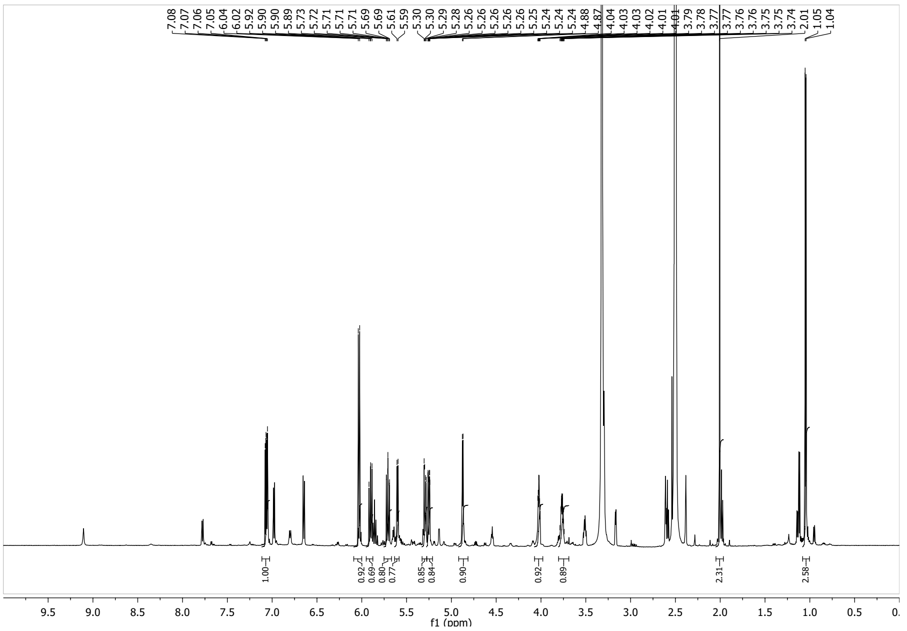

Supplementary Figure 25. ^{1}H NMR spectrum of **3** in DMSO-d_6 at 600 MHz.

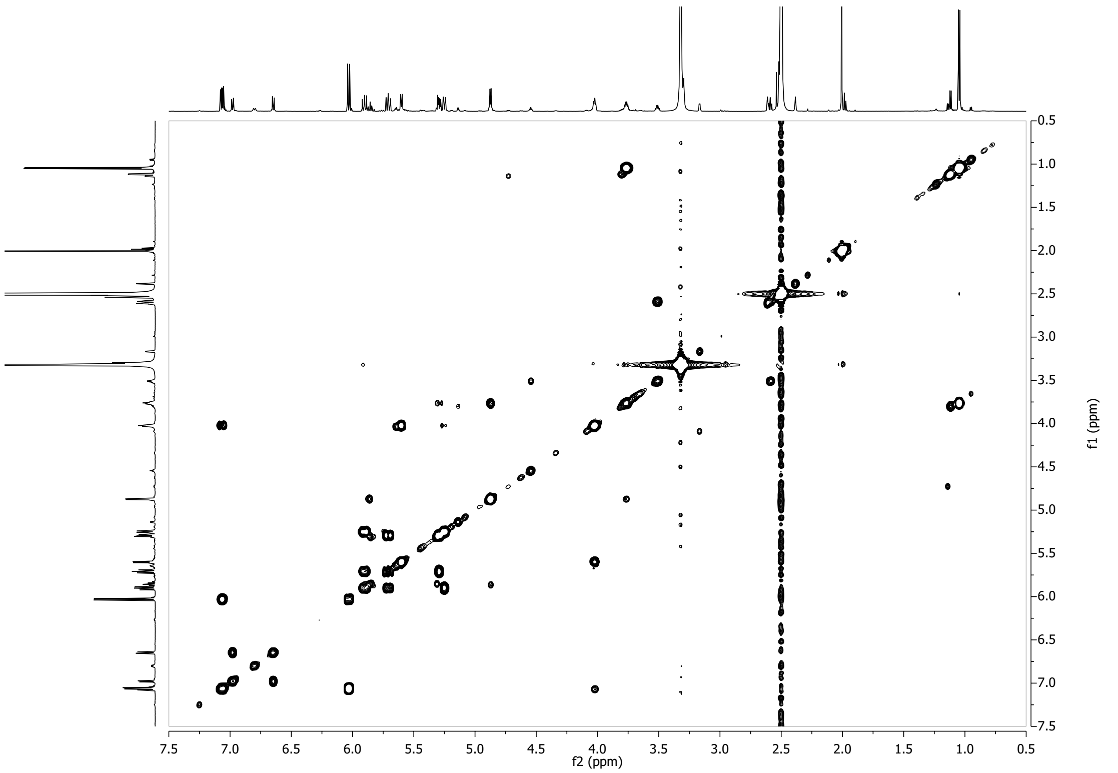

Supplementary Figure 26. COSY NMR spectrum of **3** in DMSO-d_6 at 600 MHz.

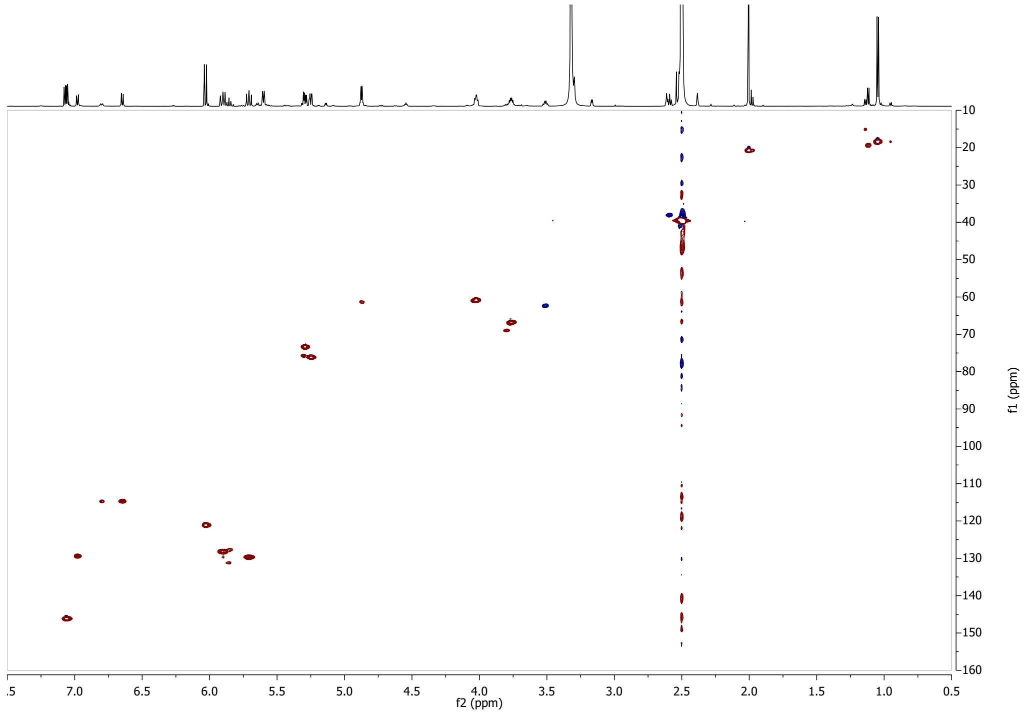

Supplementary Figure 27. HSQC NMR spectrum of **3** in DMSO-d_6 at 600 MHz.

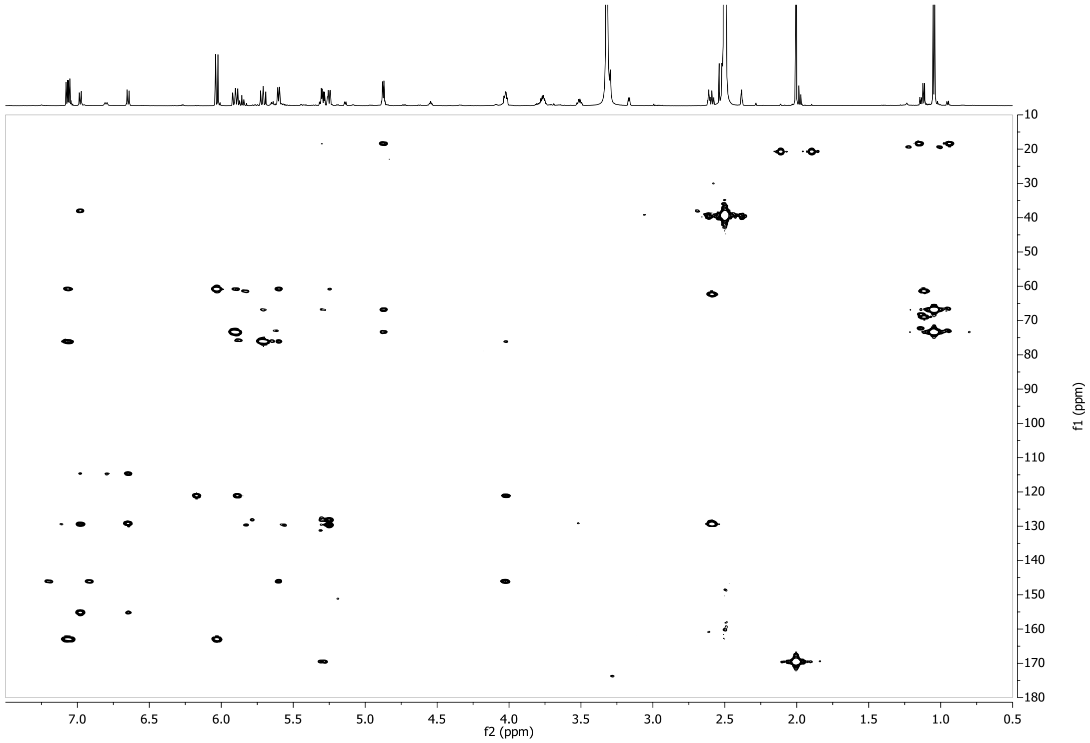

Supplementary Figure 28. HMBC NMR spectrum of **3** in DMSO-d_6 at 600 MHz.

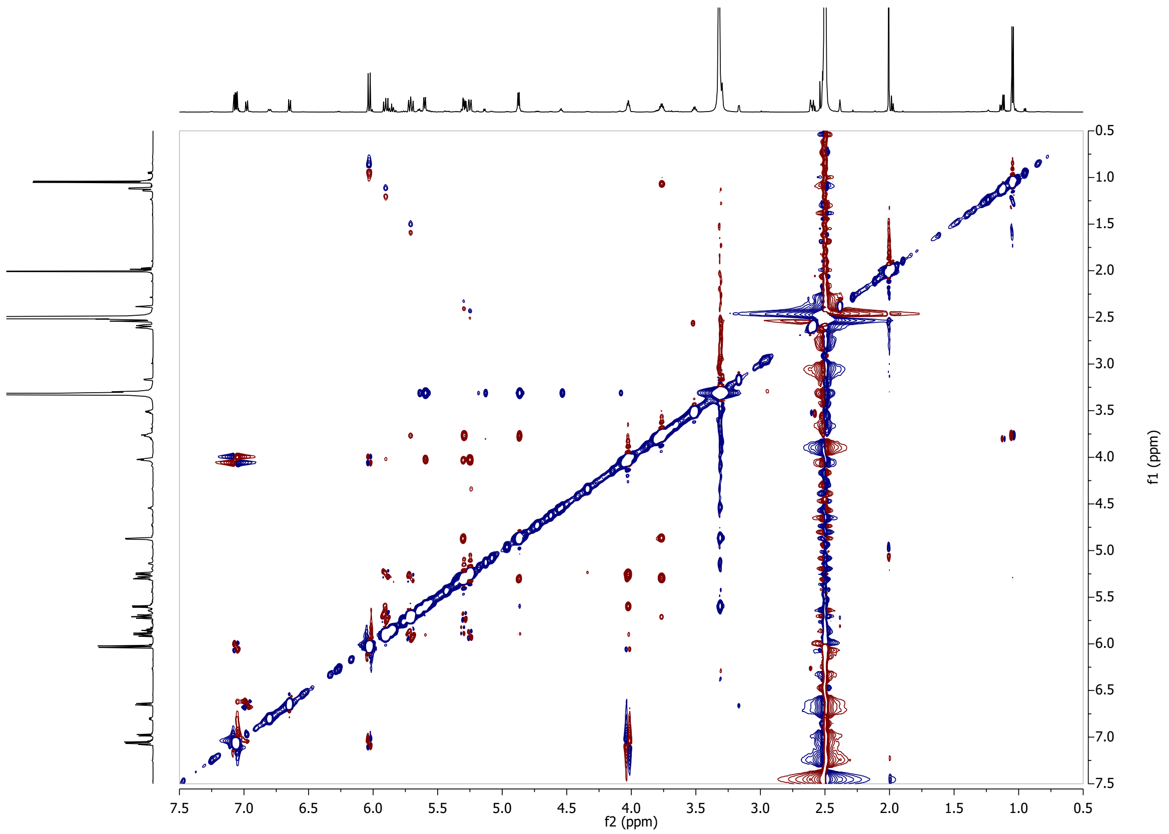

Supplementary Figure 29. ROESY NMR spectrum of **3** in DMSO-d_6 at 600 MHz.

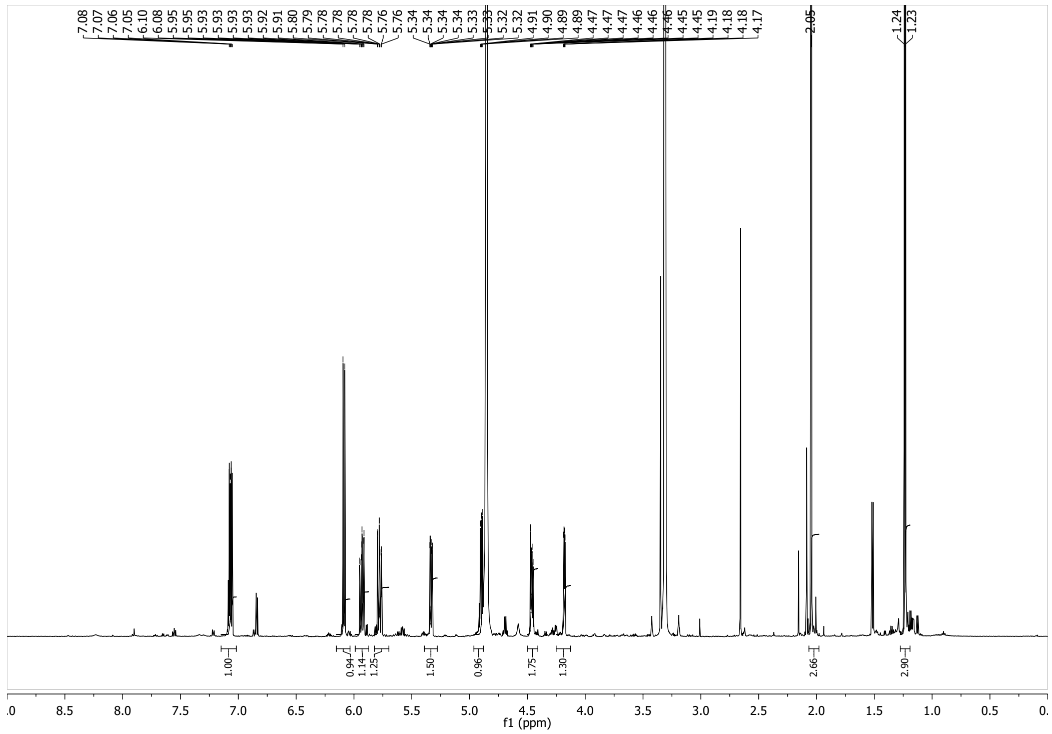

Supplementary Figure 30. ^{1}H NMR spectrum of **4** in MeOD at 600 MHz.

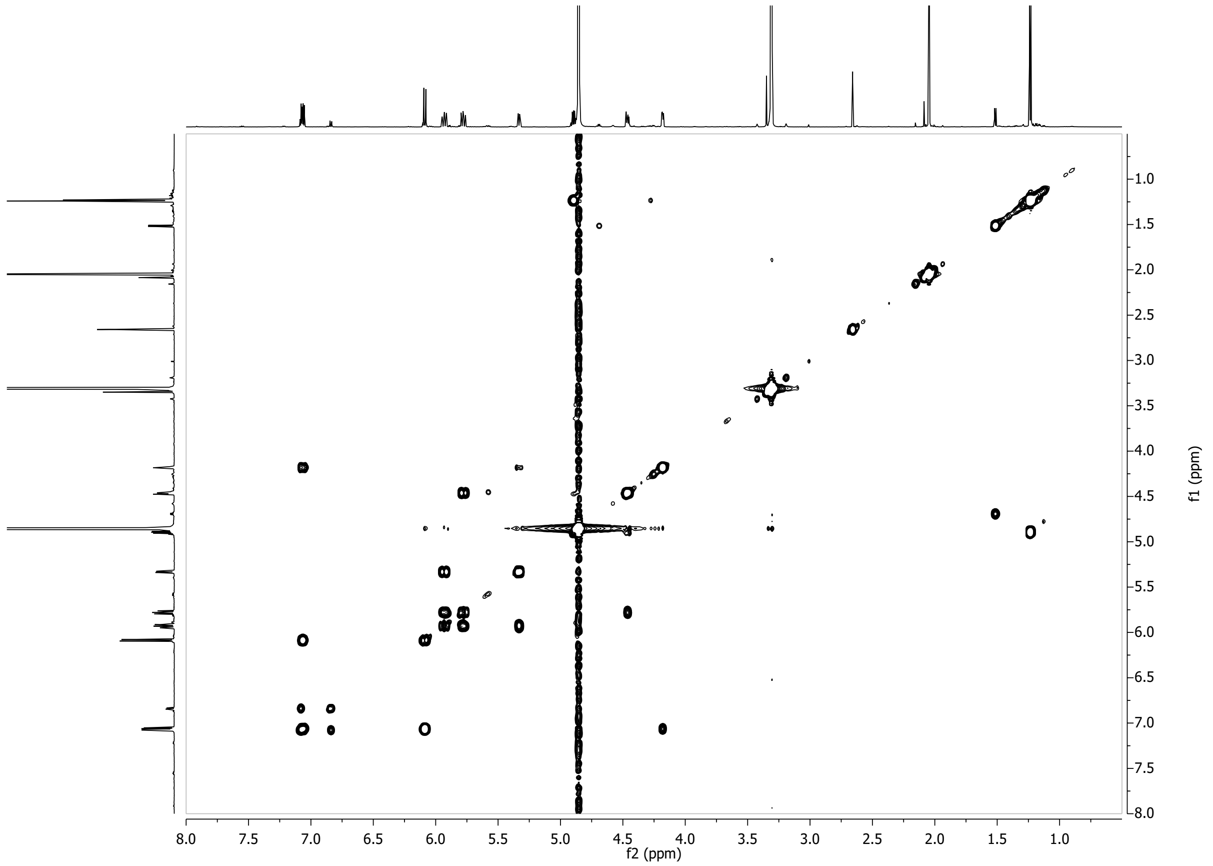

Supplementary Figure 31. COSY NMR spectrum of **4** in MeOD at 600 MHz.

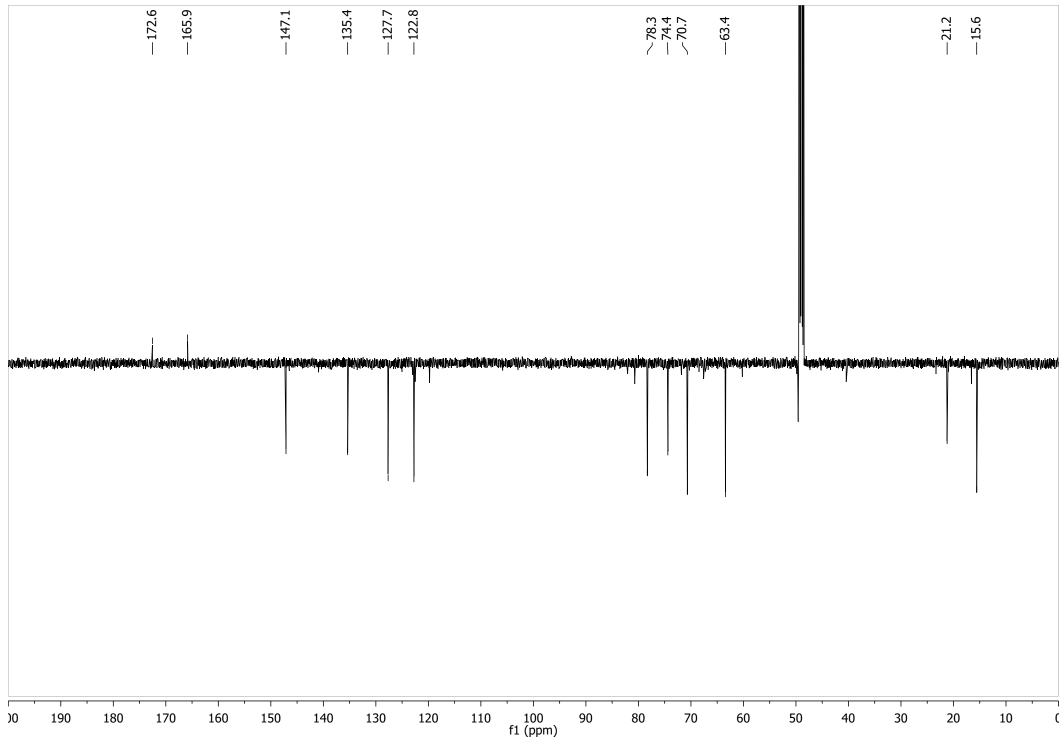

Supplementary Figure 32. [1]C NMR spectrum of **4** in MeOD at 600 MHz.

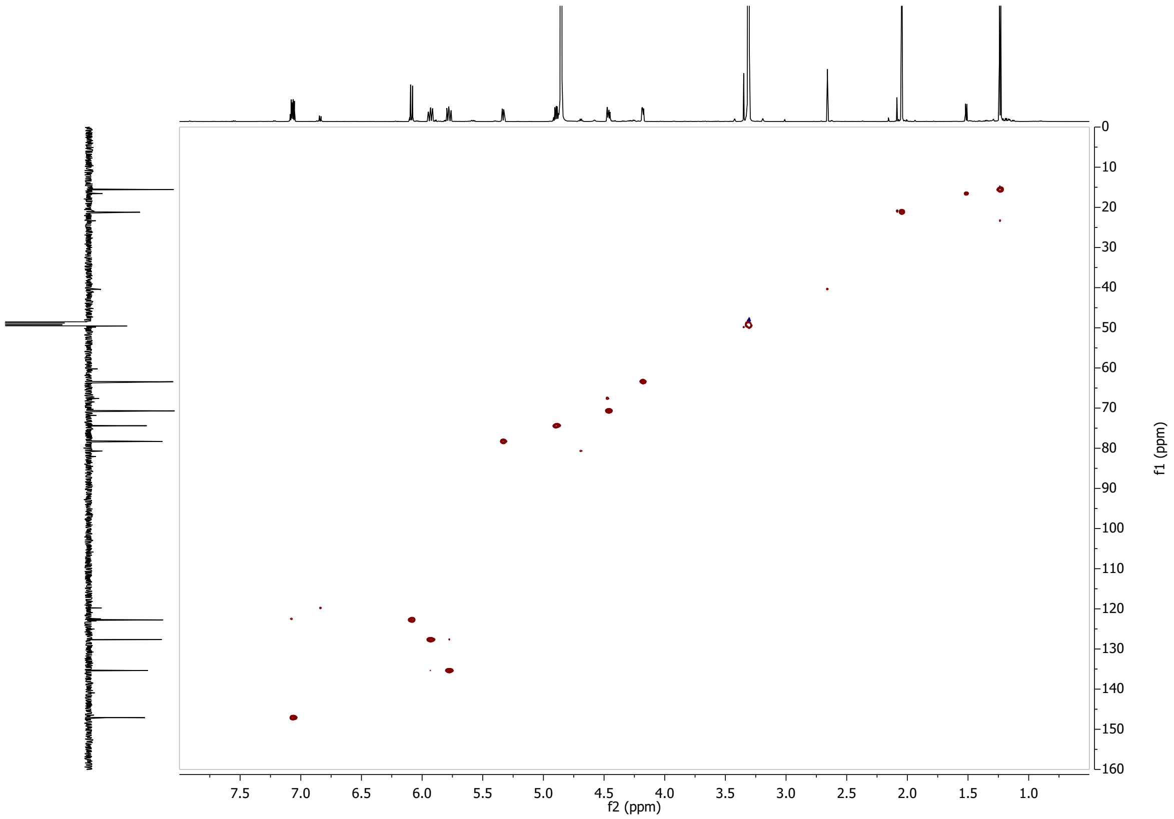

Supplementary Figure 33. HSQC NMR spectrum of **4** in MeOD at 600 MHz.

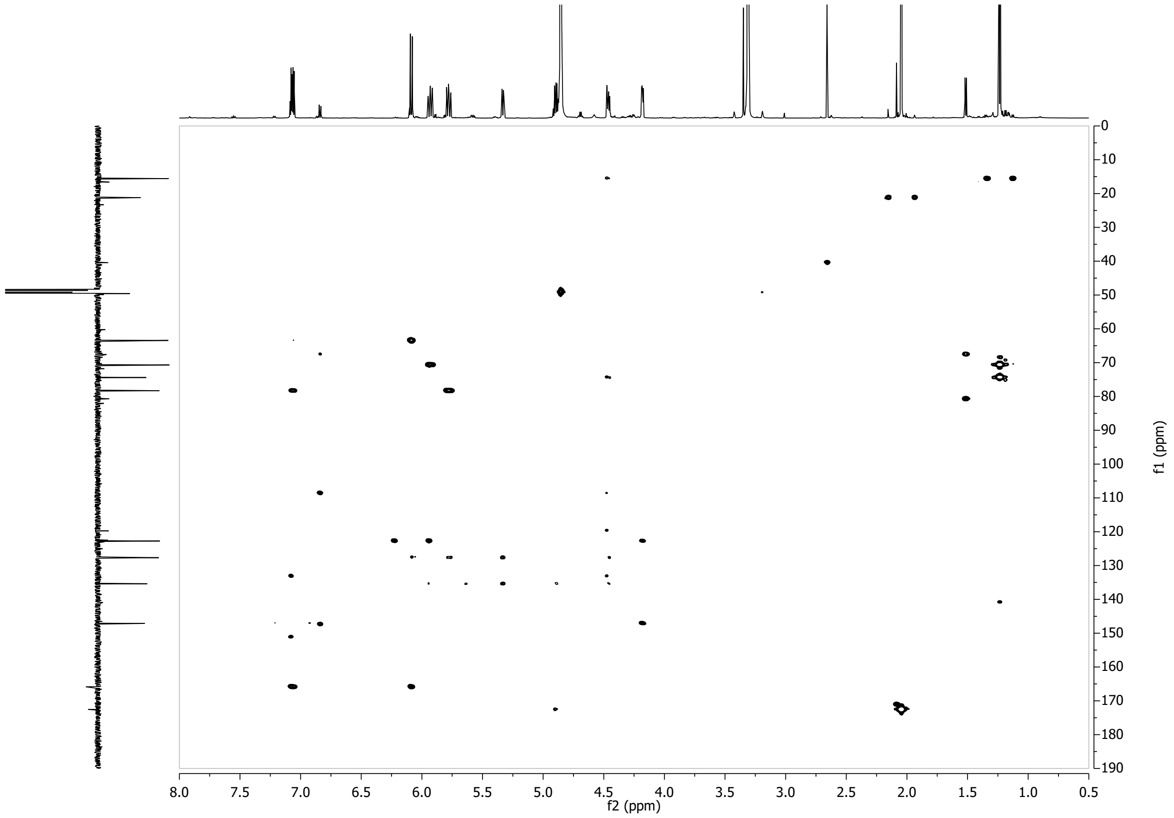

Supplementary Figure 34. HMBC NMR spectrum of **4** in MeOD at 600 MHz.

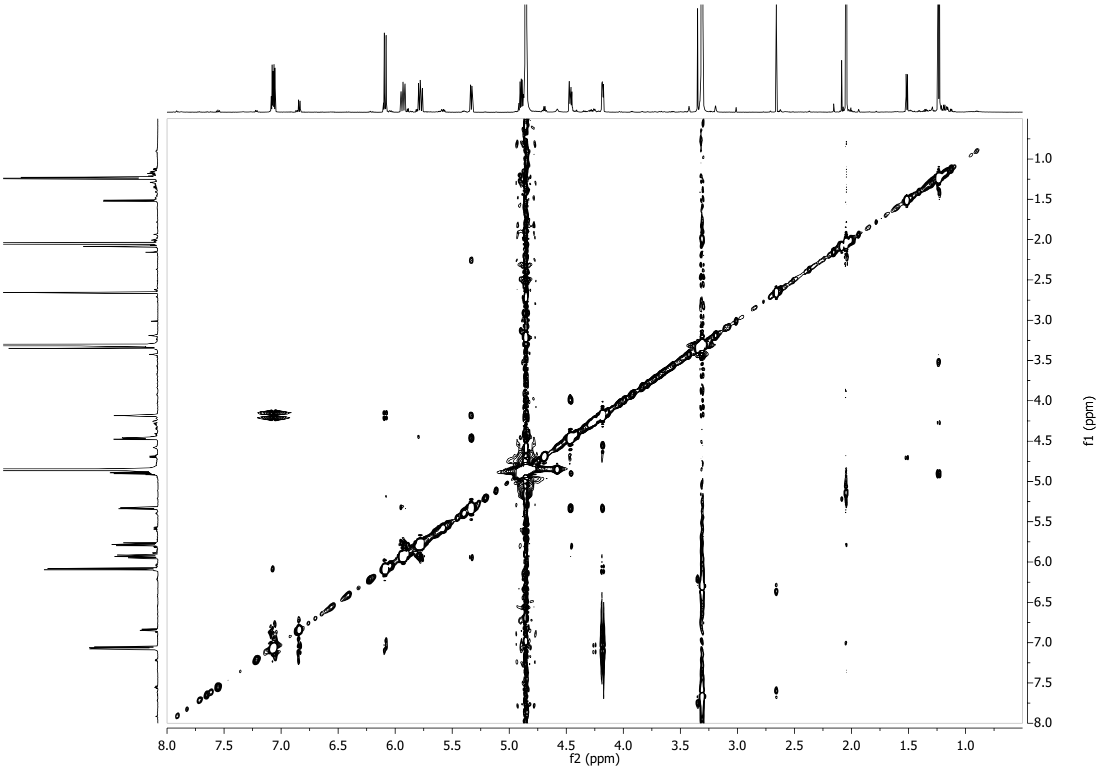

Supplementary Figure 35. ROESY NMR spectrum of **4** in MeOD at 600 MHz.

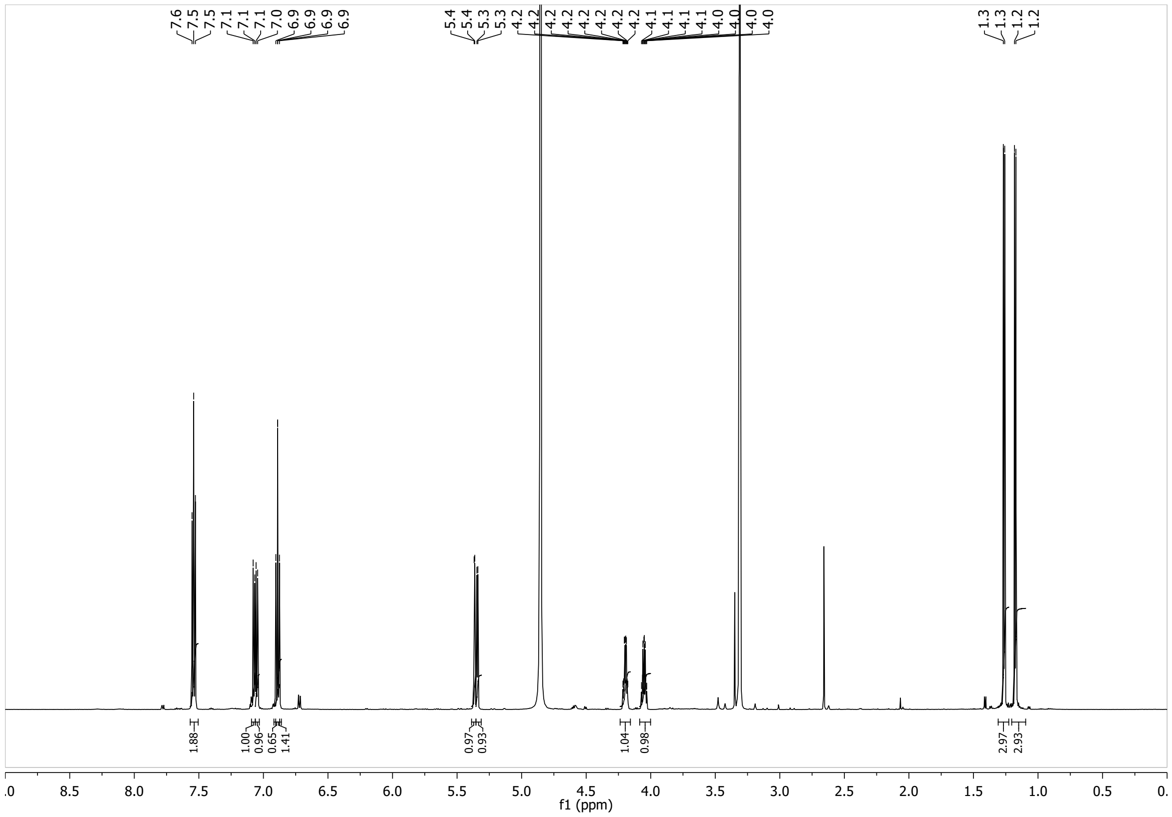

Supplementary Figure 36. ^{1}H NMR spectrum of **5** in MeOD at 600 MHz.

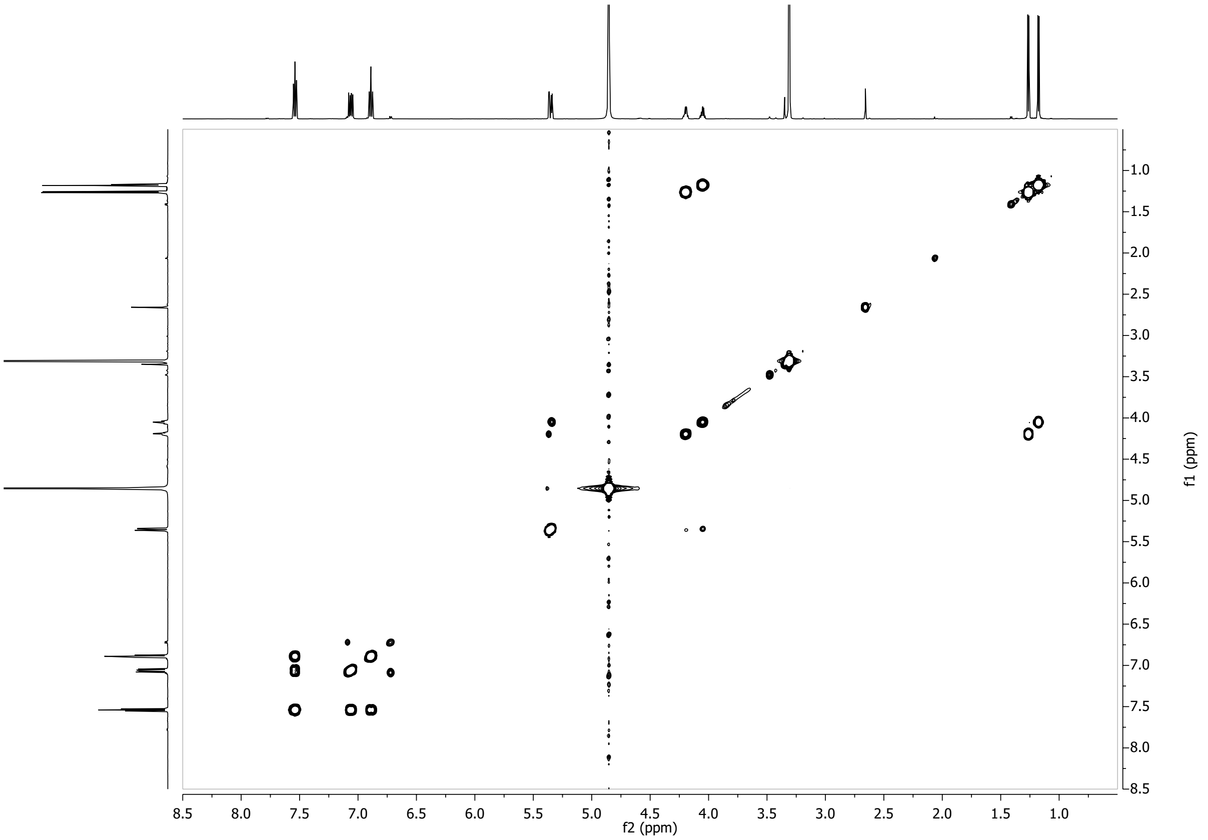

Supplementary Figure 37. COSY NMR spectrum of **5** in MeOD at 600 MHz.

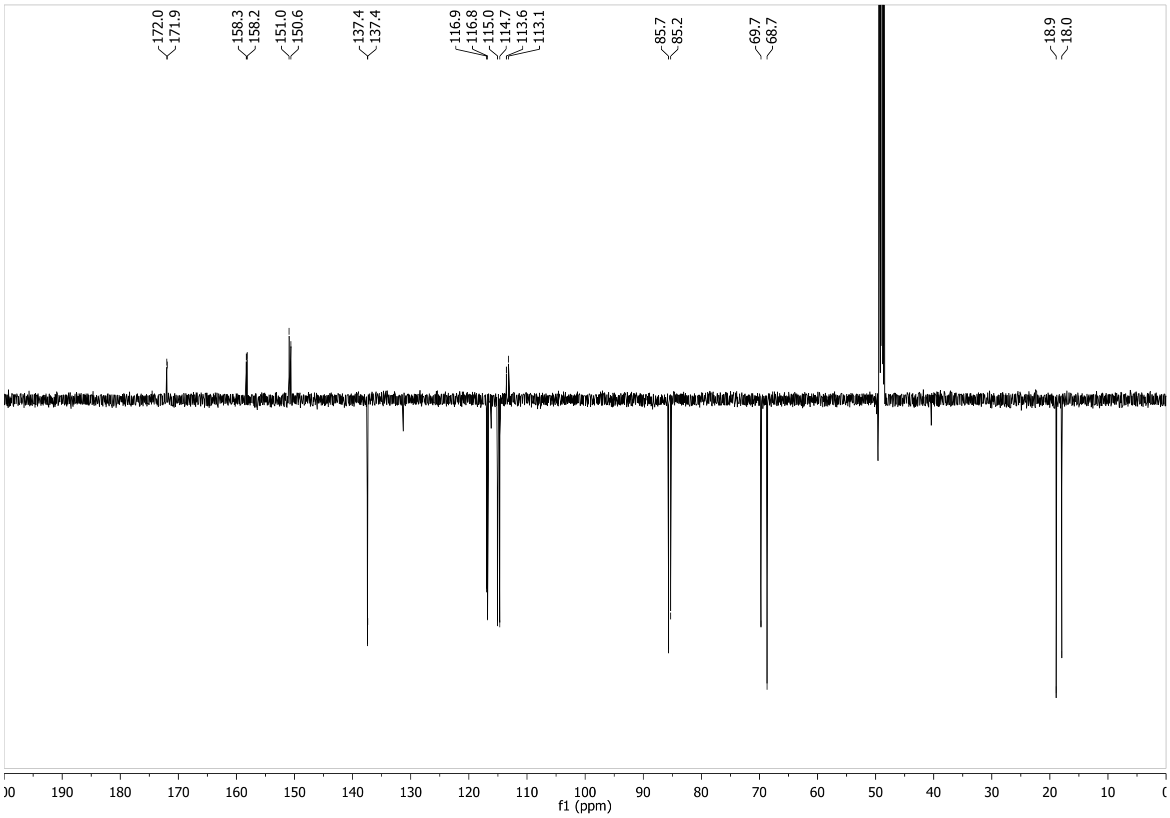

Supplementary Figure 38. [1]C NMR spectrum of **5** in MeOD at 600 MHz.

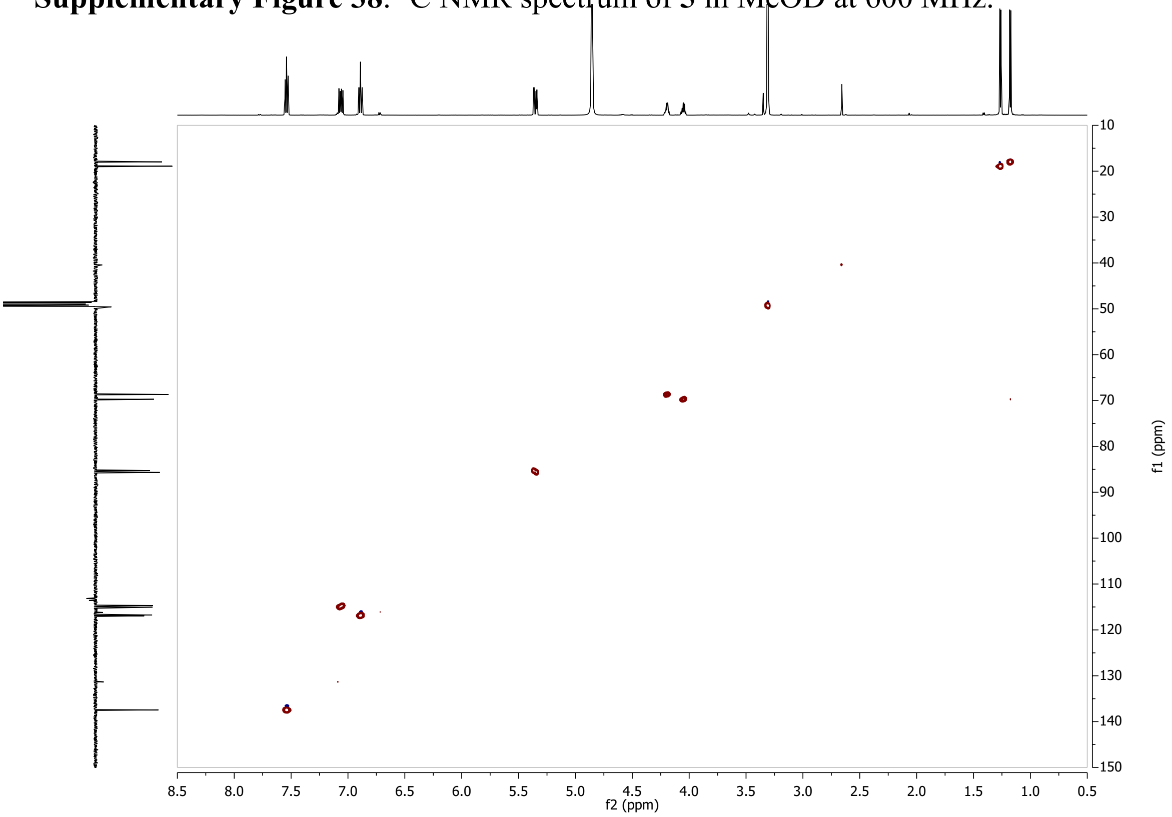

Supplementary Figure 39. HSQC NMR spectrum of **5** in MeOD at 600 MHz.

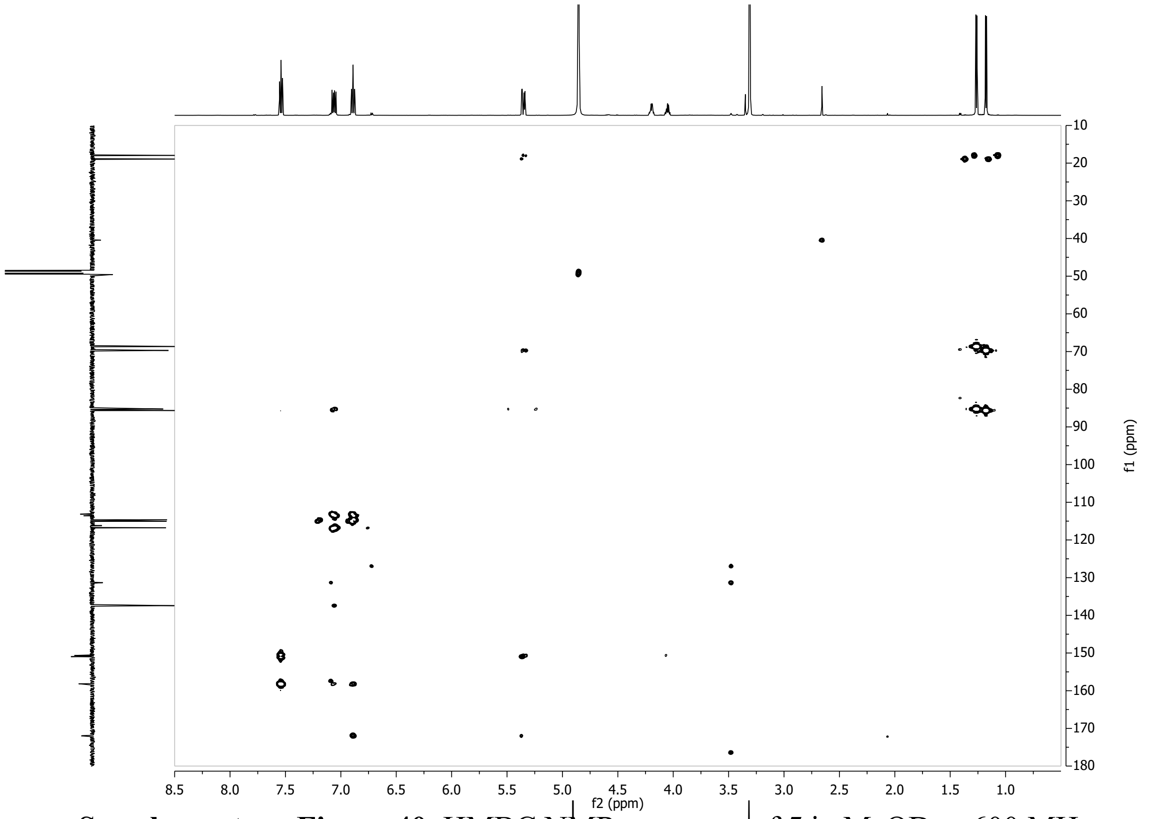

Supplementary Figure 40. HMBC NMR spectrum of **5** in MeOD at 600 MHz.

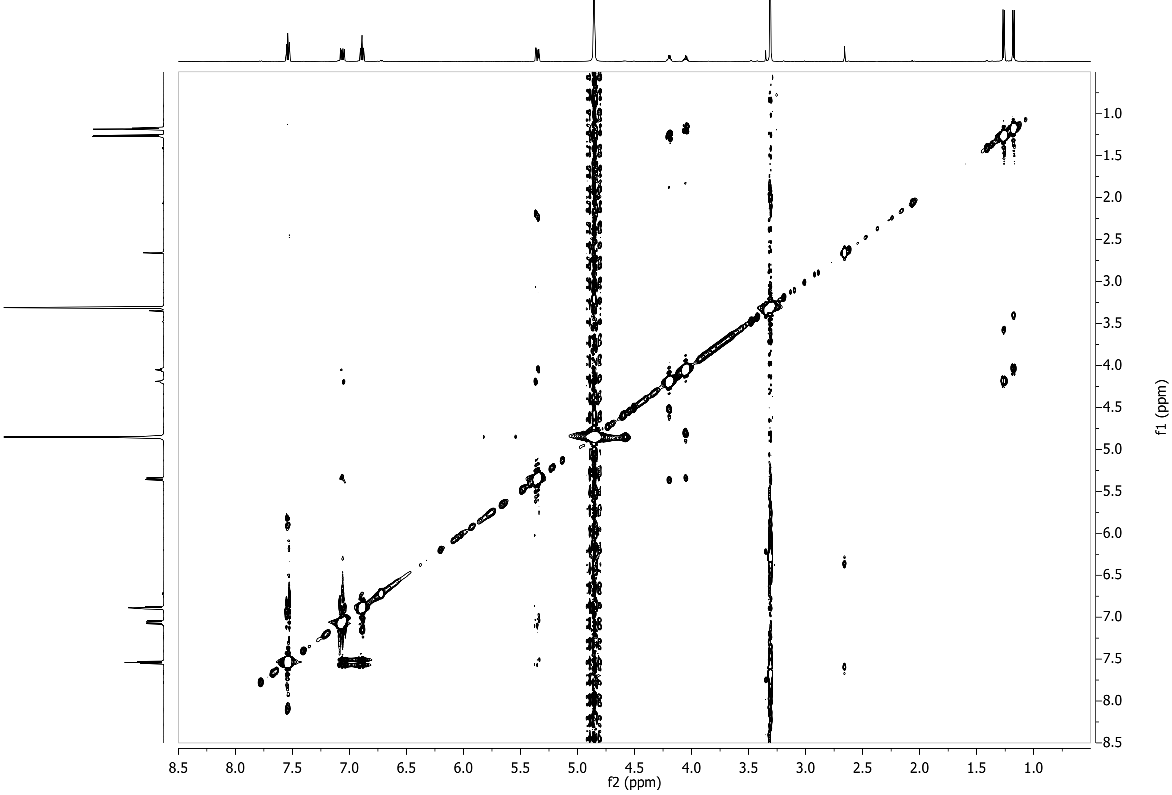

Supplementary Figure 41. ROESY NMR spectrum of **5** in MeOD at 600 MHz.

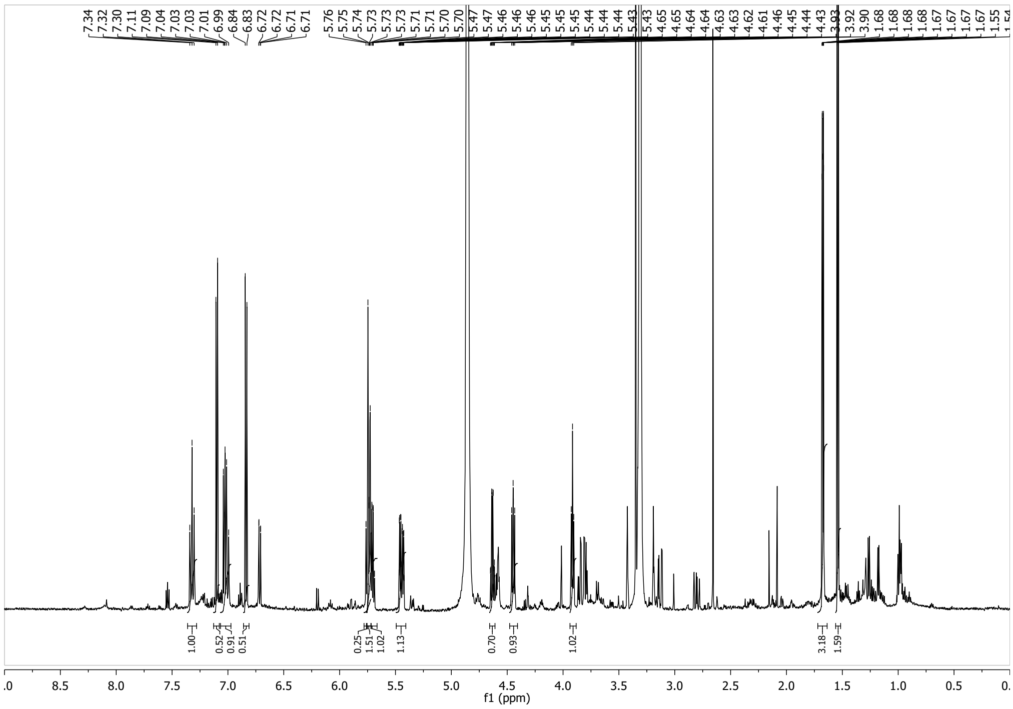

Supplementary Figure 42. ^{1}H NMR spectrum of **6** in MeOD at 600 MHz.

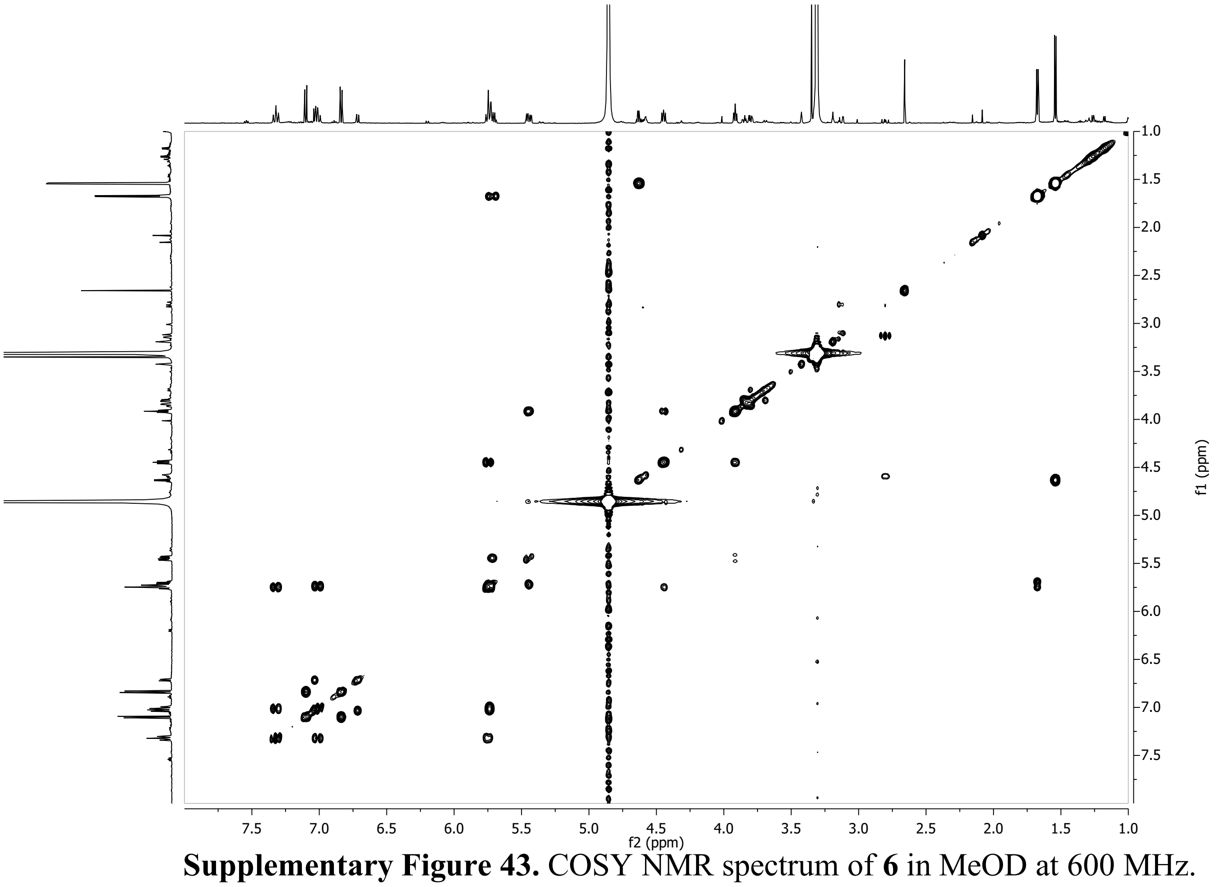

Supplementary Figure 43. COSY NMR spectrum of **6** in MeOD at 600 MHz.

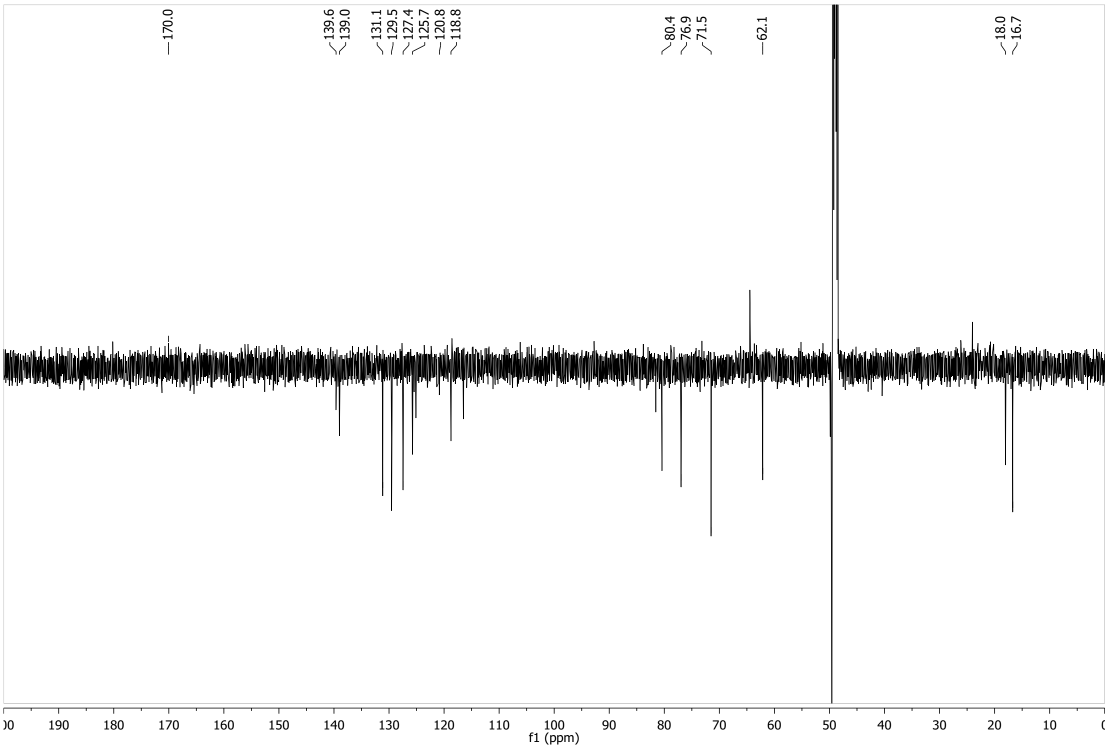

Supplementary Figure 44. ^{1}C NMR spectrum of **6** in MeOD at 600 MHz.

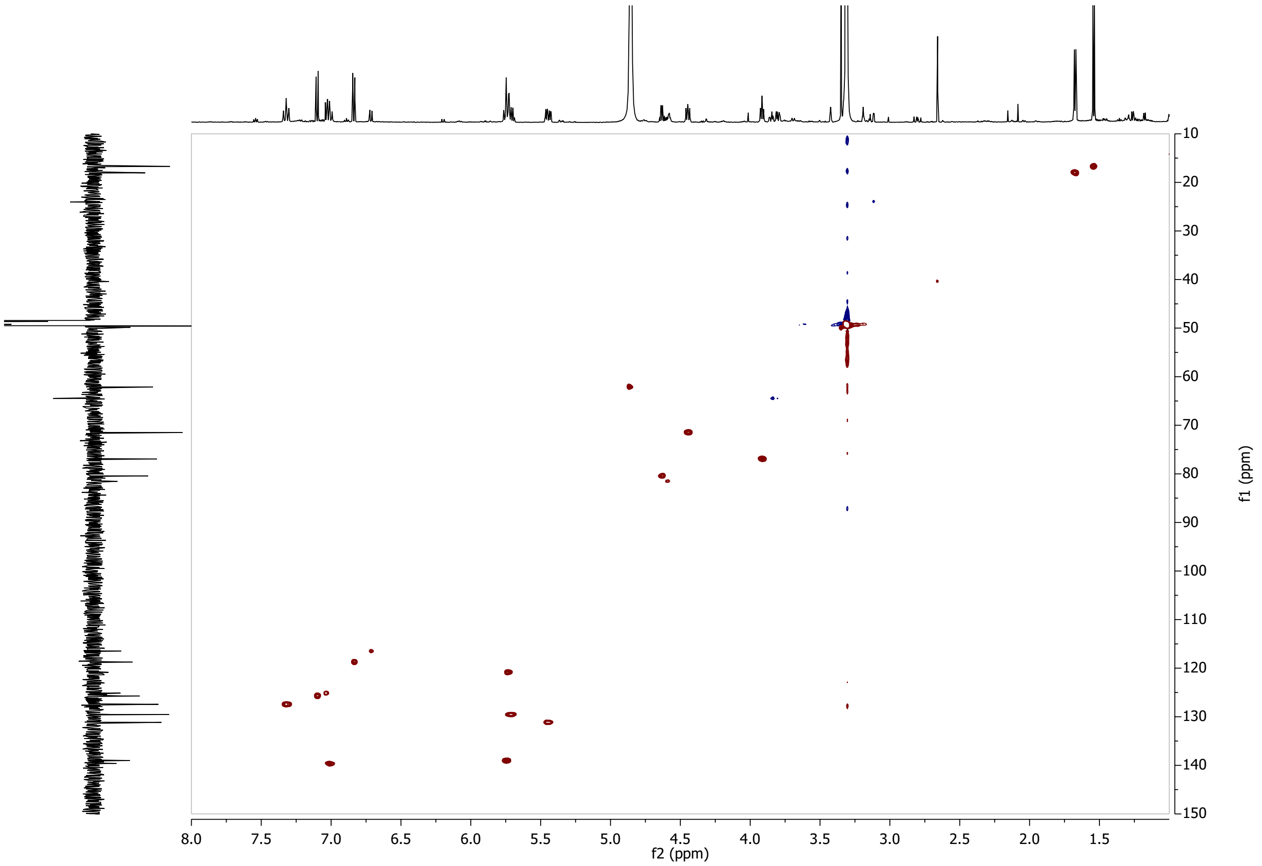

Supplementary Figure 45. HSQC NMR spectrum of **6** in MeOD at 600 MHz.

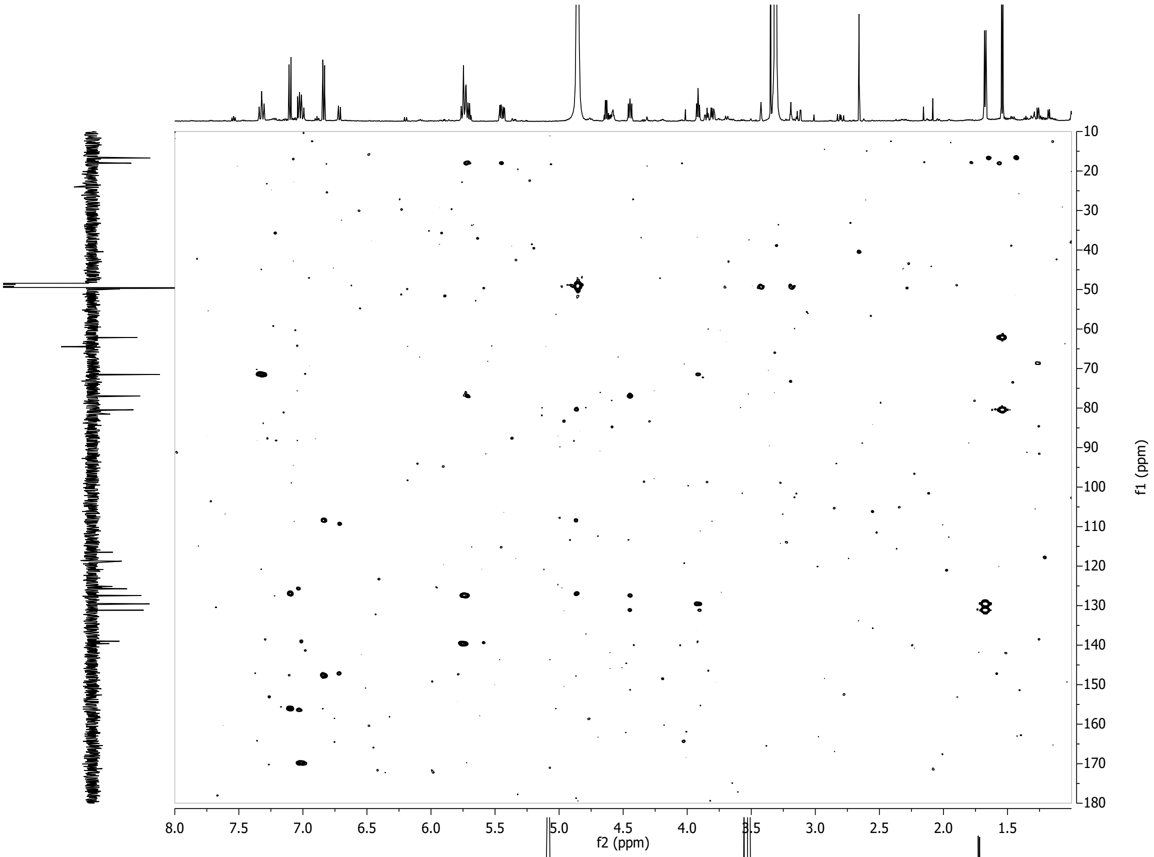

Supplementary Figure 46. HSQC NMR spectrum of **6** in MeOD at 600 MHz.

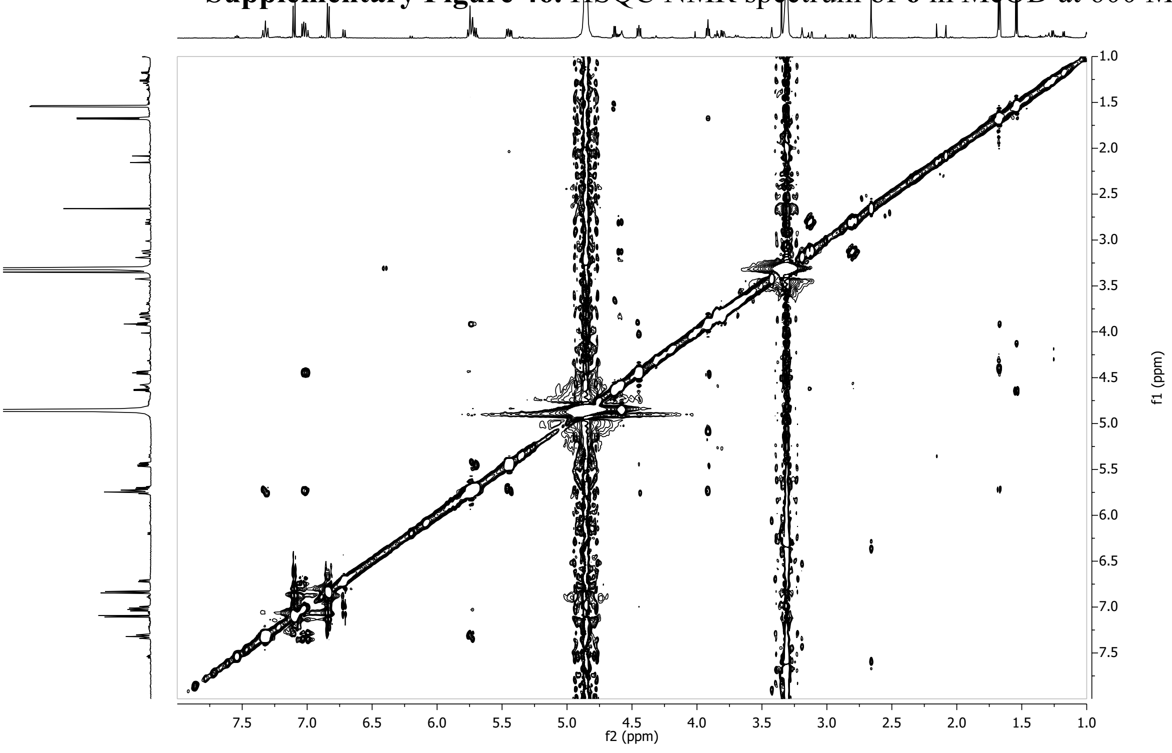

Supplementary Figure 47. ROESY NMR spectrum of **6** in MeOD at 600 MHz.

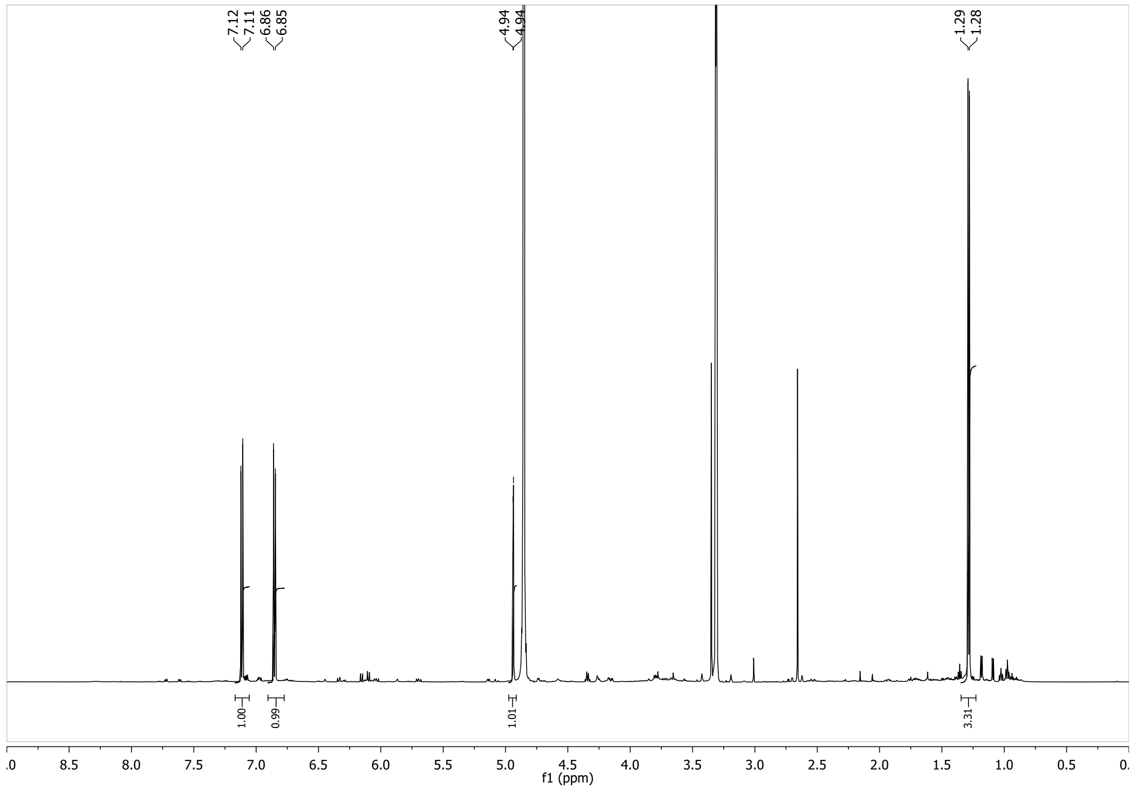

Supplementary Figure 48. ^{1}H NMR spectrum of **7** in MeOD at 600 MHz.

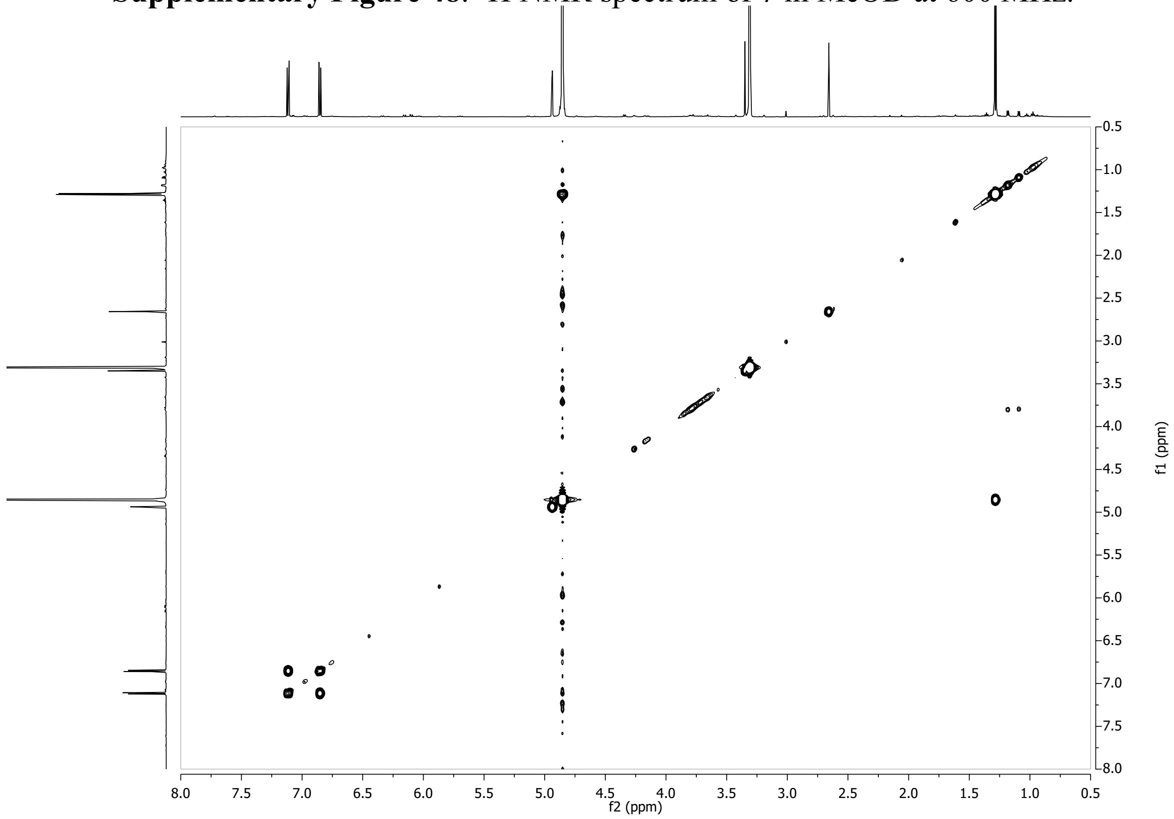

Supplementary Figure 49. COSY NMR spectrum of **7** in MeOD at 600 MHz.

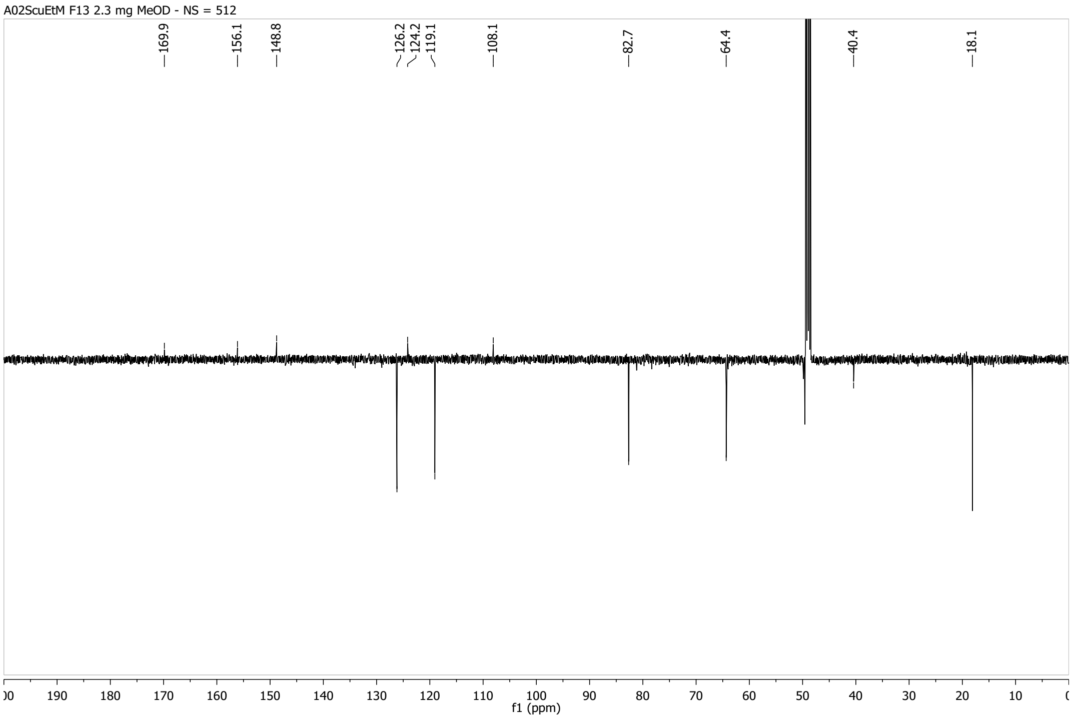

Supplementary Figure 50. [1]C NMR spectrum of **7** in MeOD at 600 MHz.

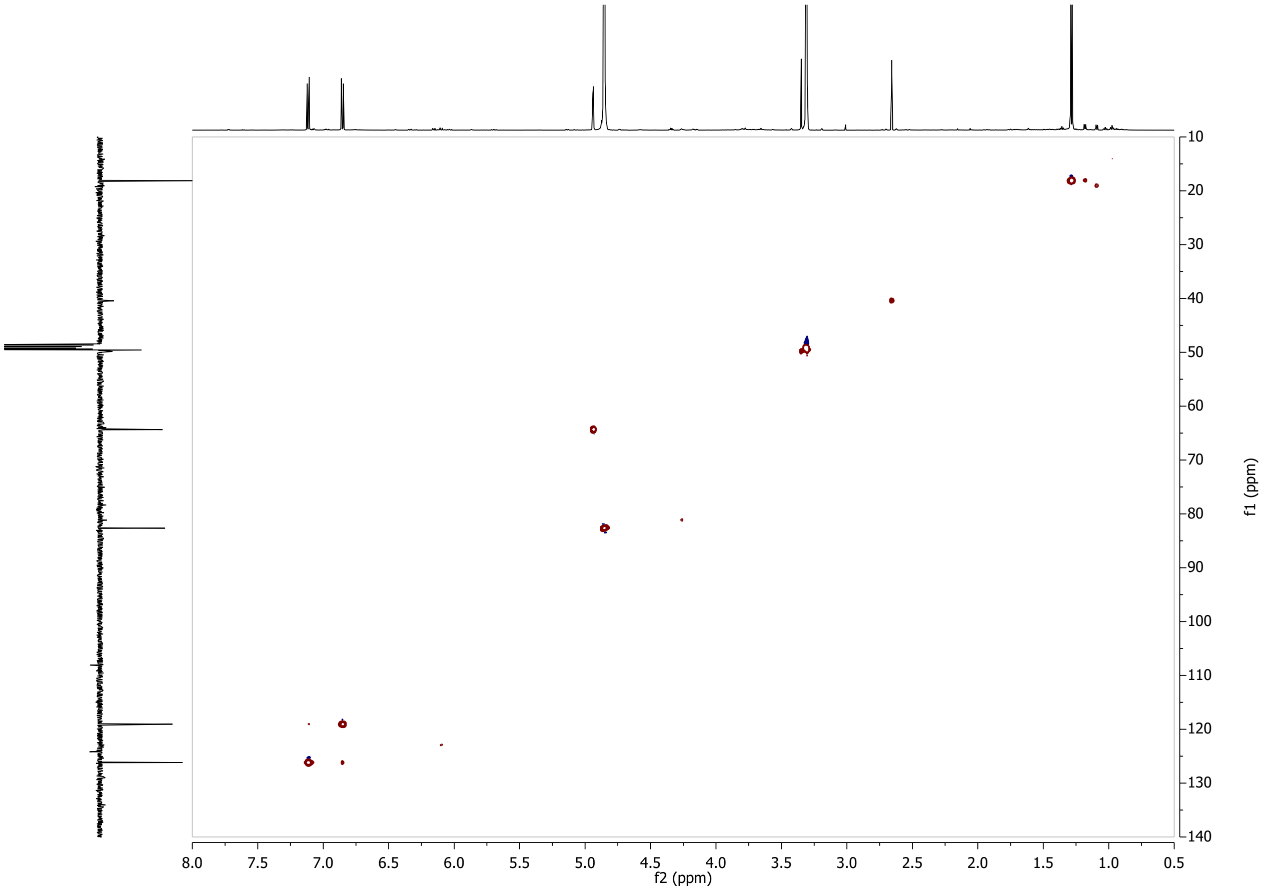

Supplementary Figure 51. HSQC NMR spectrum of **7** in MeOD at 600 MHz.

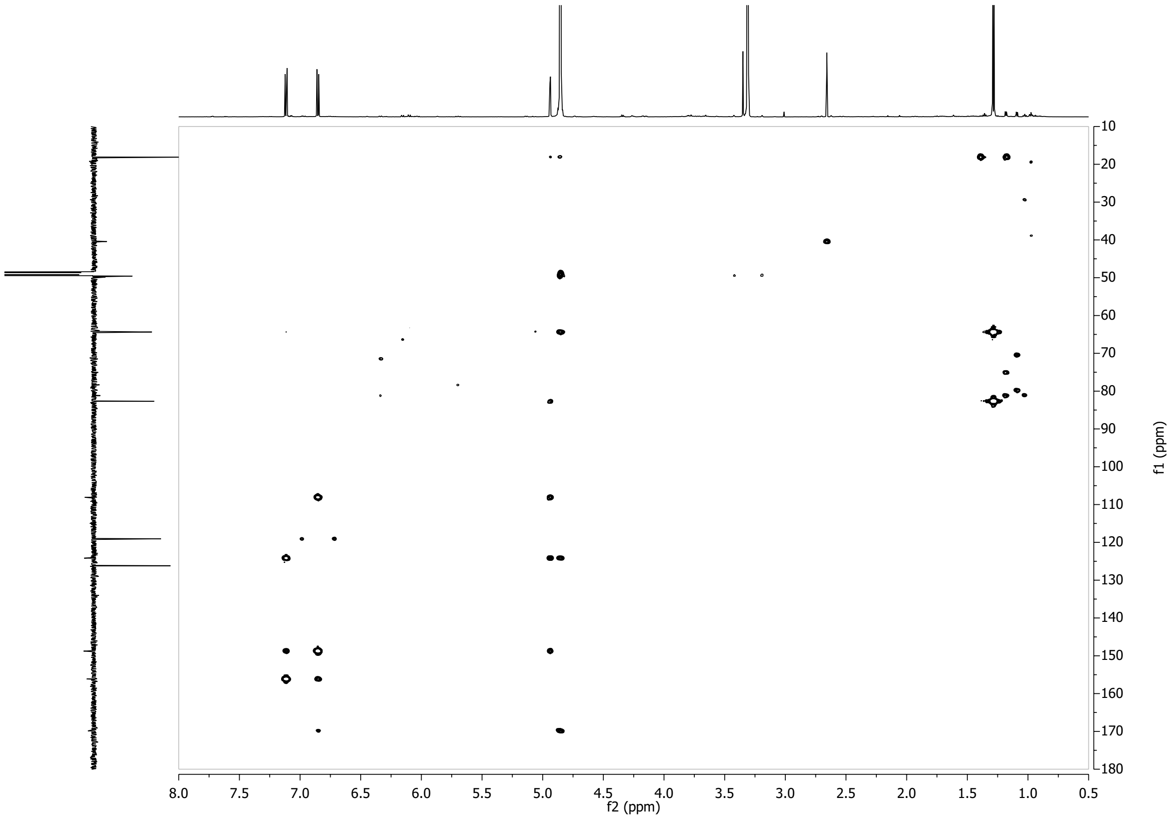

Supplementary Figure 52. HMBC NMR spectrum of **7** in MeOD at 600 MHz.

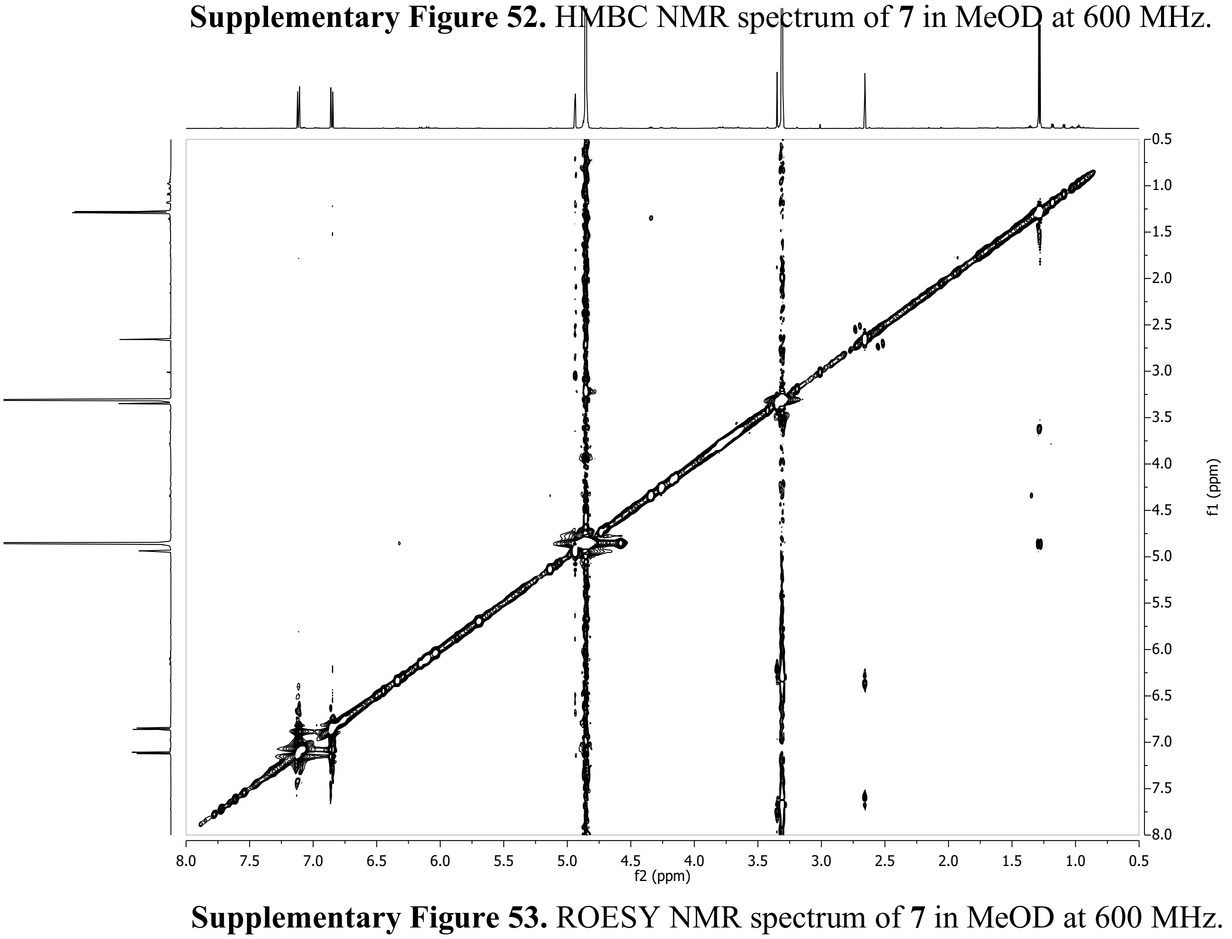

Supplementary Figure 53. ROESY NMR spectrum of **7** in MeOD at 600 MHz.

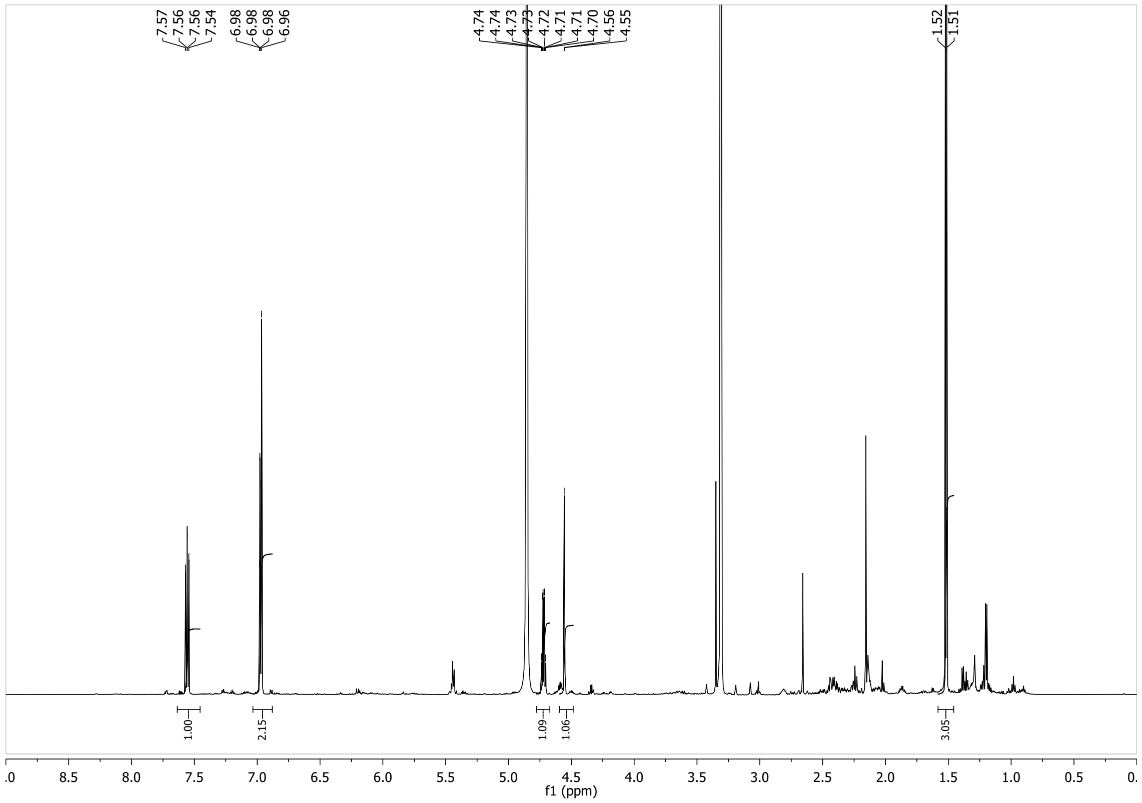

Supplementary Figure 54. ^{1}H NMR spectrum of **8** in MeOD at 600 MHz.

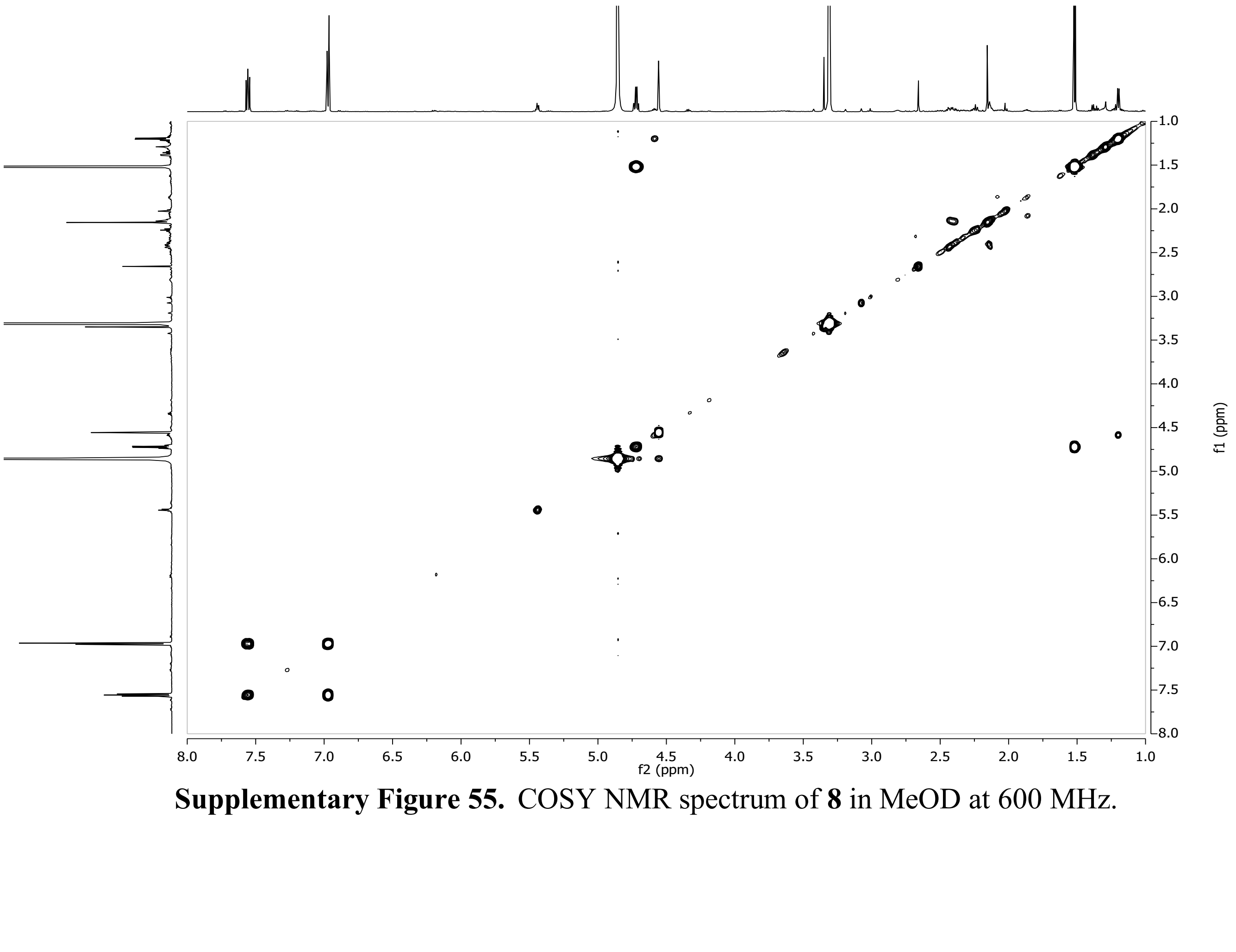

Supplementary Figure 55. COSY NMR spectrum of **8** in MeOD at 600 MHz.

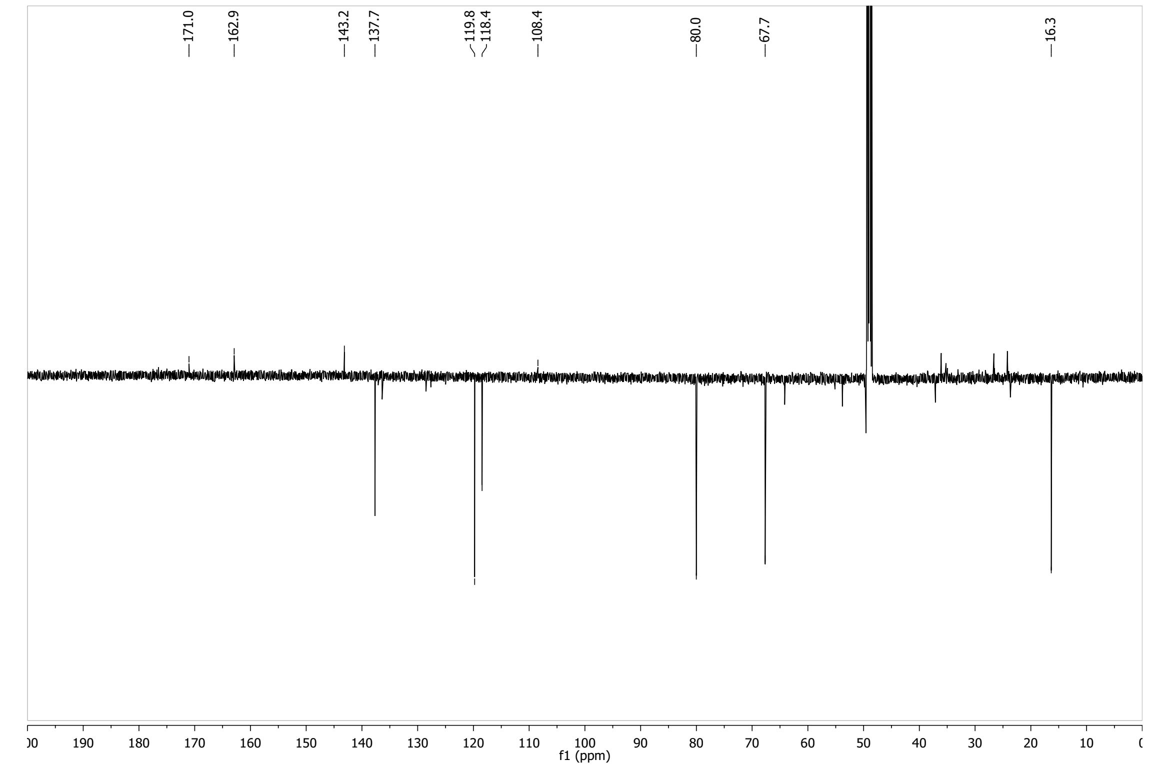

Supplementary Figure 56. ^{1}C NMR spectrum of **8** in MeOD at 600 MHz.

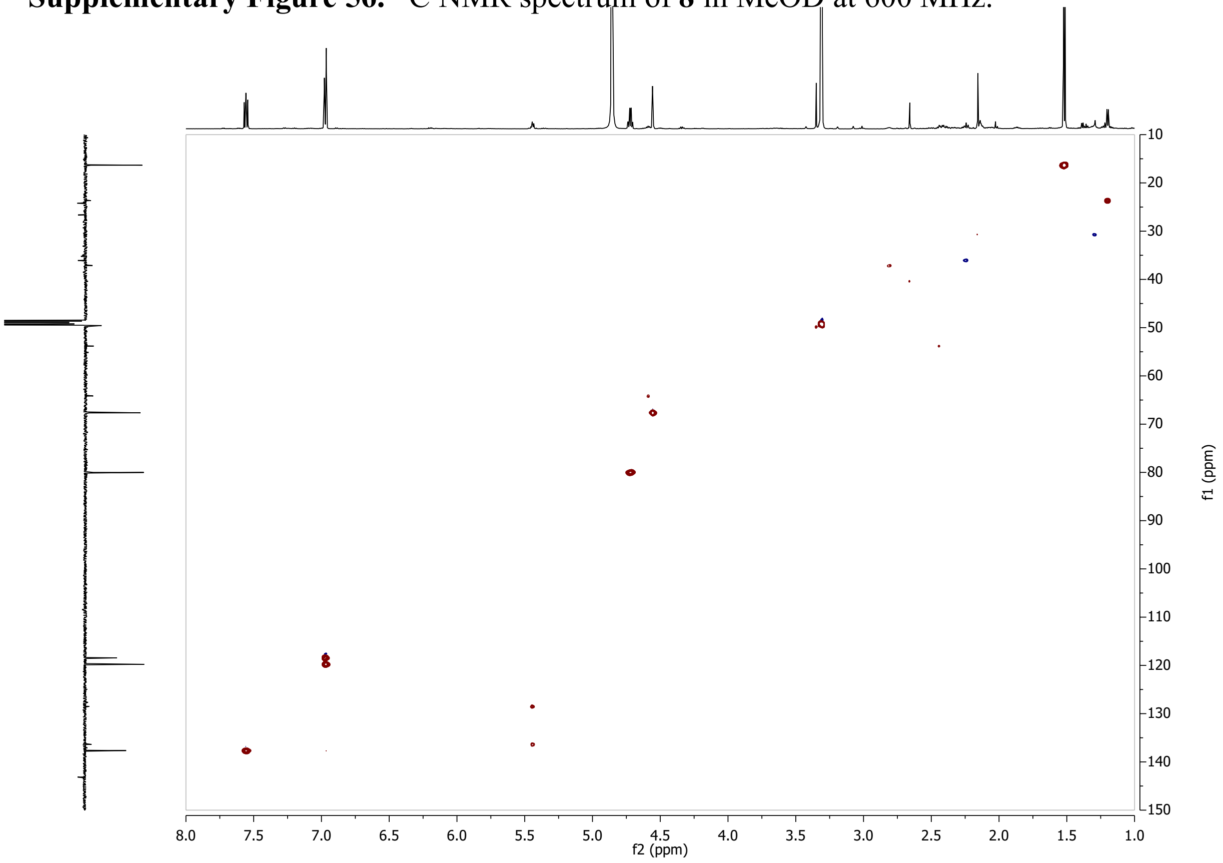

Supplementary Figure 57. HSQC NMR spectrum of **8** in MeOD at 600 MHz.

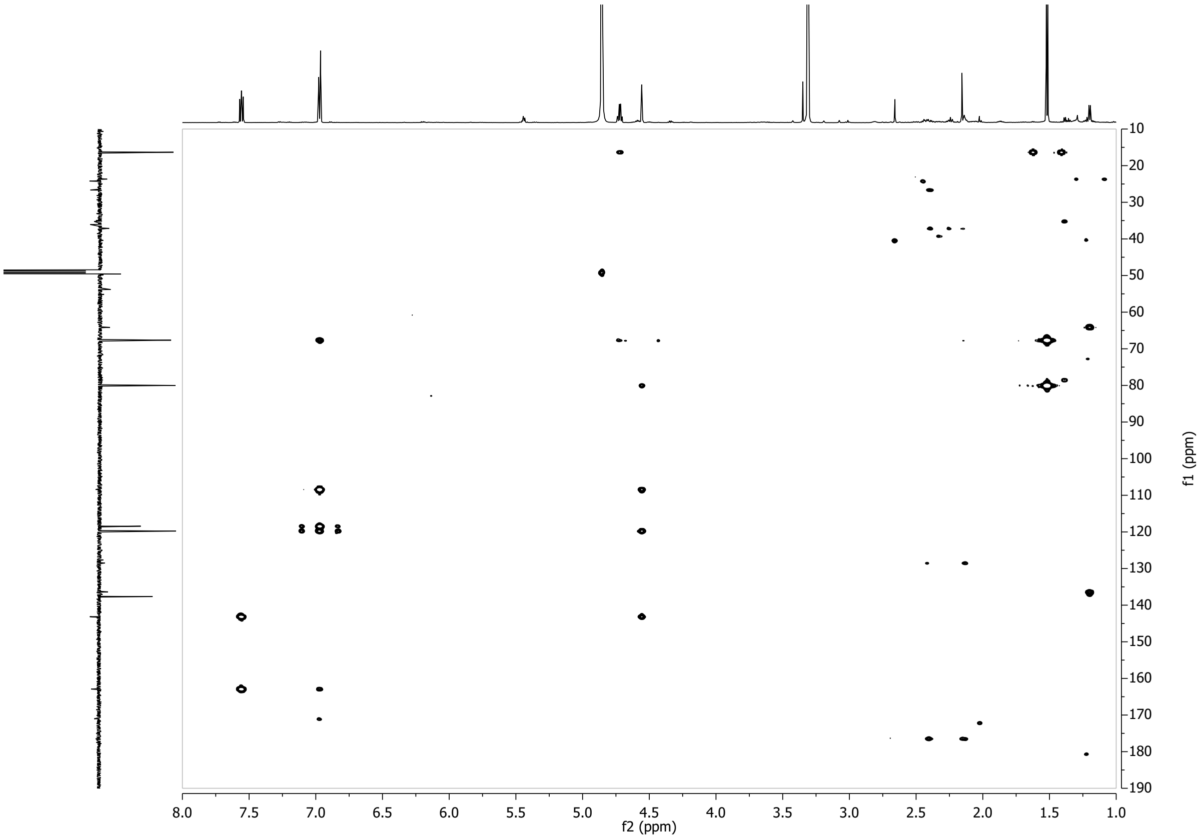

Supplementary Figure 58. HMBC NMR spectrum of **8** in MeOD at 600 MHz.

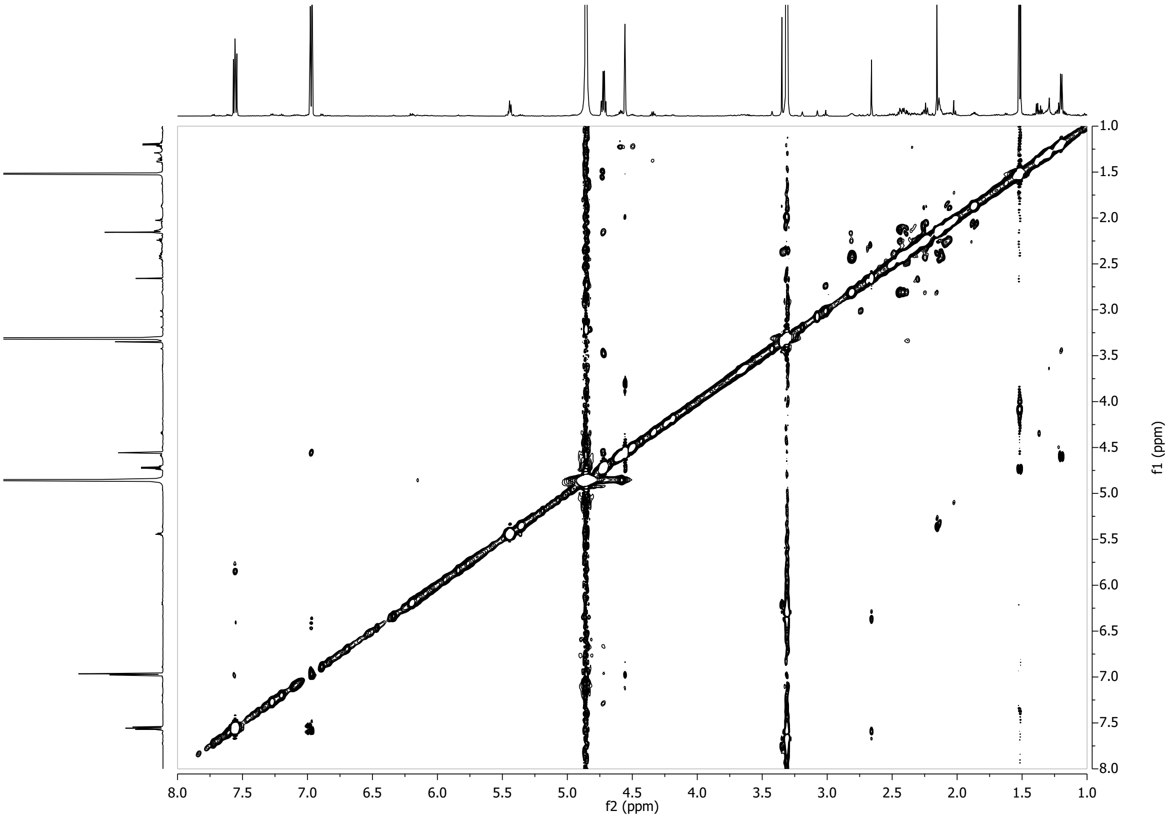

Supplementary Figure 59. ROESY NMR spectrum of **8** in MeOD at 600 MHz.

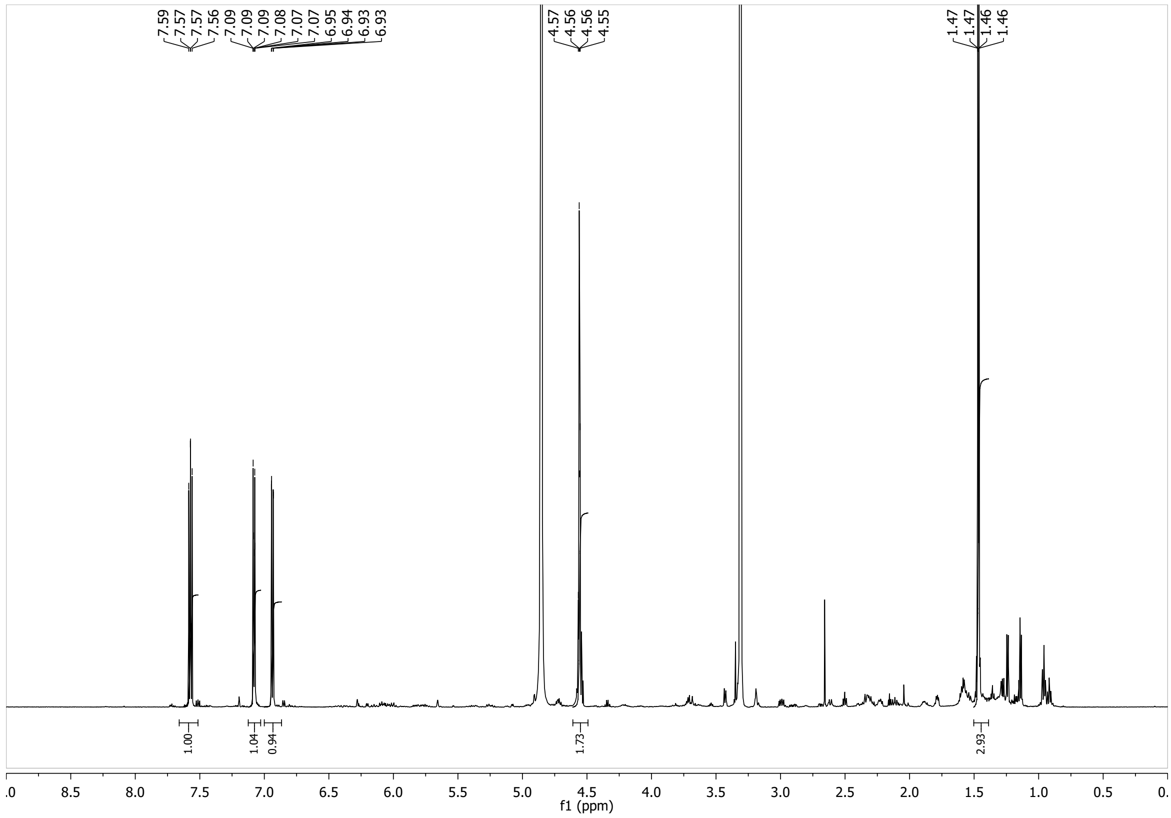

Supplementary Figure 60. ^{1}H NMR spectrum of **9** in MeOD at 600 MHz.

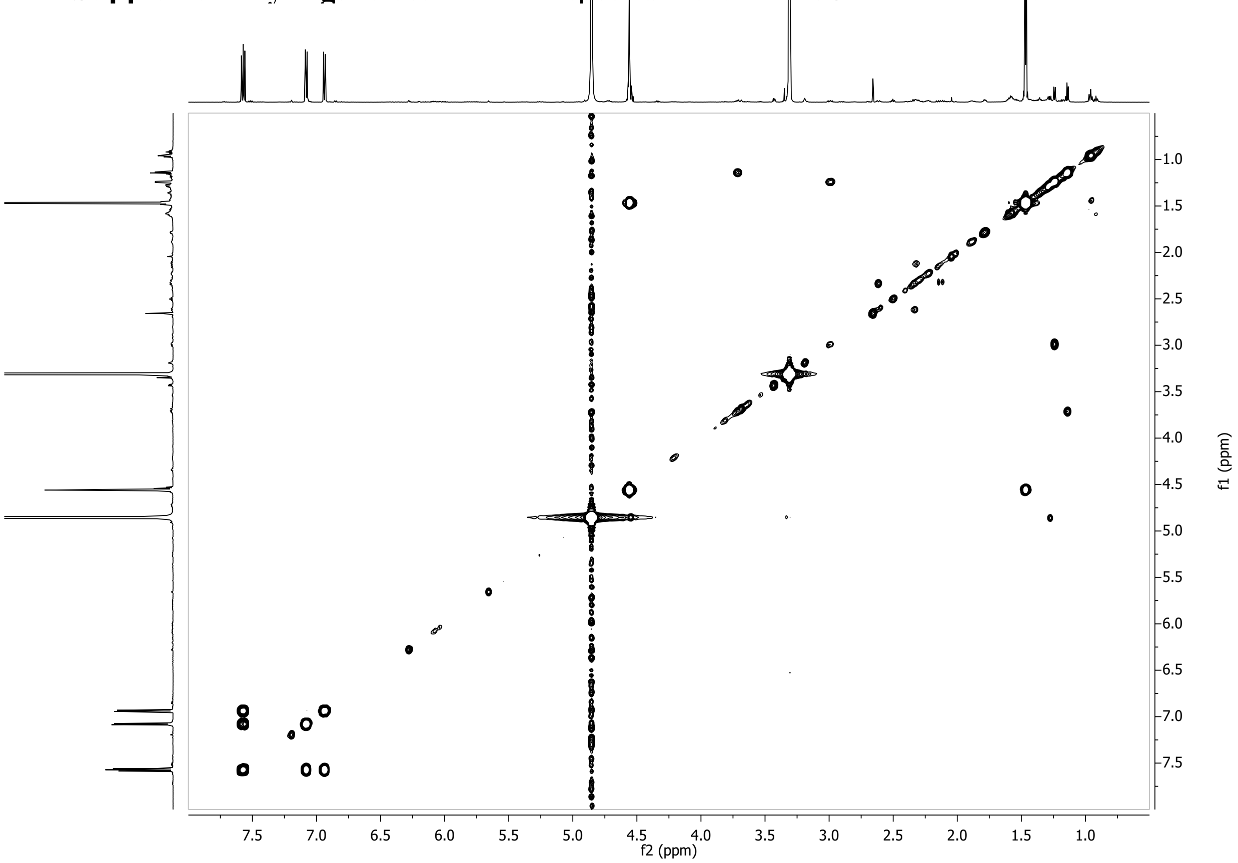

Supplementary Figure 61. COSY NMR spectrum of **9** in MeOD at 600 MHz.

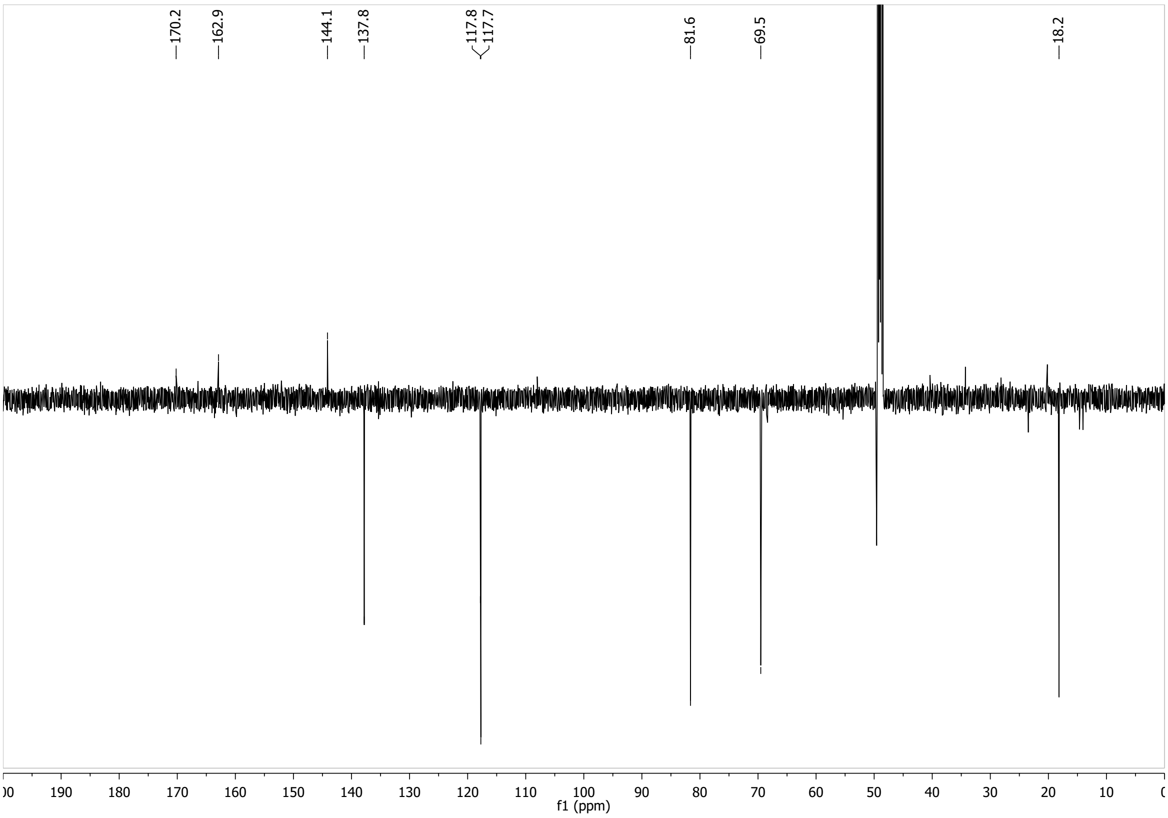

Supplementary Figure 62. ^{1}C NMR spectrum of **9** in MeOD at 600 MHz.

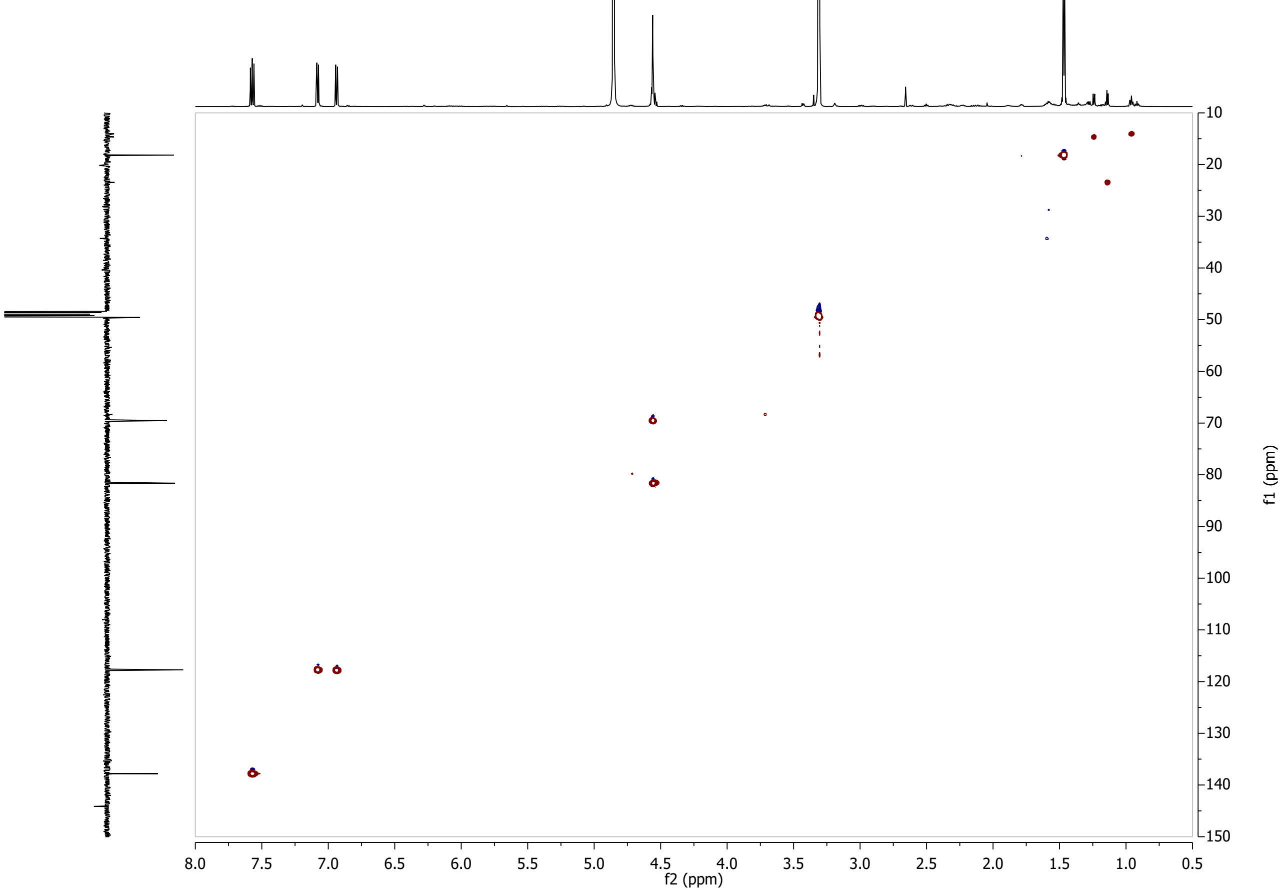

Supplementary Figure 63. HSQC NMR spectrum of **9** in MeOD at 600 MHz.

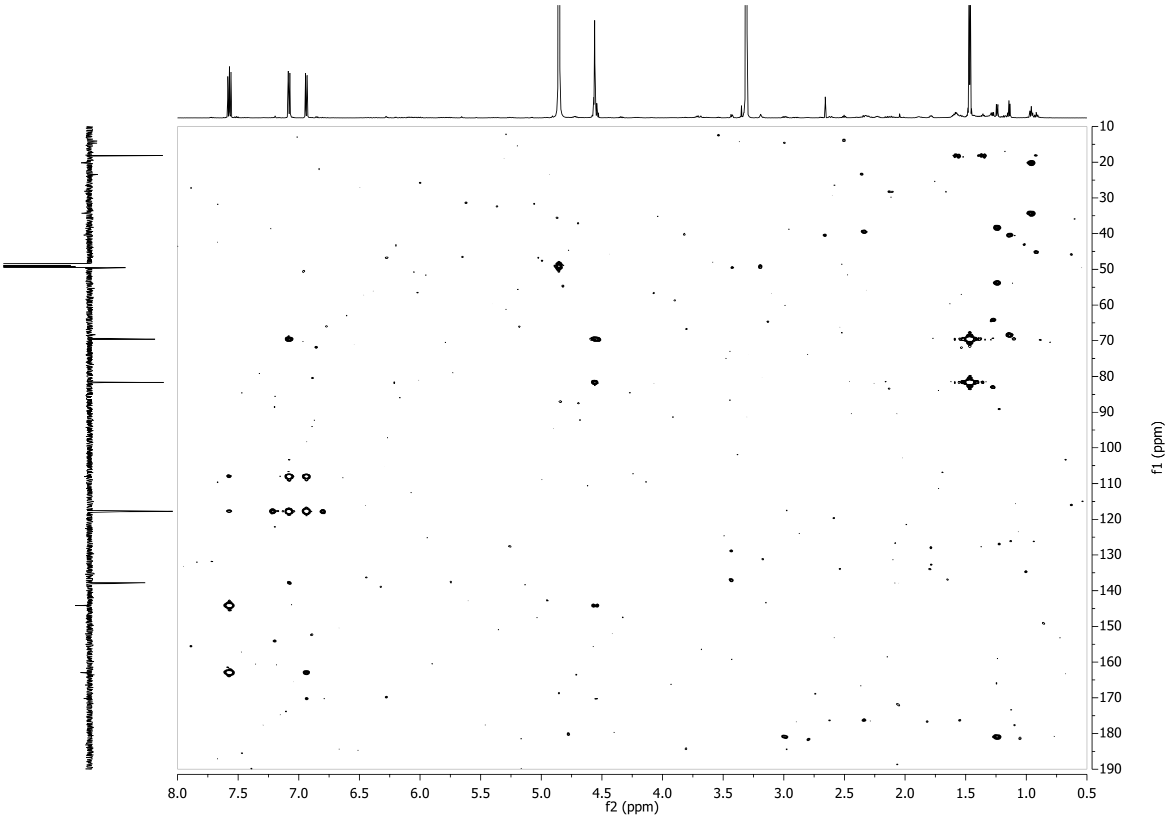

Supplementary Figure 64. HMBC NMR spectrum of **9** in MeOD at 600 MHz.

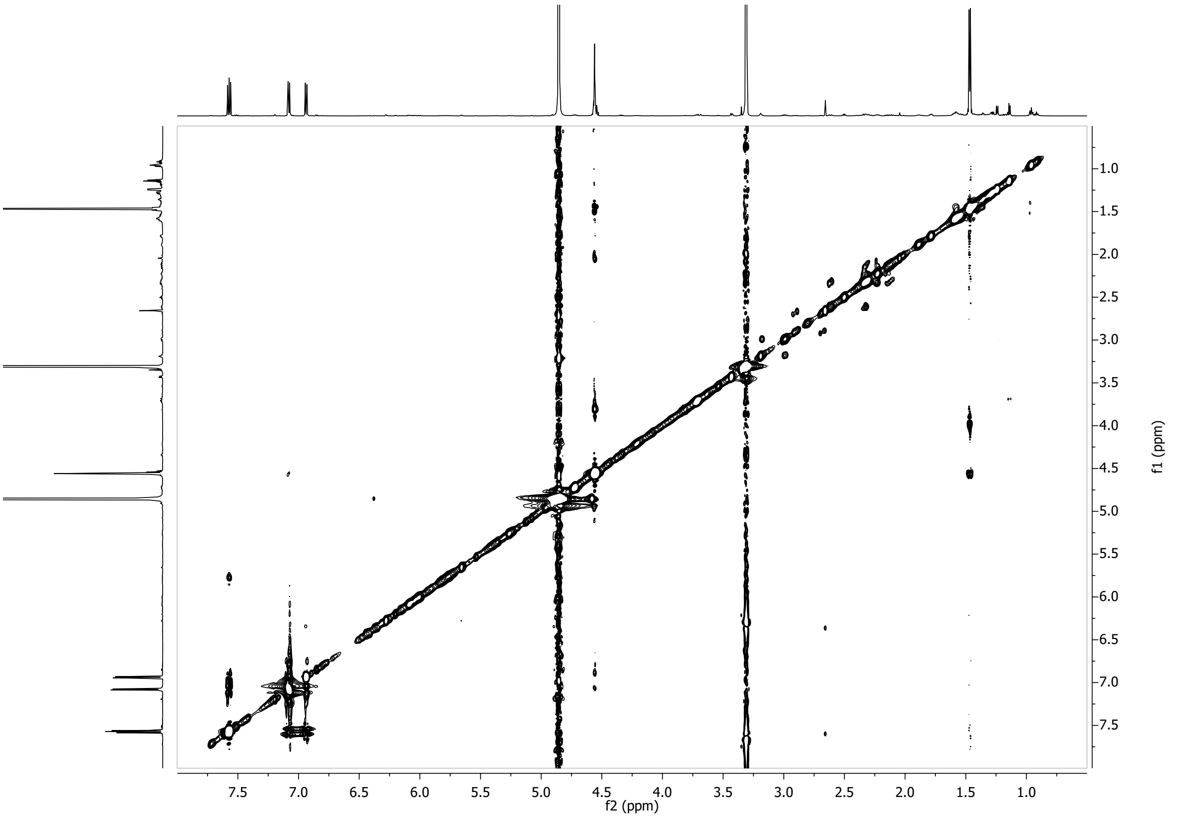

Supplementary Figure 65. ROESY NMR spectrum of **9** in MeOD at 600 MHz.

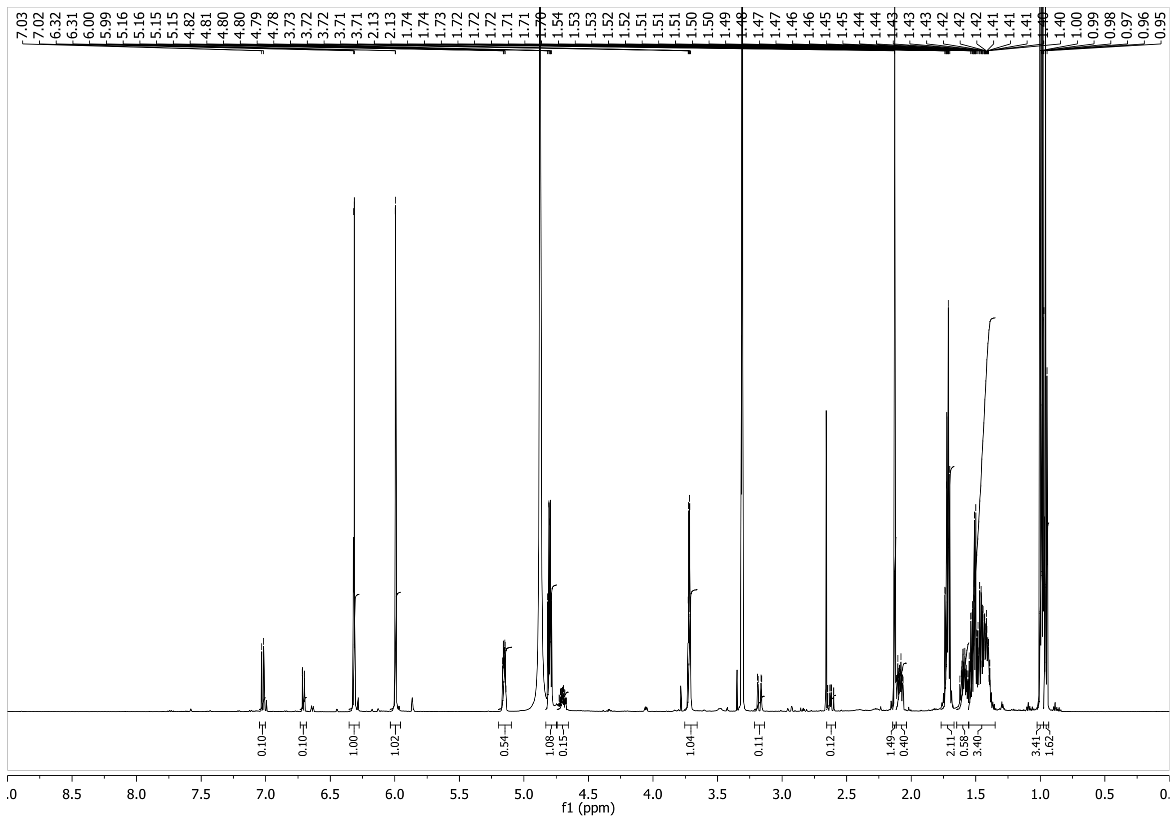

Supplementary Figure 66. ^{1}H NMR spectrum of **10, 11** and **18** (fraction F19) in MeOD at 600 MHz.

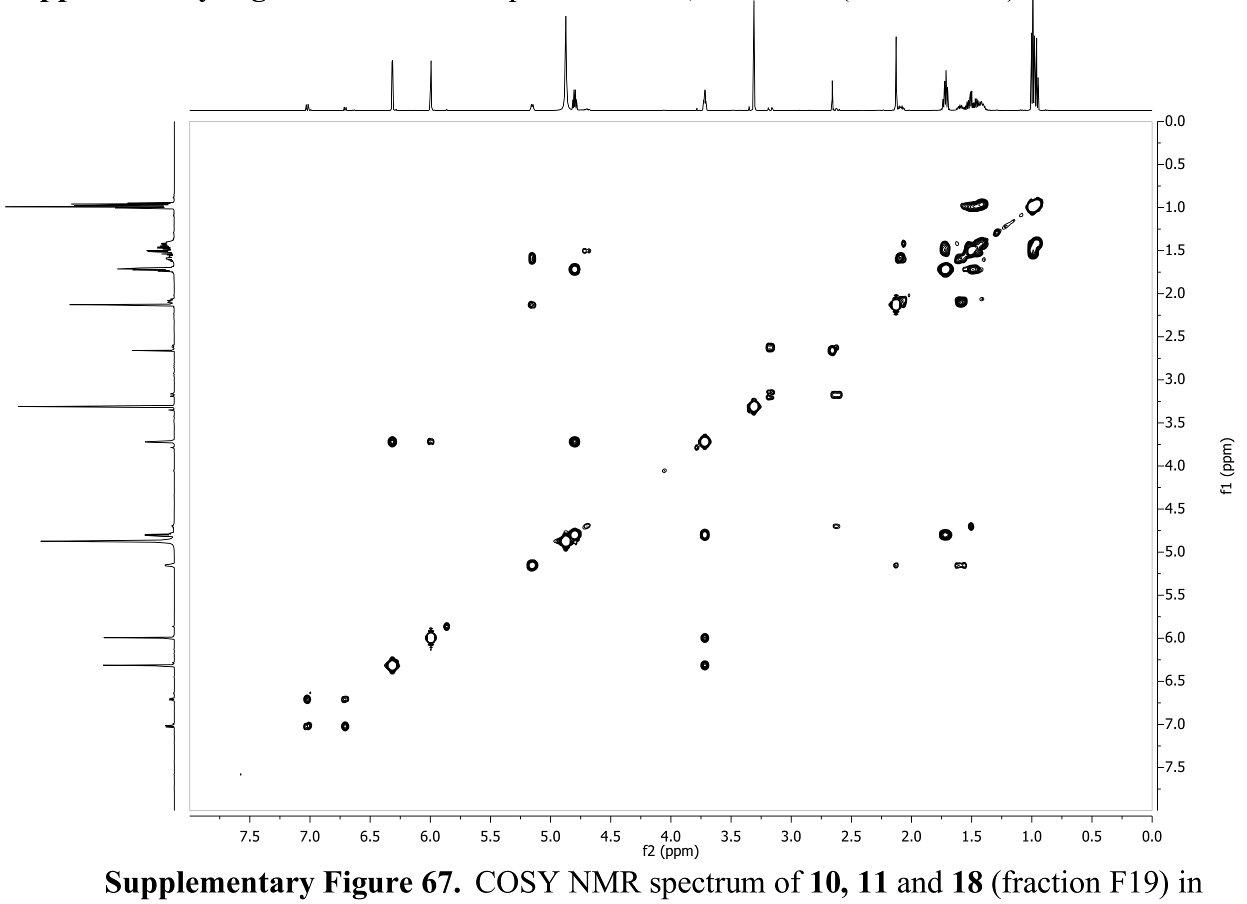

Supplementary Figure 67. COSY NMR spectrum of **10, 11** and **18** (fraction F19) in MeOD at 600 MHz.

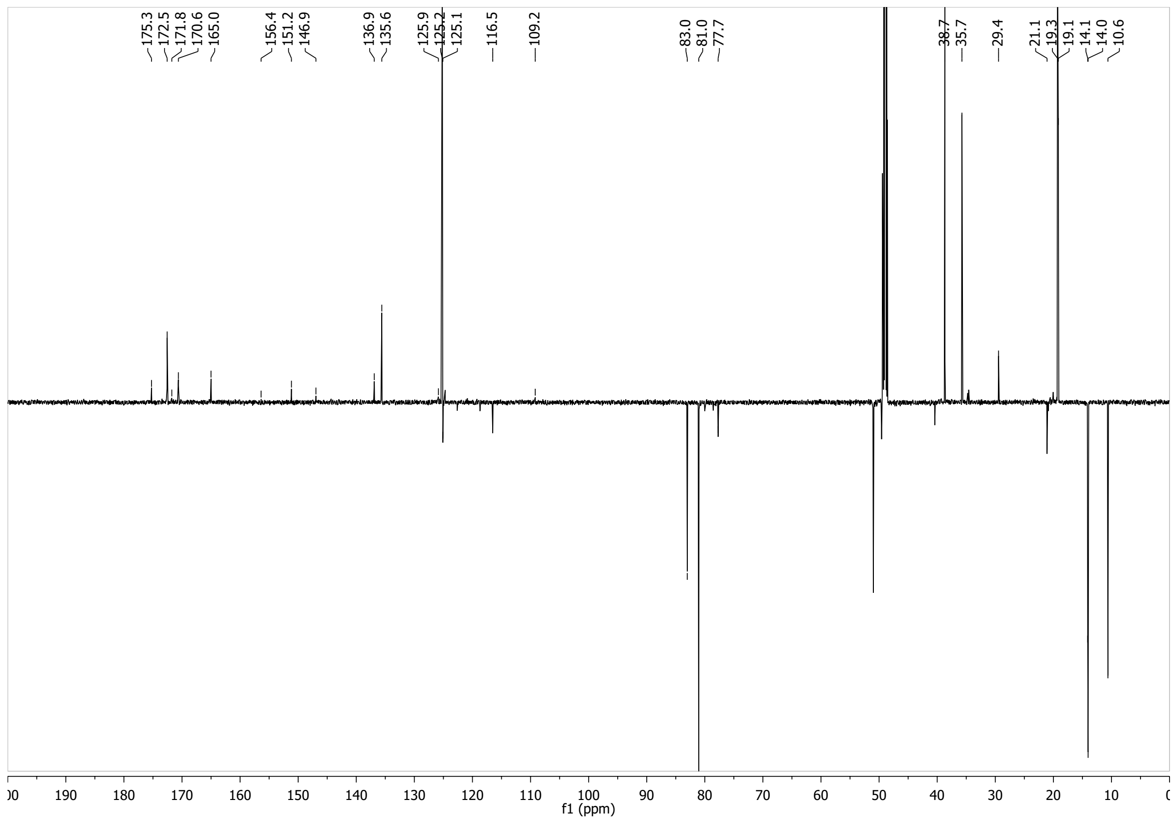

Supplementary Figure 68. [1]C NMR spectrum of **10,11** and **18** (fraction F19) in MeOD at 600 MHz.

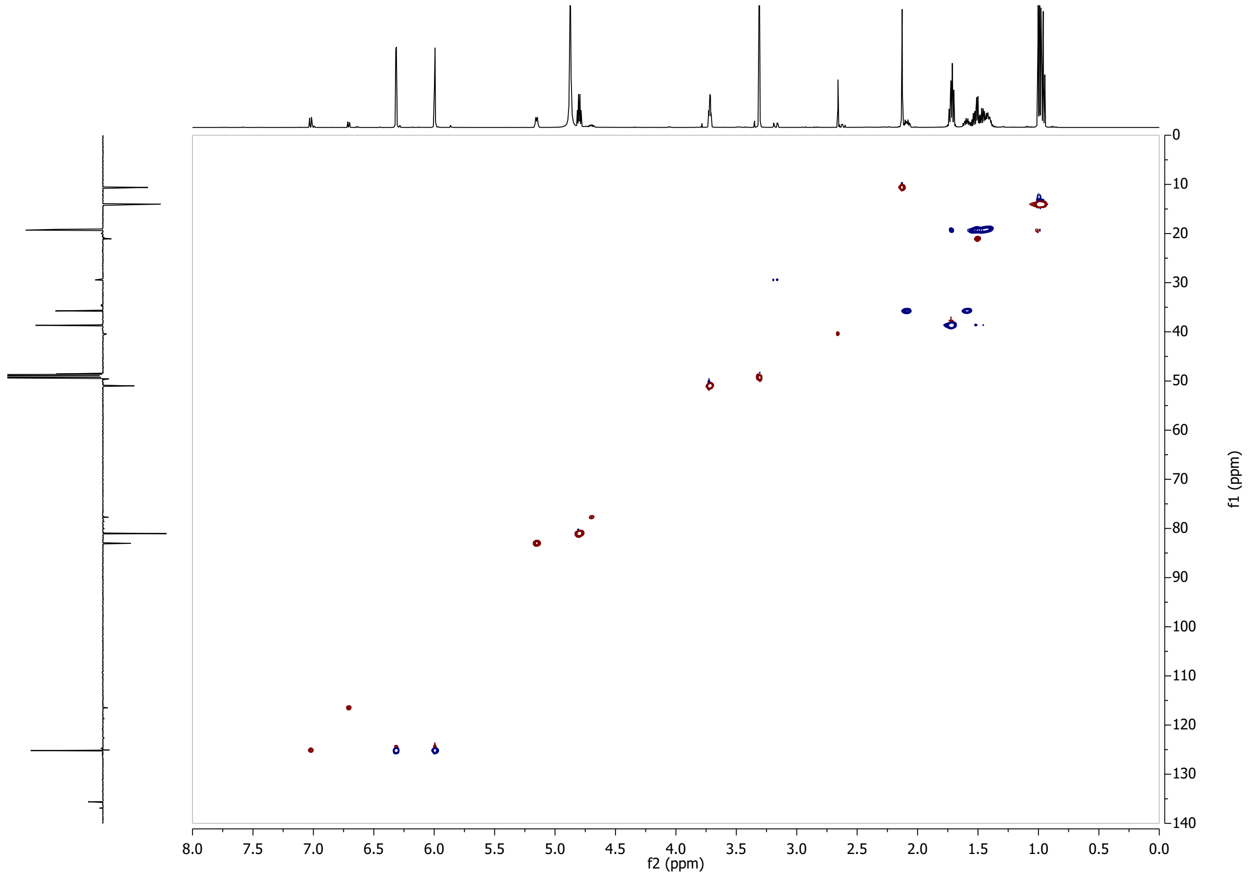

Supplementary Figure 69. HSQC NMR spectrum of **10,11** and **18** (fraction F19) in MeOD at 600 MHz.

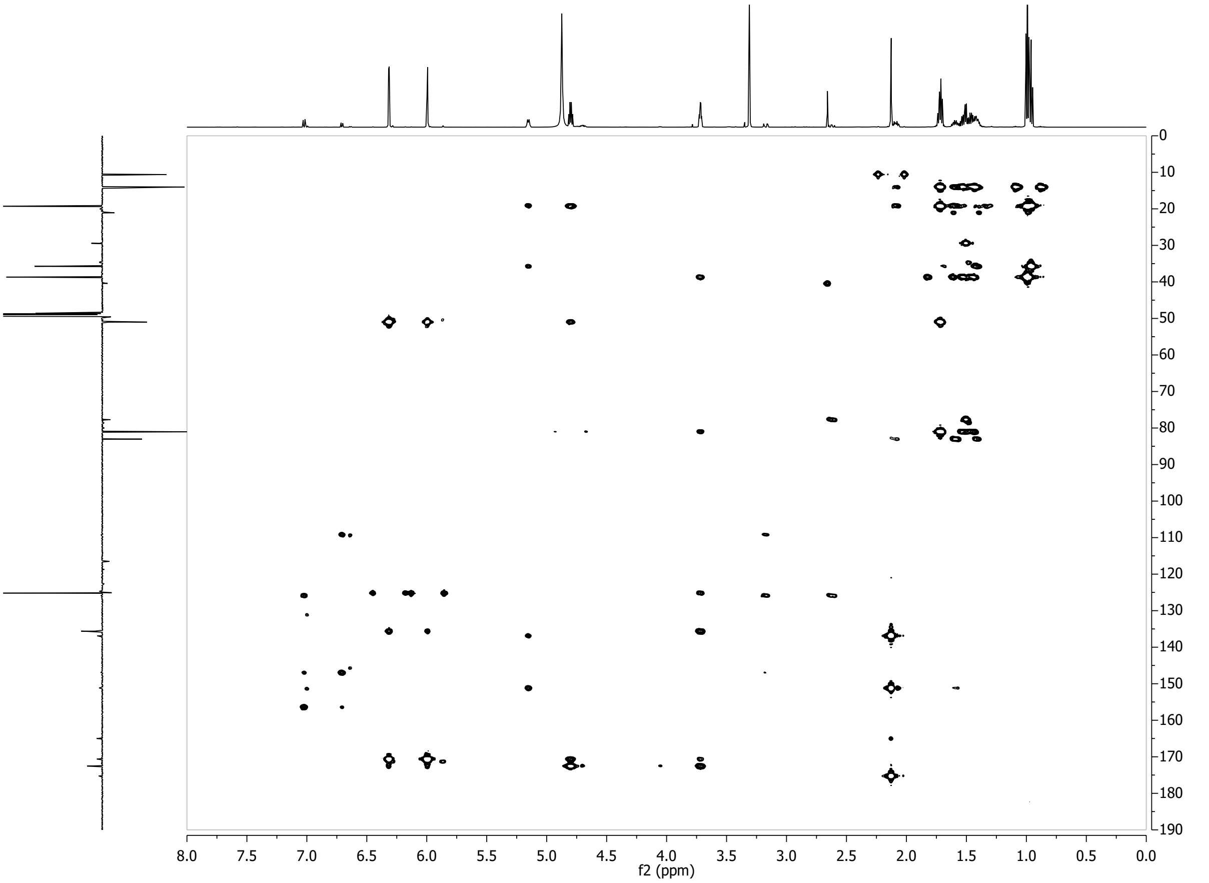

Supplementary Figure 70. HMBC NMR spectrum of **10,11** and **18** (fraction F19) in MeOD at 600 MHz.

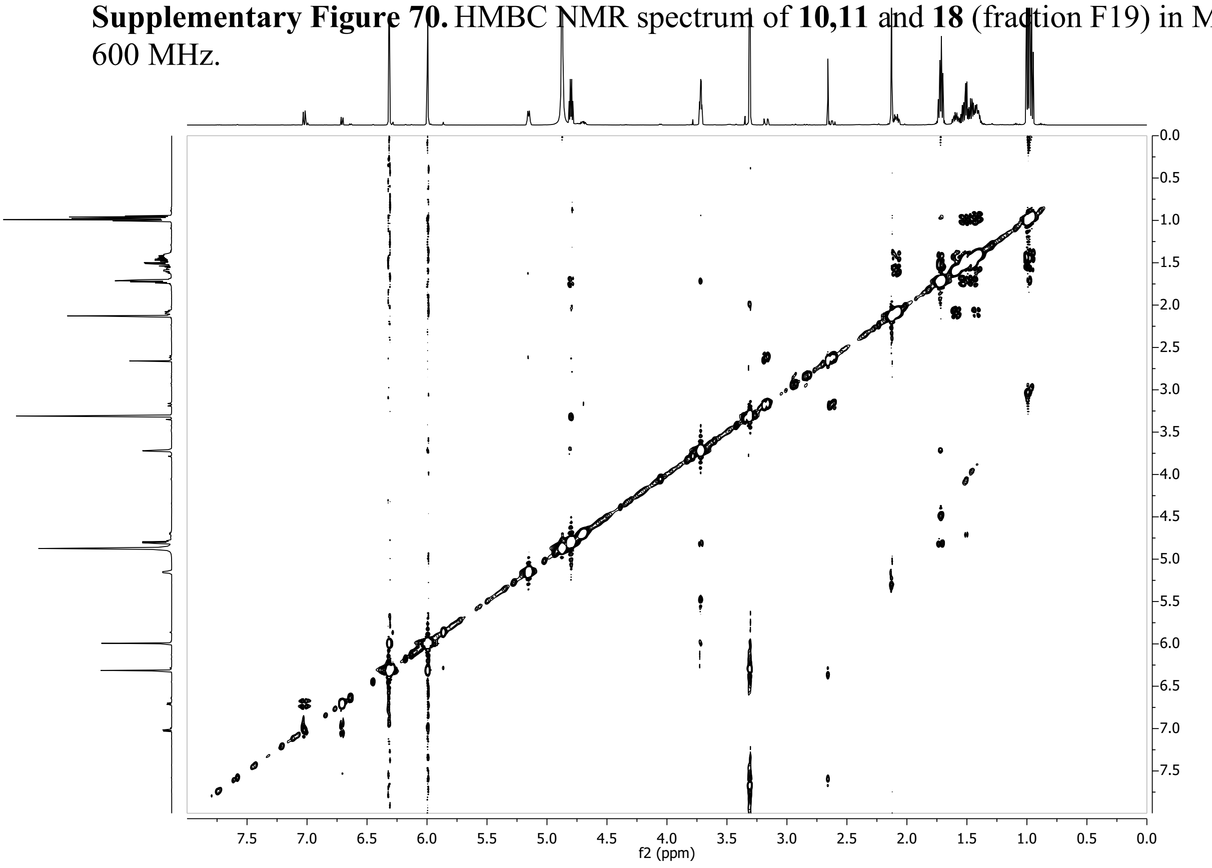

Supplementary Figure 71. ROESY NMR spectrum of **10,11** and **18** (fraction F19) in MeOD at 600 MHz.

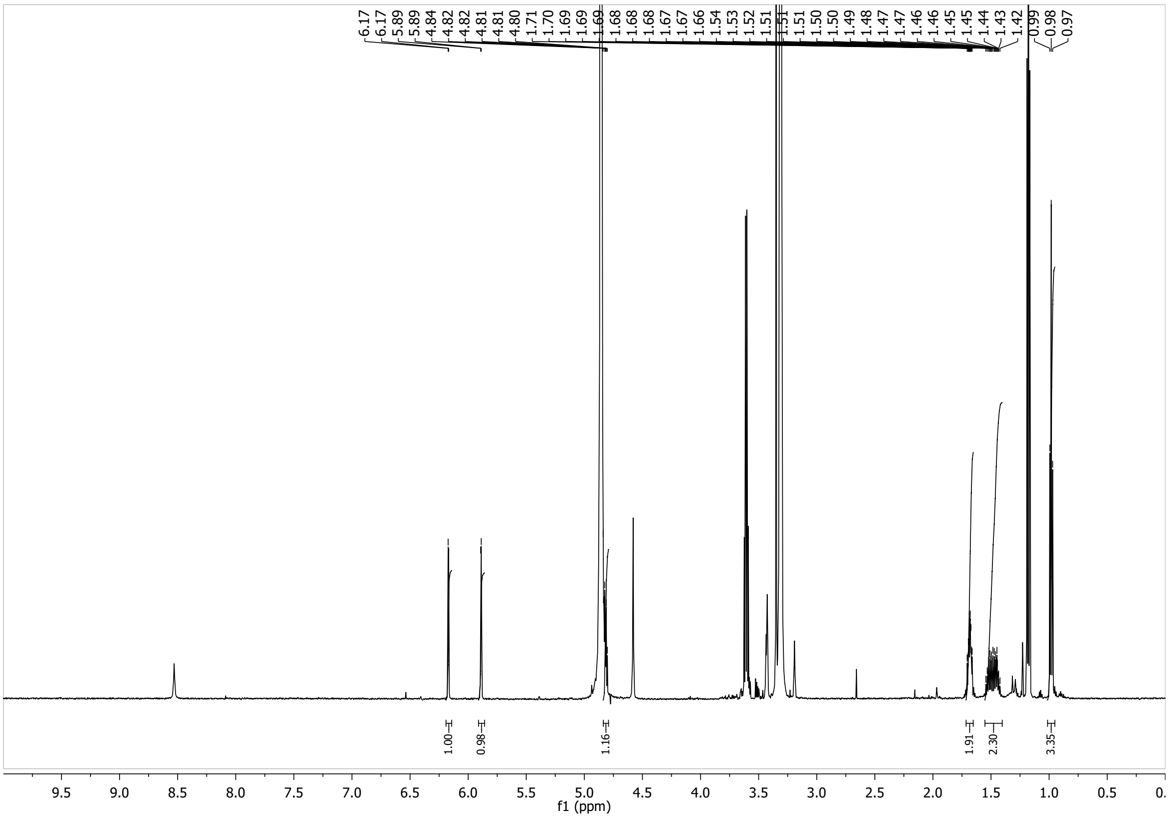

Supplementary Figure 72. ^{1}H NMR spectrum of **10** MeOD at 600 MHz.

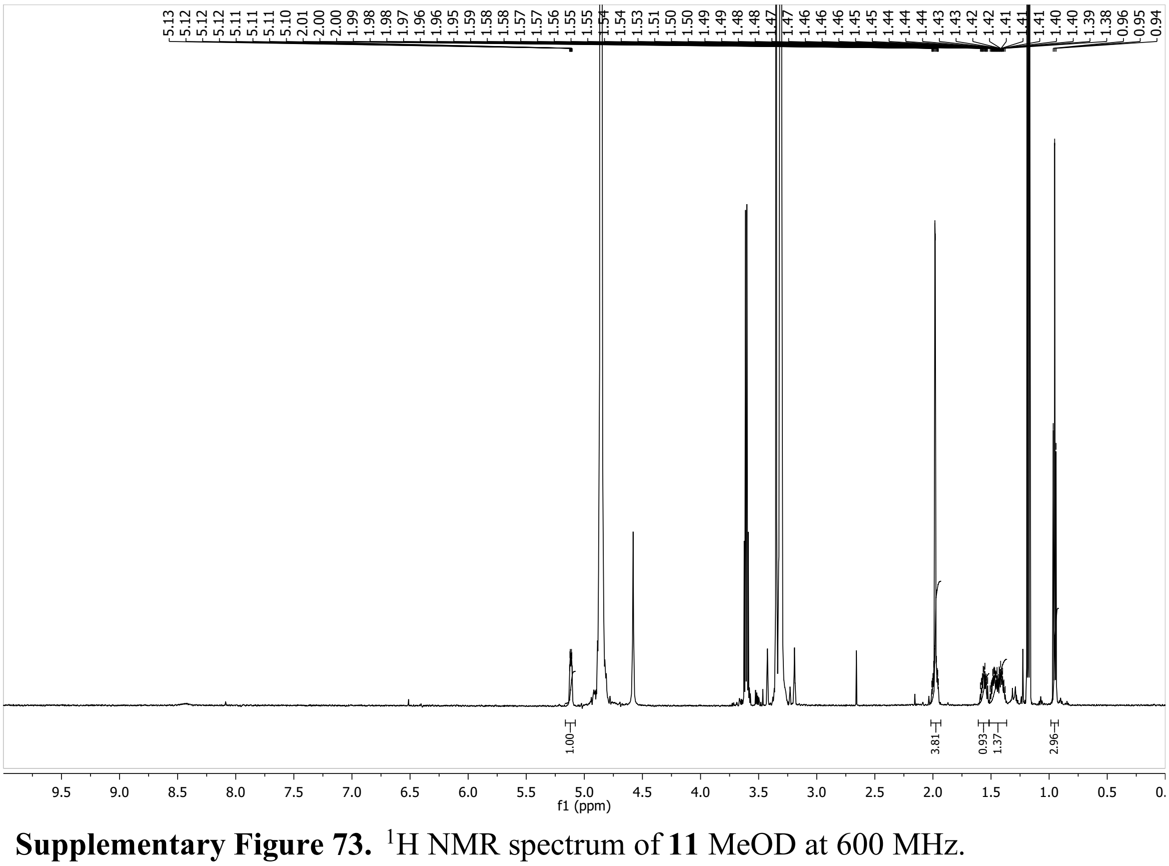

Supplementary Figure 73. ^{1}H NMR spectrum of **11** MeOD at 600 MHz.

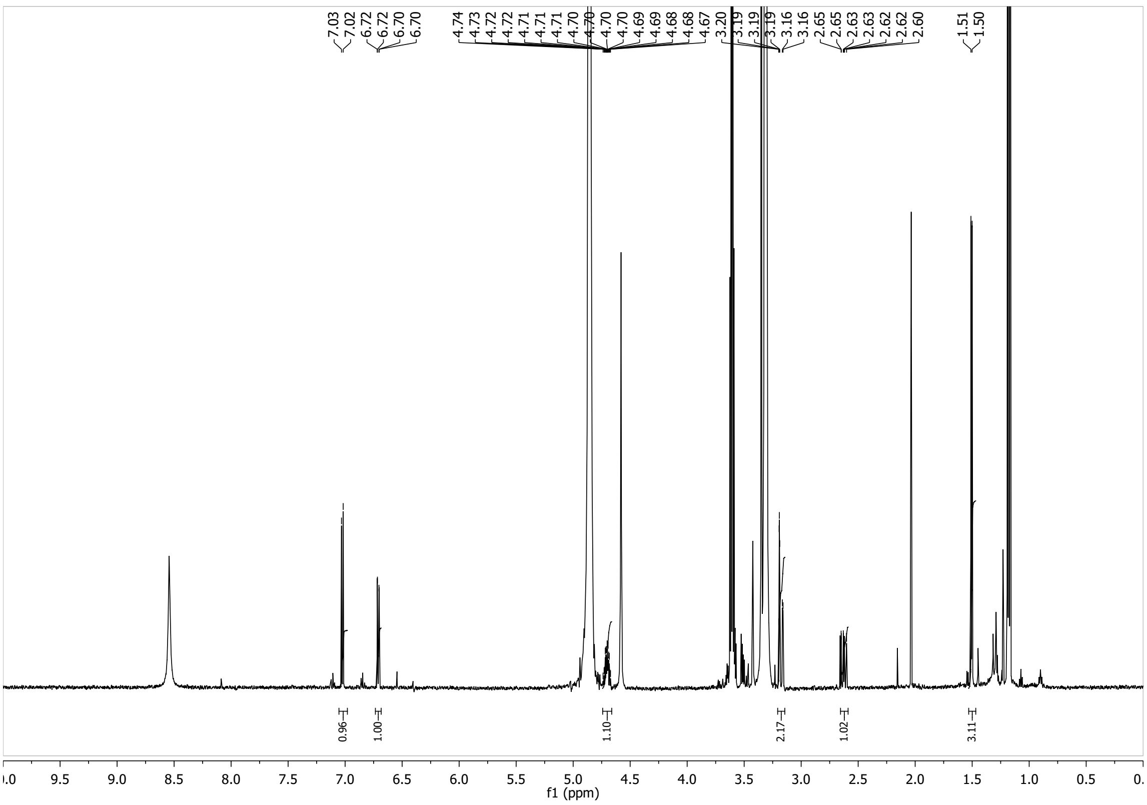

Supplementary Figure 74. ^{1}H NMR spectrum of **18** MeOD at 600 MHz.

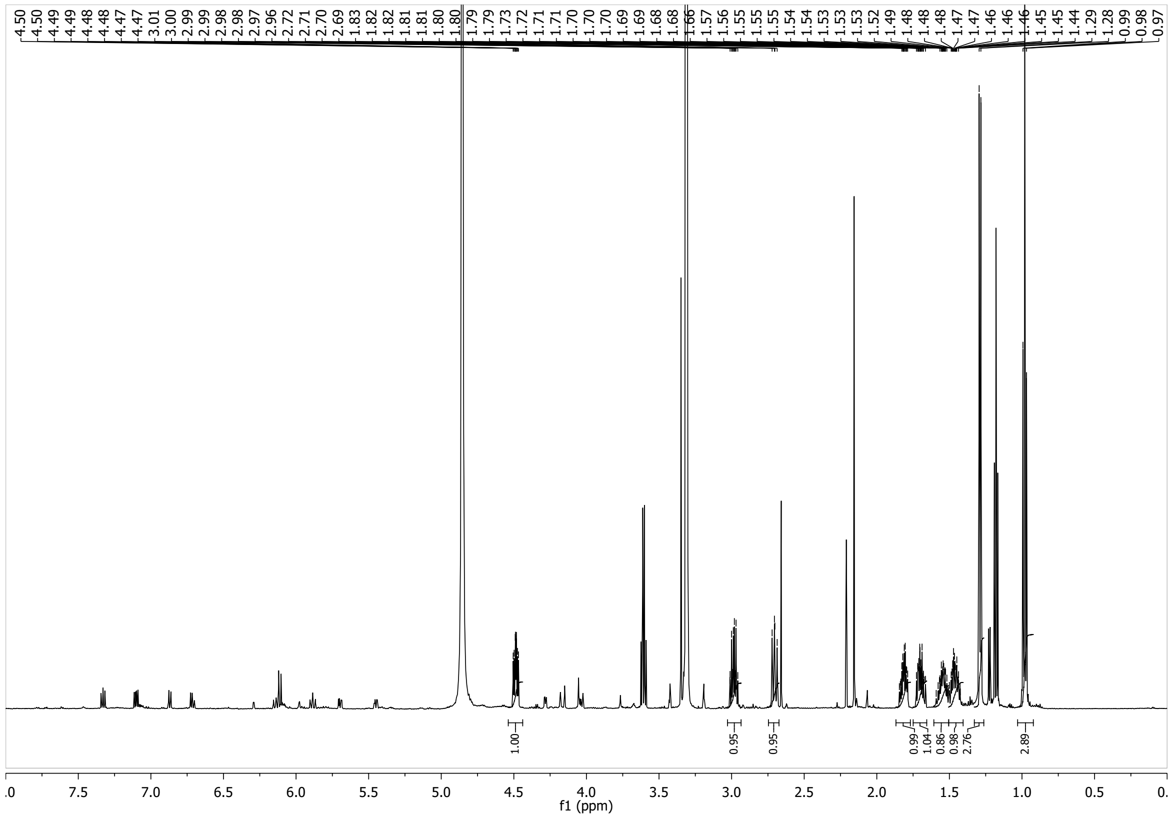

Supplementary Figure 75. ^{1}H NMR spectrum of **12** in MeOD at 600 MHz.

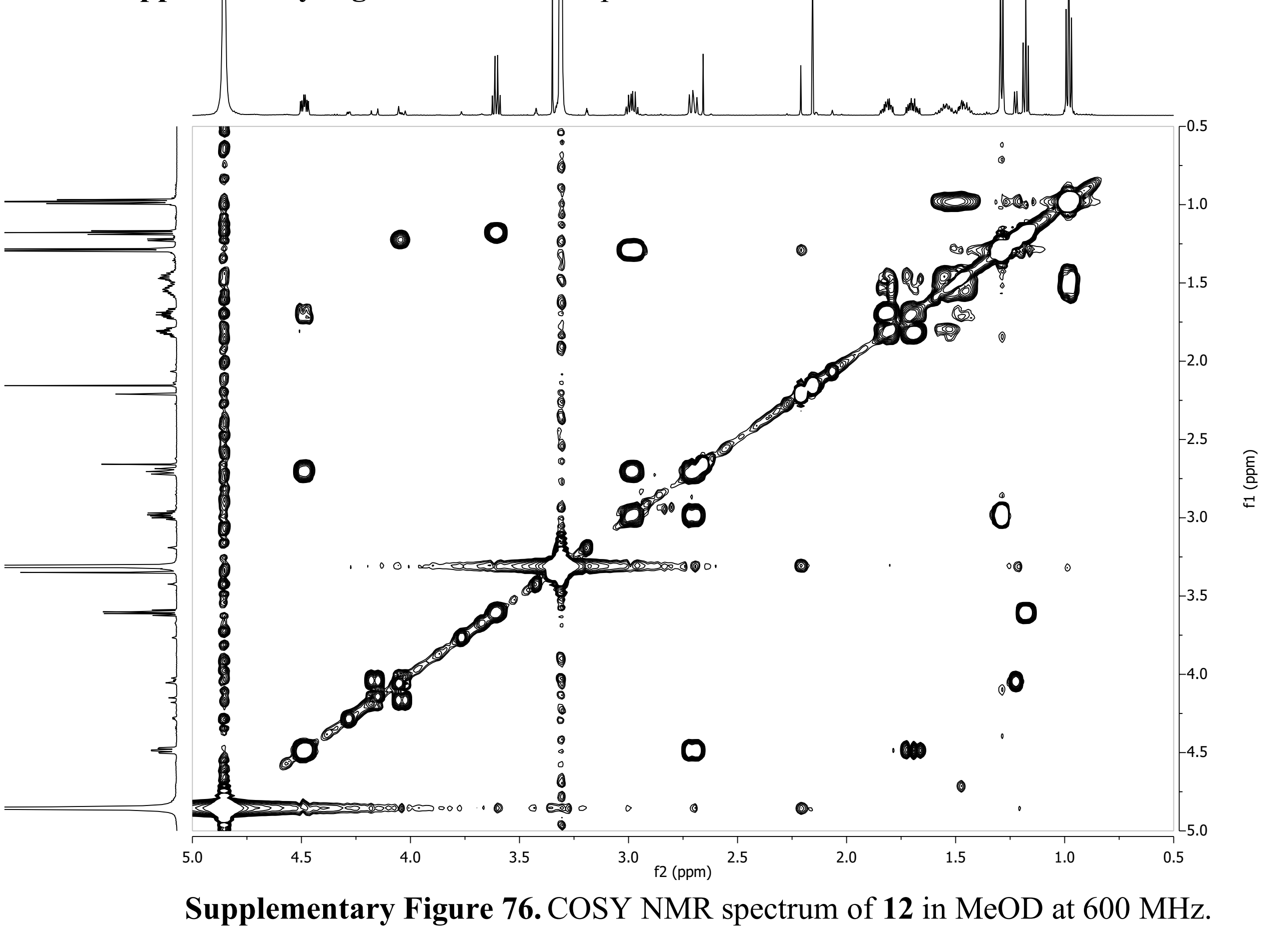

Supplementary Figure 76. COSY NMR spectrum of **12** in MeOD at 600 MHz.

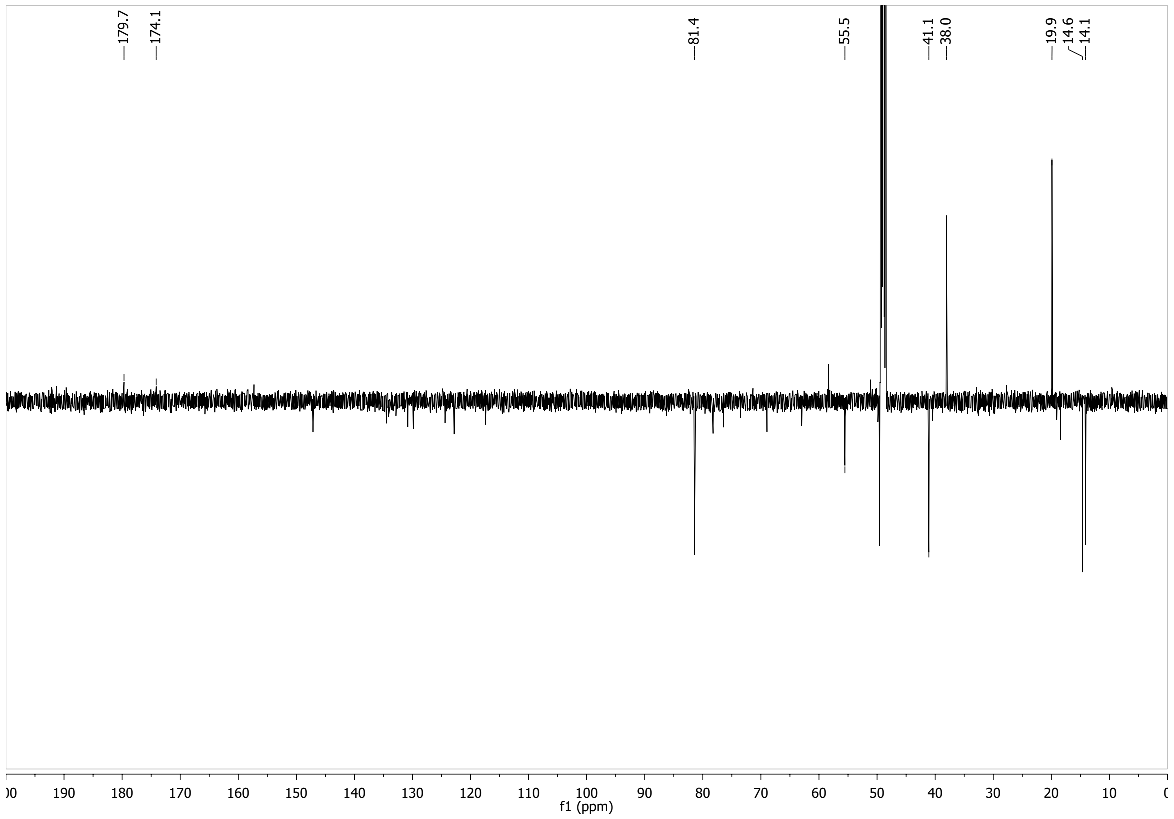

Supplementary Figure 77. ^{1}C NMR spectrum of **12** in MeOD at 600 MHz.

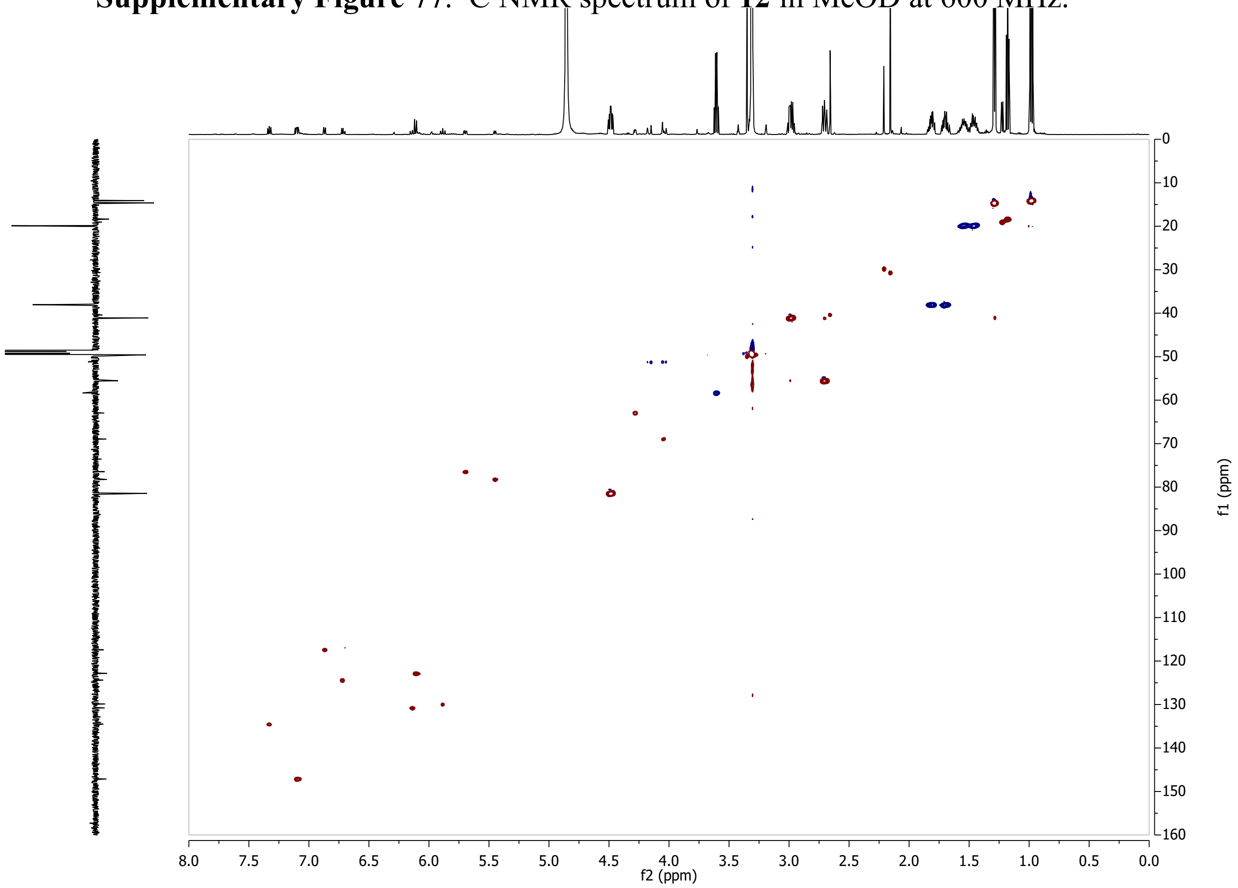

Supplementary Figure 78. HSQC NMR spectrum of **12** in MeOD at 600 MHz.

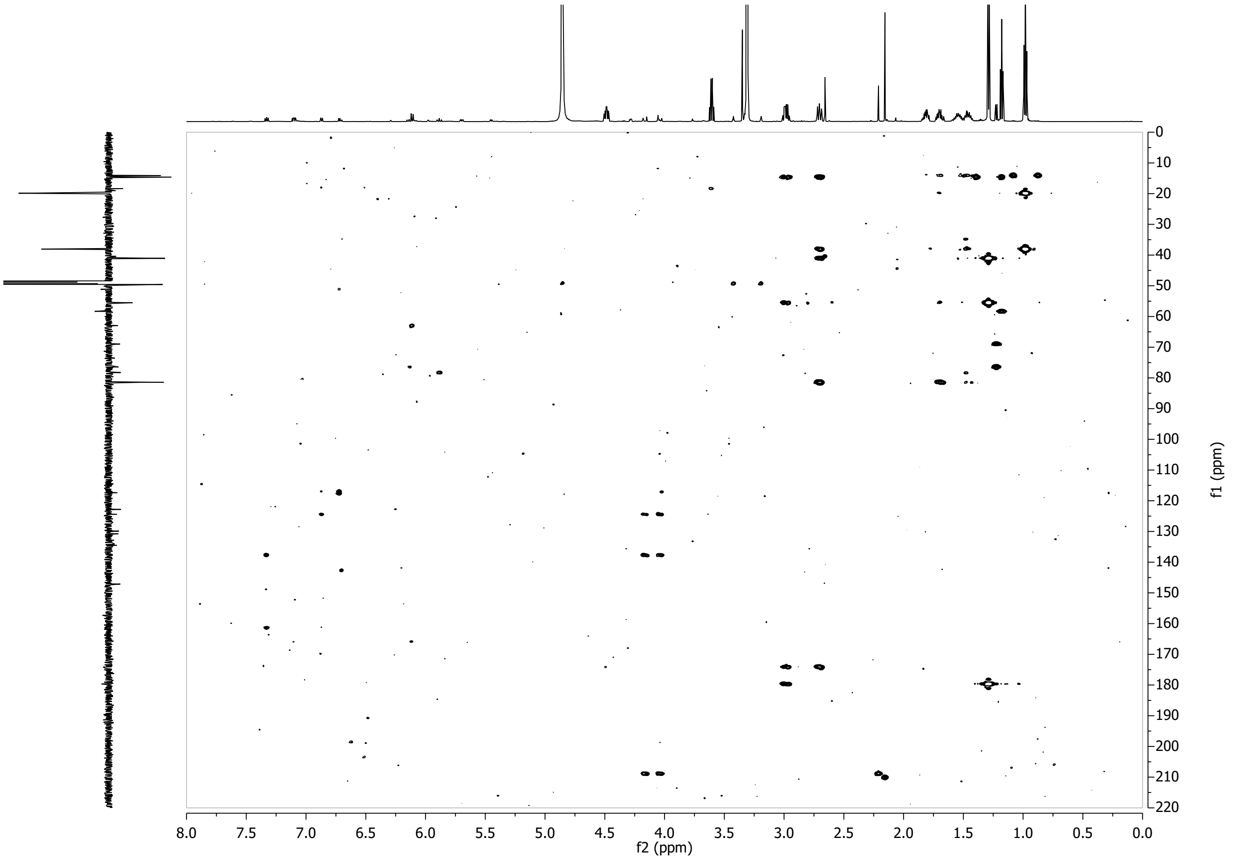

Supplementary Figure 79. HMBC NMR spectrum of **12** in MeOD at 600 MHz.

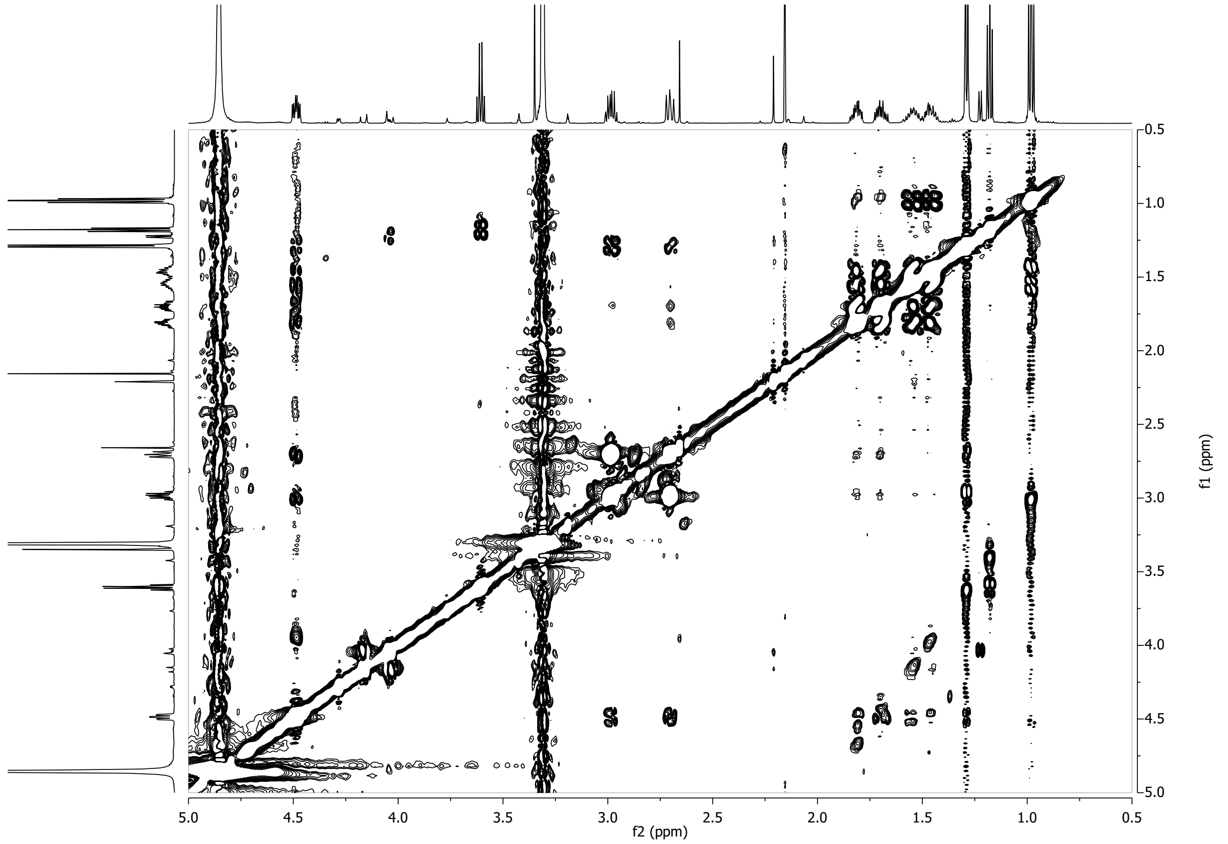

Supplementary Figure 80. ROESY NMR spectrum of **12** in MeOD at 600 MHz.

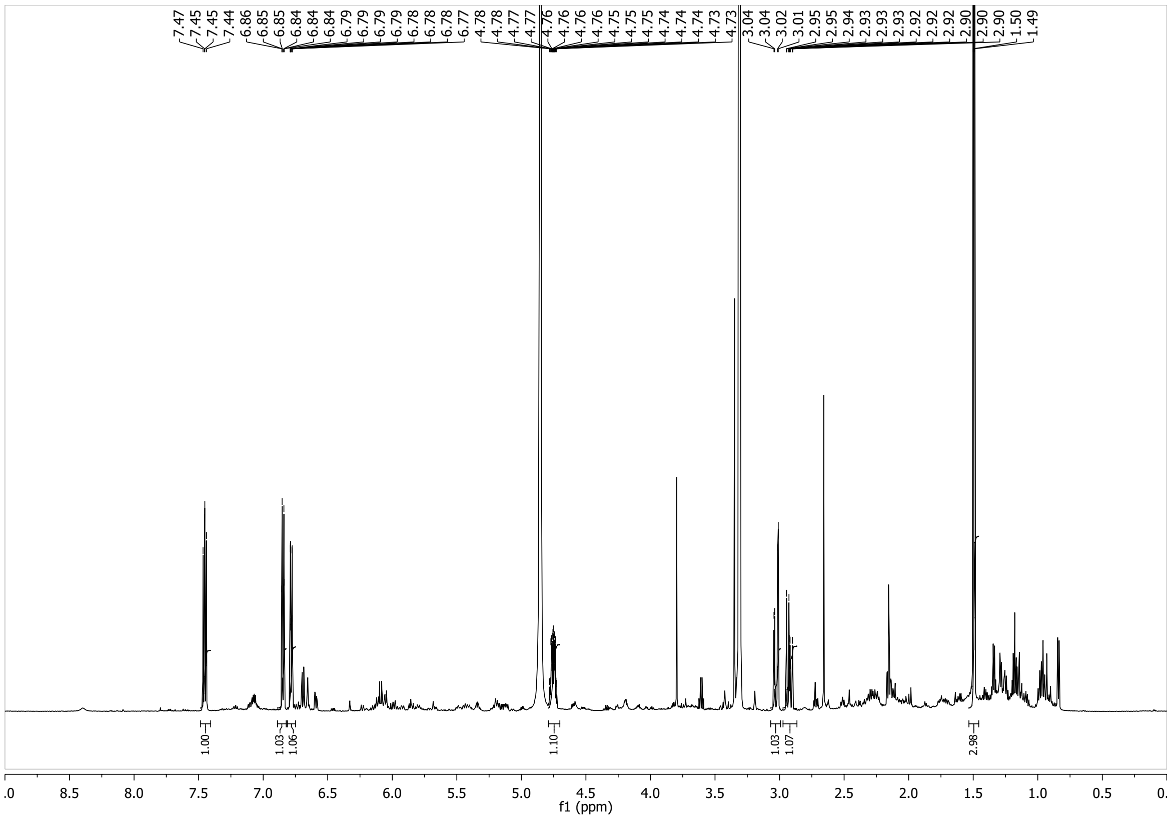

Supplementary Figure 81. ^{1}H NMR spectrum of **13** in MeOD at 600 MHz.

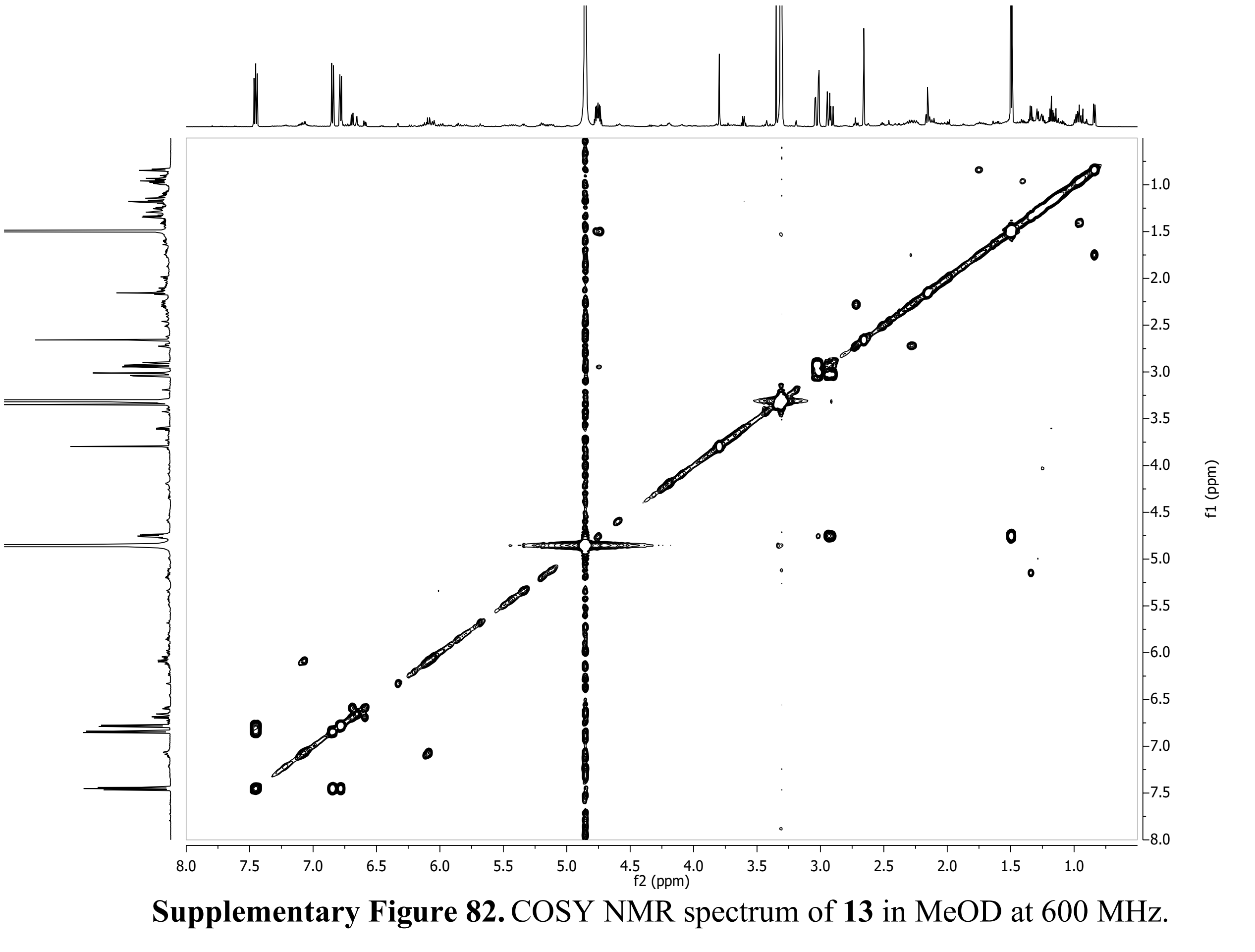

Supplementary Figure 82. COSY NMR spectrum of **13** in MeOD at 600 MHz.

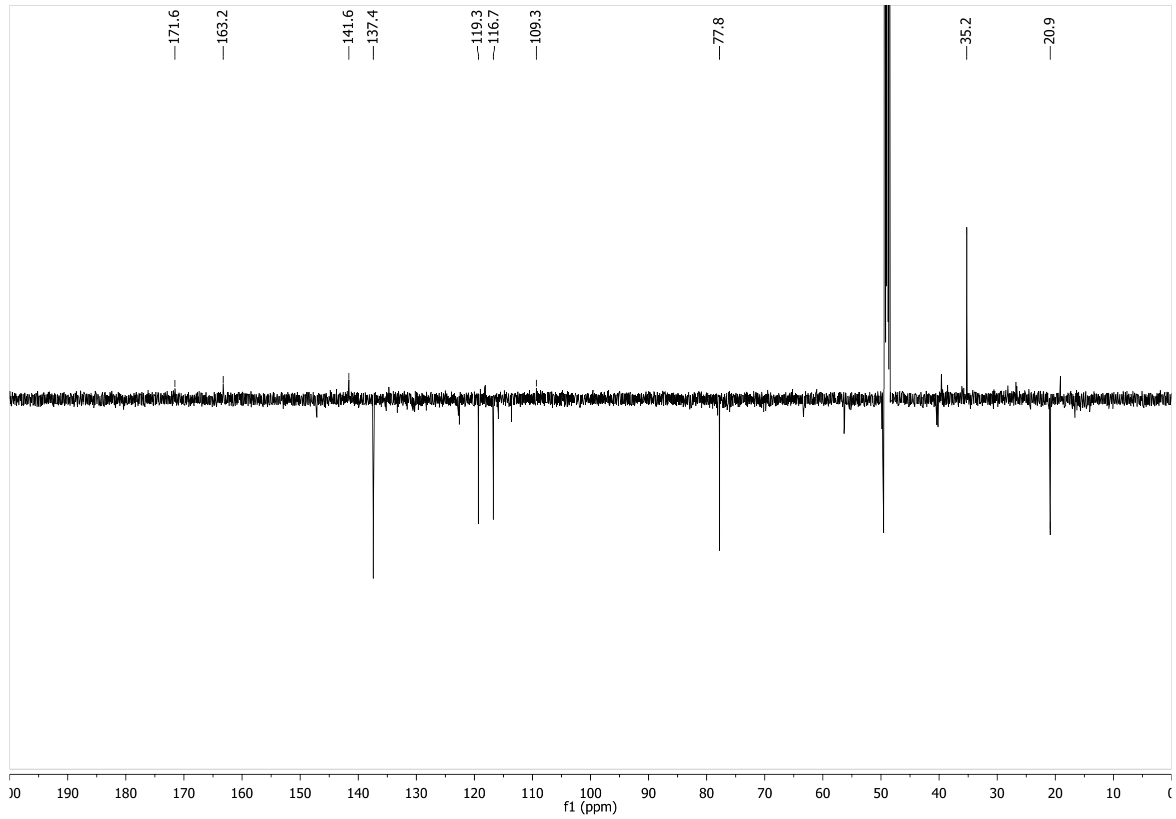

Supplementary Figure 83. ^{1}C NMR spectrum of **13** in MeOD at 600 MHz.

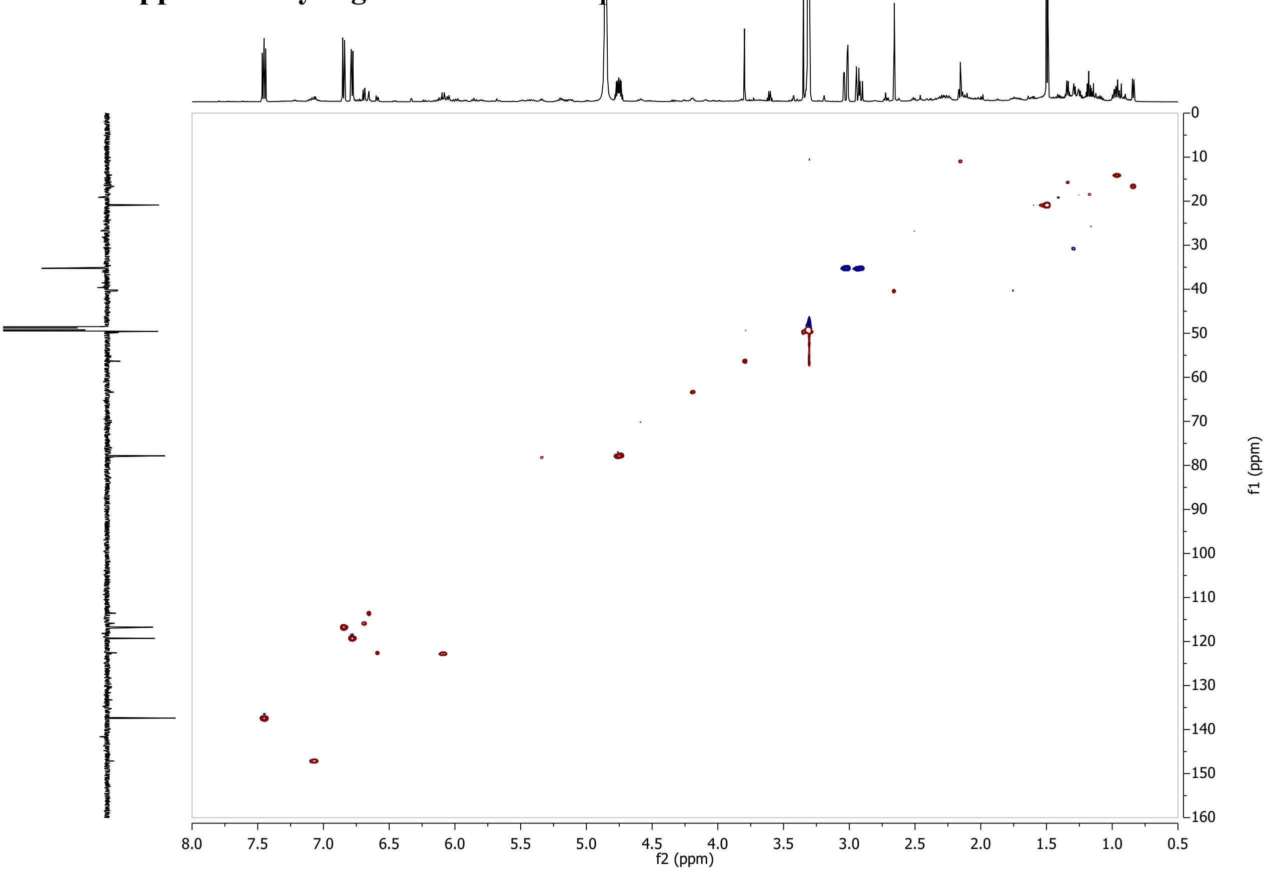

Supplementary Figure 84. HSQC NMR spectrum of **13** in MeOD at 600 MHz.

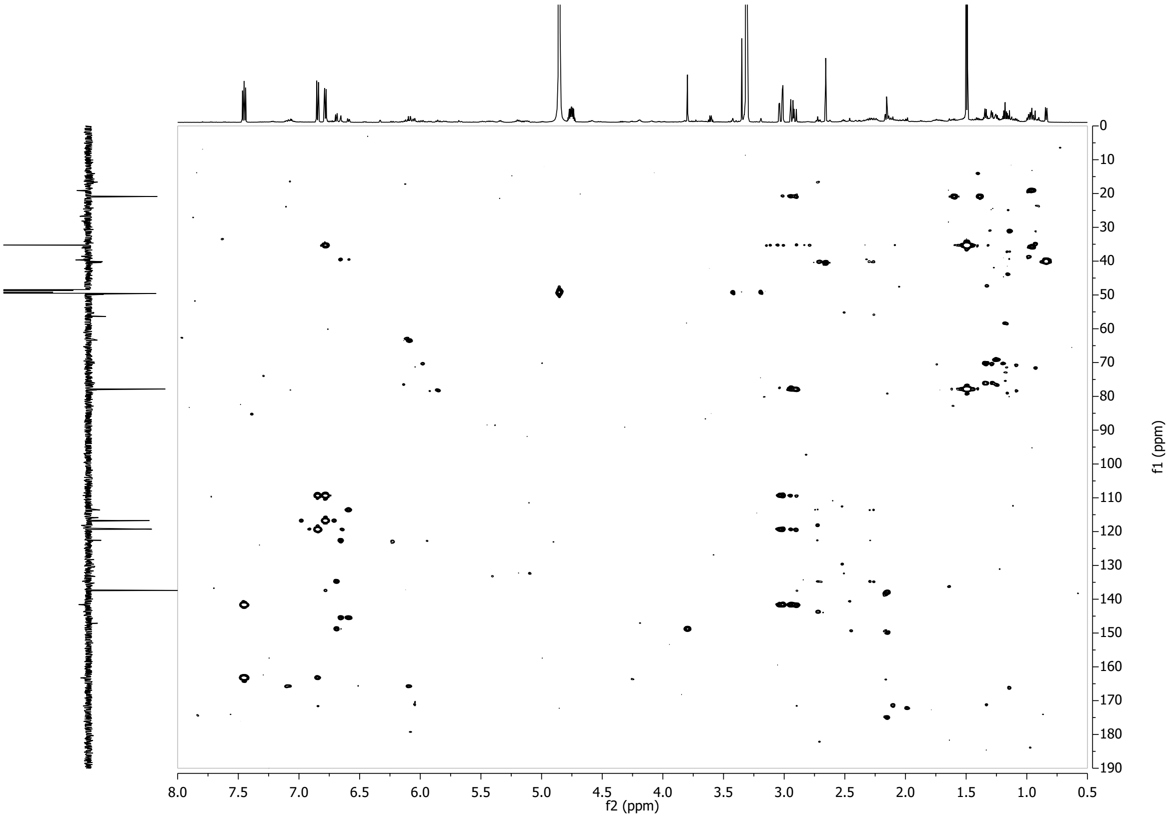

Supplementary Figure 85. HMBC NMR spectrum of **13** in MeOD at 600 MHz.

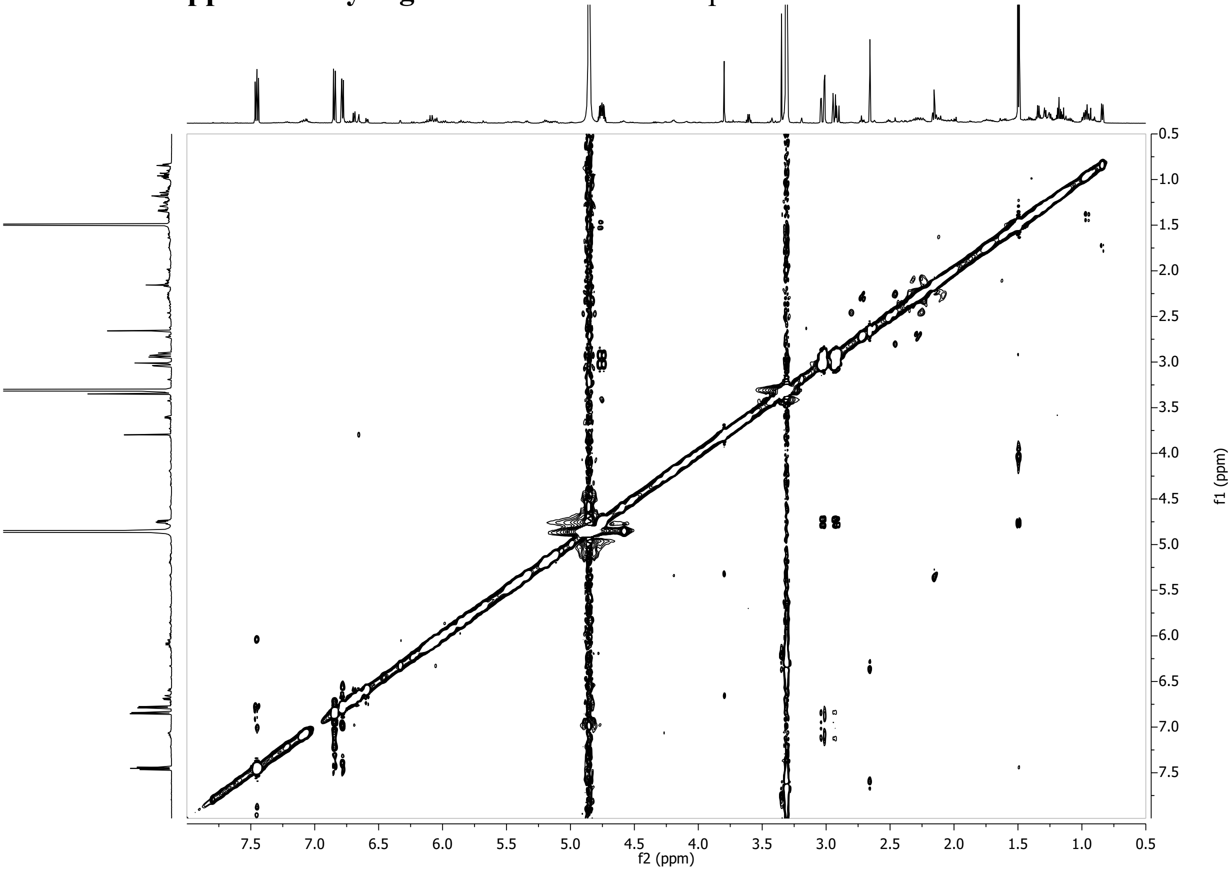

Supplementary Figure 86. ROESY NMR spectrum of **13** in MeOD at 600 MHz.

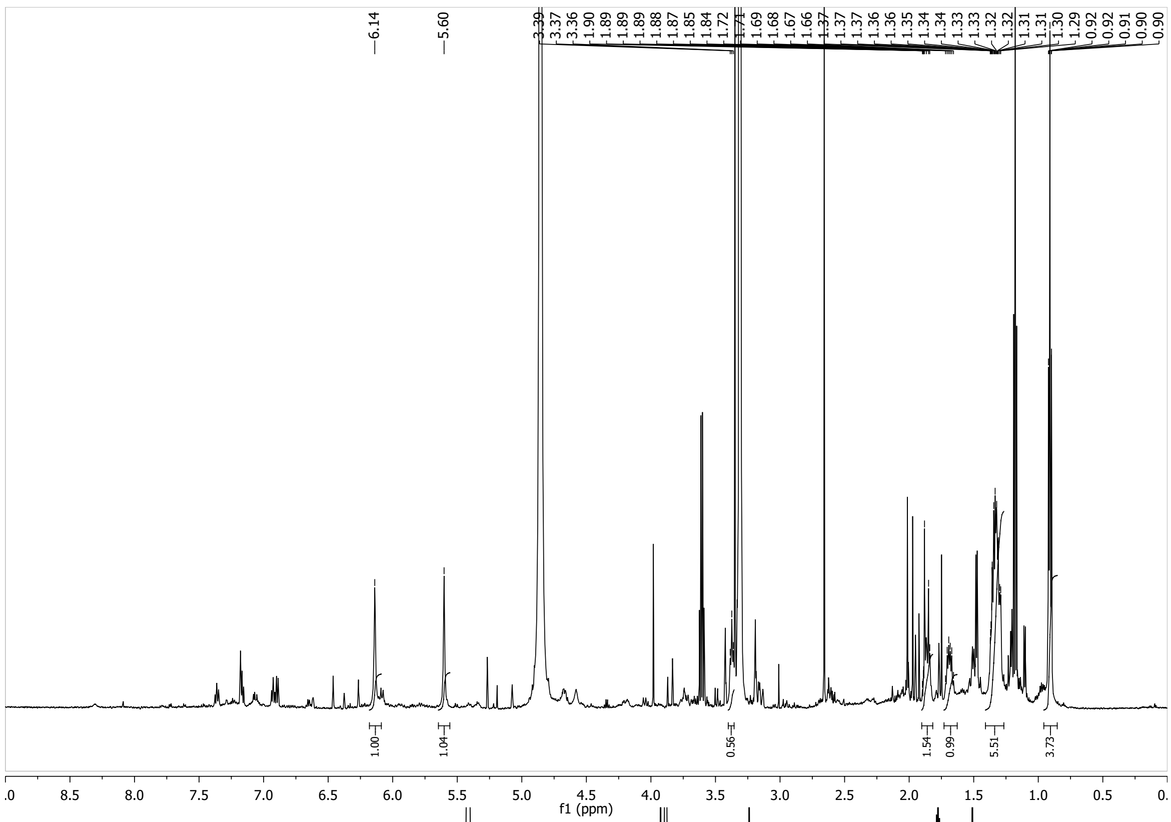

Supplementary Figure 87. ^{1}H NMR spectrum of **14** in MeOD at 600 MHz.

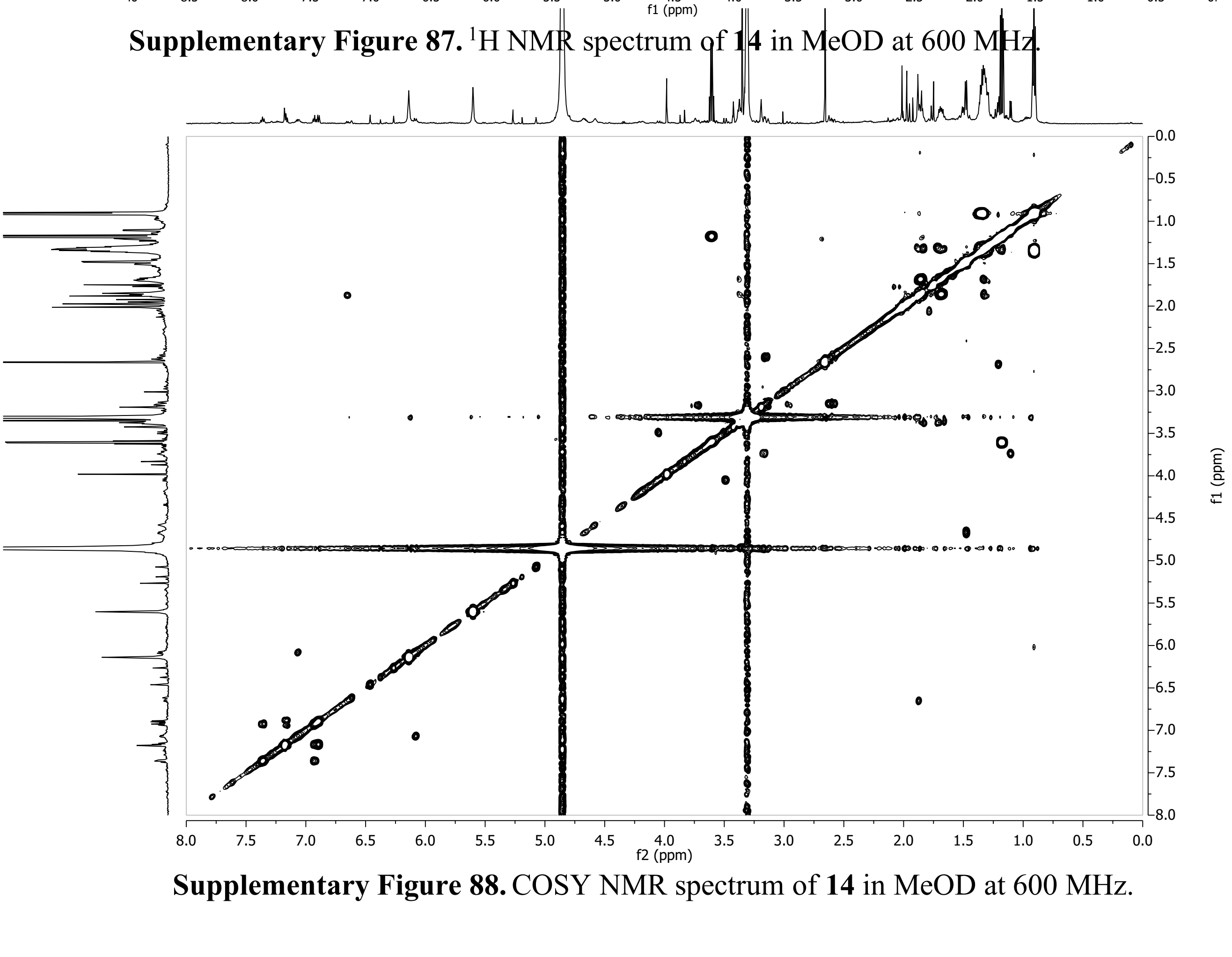

Supplementary Figure 88. COSY NMR spectrum of **14** in MeOD at 600 MHz.

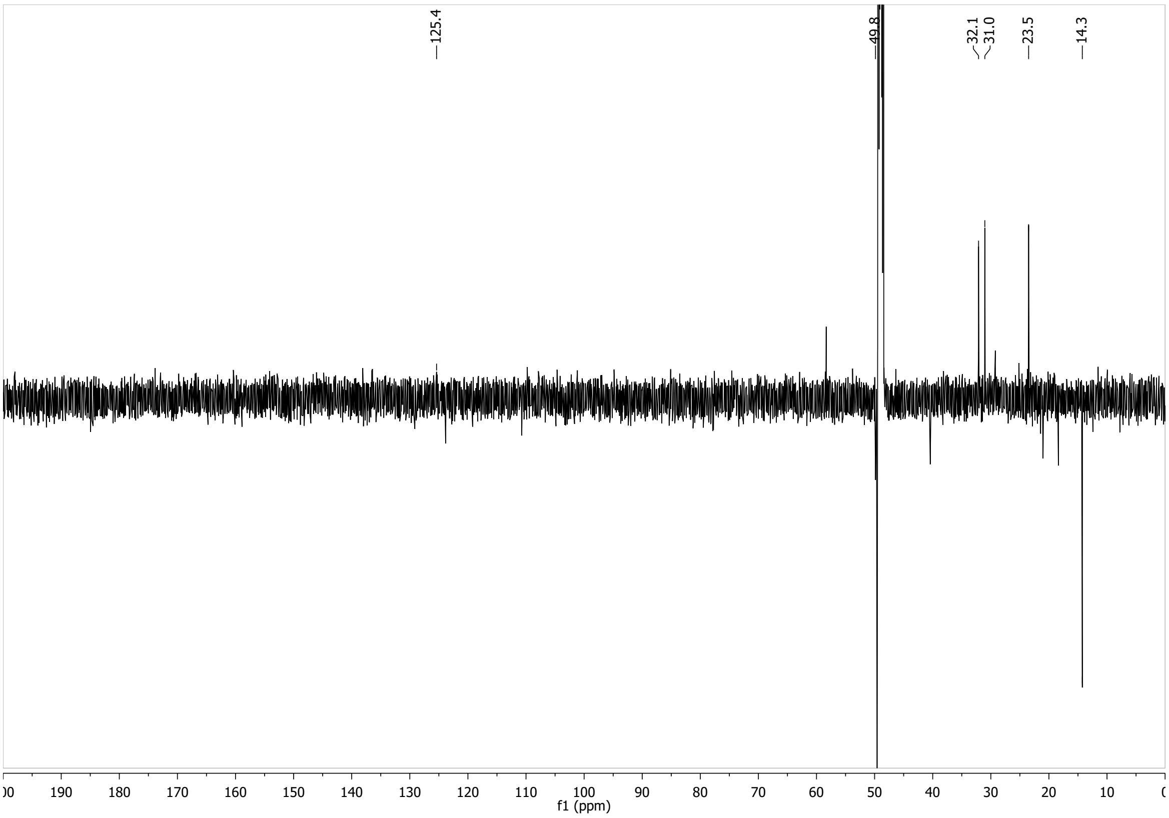

Supplementary Figure 89. [1]C NMR spectrum of **14** in MeOD at 600 MHz.

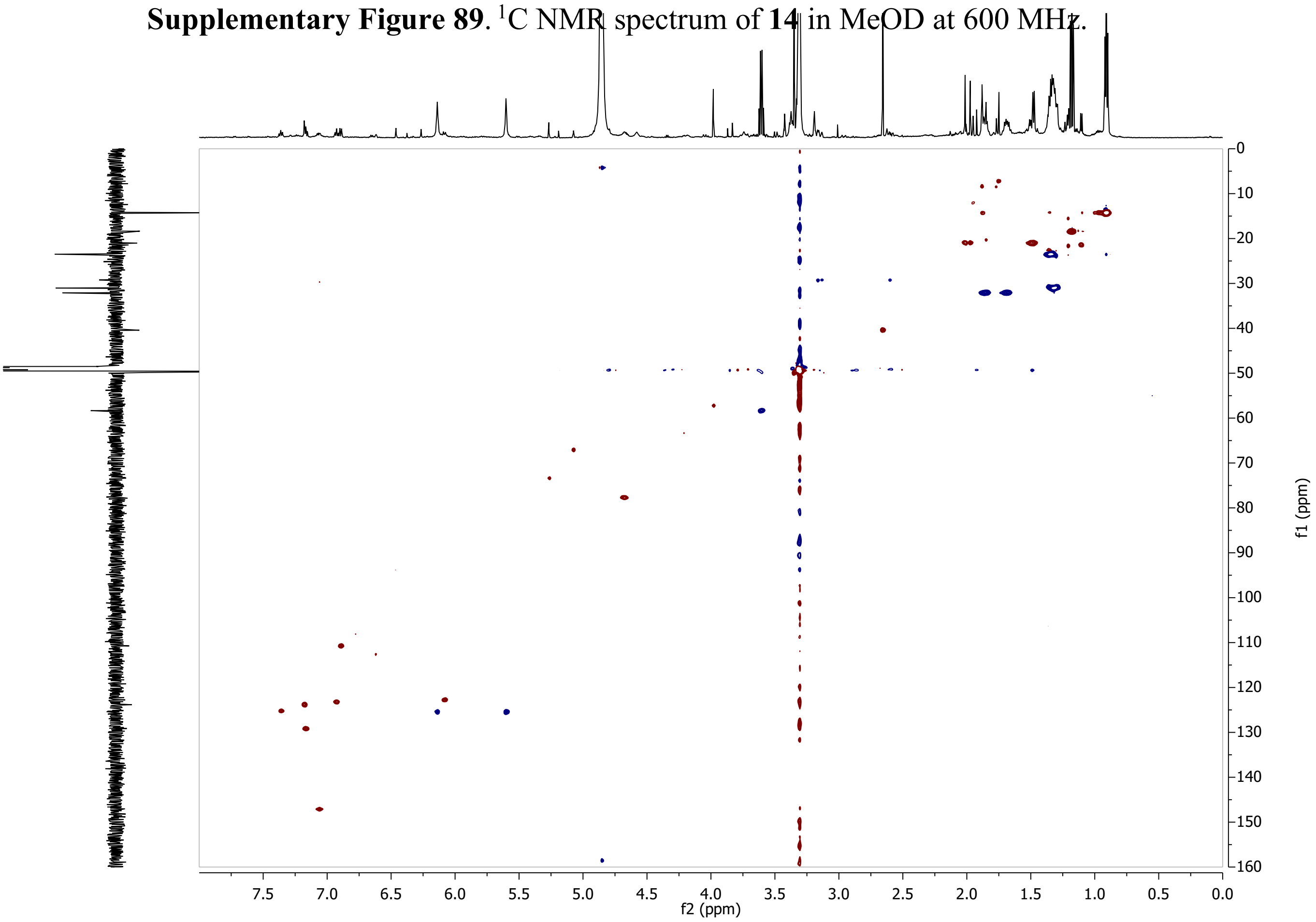

Supplementary Figure 90. HSQC NMR spectrum of **14** in MeOD at 600 MHz.

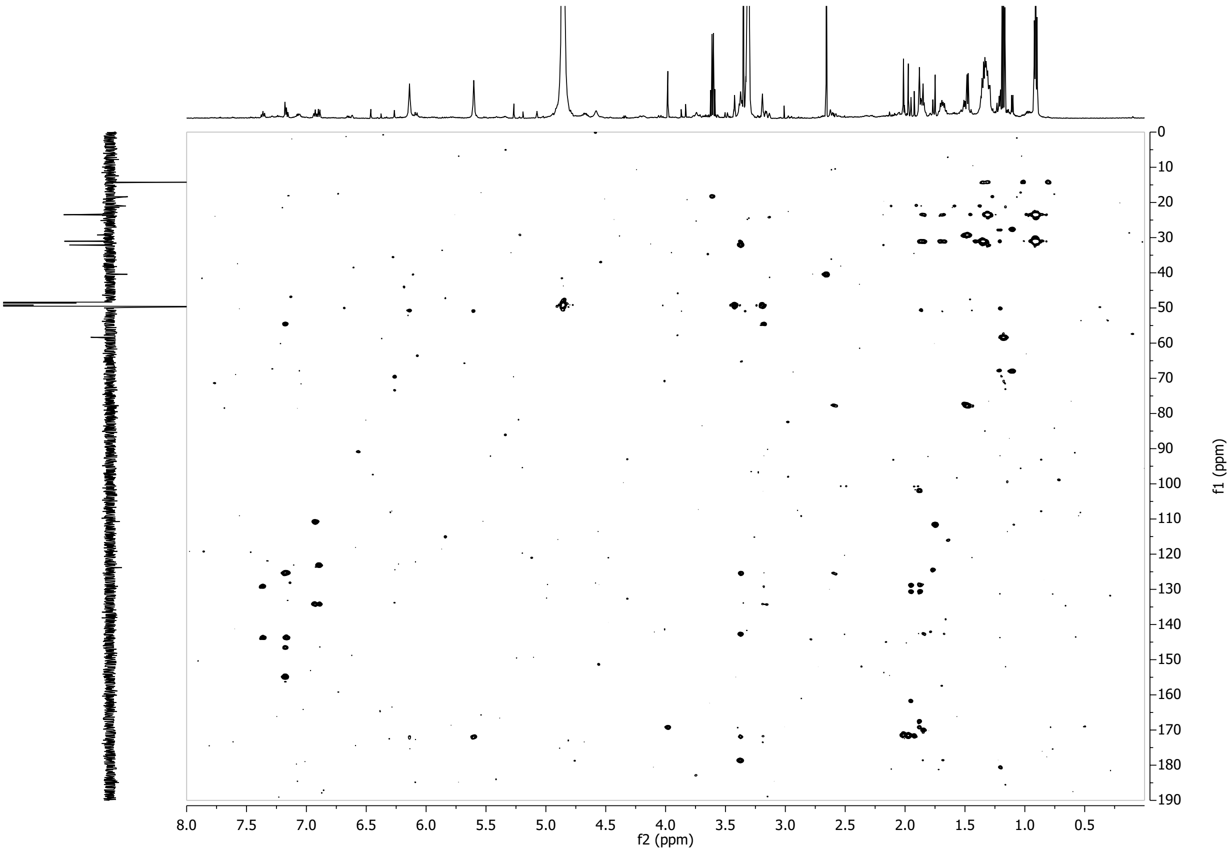

Supplementary Figure 91. HMBC NMR spectrum of **14** in MeOD at 600 MHz.

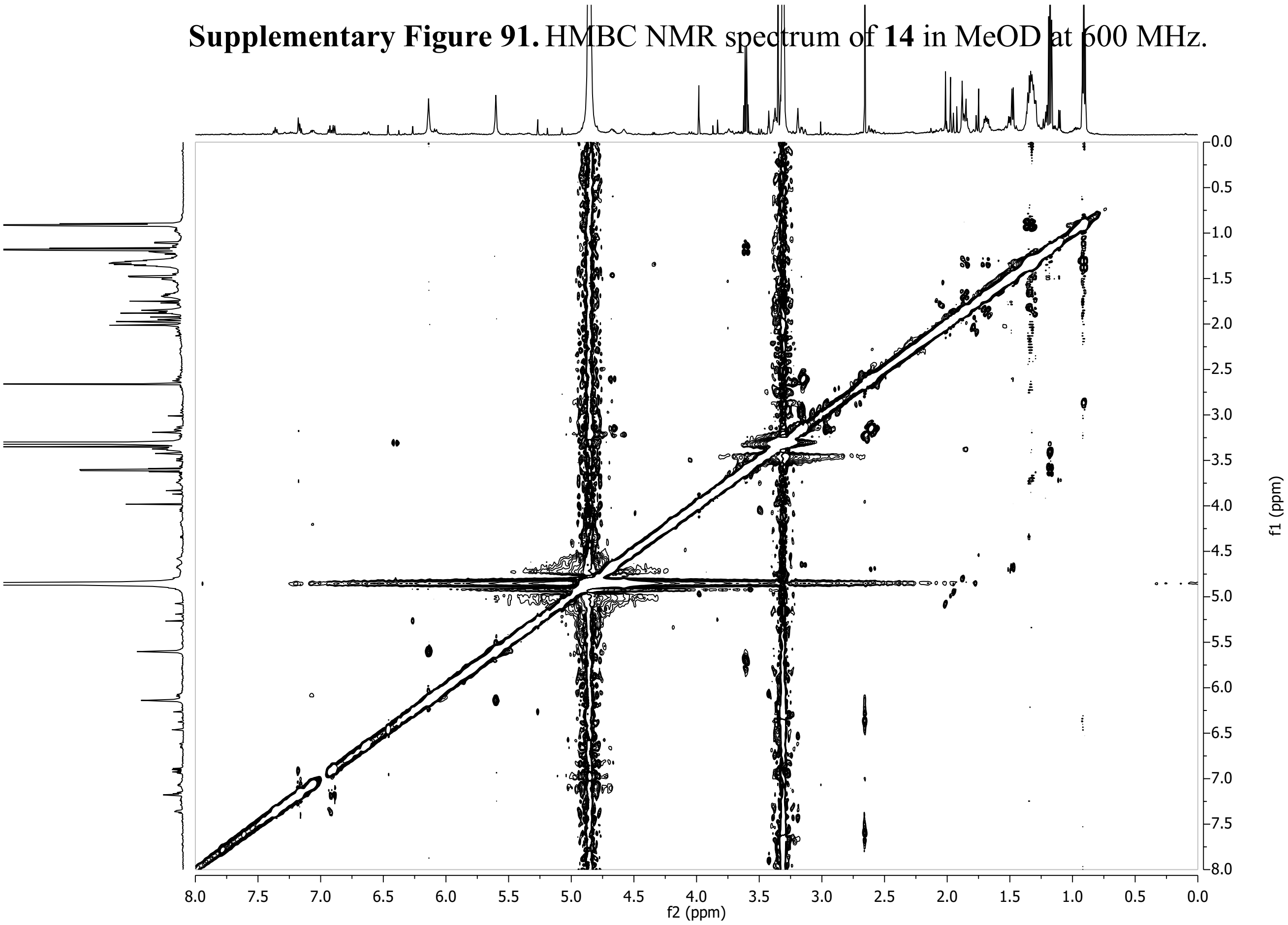

Supplementary Figure 92. ROESY NMR spectrum of **14** in MeOD at 600 MHz.

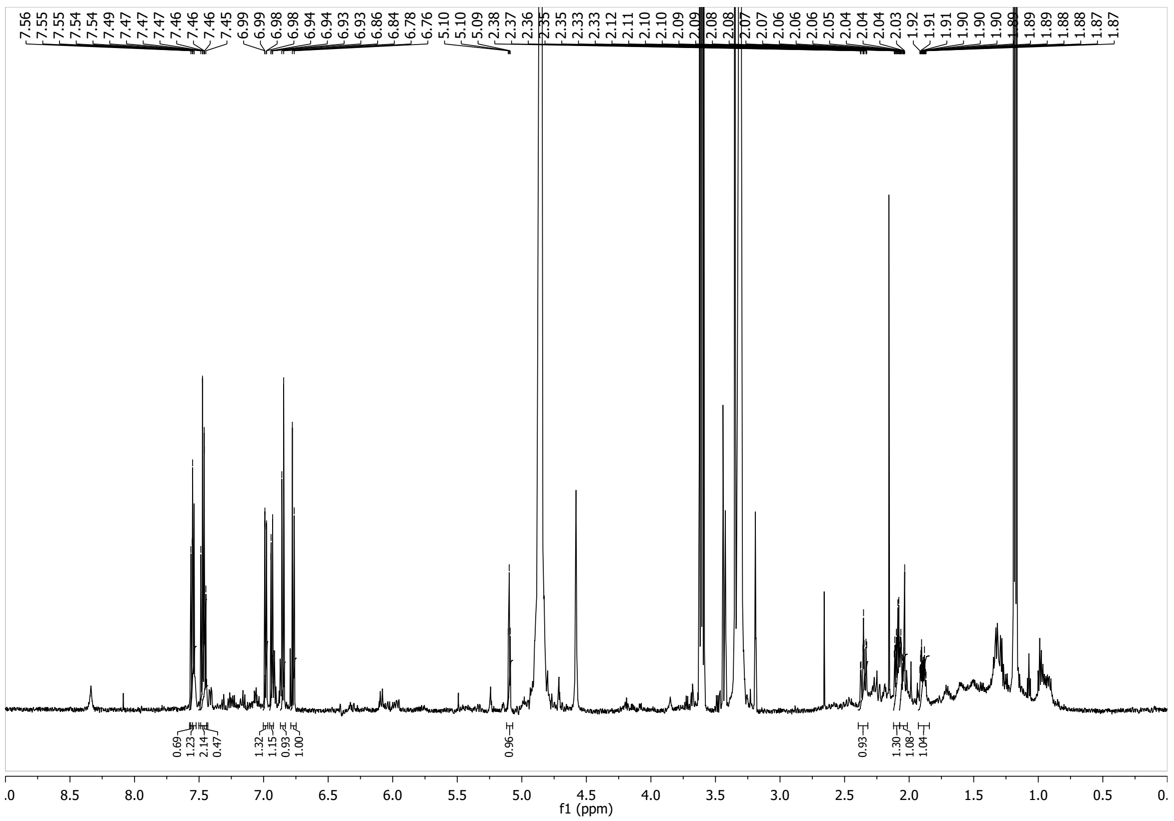

Supplementary Figure 93. ^{1}H NMR spectrum of **15** in MeOD at 600 MHz.

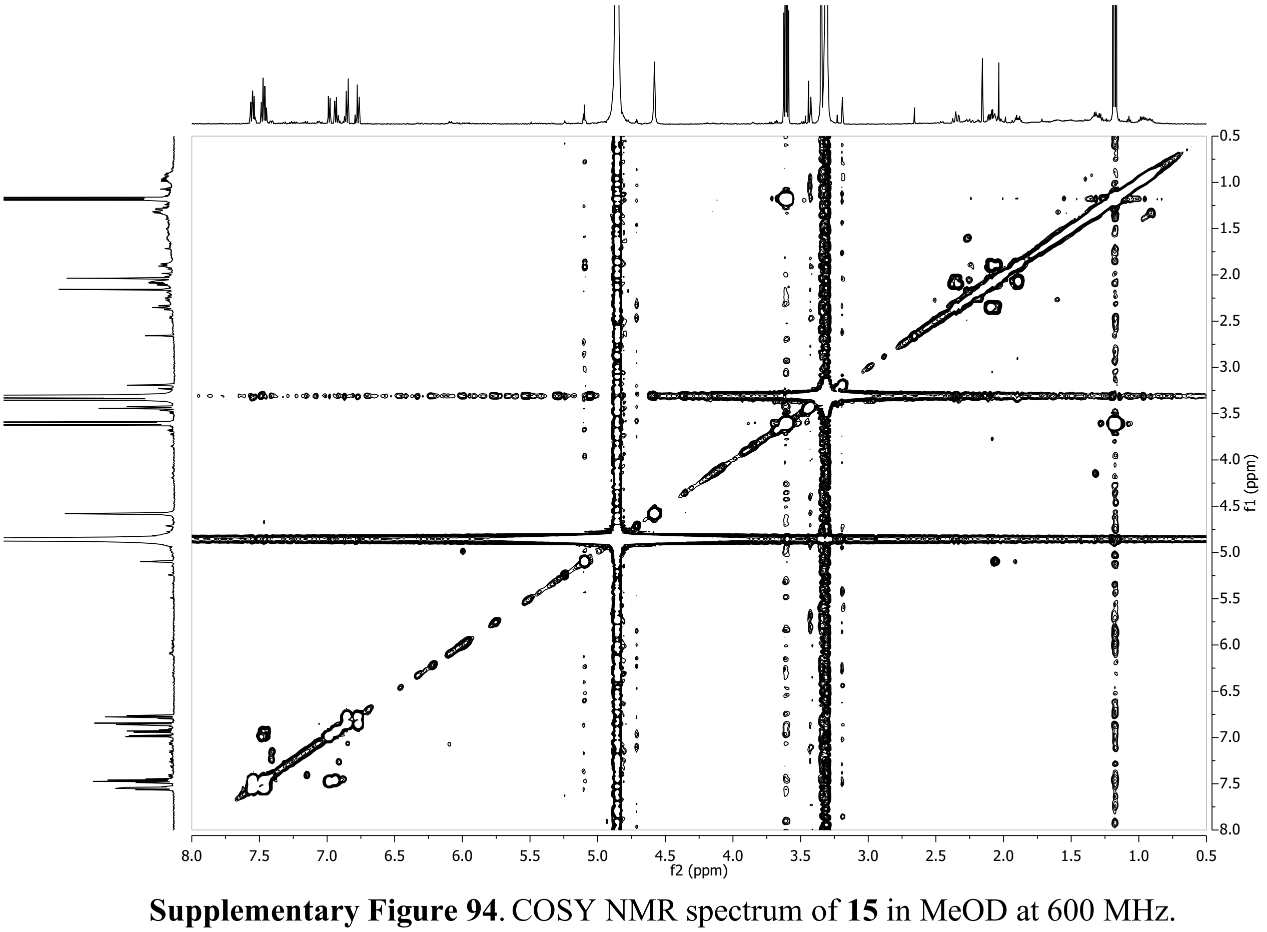

Supplementary Figure 94. COSY NMR spectrum of **15** in MeOD at 600 MHz.

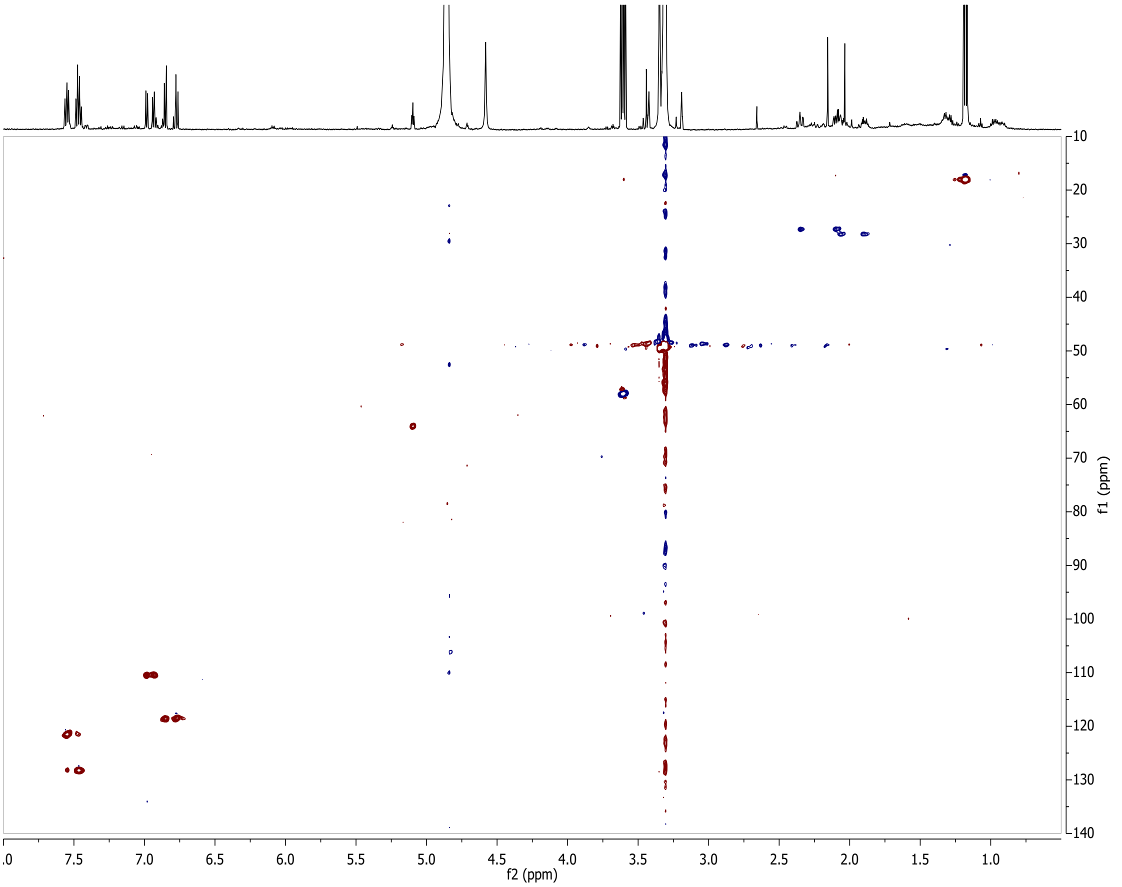

Supplementary Figure 95. HSQC NMR spectrum of **15** in MeOD at 600 MHz.

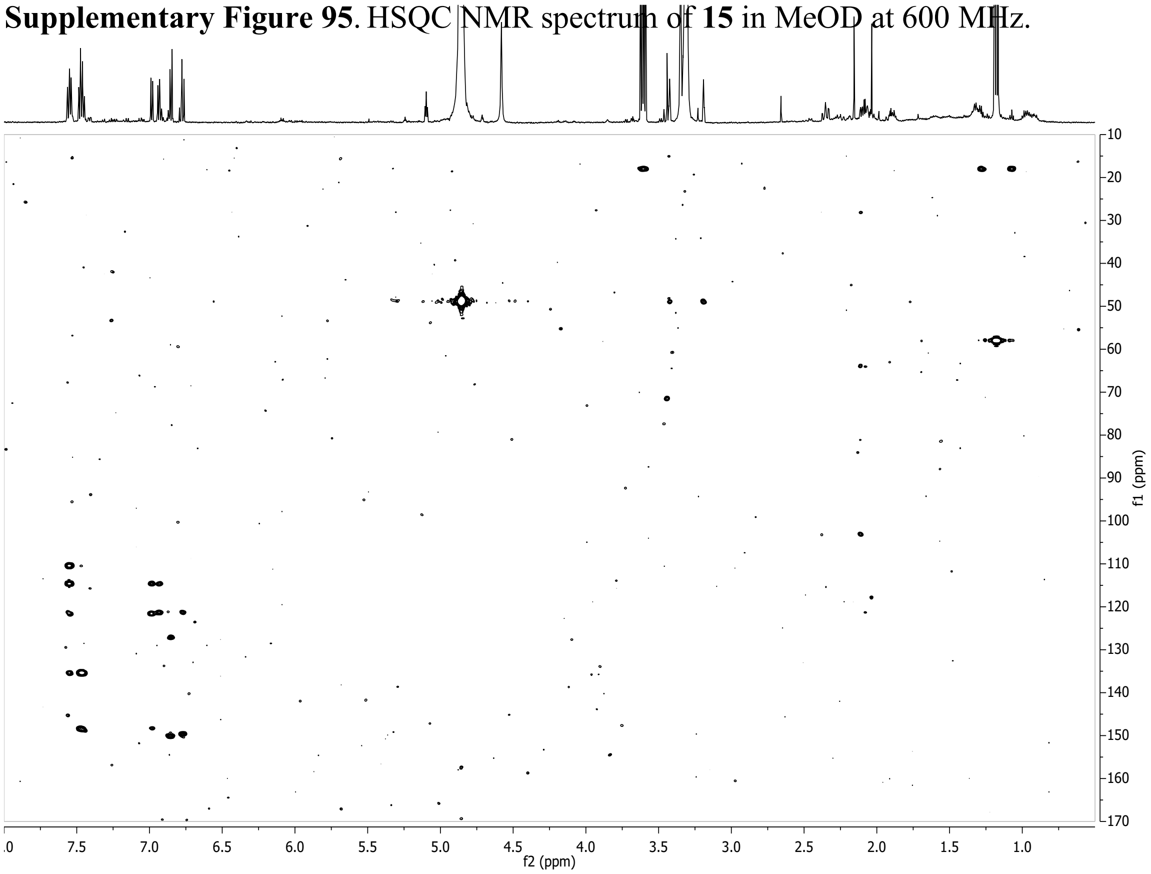

Supplementary Figure 96. HMBC NMR spectrum of **15** in MeOD at 600 MHz.

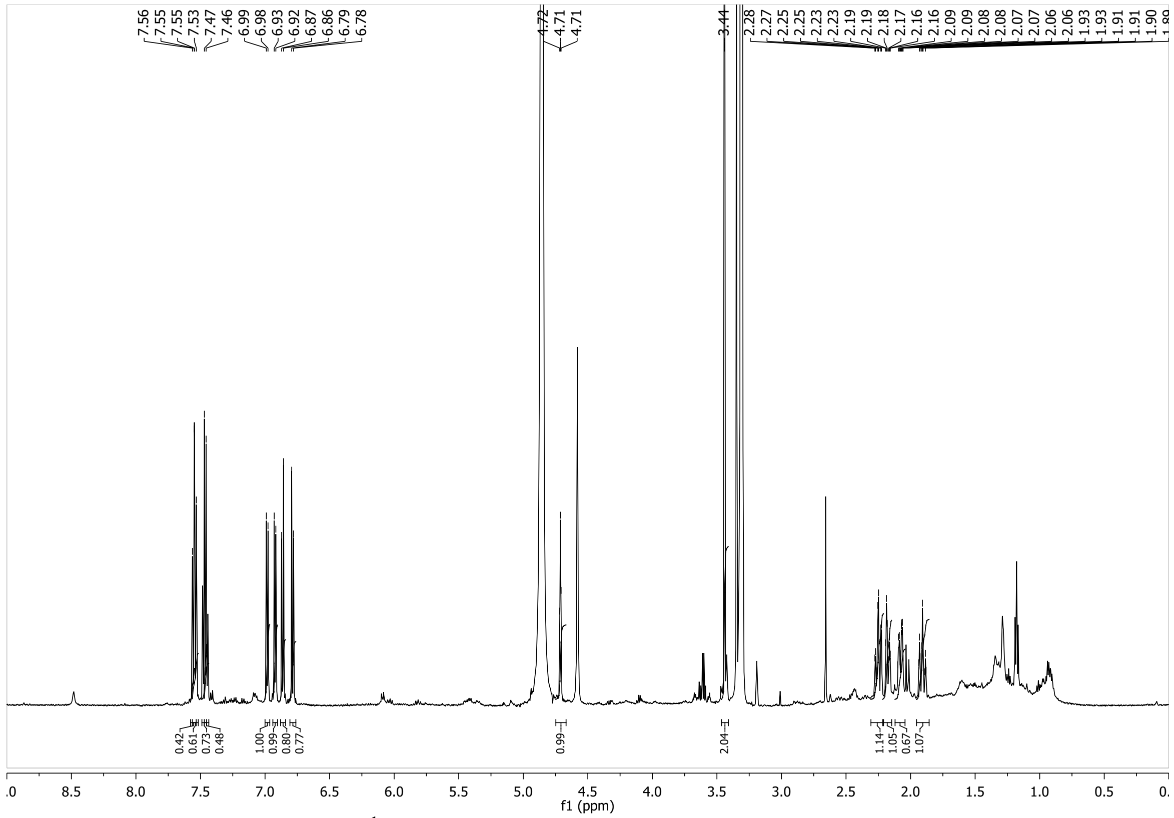

Supplementary Figure 97. ^{1}H NMR spectrum of **16** in MeOD at 600 MHz.

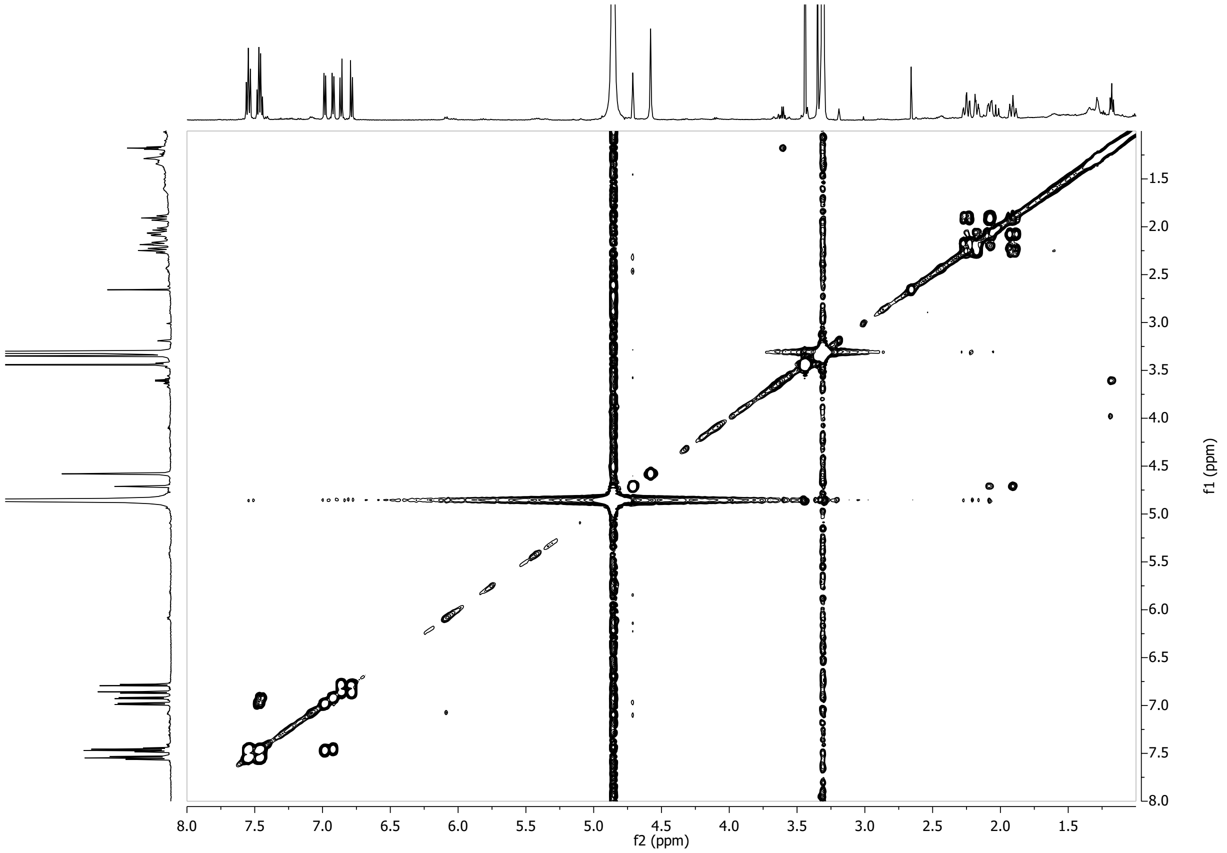

Supplementary Figure 98. COSY NMR spectrum of **16** in MeOD at 600 MHz.

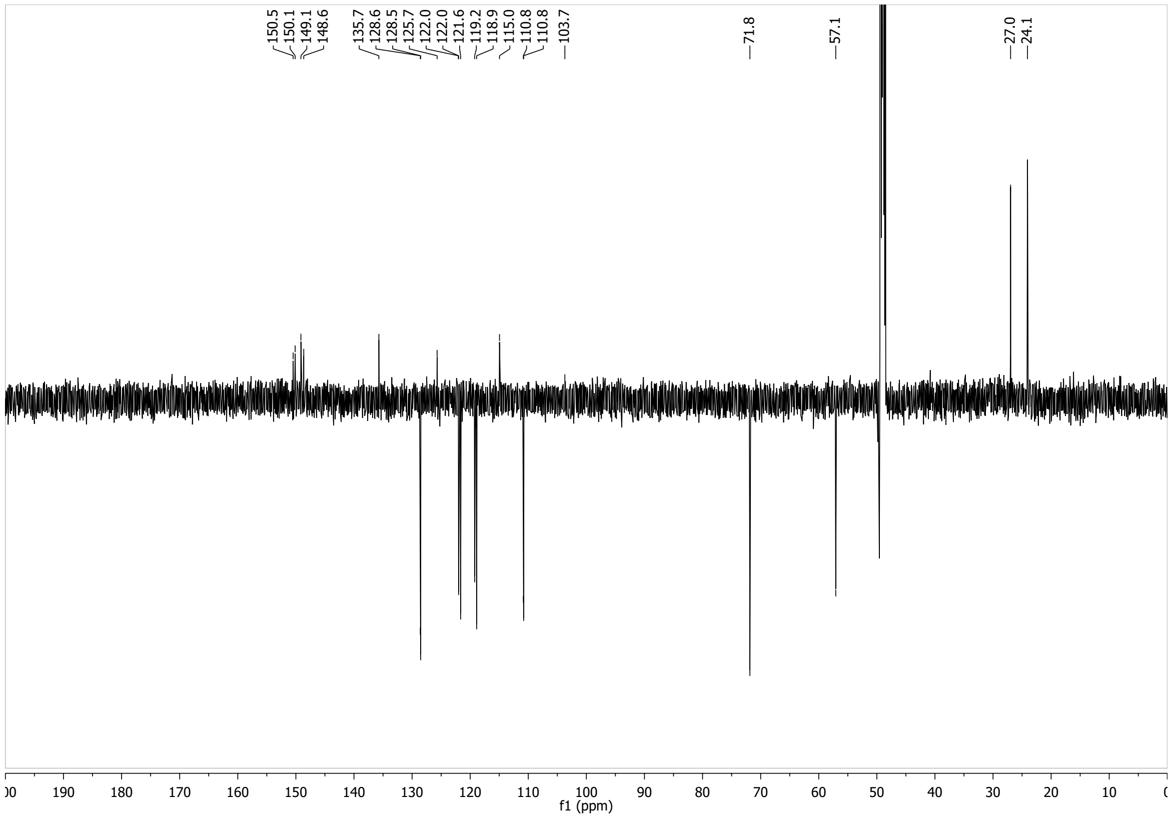

Supplementary Figure 99. [1]C NMR spectrum of **16** in MeOD at 600 MHz.

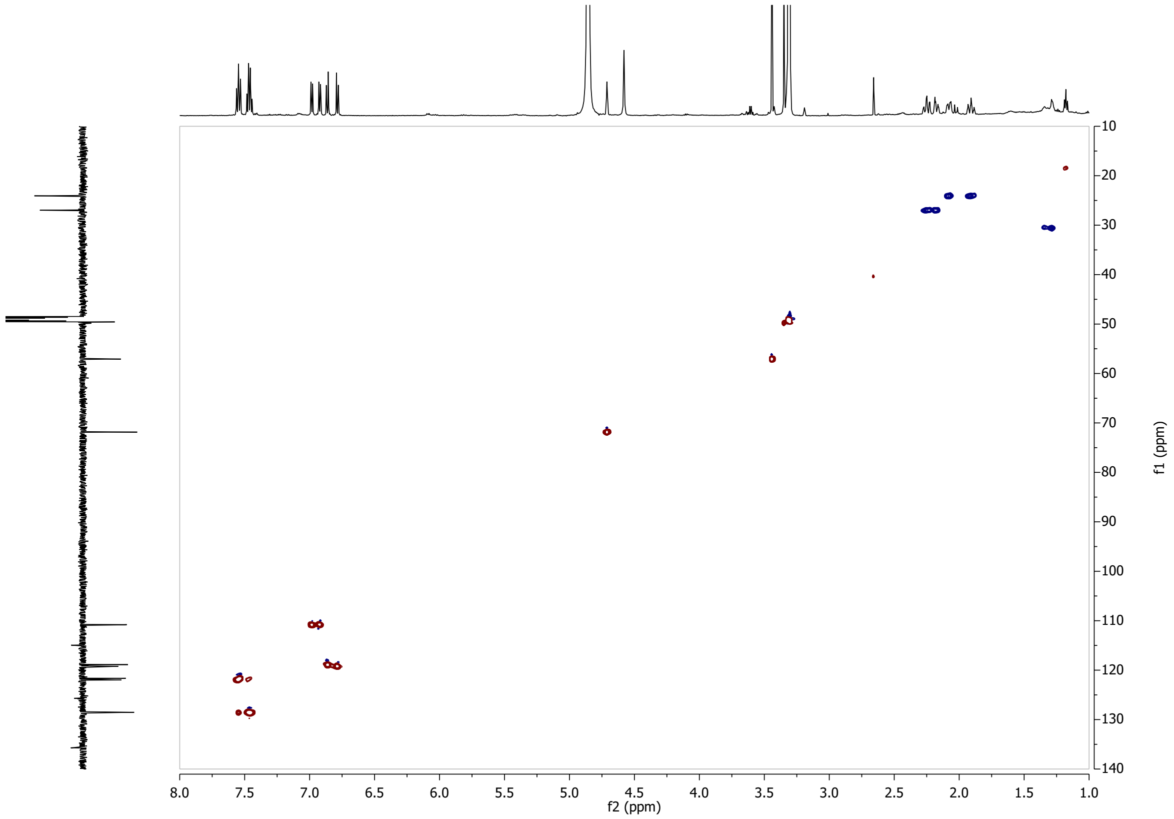

Supplementary Figure 100. HSQC NMR spectrum of **16** in MeOD at 600 MHz.

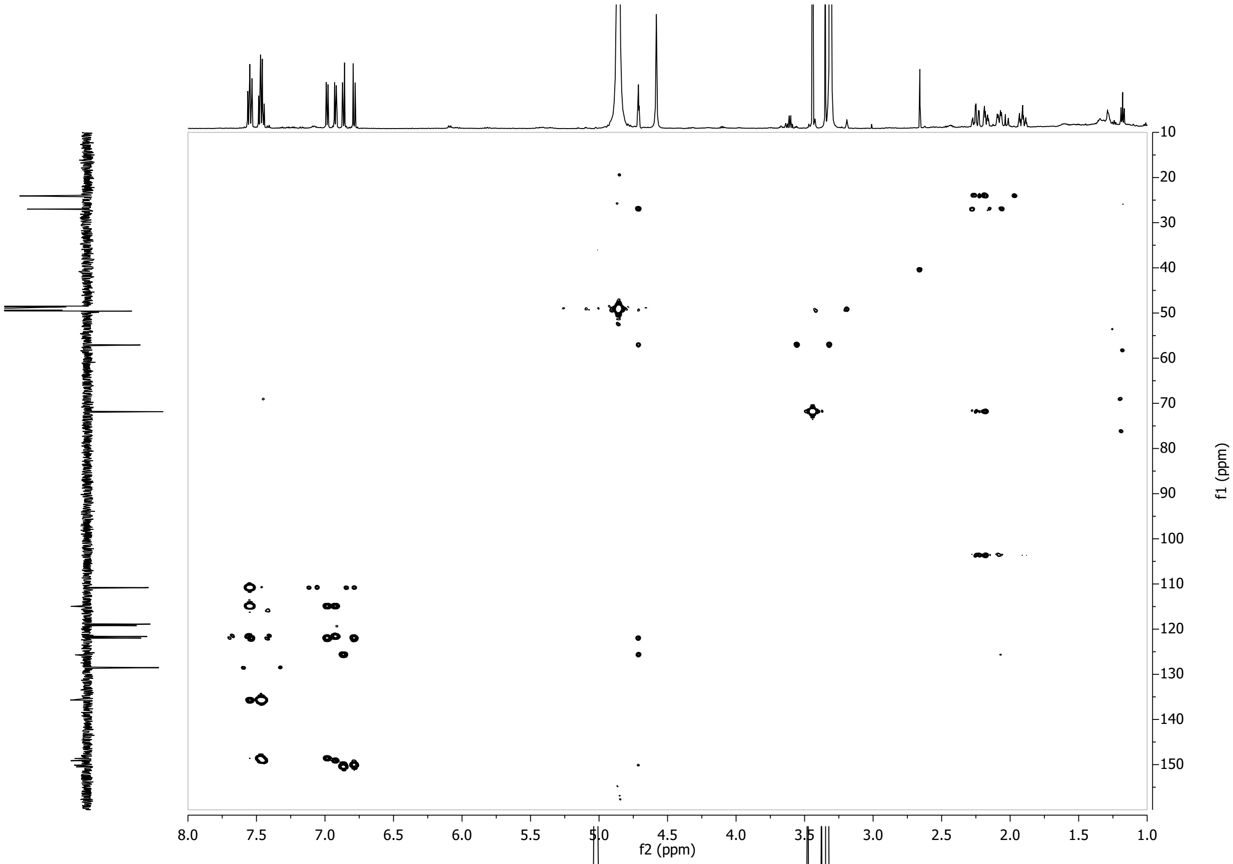

Supplementary Figure 101. HMBC NMR spectrum of **16** in MeOD at 600 MHz.

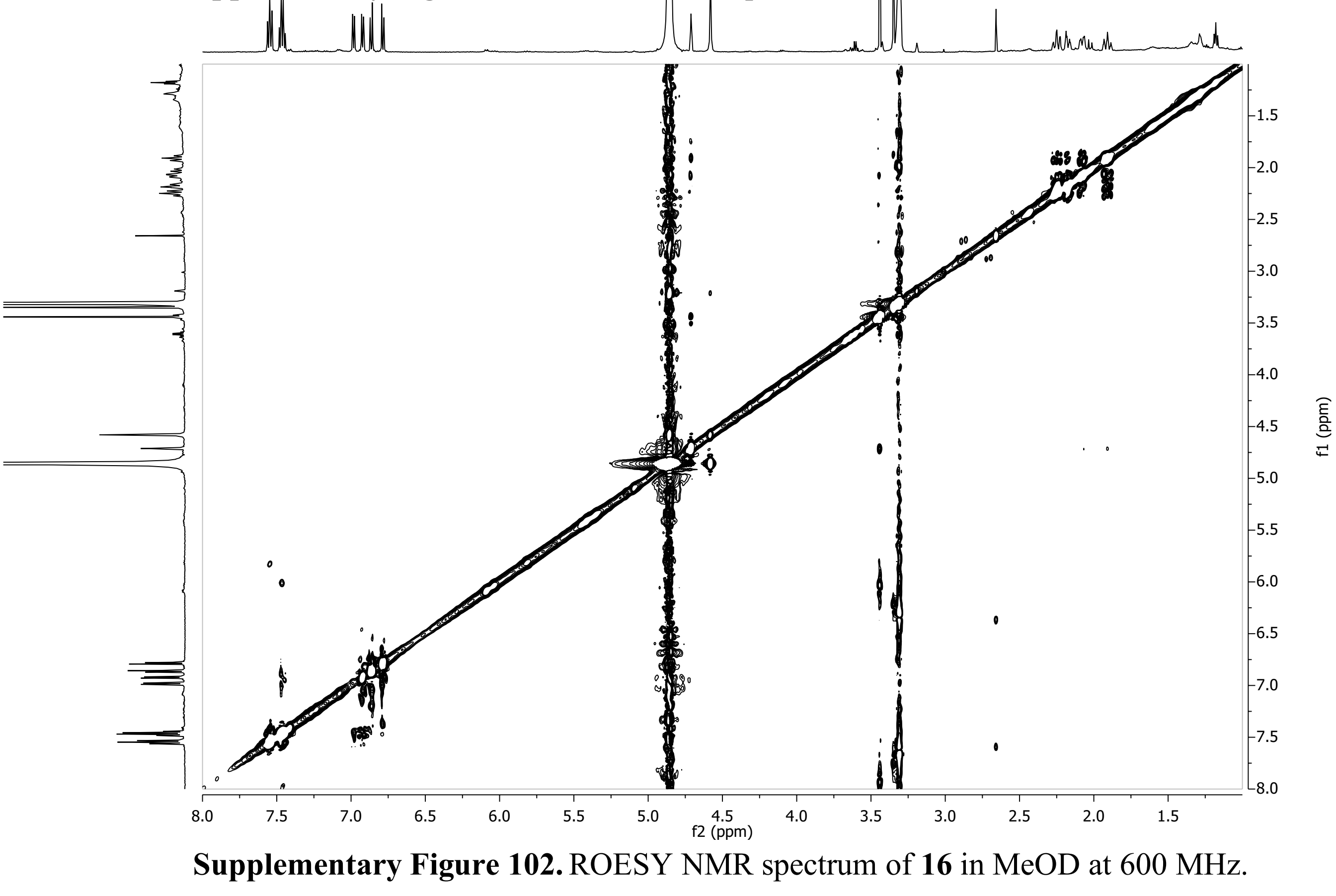

Supplementary Figure 102. ROESY NMR spectrum of **16** in MeOD at 600 MHz.

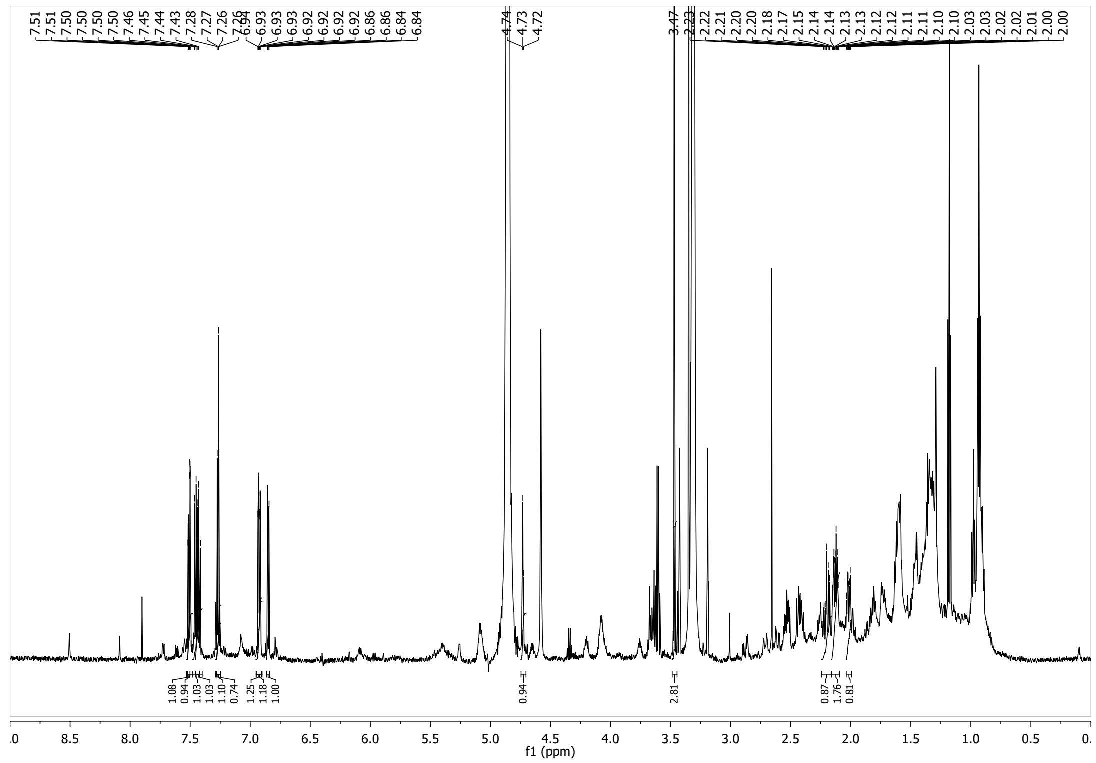

Supplementary Figure 103. ^{1}H NMR spectrum of **17** in MeOD at 600 MHz.

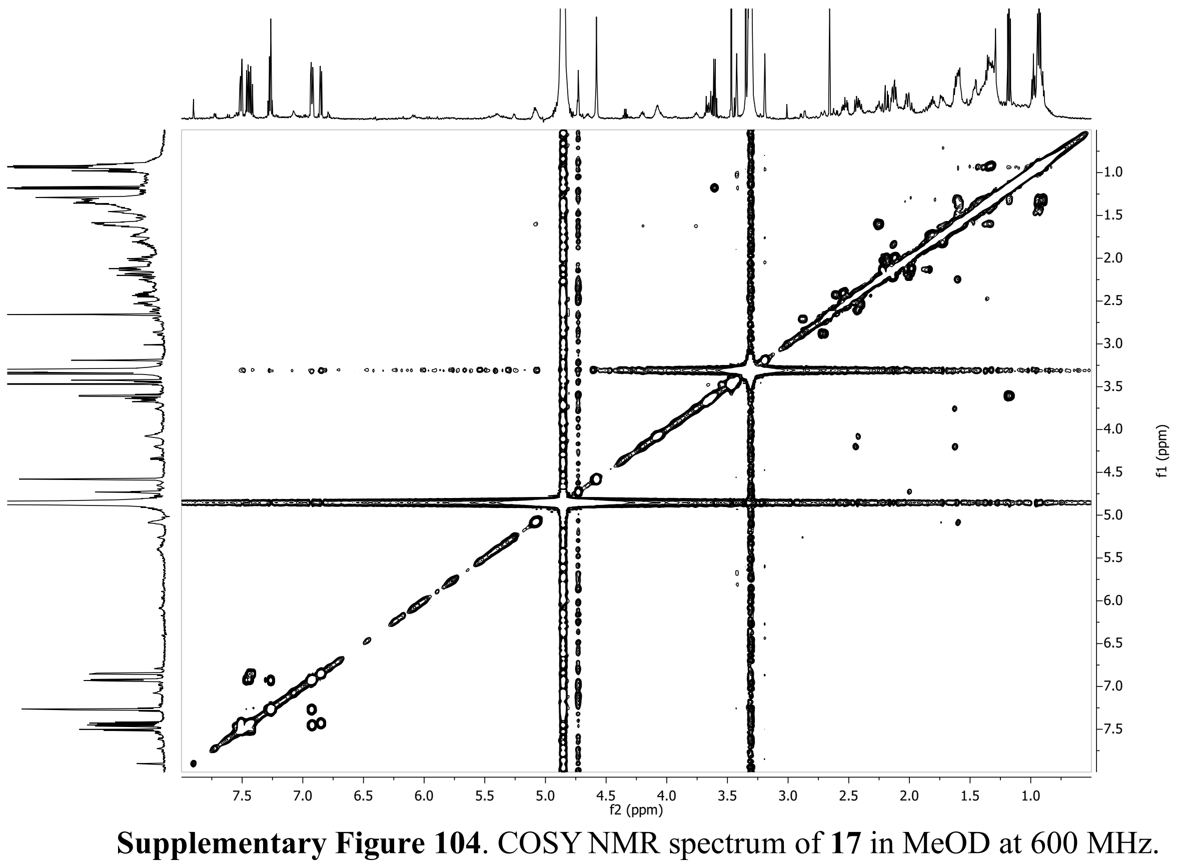

Supplementary Figure 104. COSY NMR spectrum of **17** in MeOD at 600 MHz.

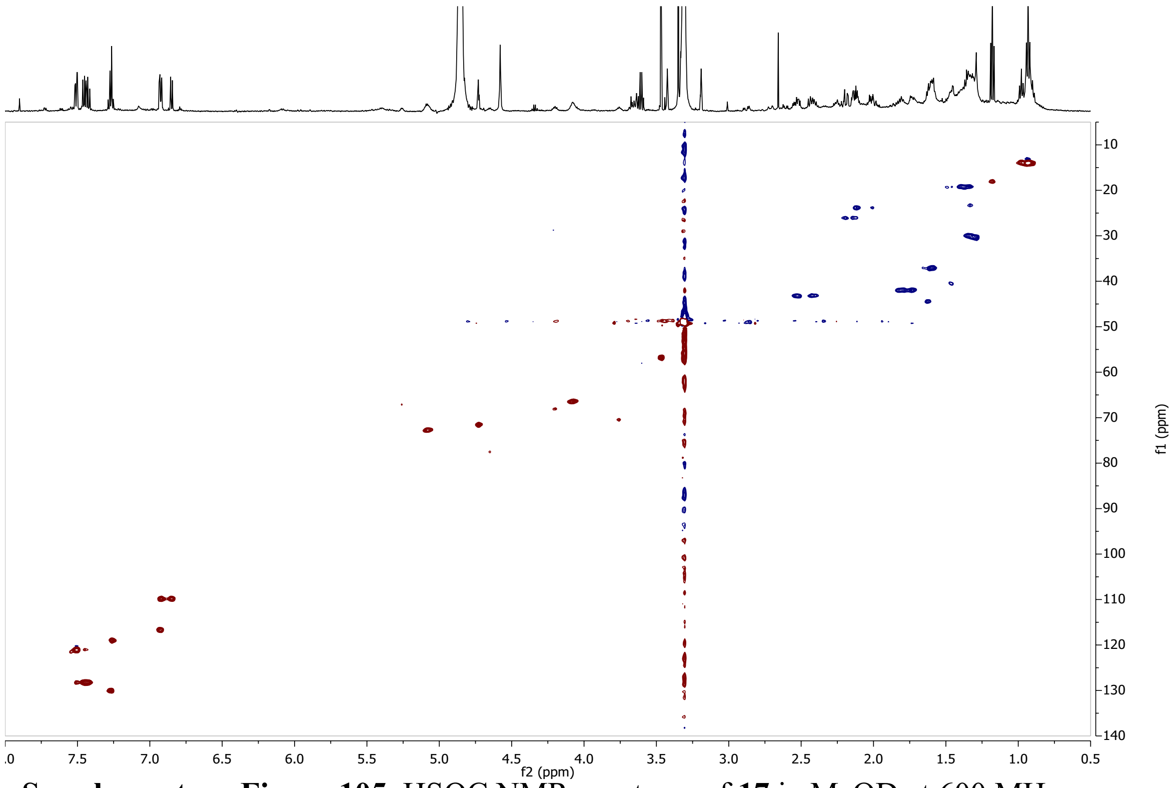

Supplementary Figure 105. HSQC NMR spectrum of **17** in MeOD at 600 MHz.

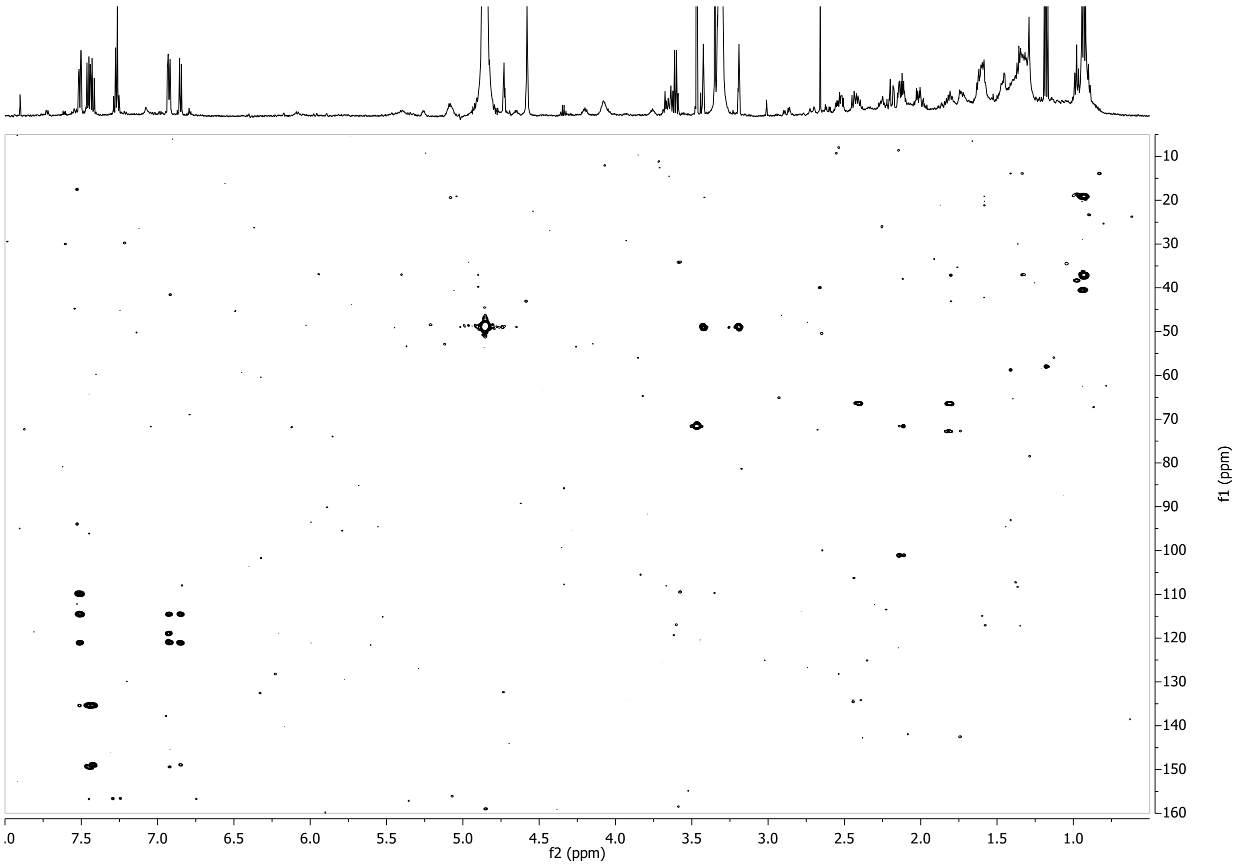

Supplementary Figure 106. HMBC NMR spectrum of **17** in MeOD at 600 MHz.

Molecular networking

Taxo PHYLUM | Taxo Class | Taxo Order | Taxo family | Taxo genus | Manually annotated | Isolated

Features coming from A02 EtM Fractions

Features coming from A02 EtM extract

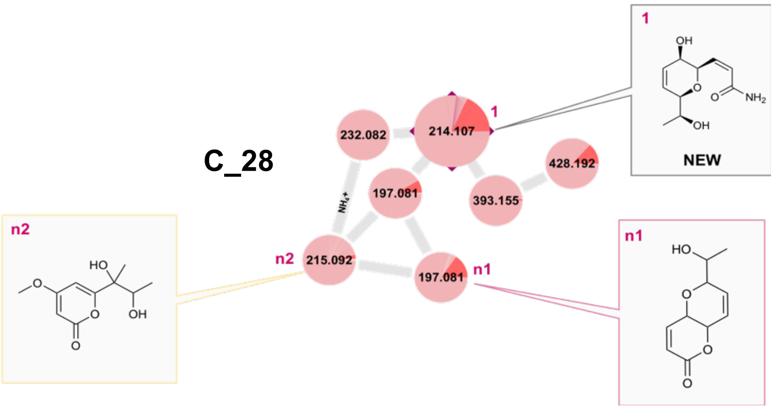

Supplementary Figure 107. Cluster C_28

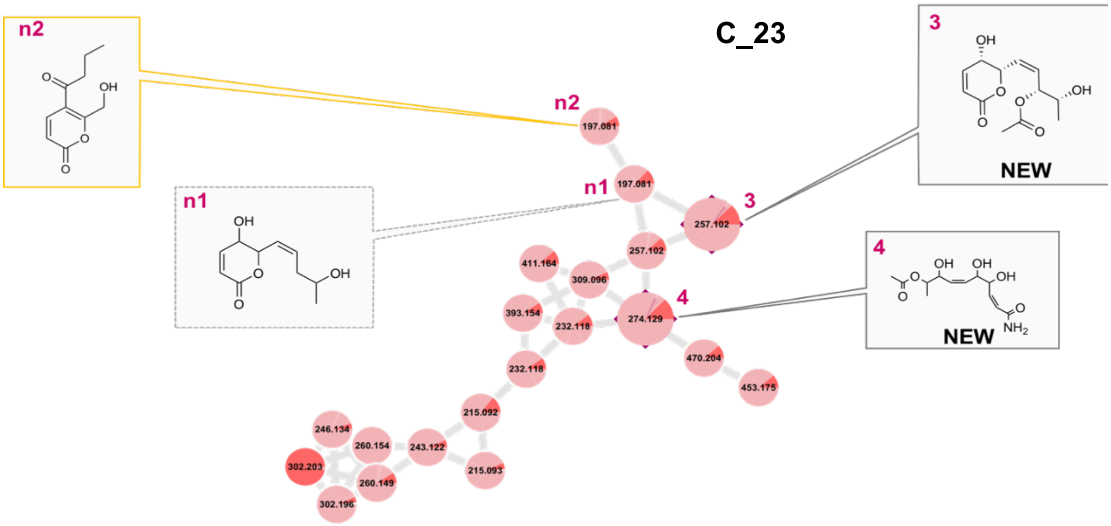

Supplementary Figure 108. Cluster C_23

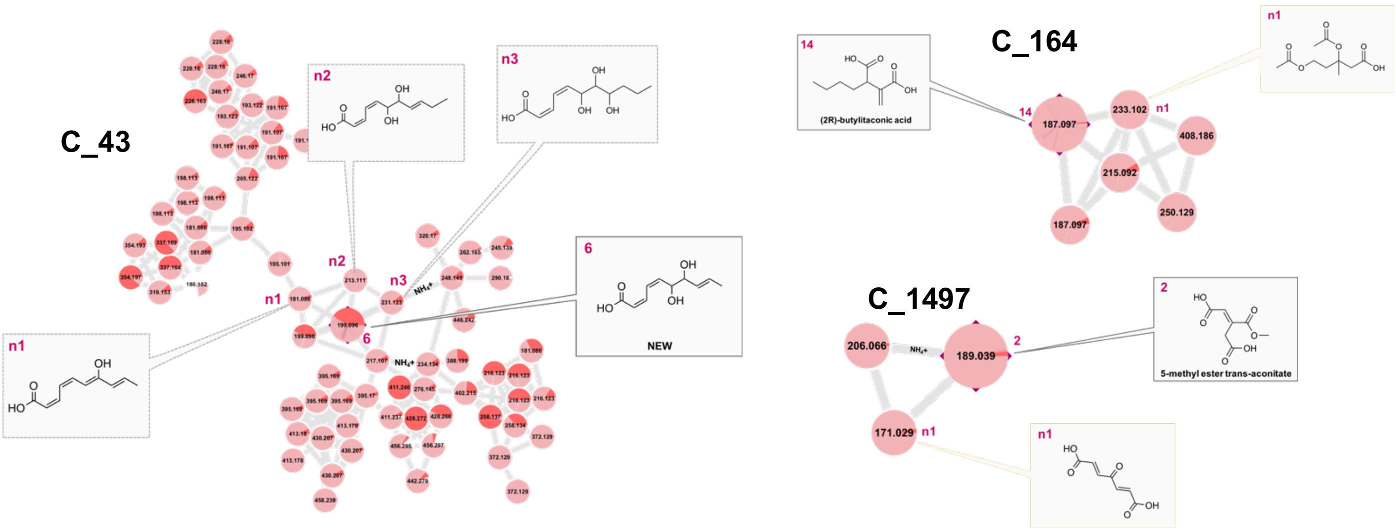

Supplementary Figure 109. Clusters C_43, C_164, C_1497

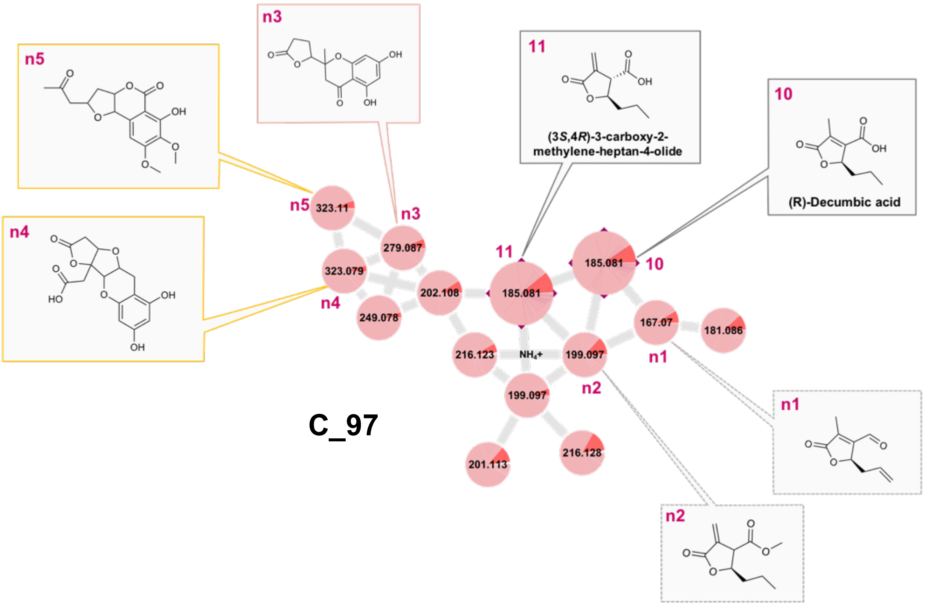

Supplementary Figure 110. Clusters C_97

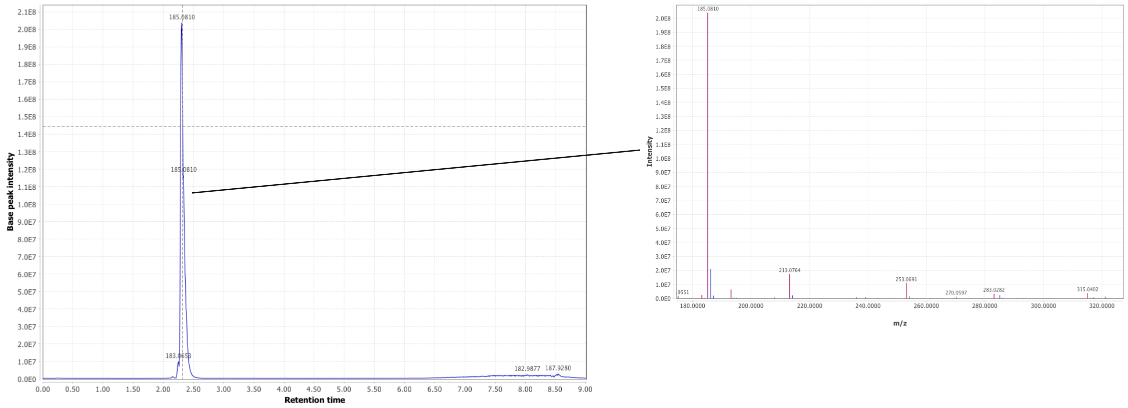

Supplementary Figure 111. UHPLC-HRMS-ESI (-) analysis of compound **12**

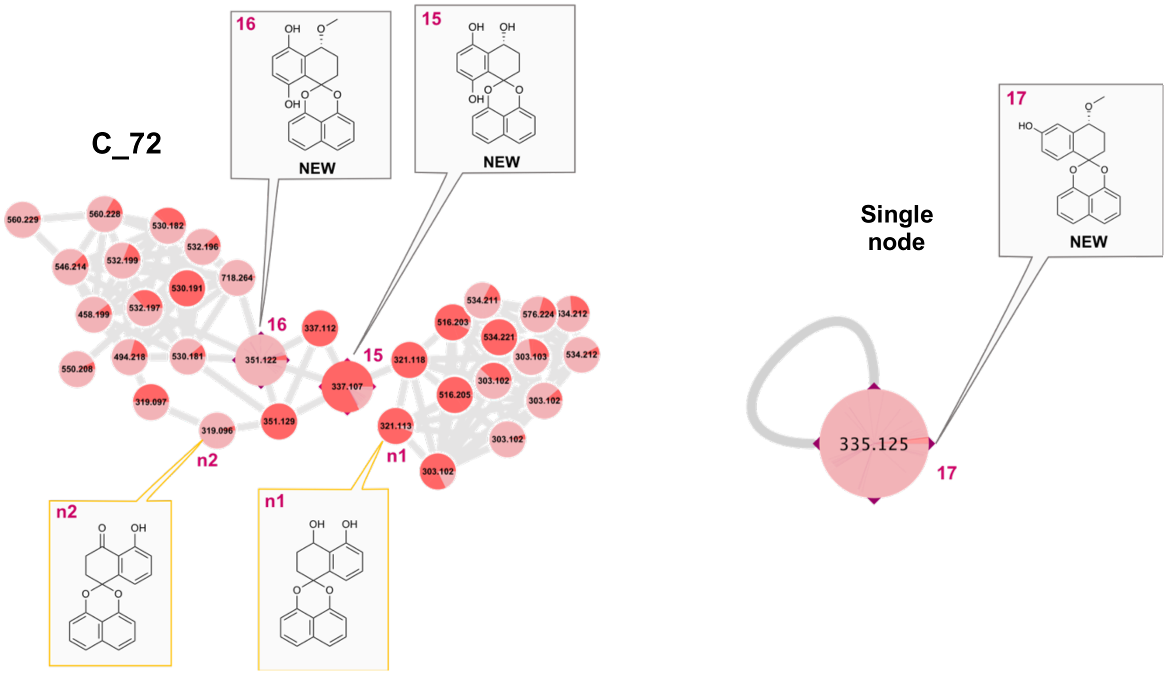

Supplementary Figure 112. Cluster C_72 and single node corresponding to compound **17**

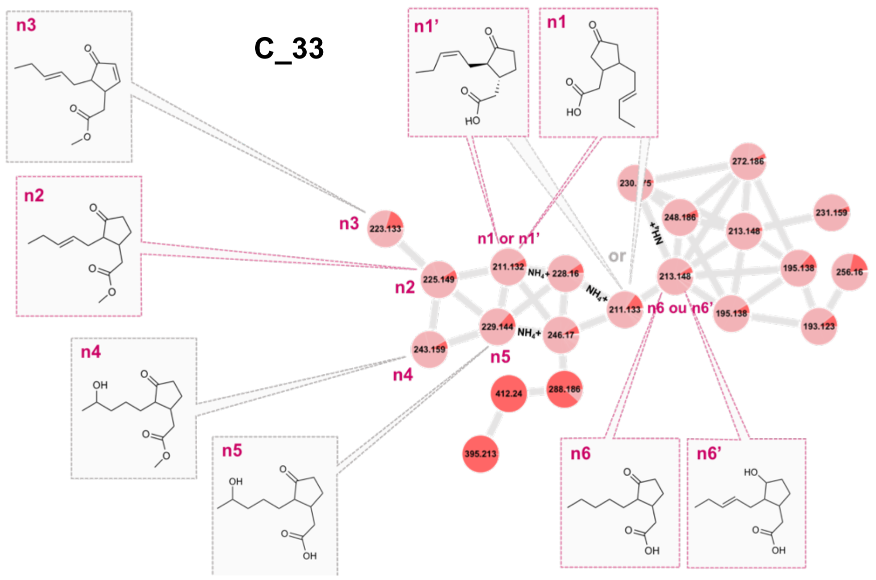

Supplementary Figure 113. Cluster C_33

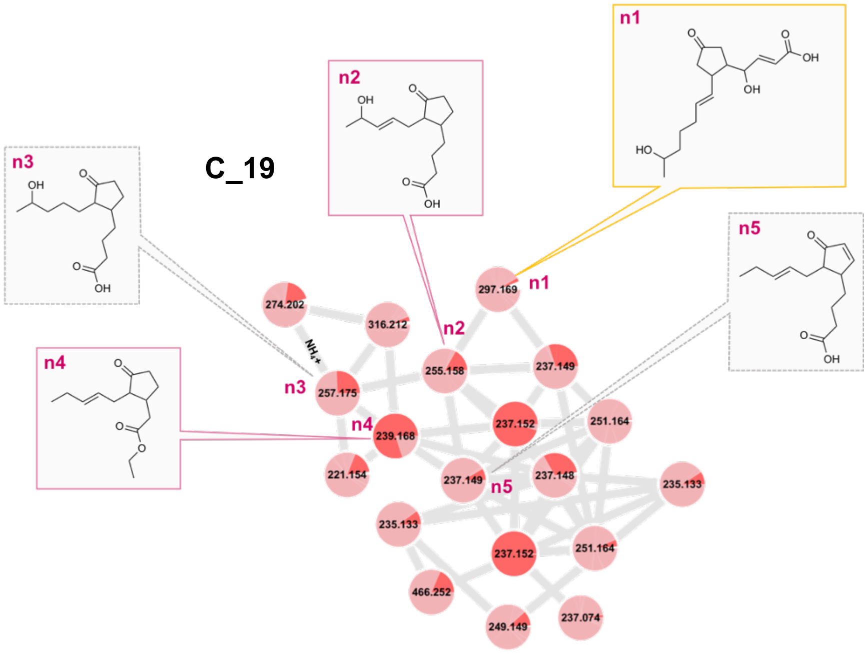

Supplementary Figure 114. Cluster C_19

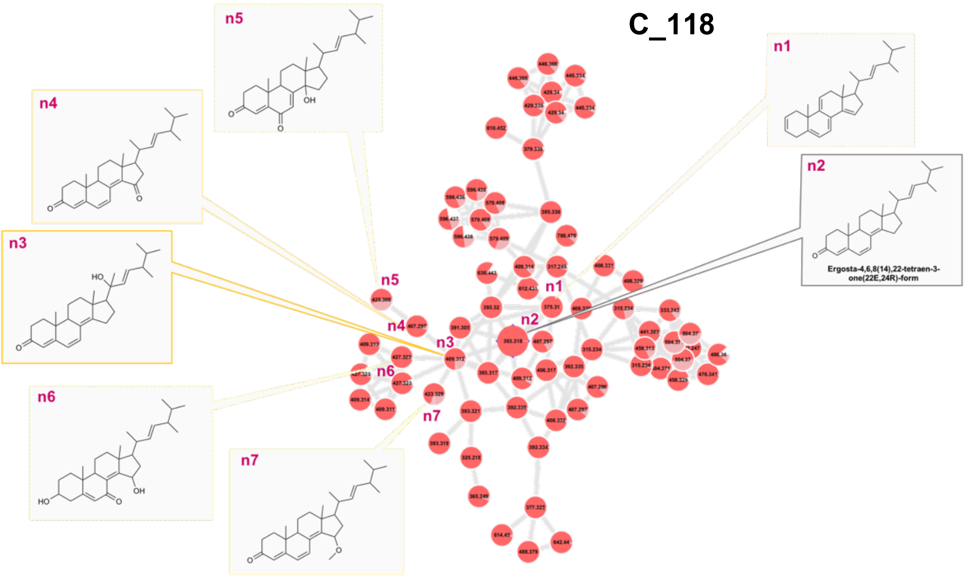

Supplementary Figure 115. Cluster C_118

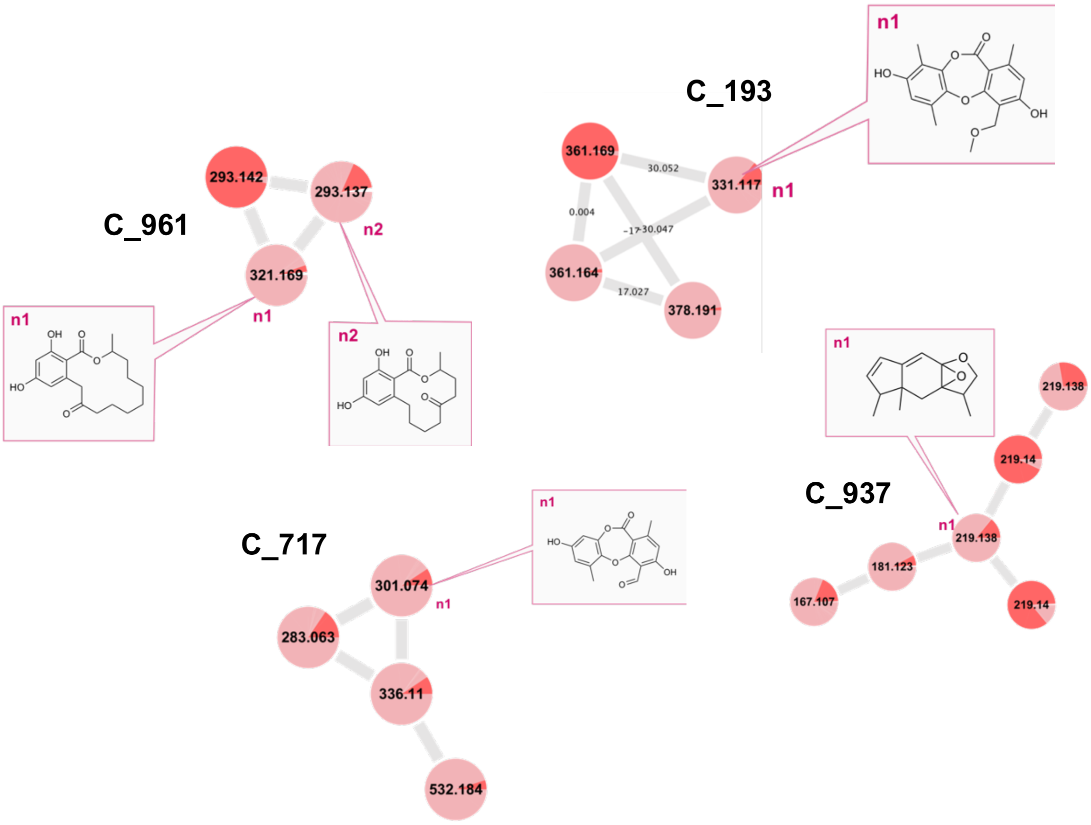

Supplementary Figure 116. Clusters C_961, C_193, C_717, C_937

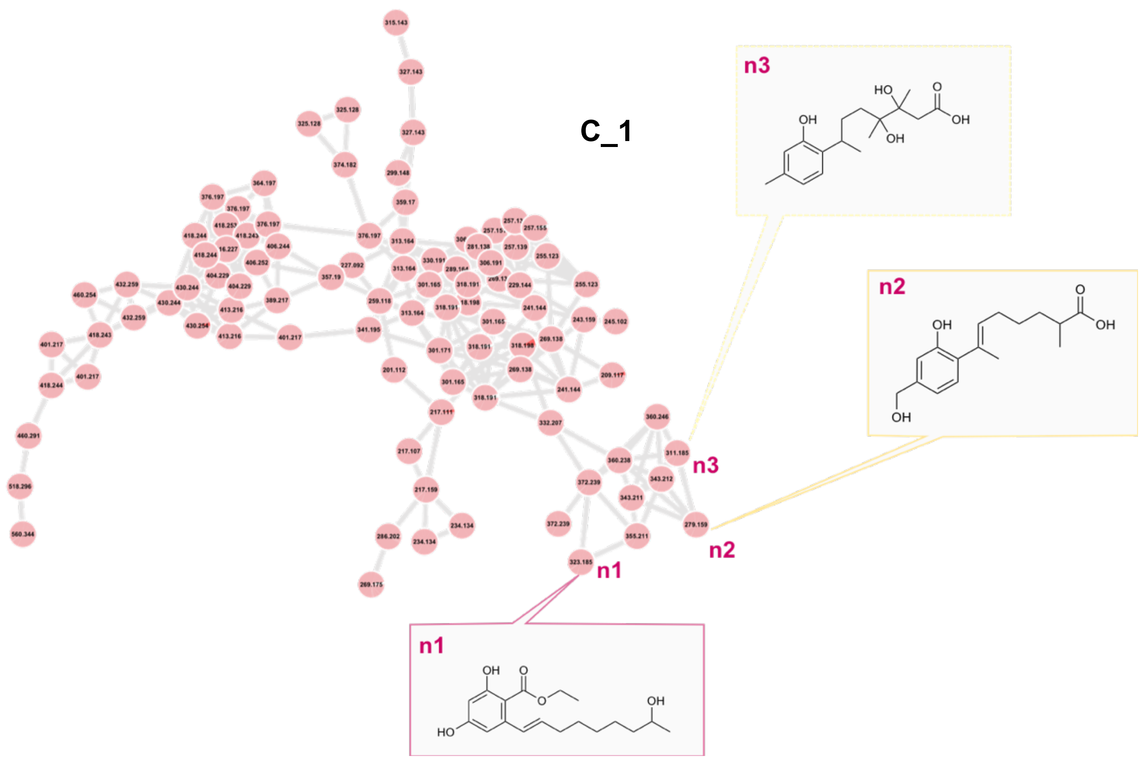

Supplementary Figure 117. Cluster C_1

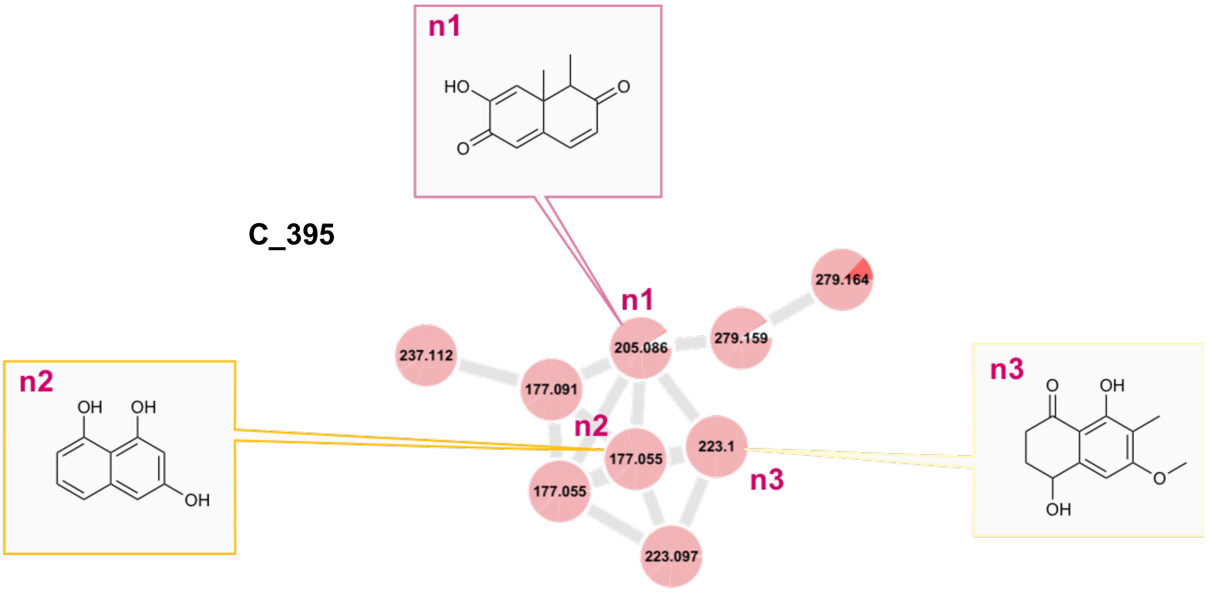

Supplementary Figure 118. Cluster C_395

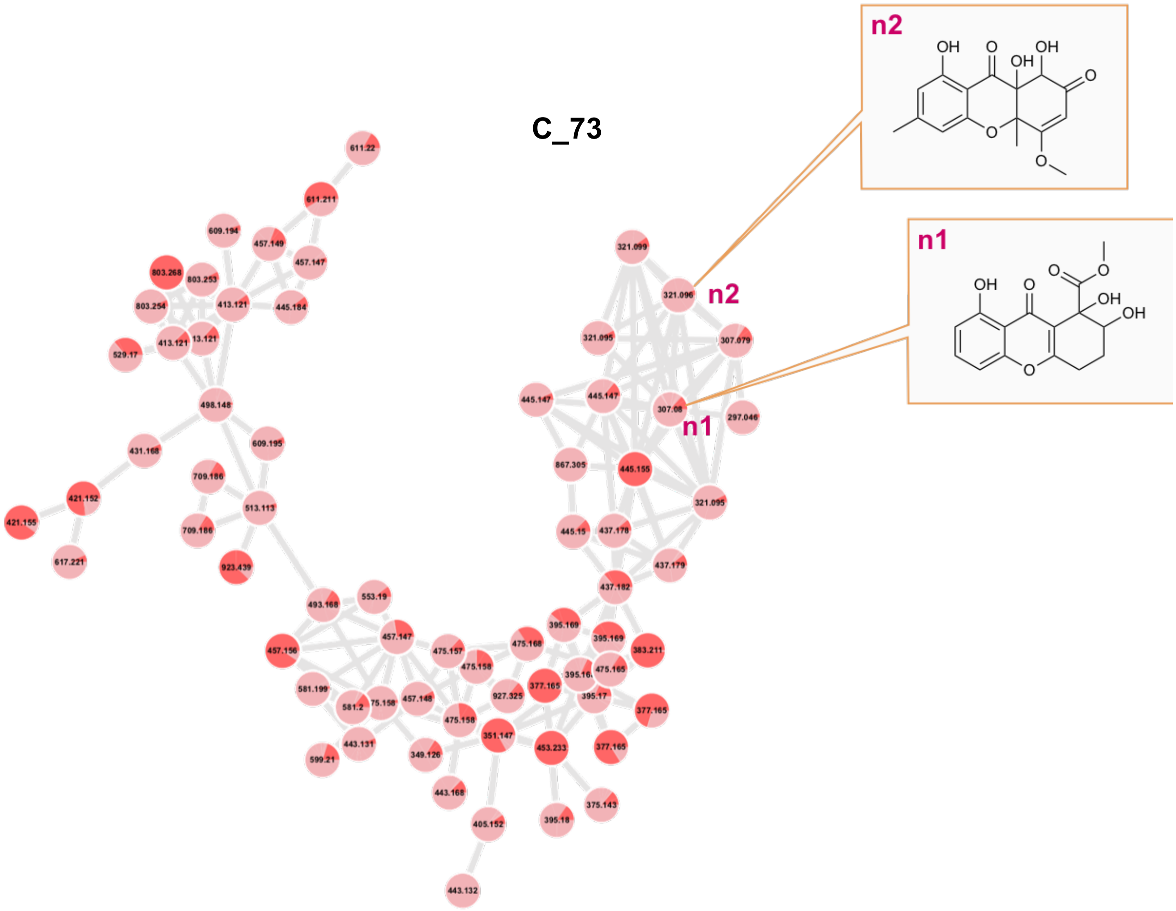

Supplementary Figure 119. Cluster C_73

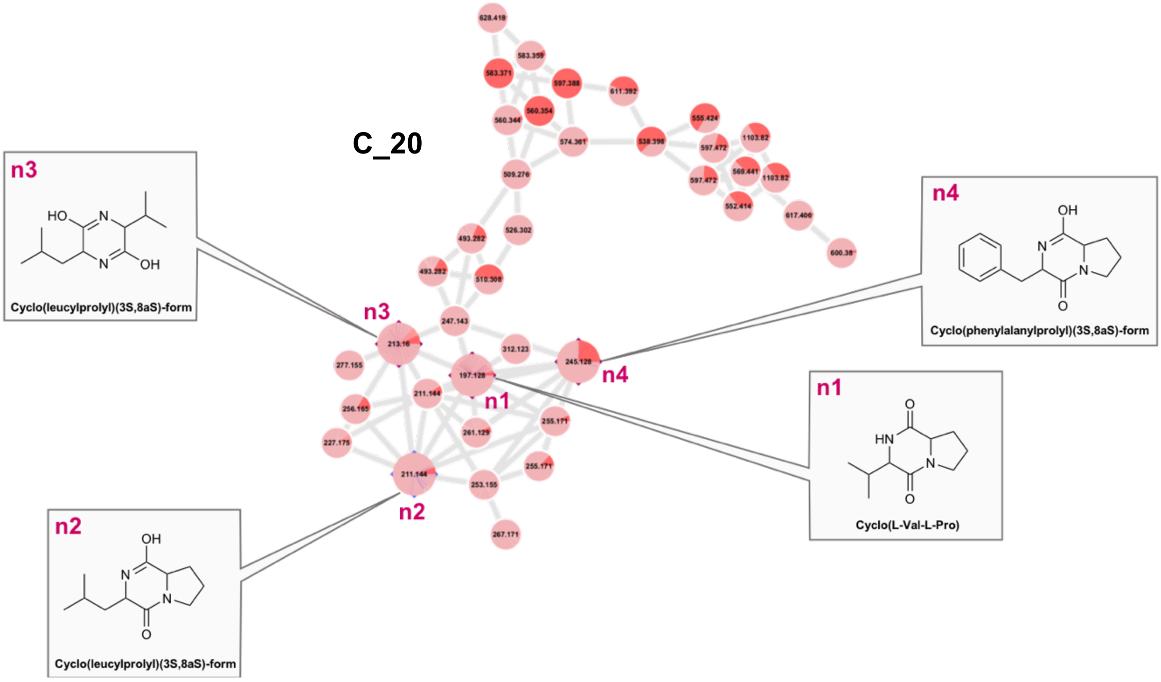

Supplementary Figure 120. Cluster C_20
